# Catalytic Asymmetric Hydration of Alkenes

**DOI:** 10.1021/jacs.6c06916

**Published:** 2026-06-02

**Authors:** Hibiki Hatano, Vitor Alcantara Fernandes, Subrata Mukherjee, Chendan Zhu, Akito Takahira, Pingyu Jiang, Mingoo Jin, Yu Harabuchi, Ruben Staub, Min Gao, Alexandre Varnek, Satoshi Maeda, Nobuya Tsuji, Benjamin List

**Affiliations:** † Graduate School of Chemical Sciences and Engineering, Hokkaido University, Sapporo, Hokkaido 060-8628, Japan; ‡ Institute for Chemical Reaction Design and Discovery (WPI-ICReDD), 12810 Hokkaido University, Sapporo 001-0021, Japan; § 28314 Max-Planck-Institut für Kohlenforschung, D-45470 Mülheim an der Ruhr, Germany; ∥ Laboratory of Chemoinformatics, UMR 7140, CNRS, University of Strasbourg, 67081 Strasbourg, France; ⊥ Department of Chemistry, Graduate School of Science, Hokkaido University, Sapporo, Hokkaido 060-0810, Japan; # ERATO Maeda Artificial Intelligence for Chemical Reaction Design and Discovery Project, Hokkaido University, Sapporo, Hokkaido 060-0810, Japan

## Abstract

The asymmetric Markovnikov hydration of alkenes to alcohols, transforming abundant feedstock chemicals and water into synthetically versatile building blocks, has long remained inaccessible. The reaction is inherently reversible because of unfavorable entropy, preventing asymmetric induction. While lowering the temperature should favor product formation in principle, such conditions are normally incompatible with the required protonation of simple alkenes and with maintaining a homogeneous reaction mixture. Here we show that this long-standing limitation is overcome by making catalytic activity viable under cryogenic conditions. Using confined strong Bro̷nsted acid catalysts and solvent-organized hydrogen-bonding networks that facilitate protonation of otherwise unreactive alkenes, we achieve a highly enantioselective hydration of electronically neutral alkenes with water. This thermodynamic temperature dependence also allows conversion of racemic tertiary alcohols into enantioenriched forms through a one-pot formal deracemization. Experimental and computational studies indicate that perfluorinated solvents form extended hydrogen-bonding networks that enhance both reactivity and stereocontrol. These findings establish a general strategy for overcoming fundamental constraints imposed by reversibility.

## Graphical abstract

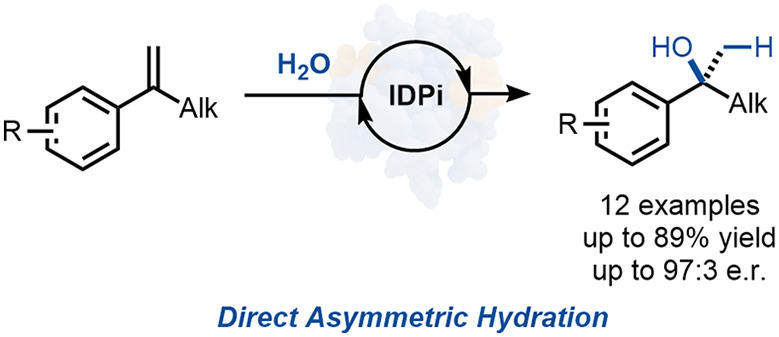

Asymmetric catalysis typically relies on kinetic differentiation between competing transition states. When a reaction is thermodynamically neutral and readily reversible, however, stereochemical information cannot be retained, as microscopic reversibility drives rapid racemization of the product.^1,2^ The hydration of alkenes represents an extreme case of this problem. The key obstacle to asymmetric olefin hydration is its unfavorable thermodynamic balance: the enthalpic gain from forming two new σ-bonds, at the expense of one π-bond and one σ-bond, is compensated by the unfavorable entropy associated with combining two molecules into one. For example, in the case of α-butylstyrene (**1a**), reactants and product have nearly identical free energies at room temperature, rendering asymmetric induction thermodynamically untenable under ambient conditions. Lowering the temperature, however, reduces the entropic contributions, thereby increasing the thermodynamic preference for product formation (Figure 1a). This thermodynamic bias disfavors reversible equilibration and allows stereocontrol to emerge. More broadly, modulation of thermodynamic parameters may bias reversible systems away from racemic equilibria and, in principle, enable net deracemization via condition-dependent cycling between stereoablative elimination (Condition I) and enantioselective hydration (Condition II). In contrast, in conventional asymmetric reactions, product formation is already thermodynamically favored and stereoselectivity is kinetically controlled, such that lowering the temperature often leads to increased selectivity, albeit with diminished conversion. The present system instead benefits from cooling through smaller entropic penalty, resulting in improved chemical yield with suppressed racemization. Achieving this design requires reaction conditions that enable molecular catalysis to activate alkenes while maintaining a homogeneous medium even under cryogenic conditions.

**Figure 1:**
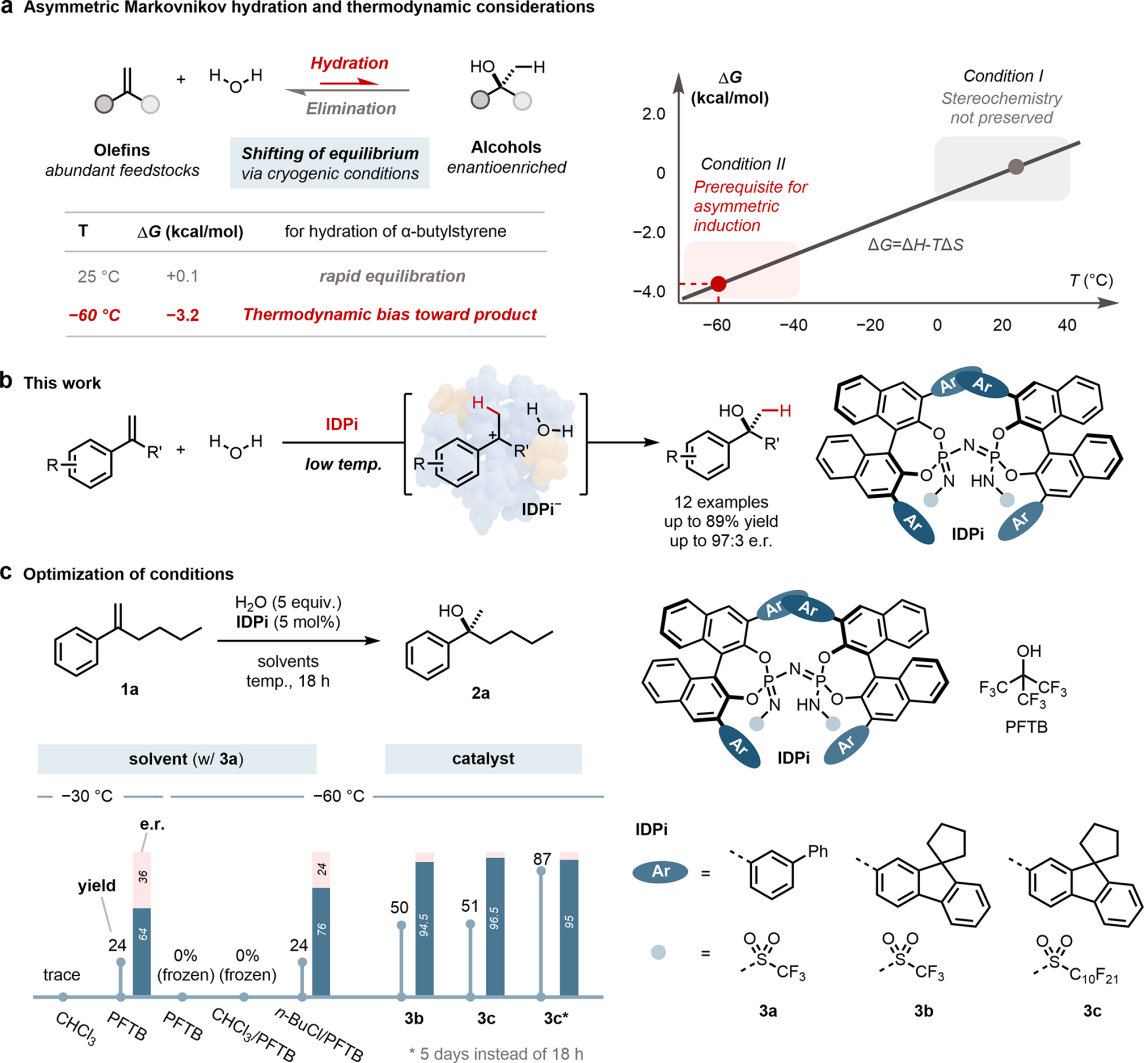
Thermodynamic rationale and development of the asymmetric hydration. (a) Concept of the catalytic asymmetric hydration of alkenes developed in this work and temperature dependence of the reaction free energy (Δ*G*) for the hydration of alkene **1a** to the corresponding tertiary alcohol. Calculations were performed at SMD­(chloroform)-ωB97M-V/def2-TZVPP//r^2^SCAN-3c level of theory. (b) This work: IDPi-catalytic asymmetric hydration of 1,1-disubstituted alkenes under cryogenic conditions. (c) Reaction development.

Water is one of the simplest and most abundant nucleophiles, yet harnessing it in catalytic asymmetric transformations has remained a persistent challenge.^3–5^ In particular, addition of water across carbon–carbon double bonds to enantioenriched alcohols provides the most direct route from alkenes, which are ubiquitous building blocks in nature and industry.^6^ Despite this apparent simplicity, the catalytic asymmetric hydration of simple alkenes has proven a formidable and long-standing goal.^7^ Beyond the thermodynamic constraints described above, practical barriers arise from limitations in both reactivity and selectivity. The alcohol products are typically more reactive than the corresponding alkenes, making control of side reactions challenging. Stereocontrol in the hydration of simple alkenes is intrinsically difficult because these substrates lack coordinating sites beyond the reacting double bond. Moreover, water is a small, three-atom molecule that demands precise spatial organization for enantioselective addition while displaying only moderate nucleophilicity compared with typical alcohols.^8^

Methods capable of addressing these constraints remain scarce. The indirect Mukaiyama hydration provides a widely used solution that circumvents the intrinsic reversibility of the direct hydration, but relies on stoichiometric oxidants and reductants instead of water,^9,10^ and no catalytic asymmetric variant has been reported to date.^11^ An enzymatic approach developed by Hauer and co-workers achieves an asymmetric hydration through the concerted protonation of a terminal olefin and deprotonation of water, representing an elegant alternative approach toward addressing this gap.^12^ While excellent enantioselectivities were obtained across a series of terminal alkenes, multisubstituted alkenes remain beyond the scope.^13^

Recently, several molecular catalysts have emerged that enable stereocontrol in reactions proceeding through carbocationic intermediates.^14–18^ The List group developed imidodiphosphorimidate (IDPi) catalysts, which feature a strongly acidic active site embedded in a highly confined chiral microenvironment,^19^ facilitating a variety of asymmetric hydrofunctionalizations of alkenes,^20–24^ paralleling recent advances in metal-catalyzed enantioselective hydrofunctionalization reactions.^25–31^ However, these acid-catalyzed transformations typically require elevated temperatures to activate the simple alkenes, a condition fundamentally incompatible with the low temperatures required for the asymmetric hydration. More recently, IDPi catalyzed asymmetric polyene cyclizations were shown to benefit from the reactivity enhancement provided by perfluorinated alcohols as solvents.^32^ Despite these advances, IDPi catalysis had not been applied to transformations involving water, whose presence potentially disrupts the ion-pairing environment that governs both reactivity and stereocontrol and prevents formation of homogeneous media at low temperature. Herein, we present a catalytic asymmetric hydration of simple alkenes (Figure 1b). Guided by the thermodynamic profile of the reaction, we identified conditions that enable hydration at cryogenic temperatures and suppress reversibility. Under these conditions, an efficient asymmetric hydration is achieved with high stereoselectivity, enabling a transformation long considered unattainable.

We initiated our study with electronically neutral alkene **1a** to evaluate the feasibility of asymmetric hydration under the low-temperature conditions identified above. Screening of chiral Bro̷nsted acid catalysts revealed that the IDPi catalyst motif was uniquely effective, providing both reactivity and selectivity (Supporting Information Figure S1). While no reaction was observed in chloroform at −30 °C, switching to perfluorinated *tert*-butanol (PFTB) enabled formation of the desired alcohol with promising enantioselectivity (Figure 1c). Further decreasing the reaction temperature, however, caused the solvent to freeze, suppressing reactivity. To maintain a homogeneous reaction mixture at cryogenic temperature, mixtures of PFTB with various organic solvents were therefore examined. Among them, 1-chlorobutane (*n*-BuCl) was identified as the optimal cosolvent, affording the tertiary alcohol in moderate yield and enantioselectivity. Under these conditions, we next examined substituent effects on the IDPi catalyst. A catalyst bearing fluorenyl groups at the 3,3′-positions of the BINOL backbone (**3b**) was found to deliver excellent enantioselectivity. Subsequent modification of the sulfonyl substituents on the nitrogen atoms revealed that perfluorinated long-chain alkyl groups (**3c**) further improved the enantioselectivity. Under the optimized conditions, the desired product was obtained in 87% yield with a 95:5 enantiomeric ratio.

Using the developed procedure, the scope of the asymmetric hydration was explored (Figure 2). Alkenes substituted with different aryl groups (**1a**–**1e**) reacted smoothly to afford the corresponding alcohols in high yields and enantioselectivities, regardless of whether electron-donating or electron-withdrawing substituents were present. The hydration of **1a** was performed on a 100 mg scale, delivering the product in 80% yield and 96.5:3.5 e.r., demonstrating the scalability of this method (Figure S7). Various alkyl-substituted alkenes also showed good reactivity and excellent enantioselectivities, with both short and long linear chains (**2f**–**2h**). Additionally, functionalized substrates, including a halide (**2i**), an alcohol (**2j**), and an ether (**2k**), underwent efficient hydration to provide the desired products in high yields. As a demonstration of the utility of this method, the natural product (−)-Gossonorol^33^ (**2l**) was synthesized successfully in excellent yield and stereoselectivity. Notably, although **2l** contains an additional alkene moiety capable of forming a tertiary carbocation, the reaction selectively activated only the desired double bond, leaving the other site untouched. This remarkable chemoselectivity highlights the potential of our asymmetric hydration for the synthesis of structurally complex and biologically active molecules.

**Figure 2:**
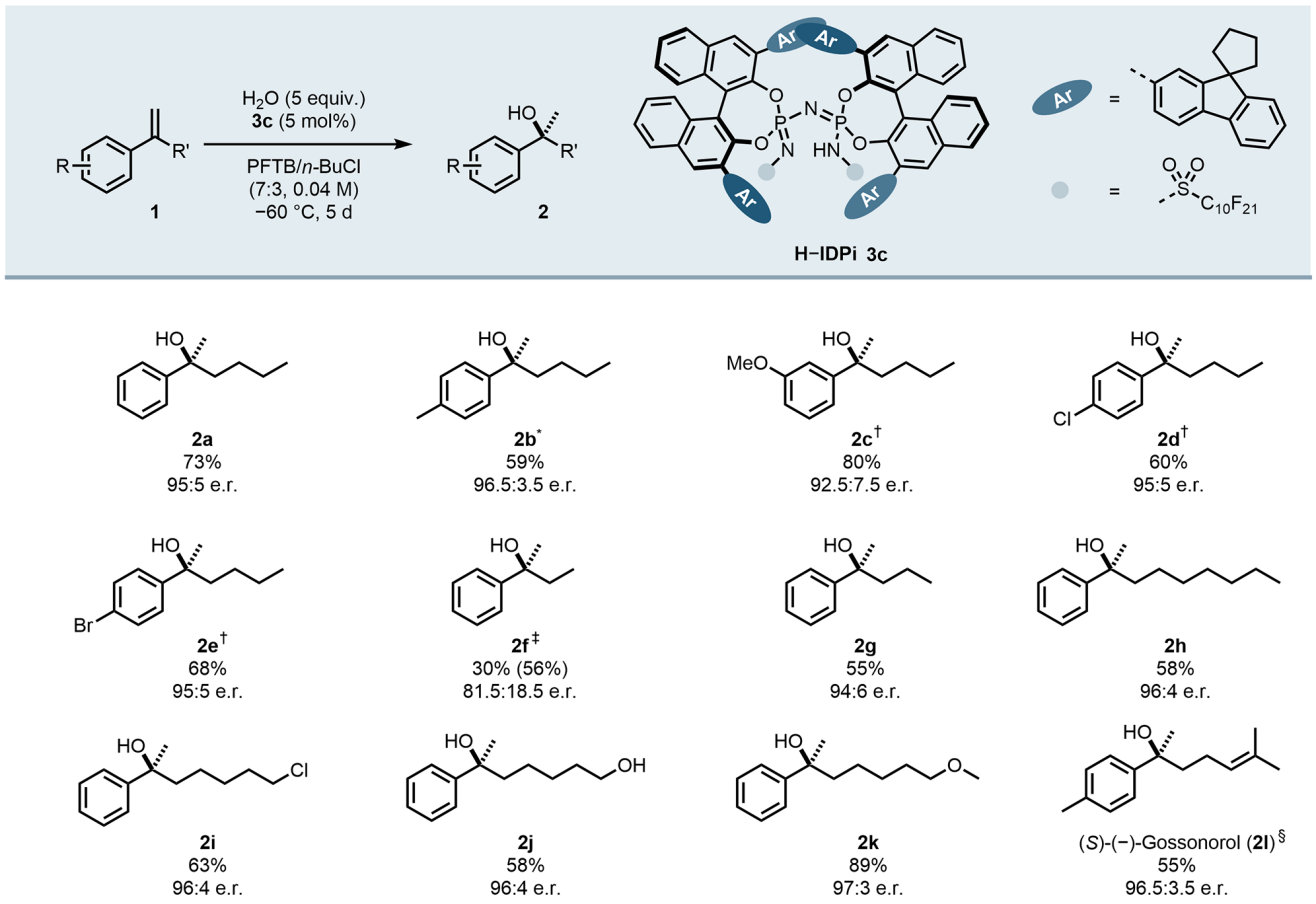
Scope of the catalytic asymmetric hydration of alkenes. Reactions were performed in 0.10 mmol scale. Unless otherwise noted, isolated yields were presented. Asterisk (*) indicates the reaction was performed at −70 °C for 7 days. Dagger (†) symbol indicates the reactions were performed at −40 °C for 3 days. Double dagger symbol (‡) indicates NMR yield is shown in brackets due to volatility of the product. Section symbol (§) indicates the reaction was performed at −70 °C.

We next examined the reversibility of the reaction to assess the temperature dependence of the free-energy landscape. At elevated temperature, treatment of enantioenriched alcohol (*S*)-**2a** with catalyst **3c** produced alkene **1a**, accompanied by substantial racemization of alcohol **2a** and formation of isomeric alkene **4a** (Figure 3a), confirming that the reaction becomes reversible under these conditions. Dehydration is further facilitated by the poor solubility of water in nonpolar solvents. NMR monitoring revealed rapid consumption of alcohol **2a** and formation of alkene **1a**, while the total yield decreased slowly over time (Figure 3b). Based on this behavior, we designed a stepwise formal deracemization strategy. Specifically, racemic alcohols undergo dehydration at room temperature in the presence of the IDPi catalyst, without additional acid-enhancing solvent. Subsequent cooling of the reaction mixture and addition of PFTB and water enables the enantioselective hydration, thereby achieving a formal deracemization (Figure 3c). Unlike existing methods that rely on redox cycles or photoexcitation to overcome microscopic reversibility,^34–39^ this approach employs coordinated control of temperature and solvent composition to convert racemic tertiary alcohols into enantioenriched products. Under optimized conditions, a series of racemic tertiary alcohols were converted into their enantiomerically enriched forms in good yields and enantioselectivities, while a slight decrease in enantioselectivity was observed due to the remaining racemic alcohol in the first step (Figure 3d).

**Figure 3:**
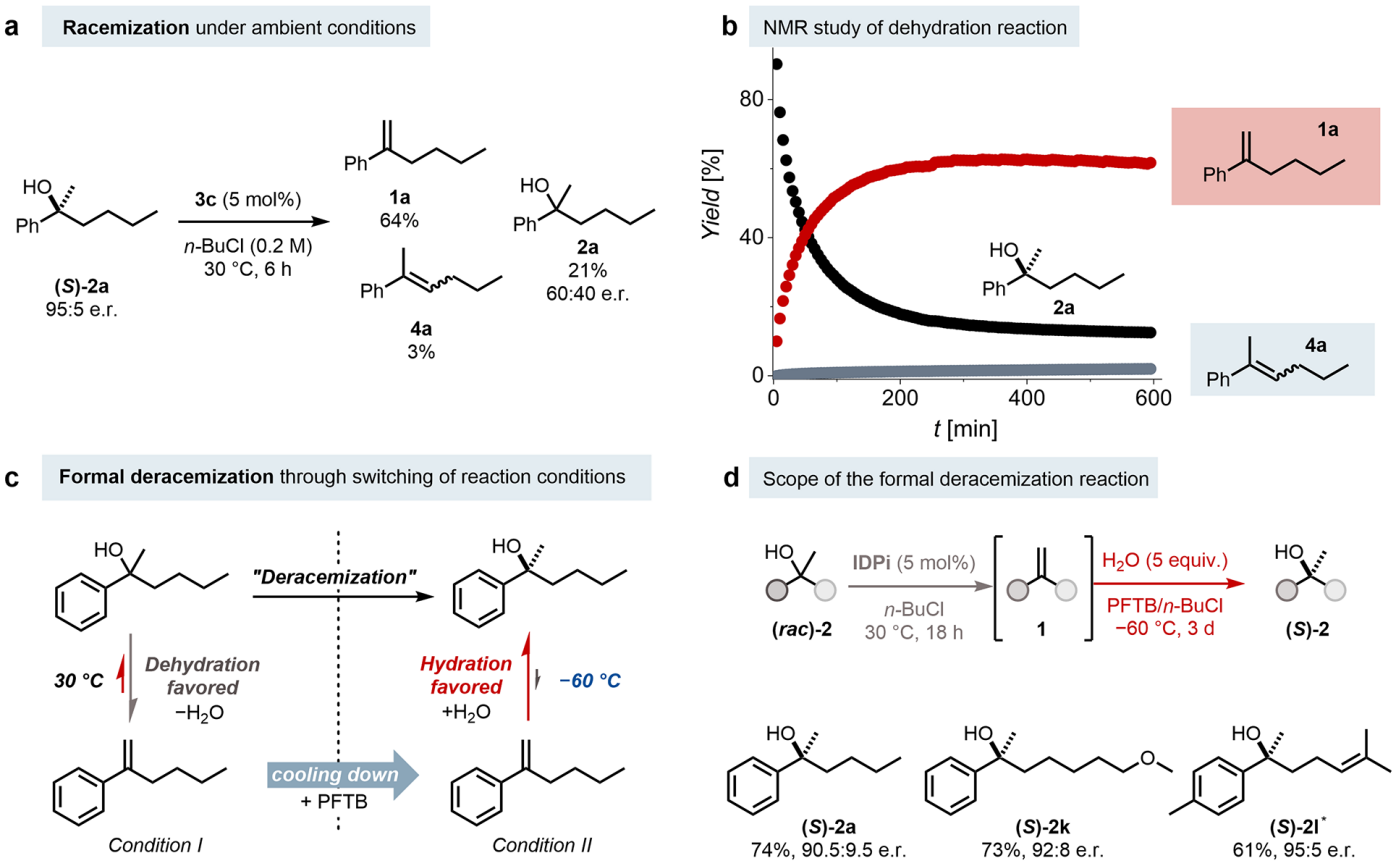
Confirmation of reversibility and its application on temperature-triggered deracemization of tertiary alcohols: (a) Dehydration and racemization of the tertiary alcohols at 30 °C. (b) NMR study of dehydration of **2a** by **3b** in CDCl_3_ at 40 °C. (c) Formal deracemization process by dehydration–hydration sequence through switching of reaction conditions. (d) Scope of formal deracemization of tertiary alcohols. Yields were determined by ^1^H NMR with mesitylene as an internal standard. Asterisk (*) indicates the first step was performed at 0.08 M for 7 h and the second step was carried out at 0.024 M and −65 °C for 12 days.

We outline a plausible mechanism (Figure 4a). In the presence of PFTB, IDPi likely associates with multiple PFTB molecules, enhancing its acidity while stabilizing the corresponding counteranion. Protonation of the olefin at low temperature leads to the formation of the ion pair **INT 1**, which is stabilized by solvation. Subsequent addition of water to the carbocation within a confined microenvironment serves as the enantio-determining step, affording the product and regenerating the IDPi catalyst. Although the reverse reaction can take place at elevated temperature due to entropic contributions, leading to significant racemization, the product remains stable at low temperature even in the presence of a strong acid catalyst.

**Figure 4:**
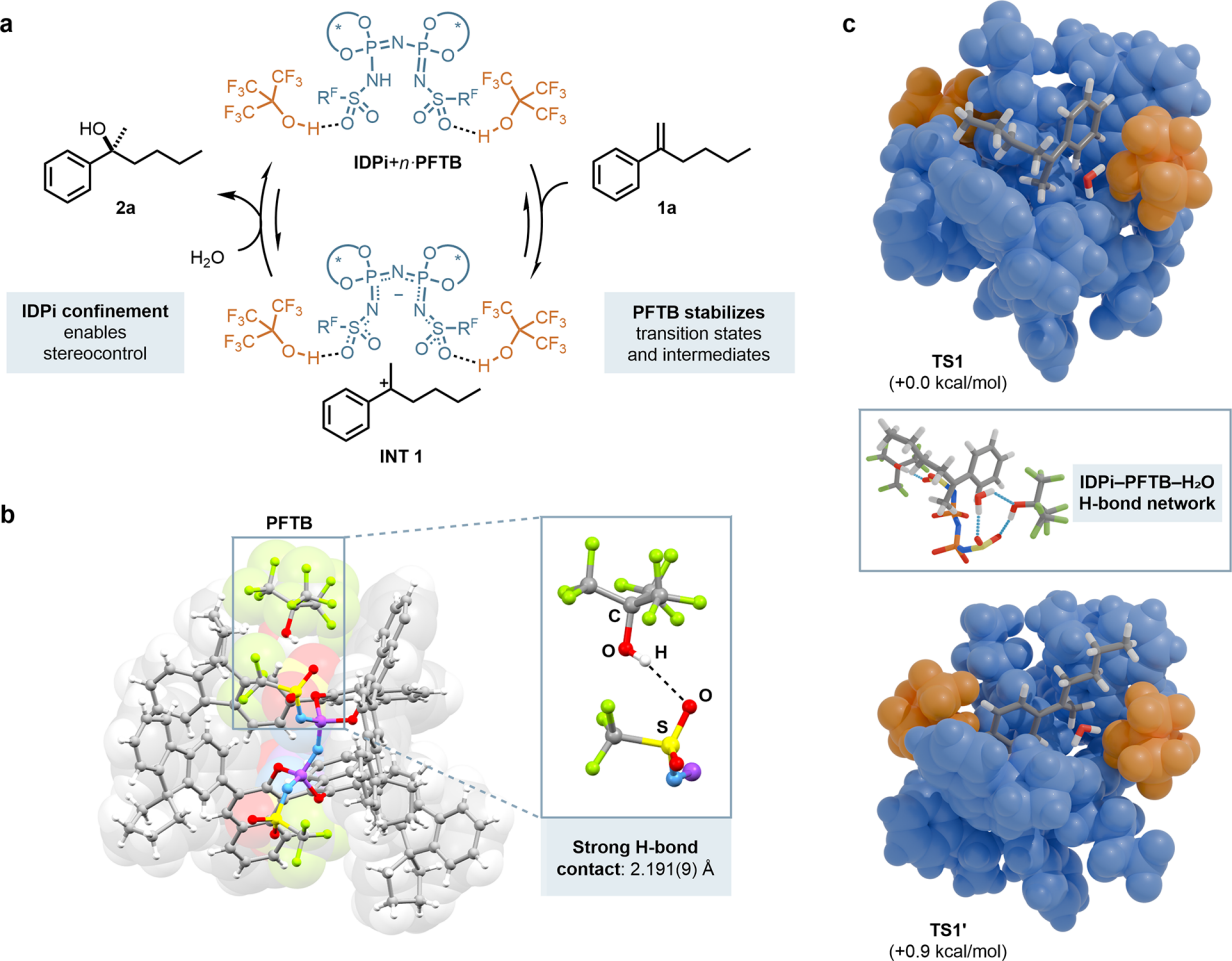
Mechanistic investigations of catalytic asymmetric hydration of alkenes: (a) proposed catalytic cycle. (b) Single-crystal X-ray diffraction structure of the cocrystallized IDPi–PFTB complex. For clarity, solvent molecules and 3-methylpyridine are omitted to highlight the key hydrogen-bonding interaction. (c) Visualization of enantio-determining transition states, calculated at SMD­(2-chlorobutane)-ωB97M-V/def2-TZVPP//r^2^SCAN-3c level of theory. Each Gibbs free energy is shown in brackets. Chiral counteranion is depicted in blue, and PFTB molecules are depicted in orange. The boxed inset highlights the hydrogen-bonding network with light-blue dotted lines. For clarity, only the heteroatoms of the chiral counteranion are shown.

To gain insight into intermolecular structures and interactions between the IDPi and PFTB, we performed cocrystallization screening to yield the single crystal containing the IDPi and PFTB. Single crystals of a cocrystallized IDPi–PFTB complex were obtained by slow diffusion of PFTB into a hexane/chloroform (1:1) solution of the IDPi catalyst in the presence of 3-methylpyridine as an acid–base pairing agent. Single-crystal X-ray diffraction analysis revealed a well-defined O–H···O=S hydrogen bond (2.191(9) Å) between PFTB and the sulfonyl group of the IDPi anion, positioning PFTB in close proximity to the anionic framework (Figure 4b). The presence of PFTB in the solid-state assembly was further supported by FT-IR spectroscopy (Figure S12). This structural arrangement gives rise to a locally confined microenvironment around the Bro̷nsted acidic site, consistent with the proposed counteranion-stabilization and preorganization effects.

To further elucidate the reaction mechanism, we performed computational analyses using water, **1a**, **3d**, and two molecules of PFTB. The corresponding enantio-determining transition states were calculated at the SMD­(2-chlorobutane)-ωB97M-V/def2-TZVPP//r^2^SCAN-3c level of theory (Figure 4c). The estimated ΔΔ*G* value was 0.9 kcal/mol, corresponding to an enantiomeric ratio of 88.5:11.5, which is consistent with the experimental result (1.5 kcal/mol, 97.5:2.5 e.r.; Figure S5). The PFTB molecules interact with the sulfonyl groups of the IDPi anion and with water, forming an extended hydrogen-bonding network that stabilizes the ion pair of the tertiary carbocation intermediate within the pocket. Distortion-interaction analysis^40^ indicates that enantioselectivity originates primarily from catalyst deformation required to accommodate the substrate within the active site (Table S3). The functional group tolerance of the alkyl substituent can also be rationalized by this model: the methyl group points toward the active site, whereas the alkyl group is directed away from it, thereby minimizing steric and electronic effects.

We have shown that asymmetric hydration of simple alkenes with water can be achieved using a confined, strongly acidic chiral Bro̷nsted acid when operated under conditions that suppress the intrinsic thermodynamic reversibility of the reaction. By achieving catalytic turnover under cryogenic conditions that offset the entropic penalty, this work establishes a general strategy for realizing transformations that are otherwise prevented by entropy-dominated thermodynamics. These findings provide a foundation for stereoselective catalysis in reactions constrained by fundamental reversibility.

## Supplementary Material



## Notes

Experimental details and analytical data for all new compounds, NMR spectra, and computational studies (PDF)
