# Supplementary material for: Catalytic Asymmetric Hydration of Alkenes
Source: J Am Chem Soc. 2026 Jun 2;148(23):23481–7. doi: 10.1021/jacs.6c06916 (PMC13281528; doi:10.1021/jacs.6c06916)
Supplement: Supplementary file 1 [file ja6c06916_si_001.pdf]

Supplementary Information

Catalytic Asymmetric Hydration of Alkenes

Authors: Hibiki Hatano, Vitor Alcantara Fernandes, Subrata Mukherjee, Chendan Zhu, Akito Takahira, Pingyu Jiang, Mingoo Jin, Yu Harabuchi, Ruben Staub, Min Gao, Alexandre Varnek, Satoshi Maeda, Nobuya Tsuji*, Benjamin List*

Corresponding author: tsuji@icredd.hokudai.ac.jp (N.T.); list@kofo.mpg.de (B.L.)

Contents

General information 2
Reaction development with different conditions 4
Full Substrate Scope and the Limitations 7
Eyring plot of dehydration 10
Substrate synthesis 12
Racemate synthesis 15
Asymmetric hydration reaction 17
Temperature-triggered deracemization of tertiary alcohols 26
Catalyst preparation 26
NMR spectra 28
HPLC traces 49
Single-crystal X-ray diffraction 64
FTIR spectroscopy: 65
Computational studies 67
References 153

General information

Chemicals

Chemicals were purchased (Aldrich, TCI, FUJIFILM Wako Chemicals, Kanto Chemicals, Oakwood Chemical, Angene Chemical) as reagent grade and used without any further purification.

Solvents

Dry solvents (THF, toluene) were purchased from Kanto and dry solvents (MeCN) was purchased from FUJIFILM Wako. In addition, *n*-BuCl was purchased from TCI and dried over molecular sieves. $CHCl_3$ was purchased from Wako Chemicals and stored over 5Å molecular sieves to remove stabilizer (EtOH).

Inert gas

Dry nitrogen was purchased from Hokkaido Air Water.

Thin Layer Chromatography (TLC) and Column Chromatography (CC)

TLC was performed using silica gel precoated glass sheets (Supelco TLC Silica gel 60 F254) purchased from Sigma-Aldrich. Visualization of TLC was done by irradiation with UV light (λ = 254 nm) or stained using phosphamolybdic acid (PMA). Column chromatography (CC) was carried out using Wako gel (70 Å, particle size 0.020−0.040 mm) using technical grade solvents as eluent. Elution was accelerated using N_2 flow. Alternatively, CC was also done by using Biotage Selekt with Renning cartridges.

Nomenclature

Nomenclature follows the suggestions proposed by the computer program Signals ChemDraw (25.5.0.5789) of Revvity signals software.

Nuclear Magnetic Resonance spectroscopy (NMR)

^{1}H, ^{13}C, ^{19}F, and ^{31}P NMR spectra were recorded on a JEOL NMR JNMECZ400S spectrometer in a suitable deuterated solvent. Data are reported as follows: chemical shift, multiplicity (s = singlet, d = doublet, t = triplet, q = quartet, p = pentet, m = multiplet), coupling constants (Hz) and integration.^{1}H spectra were recorded at 298 K unless otherwise noted, processed with JASON 5.1 program, and coupling constants are reported as observed. ^{1}H chemical shifts (δ) are reported in ppm relative to the protonated solvent resonance employed as the internal standard ($CDCl_3$ δ = 7.26). ^{13}C chemical shifts are reported in ppm with the solvent resonance as the internal standard ($CDCl_3$ δ = 77.16). All spectra are broadband decoupled unless otherwise noted.

Mass Spectrometry (MS)

High resolution mass spectrometry (HRMS) was performed on a JEOL JMS-T2000GC (EI) or Thermo Scientific Exactive Plus (ESI). The ionization method and mode of detection employed is indicated for the respective experiments and all masses are reported in atomic units per elementary charge (m/z) with an intensity normalized to the most intense peak.

Specific Rotations

Specific rotations ($[\alpha]_D^{20}$) were measured with a JASCO P-2200 Polarimeter at the indicated temperature with a sodium lamp (sodium D line, l = 589 nm). Measurements were performed in

an acid resistant 10 mL cell (100 mm length) with described concentrations reported in the corresponding solvent.

Reactor
In case the reaction temperature was lower than room temperature (25 °C), the vials and flasks were cooled using the UCR-150N (TechnoSigma Co., Ltd).

High Performance Liquid Chromatography
High performance liquid chromatography (HPLC) was performed on JASCO PU-4580 liquid chromatograph (AS-4550 auto sampler, DG-4580 degassing unit, MX-4580-D dynamic mixing unit, LC-NetII/ADC interface box, MD-4010 photo diode array detector, CO-4065 column oven) using Daicel columns with a chiral stationary phase. All solvents used were HPLC-grade solvents purchased from FUJIFILM Wako Chemicals.

Ultra High Performance Liquid Chromatography
Ultra High Performance Liquid Chromatography (UPLC) was performed on JASCO PU-4285 liquid chromatograph (AS-4250 auto sampler, LC-NetII/ADC interface box, MD-4010 photo diode array detector, CO-4062 column oven) using Daicel columns with a chiral stationary phase. All solvents used were HPLC-grade solvents purchased from FUJIFILM Wako Chemicals.

Gas Chromatography (GC)
Gas chromatography (GC) was performed on Shimadzu GC-2025 instruments (split-mode capillary injection system, flame ionization detector (FID), hydrogen/nitrogen carrier gas) using CP-Chirasil-DEX CB.

Reaction development with different conditions

We have evaluated a series of chiral Brønsted acid catalysts. Chiral BINOL-phosphoric acid[1, 2] and IDP[3] did not afford any conversion of the substrate under the reaction conditions. The more acidic disulfonimide (DSI)[4] provided some conversion; however, the product was obtained in nearly racemic form, and a significant amount of olefin isomerization was observed. Imino-imidodiphosphate (iIDP)[5] also failed to promote the substrate conversion. In stark contrast, IDPi **3a** furnished the desired product in promising yield and with moderate enantioselectivity. Further, IDPi **3b** and **3c** delivered even higher enantioselectivities, ultimately identifying this catalyst class as the most suitable motif for this transformation.

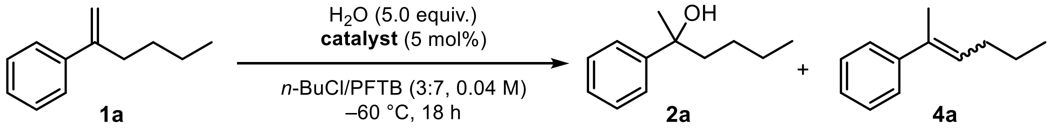

entry	catalyst	**2a** yield (%)	e.r. (**2a**)	**4a** yield (%)
1	(*S*)-**CPA**	0	-	0
2	(*S*)-**IDP**	0	-	0
3	(*R*)-**DSI**	20	50.5:49.5	50
4	(*S*)-**iIDP**	0	-	0
5	**3a**	23	76:24	8
6	**3b**	50	94.5:5.5	5
7	**3c**	40	97:3	4

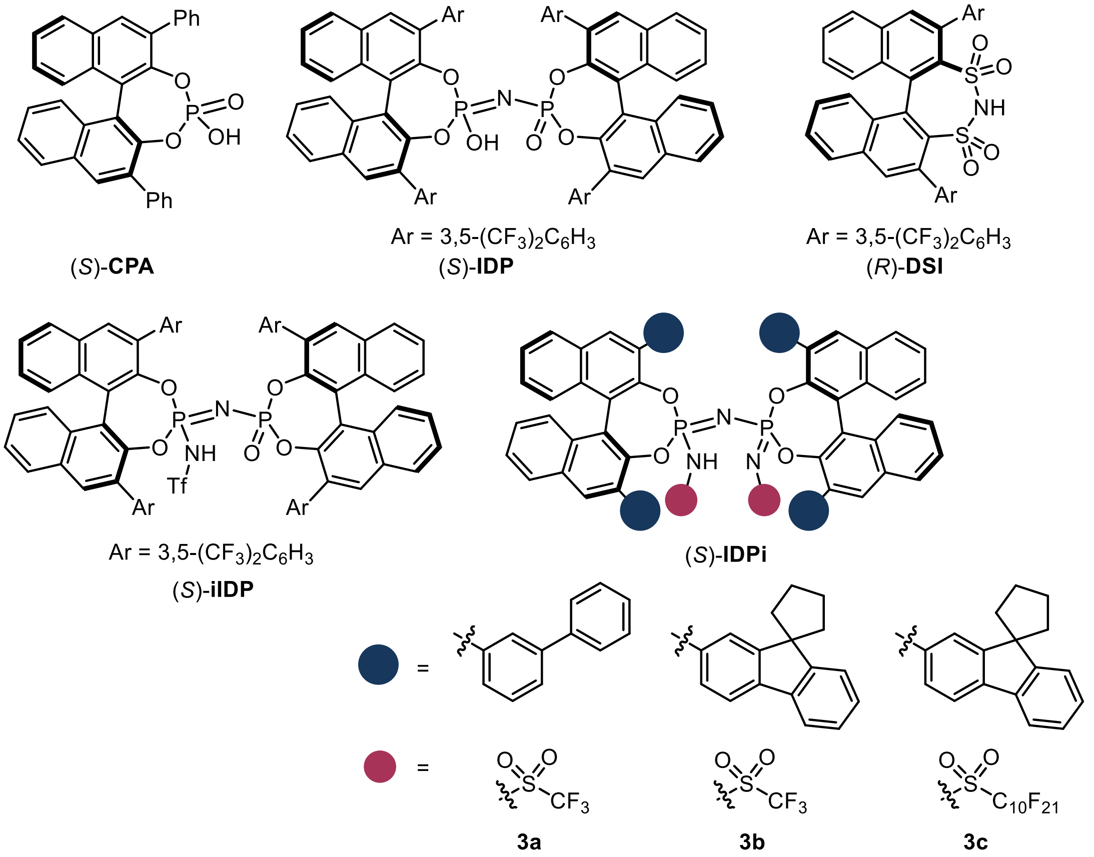

Figure S1. Results of catalyst screening

In addition to the screening results shown in Figure 1c (entries 1, 2, and 5–7), we investigated other conditions using IDPi **3a**. Similar to $CHCl_3$, *n*-BuCl alone was also unable to facilitate protonation presumably due to the lack of the acidity-enhancing effect of perfluorinated alcohol (entry 3). In the presence of PFTB, while the desired products were obtained, significant racemization and decomposition were observed at –30 °C regardless of the presence of *n*-BuCl (entries 2 and 4).

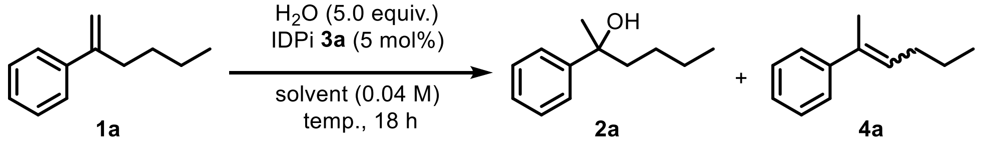

ently	solvents	temp (°C)	**1a** conv. (%)	**2a** yield (%)	e.r. (**2a**)	**4a** yield (%)
1	$CHCl_3$	−30	2	n.d.	-	n.d.
2	PFTB	−30	91	24	36:64	13
3	*n*BuCl	−30	10	n.d.	-	n.d.
4	*n*BuCl/PFTB (3:7)	−30	full	18	48.5:51.5	17
5	PFTB	−60	4	n.d.	-	n.d.
6	$CHCl_3$/PFTB (3:7)	−60	0	n.d.	-	n.d.
7	*n*BuCl/PFTB (3:7)	−60	58	24	24:76	1

Conversions and yields were calculated by ^1H NMR using mesitylene as an internal standard.

Figure S2. Additional screening results with IDPi **3a**

Then, the effects of concentration were evaluated. Compared to the standard conditions (entry 1), at higher concentrations, the chemical yield was decreased, while enantioselectivity was largely unaffected (entries 2 and 3).

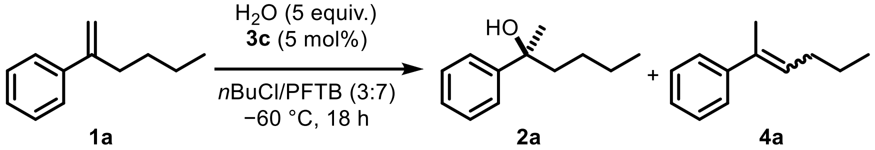

entry	conc. (M)	**1a** conv.(%)	**2a** yield (%)	e.r. (**2a**)	**4a** yield (%)
1	0.04	67	51	3.5:96.5	1
2	0.08	91	39	4:96	1
3	0.16	95	18	4.5:95.5	4

Conversions and yields were calculated by ^1H NMR using mesitylene as an internal standard.

Figure S3. Effect of concentration

Additionally, we have performed a screening evaluating the ratio of PFTB and *n*-BuCl (Figure S4), demonstrating that the PFTB ratio directly affects catalytic activity, while the mixture with *n*-BuCl is necessary to maintain a homogeneous reaction mixture.

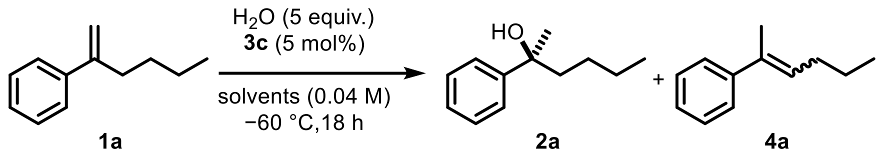

entry	solvents ratio	**1a** conv. (%)	**2a** yield (%)	e.r. (**2a**)	**4a** yield (%)
1	PFTB only	0	n.d.	-	n.d.
2	*n*BuCl/PFTB (2:8)	84	10	3:97	4
3	*n*BuCl/PFTB (3:7)	67	51	3.5:96.5	1
4	*n*BuCl/PFTB (4:6)	63	27	3.5:96.5	1
5	*n*BuCl/PFTB (5:5)	39	22	9:91	4
6	*n*BuCl/PFTB (7:3)	26	trace	-	n.d.
7	*n*BuCl only	7	n.d.	-	n.d.

Conversions and yields were calculated by ^{1}H NMR using mesitylene as an internal standard.

Figure S4. Effect of mixing ratio between *n*-BuCl and PFTB

After establishing the reaction conditions, further optimization was performed. Compared to the standard conditions (entry 2), the use of other fluorinated solvents, including hexafluoroisopropanol (HFIP) and hexafluorophenylpropanol (HFPP), failed to promote the reaction (entries 3 and 4). This is presumably due to their insufficient acidity to further enhance the catalytically active species. In the case of HFIP, its relatively high melting point also posed an additional challenge, as the reaction mixture was completely frozen. To date, PFTB appears to be the only solvent that can form a homogeneous mixture with *n*-BuCl under these conditions. Furthermore, no desired product was observed without PFTB, presumably due to the lack of acidity-enhancing effects (entry 1). Additionally, different co-solvents were investigated. However, other common solvents, including THF, toluene, and pentane, gave inferior results (entries 5–7). While 2-chlorobutane furnished the desired product with comparable enantioselectivity, a greater extent of isomerization was observed (entry 8). The use of catalyst **3d** instead of **3c** resulted in slightly reduced reactivity with comparable enantioselectivity.

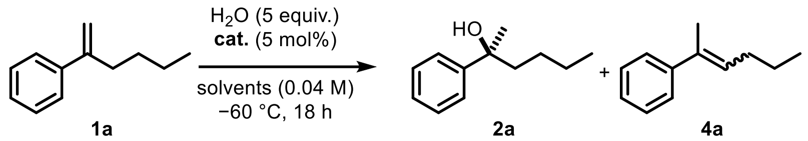

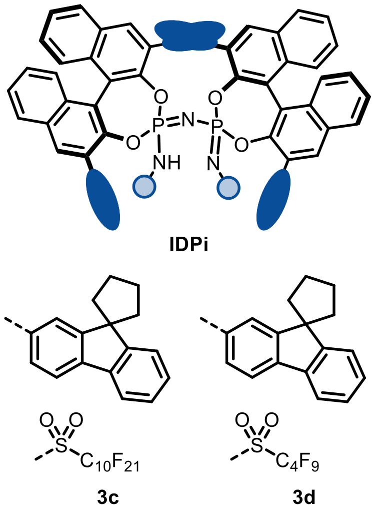

entry	solvents	cat.	**1a** conv. (%)	**2a** yield (%)	e.r. (**2a**)	**4a** yield (%)
1	*n*-BuCl only	**3c**	7	n.d.	-	n.d.
2	*n*-BuCl/PFTB (3:7)	**3c**	67	51	96.5:3.5	1
3	*n*-BuCl/HFIP (3:7)	**3c**	27	n.d.	-	n.d.
4	*n*-BuCl/HFPP (3:7)	**3c**	25	n.d.	-	n.d.
5	THF/PFTB (3:7)	**3c**	27	n.d.	-	n.d.
6	Toluene/PFTB (3:7)	**3c**	39	4	89.5:10.5	n.d.
7	pentane/PFTB (3:7)	**3c**	34	13	93:7	n.d.
8	*sec*-BuCl/PFTB (3:7)	**3c**	84	52	96.5:3.5	25
9	*n*-BuCl/PFTB (3:7)	**3d**	34	19	96:4	trace
10	*sec*-BuCl/PFTB (3:7)	**3d**	68	30	97.5:2.5	4

Yields were calculated by ^{1}H NMR using mesitylene as an internal standard.

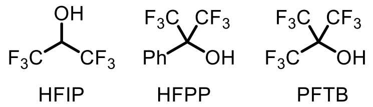

Figure S5. Evaluation of solvents

Next, temperature and reaction time were evaluated (Figure S6). At –50 °C, while the enantioselectivity at 18 h was comparable to that under the optimal conditions, significant racemization was observed upon extending the reaction time. At –40 °C, an even higher degree of racemization and erosion of enantioselectivity were observed.

Temperature and reaction time:

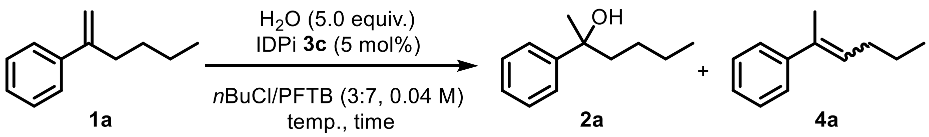

ently	temp (°C)	time (h)	**1a** conv. (%)	**2a** yield (%)	e.r. (**2a**)	**4a** yield (%)
1	−60	18	67	51	3.5:96.5	1
2	−50	18	92	57	4.5:95.5	1
3	−50	54	94	37	10.5:89.5	9
4	−40	18	89	53	10:90	10
5	−40	54	93	36	28.5:71.5	22

Conversions and yields were calculated by ^{1}H NMR using mesitylene as an internal standard.

Figure S6. Additional results for condition screening

Full Substrate Scope and the Limitations

The scope is shown below with conversion, NMR yields of both the desired product and the isomerized olefin to demonstrate the reliability of the isolated yields and mass balance (Figure S7). In some cases, conversion was intentionally moderated to preserve enantioselectivity. In other cases, competing side product formation, primarily olefin isomerization, led to decreased chemical yields.

Additionally, the limitations of the method are also described. The diene substrate was converted into the corresponding allylic alcohol **2m** with good enantioselectivity, albeit with poor chemical yield owing to the formation of unidentified side products, indicating that product decomposition is the primary challenge for this substrate class. In contrast, the dialkyl-substituted olefin suffered from poor reactivity, and the corresponding alcohol **2n** was obtained with moderate enantioselectivity and yield, presumably due to competing olefin isomerization and insufficient carbocation stabilization. These results indicate that while the hydration of non-styrene olefins is kinetically accessible and thermodynamically favorable over the starting olefin, the resulting alcohols are prone to further decomposition via carbocation intermediates to give thermodynamically more stable undesired compounds. With internal olefin **4a** as a substrate, no formation of the corresponding hydration product was detected, and **4a** was recovered largely unchanged. This result suggests that the internal olefin **4a** is significantly less reactive than the *exo*-olefin **1a** under the present conditions, presumably due to a relatively higher kinetic barrier for protonation. When MeOH was employed instead of water, the corresponding methyl ether was obtained in 16% yield with an enantiomeric ratio of 56:44. The lower enantioselectivity may be attributed to the absence of one of the hydrogen bonding interactions, although the steric influence of the introduced alkyl group may also contribute.

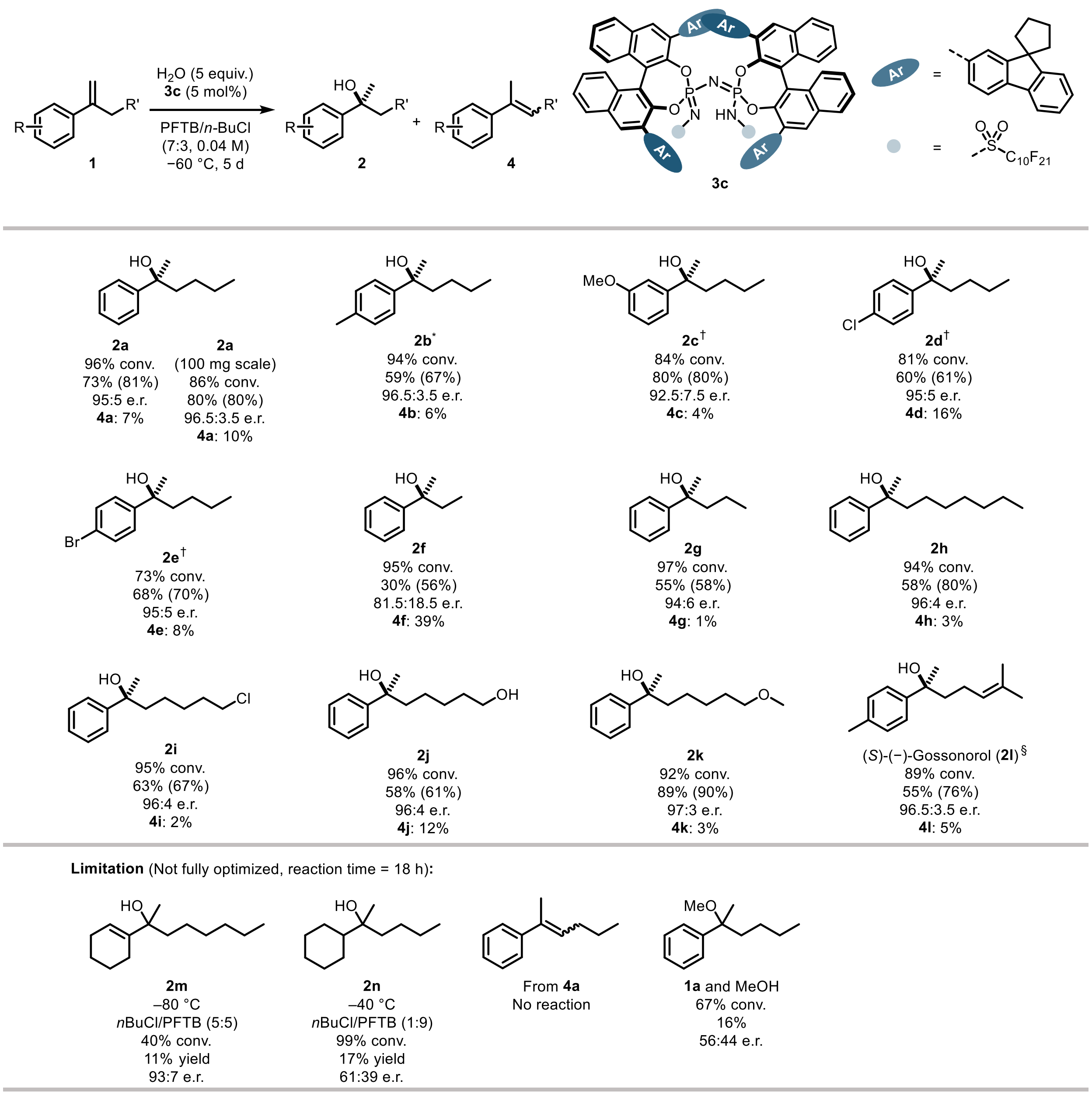

Figure S7. Scope and limitations of the catalytic asymmetric hydration of alkenes with conversion of 1, isolated yield of 2, and NMR yields of 2 and 4: Reactions were performed on a 0.10 mmol scale. Isolated yields are shown with NMR yields in brackets. Conversion is based on the remaining starting material, determined by ^{1}H NMR using dibromomethane as an internal standard. NMR yields of **2** and **4** were also determined by ^{1}H NMR using the same internal standard. For the limitations, reactions were performed on a 9.0 μmol scale, and all yields were determined by ^{1}H NMR using mesitylene as an internal standard. *The reaction was performed at –70 °C for 7 days. †The reactions were performed at –40 °C for 3 days. §The reaction was performed at –70 °C.

To examine whether kinetic resolution occurs during the dehydration reaction, we performed the dehydration of racemic alcohol and evaluated the enantioselectivity of the remaining alcohol

(Figure S8). However, no appreciable enantioenrichment of the recovered alcohol was observed; the recovered alcohol remained essentially racemic within experimental error. This result indicates that kinetic resolution is not significant under these conditions. We attribute this to the highly reversible nature of the transformation under the dehydration conditions, resulting in racemization, as also suggested by the reversibility experiments shown in Figure 3a.

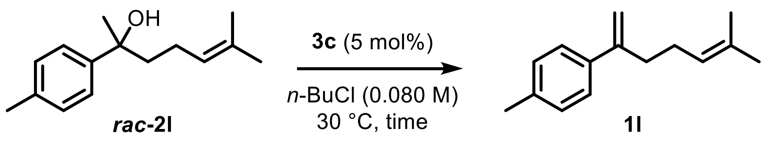

entry	time (min)	**1l** yield (%)	recovered **2l** (%)	e.r. (**2l**)
1	5	17	82	52:48
2	300	88	10	52.5:47.5

Yields were calculated by ^{1}H NMR using mesitylene as an internal standard.

Figure S8. No kinetic resolution during the dehydration reaction

Eyring plot of dehydration

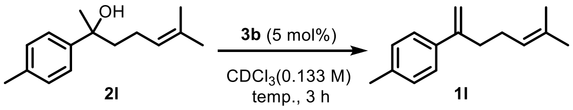

The Eyring plot was constructed according to the previously described protocol[6]. Kinetic measurements were performed at 283.15, 298.15, 303.15, and 313.15 K, and the results at each temperature are summarized in Figure S9. The observed reaction rate constant, kobs, was determined by nonlinear curve fitting using OriginPro 2026 (OriginLab Corporation, Northampton, MA, USA).

The Eyring plot is shown in Figure S10, and the calculated values of Δ*S*, Δ*H*, and Δ*G* at each temperature are summarized in Table S1.

Experimental procedure: IDPi catalyst **3b** (7.12 mg, 3.90 µmol, 0.05 equiv.) and $CDCl_3$ (0.48 mL) were added to an NMR tube, and the mixture was cooled to 0 °C. A solution of **2l** in $CDCl_3$ (0.120 mL, 76.5 µmol, 0.65 M, 1.0 equiv.) and 1,2-dichloroethane (6.0 µL, 76.2 µmol) as an internal standard were then added. The mixture was subsequently warmed to the reaction temperature, and ^{1}H NMR spectra were recorded every 5 min for 3 h.

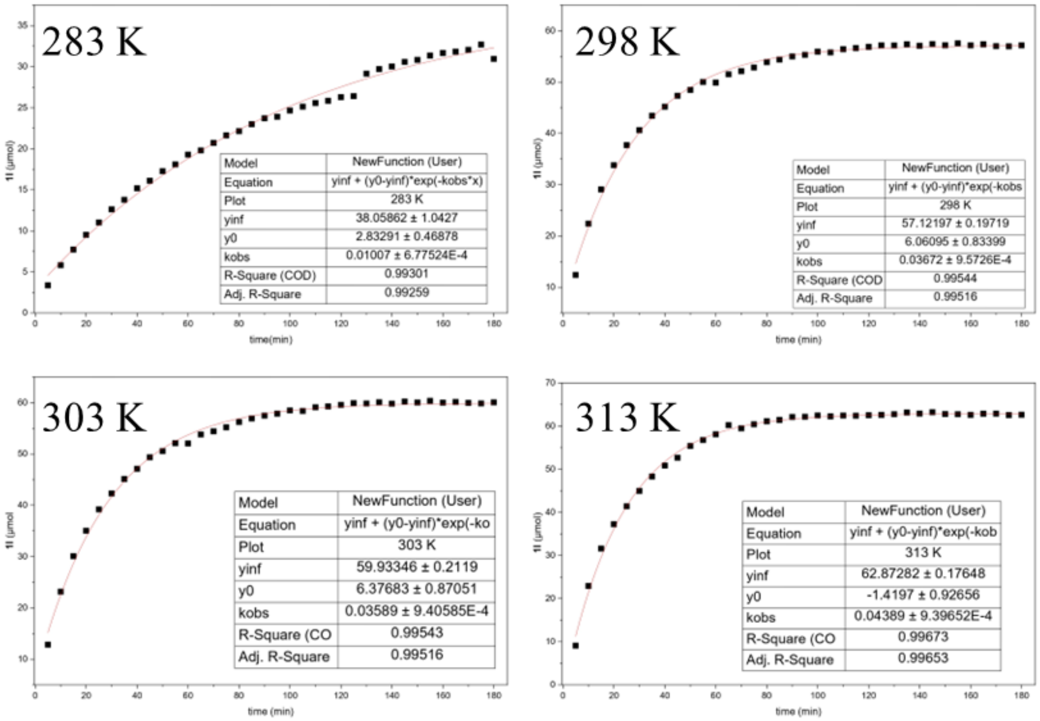

Figure S9. Measurement results of kinetics at 283 K–313 K

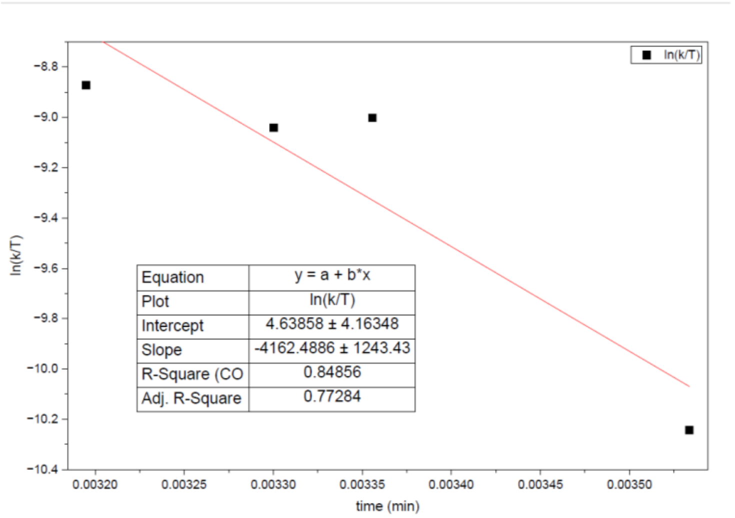

Figure S10. Eyring plot of dehydration

a

1/T	$\ln(k_{obs}/T)$
0.003533569	-10.24364147
0.003355705	-9.00152719973525
0.00330033	-9.04102937933199
0.003194888	-8.87227196582215

ΔH = −9.73 kcal/mol
ΔS = −33.1 cal/mol

b

temp (°C)	ΔG (kcal/mol)
40	20.1
30	19.8
25	19.5
10	19.1

Table S1. Summary of the Eyring analysis (a) Calculated ΔH and ΔS from the Eyring plot (b) Calculated ΔG at various temperatures

Substrate synthesis

Substrate **1a-c**,[7] **1e**,[8] **1f**,[9] **1g**,[7] **1h**,[10] **1l**[11] were synthesized according to **GP1** or reported procedures. The analytical data agreed with the reported data.

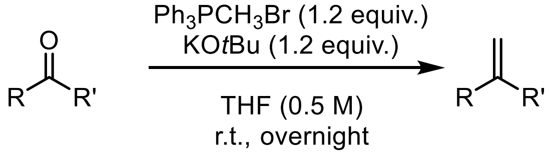

General Procedure 1 (GP 1)

To a Schlenk flask under nitrogen was charged with KO*t*Bu (1.2 equiv.) and Ph_3PCH_3Br (1.2 equiv.). Dry THF (0.5 M) was added to the suspension, and the mixture was stirred at room temperature for 3 h. The corresponding ketone (1.0 equiv.) was then added, and the resulting mixture was stirred overnight. *n*-Hexane was subsequently added to the reaction mixture, and the insoluble materials were filtered off. The liquid phase was purified by flash column chromatography on silica gel.

1-chloro-4-(hex-1-en-2-yl)benzene (1d)

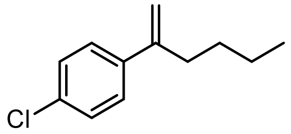

Prepared according to GP1, stirred overnight, eluent: hexane, 1.59 g (84%), colorless oil.

^{1}H NMR (400 MHz, $CDCl_3$) δ7.36 – 7.31 (m, 2H), 7.31 – 7.26 (m, 2H), 5.27 – 5.22 (m, 1H), 5.10 – 5.05 (m, 1H), 2.48 (t, *J* = 7.6 Hz, 2H), 1.48 – 1.27 (m, 4H), 0.91 (t, *J* = 7.2 Hz, 3H).

^{13}C NMR (100 MHz, $CDCl_3$) δ 174.7, 140.0, 133.1, 128.5, 127.5, 112.7, 35.1, 30.4, 22.5, 14.1.

HRMS m/z (EI): calculated for $C_{12}H_{15}Cl$ $[M]^+$: 194.08568, found 194.08525.

(7-chlorohept-1-en-2-yl)benzene (1i)

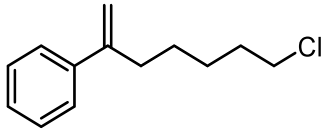

To a Schlenk flask under nitrogen was added triphenylphosphine (1.39 g, 5.30 mmol, 1.12 equiv.), dry THF (12 mL), and *N*-chlorosuccinimide (715 mg, 5.36 mmol, 1.13 equiv.). Subsequently, a solution of 6-phenylhept-6-en-1-ol (**1j**, 899 mg, 4.72 mmol, 1.00 equiv.) in THF (5.7 mL) was added, and the mixture was stirred overnight. After addition of saturated NH_4Cl aqueous solution, the aqueous phase was extracted with Et_2O twice. The combined organic layers were washed with brine, dried over anhydrous Na_2SO_4, filtrated, and concentrated under reduced pressure. The residue was purified by column chromatography on SiO_2 using Et_2O/hexane = 0-30% as eluent to afford the desired product as a colorless oil (844 mg, 86%).

^{1}H NMR (400 MHz, CDCl$_3$) δ 7.42 – 7.37 (m, 2H), 7.35 – 7.29 (m, 2H), 7.29 – 7.23 (m, 1H), 5.26 (d, *J* = 1.4 Hz, 1H), 5.07 – 5.03 (m, 1H), 3.50 (t, *J* = 6.7 Hz, 2H), 2.56 – 2.47 (m, 2H), 1.81 – 1.71 (m, 2H), 1.43 –1.50 (m, 4H).

^{13}C NMR (100 MHz, CDCl$_3$) δ 148.4, 141.3, 128.4, 127.4, 126.2, 112.5, 45.1, 35.3, 32.5, 27.5, 26.6.

The analytical data were consistent with the reported data.[12]

Methyl 6-phenylhept-6-enoate (S-1a)

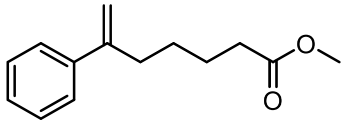

Prepared according to GP1, stirred for 18 h in 0.2 M THF, eluent: Et_2O/hexane= 0-5%, 7.46 g (79%), colorless solid.

^{1}H NMR (400 MHz, CDCl$_3$) δ 7.41 – 7.35 (m, 2H), 7.35 – 7.28 (m, 2H), 7.28 – 7.22 (m, 1H), 5.26 (s, 1H), 5.05 (s, 1H), 3.64 (s, 3H), 2.51 (t, *J* = 7.6 Hz, 2H), 2.29 (t, *J* = 7.6 Hz, 2H), 1.70 – 1.60 (m, 2H), 1.52 – 1.42 (m, 2H).

^{13}C NMR (100 MHz, CDCl$_3$) δ 174.2, 148.2, 141.2, 128.4, 127.4, 126.2, 112.6, 51.6, 35.0, 34.0, 27.7, 24.7.

HRMS m/z (ESI): calculated for $C_{14}H_{18}O_2Na$ $[M+Na]^+$: 241.11990, found 241.11937.

6-phenylhept-6-en-1-ol (1j)

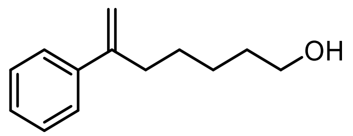

To a Schlenk flask under nitrogen with $LiAlH_4$ (2.26 g, 59.6 mmol, 2.00 equiv.) was added dry THF (250 mL). Subsequently, the stock solution of methyl 6-phenylhept-6-enoate in THF (44.1 mL) was added dropwise at –78 °C and the reaction mixture was then warmed to room temperature and stirred overnight.

After addition of water (2.2 mL), 15% NaOH (aq) (2.2 mL), water (6.6 mL) and Na_2SO_4, the mixture was filtered off through a short pad of SiO_2 and washed with Et_2O to afford the desired product as a colorless oil (5.72g, quant.).

^{1}H NMR (400 MHz, CDCl$_3$) δ 7.45 – 7.37 (m, 2H), 7.36 – 7.29 (m, 2H), 7.28 – 7.21 (m, 1H), 5.26 (s, 1H), 5.05 (s, 1H), 3.60 (t, *J* = 6.5 Hz, 2H), 2.57 – 2.46 (m, 2H), 1.62 – 1.32 (m, 7H).

^{13}C NMR (100 MHz, CDCl$_3$) δ 148.6, 141.4, 128.4, 127.4, 126.2, 112.4, 63.0, 35.4, 32.6, 28.1, 25.5.

The analytical data were consistent with the reported data.[13]

(7-methoxyhept-1-en-2-yl)benzene (1k)

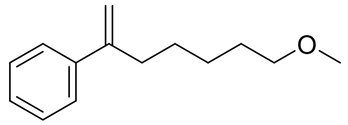

To an oven-dried Schlenk flask under nitrogen with sodium hydride (60% dispersion in paraffin liquid, 420 mg, 10.5 mmol, 2.00 equiv.) was added dry THF (2.8 mL). Then, a solution of 6-phenylhept-6-en-1-ol (**1j**) (1.00 g, 5.26 mmol, 1.0 equiv.) in THF (2.6 mL) was added, dropwise. This mixture was stirred at room temperature for one hour. Next, MeI (1.31 mL, 21.0 mmol, 4.00 equiv.) was added and the reaction mixture was stirred for 16 hours. After addition of water (10 mL), the aqueous phase was extracted with EtOAc (3x30 mL). The combined organic layers were washed with brine, dried over anhydrous Na_2SO_4 and concentrated under reduced pressure. The residue was purified by column chromatography on SiO_2 using EtOAc/hexanes = 0% to 4% as eluent to afford the desired product as a colorless oil (0.90 g, 84%).

^{1}H NMR (400 MHz, CDCl$_3$) δ 7.42 – 7.37 (m, 2H), 7.36 – 7.29 (m, 2H), 7.29 – 7.23 (m, 1H), 5.26 (d, *J* = 1.3 Hz, 1H), 5.05 (d, *J* = 1.3 Hz, 1H), 3.35 (t, *J* = 6.6 Hz, 2H), 3.32 (s, 3H), 2.56 – 2.46 (m, 2H), 1.62 – 1.53 (m, 2H), 1.52 – 1.32 (m, 4H).

^{13}C NMR (100 MHz, CDCl$_3$) δ 148.7, 141.5, 128.4, 127.4, 126.2, 112.3, 73.0, 58.7, 35.4, 29.6, 28.2, 26.0.

HRMS m/z (ESI): calculated for $C_{14}H_{21}O$ $[M+H]^+$: 205.15869, found 205.15843.

Racemate synthesis

General Procedure (GP2)

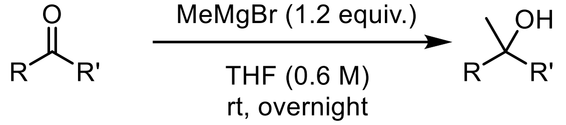

A Schlenk flask under nitrogen was charged with ketone (1.0 equiv.) and dry THF (0.6 M), MeMgBr solution (3.0 M in Et_2O, 1.2 equiv.) was then added dropwise, and the resulting mixture was stirred overnight. The reaction was quenched by addition of saturated aqueous NH_4Cl, followed by extraction of the aqueous phase with Et_2O three times. The combined organic layers were washed with brine, dried over anhydrous Na_2SO_4, filtered, and concentrated under reduced pressure. The crude mixture was purified by flash column chromatography on silica gel.

Unless otherwise noted, the racemates were prepared according to **GP2**.

Analytical data is consistent with the enantiomerically enriched samples.

The racemates of **2j** and **2k** were prepared according to the following scheme.

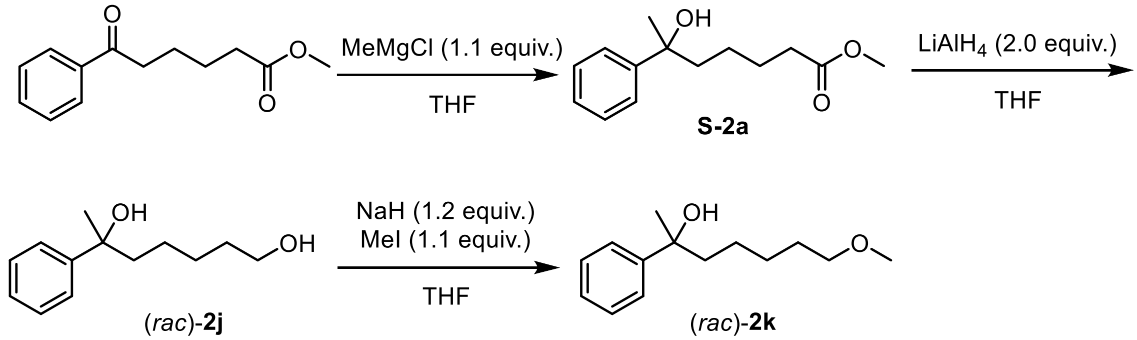

Methyl 6-hydroxy-6-phenylheptanoate (S-2a)

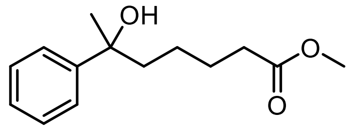

To a Schlenk flask under nitrogen with methyl 6-oxo-6-phenylhexanoate (6.99 g, 31.7 mmol, 1.0 equiv.) was added dry THF (158 mL) and MeMgCl solution (3.0 M in Et_2O, 12.0 mL, 36.0 mmol, 1.1 equiv.) at 0 °C. The reaction mixture was then warmed to room temperature and stirred for 20 min. After addition of saturated NH_4Cl aqueous solution, the aqueous phase was extracted with Et_2O twice. The combined organic layers were washed with brine, dried over anhydrous Na_2SO_4, filtrated, and concentrated under reduced pressure. The residue was purified by column chromatography on SiO_2 using Et_2O/hexane = 0-60% as eluent to afford the desired product as a colorless oil (6.17 g, 82%).

^{1}H NMR (400 MHz, $CDCl_3$) δ 7.47 – 7.36 (m, 2H), 7.36 – 7.26 (m, 2H), 7.26 – 7.19 (m, 1H), 3.62 (s, 3H), 2.24 (t, J = 7.5 Hz, 2H), 1.91 – 1.71 (m, 2H), 1.69 (s, 1H), 1.67 – 1.50 (m, 5H), 1.37 – 1.23 (m, 1H), 1.22 - 1.08 (m, 1H).

^{13}C NMR (100 MHz, $CDCl_3$) δ 174.2, 148.0, 128.2, 126.6, 124.8 , 74.6, 51.6, 43.8, 34.0, 30.3, 25.3, 23.6.

Analytical data is consistent with the reported values.[14]

6-phenylheptane-1,6-diol ((*rac*)-2j)

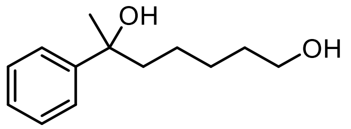

To a Schlenk flask under nitrogen with $LiAlH_4$ (968 mg, 25.5 mmol, 2.0 equiv.) was added dry THF (50.0 mL). Subsequently, a solution of methyl 6-hydroxy-6-phenylheptanoate (3.00 g, 12.7 mmol, 1.0 equiv.) in dry THF (13.0 mL) was added dropwise at 0 °C. The reaction mixture was then warmed to room temperature and stirred for 5 h. After addition of water (1.0 mL), 15% NaOH (aq) (1.0 mL), water (3.0 mL), and Na_2SO_4. The mixture was filtered off through a short pad of SiO_2 and washed with Et_2O to afford the desired product as a colorless oil (2.46 g, 93%).

7-methoxy-2-phenylheptan-2-ol ((*rac*)-2k)

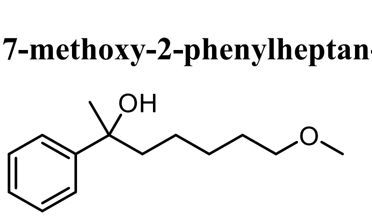

To a Schlenk flask under nitrogen with sodium hydride (60%, dispersion in paraffin liquid, 280 mg, 7.00 mmol, 1.21 equiv.) was added dry THF (5.8 mL) and 6-phenylheptane-1,6-diol ((*rac)*-

2j) (1.21 g, 5.80 mmol, 1.0 equiv.). Subsequently, MeI (0.399 mL, 6.37 mmol, 1.1 equiv.) was added and the reaction mixture was stirred for 3 hours at room temperature. After addition of saturated NH_4Cl solution the aqueous phase was extracted with Et_2O two times. The combined organic layers were washed with brine, dried over anhydrous Na_2SO_4 and concentrated under reduced pressure. The residue was purified by column chromatography on SiO_2 using Et_2O/hexane = 10-60% as eluent to afford the desired product as a colorless oil (757 mg, 59%).

Asymmetric hydration reaction

General procedure for asymmetric hydration reaction

To IDPi (13.6 mg, 5.00 μmol, 0.05 equiv.) in a 35 ml Schlenk flask were added 1-chlorobutane (250 μL), nonafluoro-*tert*-butyl alcohol (1.75 mL) and water (9.0 μL, 5 equiv.) at room temperature. The mixture was cooled to the reaction temperature and stirred for 10 min. Next, alkene solution (500 μL, 0.10 mmol, 0.2 M in 1-chlorobutane, 1 equiv.) was added, dropwise, to the reaction mixture. After stirring for the specified duration, the mixture was quenched with triethylamine (50 μL) and stirred at the same temperature for 10 min. Cooling was removed, the mixture was allowed to warm up, then dibromomethane (7.0 μL, 0.10 mmol) was added. An aliquot (100 μL) was taken, and dissolved in $CDCl_3$ (500 μL), for conversion determination by ^{1}H NMR. Solvents were removed under reduced pressure. Purification by column chromatography with silica gel (Rening 5g) using 3-10% Et_2O/hexane or 5-15% ether/pentane as an eluent afforded the product as a colorless oil.

(*S*)-2-phenylhexan-2-ol (2a)

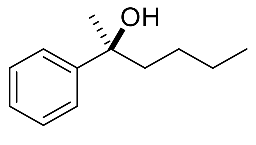

0.1 mmol scale, −60 °C for 5 days, 13.0 mg, 73%, a colorless oil.

e.r. = 95:5

The enantiomeric excess was determined by chiral HPLC analysis on IJ-3 column. Conditions: isopropanol/*n*-hexane = 6:94, flow rate = 0.3 mL/min, oven temperature = 40 °C, UV-vis detection at λ = 210 nm, t_{R1} = 15.413 min (minor), t_{R2} = 17.730 min (major).

^{1}H NMR (400 MHz, CDCl$_3$) δ = 7.44 – 7.42 (m, 2H), 7.36 – 7.32 (m, 2H), 7.26 – 7.21 (m, 1H), 1.85 – 1.71 (m, 3H), 1.56 (s, 3H), 1.30 – 1.22 (m, 3H), 1.15 – 1.08 (m, 1H), 0.85 (t, *J* = 7.3 Hz, 3H).

^{13}C NMR (100 MHz, CDCl$_3$) δ 148.2, 128.2, 126.6, 124.9, 74.8, 44.1, 30.3, 26.3, 23.2, 14.2.

HRMS m/z (EI): calculated for $C_{12}H_{18}O$ $[M]^+$: 178.13522, found 178.13569.

(*S*)-2-(*p*-tolyl)hexan-2-ol (2b)

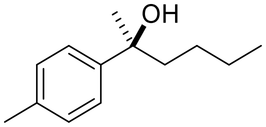

0.1 mmol scale, −70 °C for 7 days, 11.4 mg, 59%, colorless oil.

e.r. = 3.5:96.5

The enantiomeric excess was determined by chiral HPLC analysis on IJ-3 column. Conditions: isopropanol/*n*-hexane = 2.5:97.5, flow rate = 0.3 mL/min, oven temperature = 40 °C, UV-vis detection at λ = 210 nm, t_{R1} = 18.217 min (minor), t_{R2} = 22.073 min (major).

^{1}H NMR (400 MHz, CDCl$_3$) δ 7.35 – 7.29 (m, 2H), 7.19 – 7.13 (m, 2H), 2.34 (s, 3H), 1.87 – 1.73 (m, 2H), 1.69 (brs, 1H), 1.54 (s, 3H), 1.59 – 1.47 (m, 3H), 1.18 – 1.06 (m, 1H), 0.85 (t, *J* = 7.1 Hz, 3H).

^{13}C NMR (100 MHz, CDCl$_3$) δ 145.3, 136.1, 128.9, 124.8, 74.7, 44.1, 30.3, 26.3, 23.2, 21.1, 14.2.

HRMS m/z (EI): calculated for $C_{13}H_{20}O$ $[M]^+$: 192.15087, found 192.15112.

(*S*)-2-(3-methoxyphenyl)hexan-2-ol (2c)

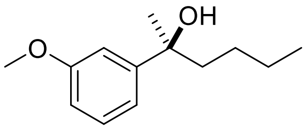

0.1 mmol scale, −40 °C for 3 days, 16.7 mg, 80%, colorless oil.

e.r. = 7.5:92.5

The enantiomeric excess was determined by chiral HPLC analysis on IJ-3 column. Conditions: isopropanol/*n*-hexane = 2.5:97.5, flow rate = 0.3 mL/min, oven temperature = 40 °C, UV-vis detection at λ = 210 nm, t_{R1} = 23.780 min (minor), t_{R2} = 29.293 min (major).

^{1}H NMR (400 MHz, $CDCl_3$) δ 7.26 (t, *J* = 7.9 Hz, 1H), 7.04 – 6.96 (m, 2H), 6.78 (ddd, *J* = 0.6, 2.5, 8.1 Hz, 1H), 3.82 (s, 3H), 1.87 – 1.75 (m, 2H), 1.74 (brs, 1H), 1.54 (s, 3H), 1.57 – 1.49 (m, 3H), 1.33 – 1.19 (m, 1H), 0.86 (d, *J* = 6.9 Hz, 3H).

^{13}C NMR (100 MHz, $CDCl_3$) δ 159.6, 150.1, 129.2, 117.4, 111.6, 111.1, 74.8, 55.3, 44.0, 30.3, 26.3, 23.2, 14.2.

HRMS m/z (EI): calculated for $C_{13}H_{20}O_2$ $[M]^+$: 208.14578, found 208.14633.

(*S*)-2-(4-chlorophenyl)hexan-2-ol (2d)

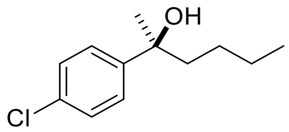

0.1 mmol scale, −40 °C for 3 days, 12.8 mg, 60%, colorless oil.

e.r. = 5:95

The enantiomeric excess was determined by chiral HPLC analysis on IJ-3 column. Conditions: isopropanol/*n*-hexane = 2.5:97.5, flow rate = 0.3 mL/min, oven temperature = 40 °C, UV-vis detection at λ = 210 nm, t_{R1} = 21.093 min (minor), t_{R2} = 22.807 min (major).

^{1}H NMR (400 MHz, $CDCl_3$) δ 7.39 – 7.32 (m, 2H), 7.32 – 7.27 (m, 2H), 1.83 – 1.72 (m, 2H), 1.69 (brs, 1H), 1.53 (s, 3H), 1.33 – 1.17 (m, 3H), 1.14 – 1.00 (m, 1H), 0.84 (t, *J* = 7.1 Hz, 3H).

^{13}C NMR (100 MHz, $CDCl_3$) δ 146.7, 132.4, 128.3, 126.5, 74.6, 44.1, 30.4, 26.2, 23.1, 14.1.

HRMS m/z (EI): calculated for $C_{12}H_{17}ClO$ $[M]^+$: 212.09624, found 212.09683.

(*S*)-2-(4-bromophenyl)hexan-2-ol (2e)

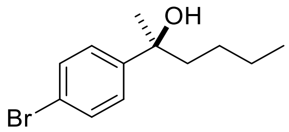

0.1 mmol scale, −40 °C for 3 days, 17.5 mg, 68%, colorless oil.

e.r. = 5:95

The enantiomeric excess was determined by chiral HPLC analysis on IJ-3 column. Conditions: isopropanol/*n*-hexane = 2.5:97.5, flow rate = 0.3 mL/min, oven temperature = 40 °C, UV-vis detection at λ = 210 nm, t_{R1} = 22.893 min (minor), t_{R2} = 24.877 min (major).

^{1}H NMR (400 MHz, $CDCl_3$) δ 7.49 – 7.41 (m, 2H), 7.34 – 7.27 (m, 2H), 1.85 – 1.71 (m, 2H), 1.68 (brs, 1H), 1.53 (s, 3H), 1.34 – 1.17 (m, 3H), 1.16 – 0.98 (m, 1H), 0.84 (t, *J* = 7.2 Hz, 3H).

^{13}C NMR (100 MHz, CDCl$_3$) δ 147.2, 131.3, 126.9, 120.5, 74.7, 44.0, 30.4, 26.2, 23.1, 14.1.
HRMS m/z (EI): calculated for $C_{12}H_{17}BrO$ $[M]^+$: 256.04573, found 256.04594.

(*S*)- 2-phenylbutan-2-ol (2f)

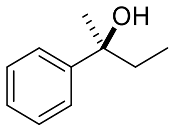

0.1 mmol scale, −60 °C for 5 days, 4.5 mg, 56% NMR yield, 30% isolated yield, colorless oil.
*Purification with 3–20% ether/pentane.
e.r. = 18.5:81.5
The enantiomeric excess was determined by chiral HPLC analysis on IJ-3 column. Conditions: isopropanol/*n*-hexane = 2.5:97.5, flow rate = 0.3 mL/min, oven temperature = 40 °C, UV-vis detection at λ = 210 nm, t_{R1} = 25.283 min (minor), t_{R2} = 27.847 min (major).
^{1}H NMR (400 MHz, CDCl$_3$) δ 7.47 – 7.41 (m, 2H), 7.37 – 7.31 (m, 2H), 7.25 – 7.21 (m, 1H), 1.92 – 1.76 (m, 2H), 1.68 (brs, 1H), 1.55 (s, 3H), 0.80 (t, *J* = 7.4 Hz, 3H).
^{13}C NMR (100 MHz, CDCl$_3$) δ 147.9, 128.2, 126.6, 125.0, 75.1, 36.8, 29.8, 8.5.
HRMS m/z (EI): calculated for $C_{10}H_{14}O$ $[M]^+$: 150.10392, found 150.10349.

(*S*)-2-phenylpentan-2-ol (2g)

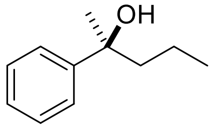

0.1 mmol scale, −60 °C for 5 days, 9.0 mg, 55%, a colorless oil.
e.r. = 6:94
The enantiomeric excess was determined by chiral HPLC analysis on IJ-3 column. Conditions: isopropanol/*n*-hexane = 6:94, flow rate = 0.3 mL/min, oven temperature = 40 °C, UV-vis detection at λ = 210 nm, t_{R1} = 16.41 min (minor), t_{R2} = 18.69 min (major).
^{1}H NMR (400 MHz, CDCl$_3$) δ = 7.45 – 7.42 (m, 2H), 7.36 – 7.32 (m, 2H), 7.26 – 7.21 (m, 1H), 1.81 – 1.76 (m, 2H), 1.63 (brs, 1H), 1.56 (s, 3H), 1.43 – 1.12 (m, 2H), 0.86 (t, *J* = 7.3 Hz, 3H).
^{13}C NMR (100 MHz, CDCl$_3$) δ 148.2, 128.2, 126.6, 124.9, 74.9, 46.7, 30.3, 17.4, 14.5.
HRMS m/z (EI): calculated for $C_{11}H_{16}O$ $[M]^+$: 164.11957, found 164.11958.

(*S*)-2-phenylnonan-2-ol (2h)

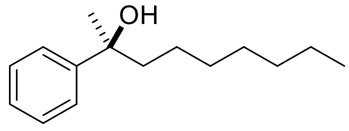

0.1 mmol scale, −60 °C for 5 days, 12.7 mg, 58%, colorless oil.

e.r. = 4:96

The enantiomeric excess was determined by chiral HPLC analysis on IJ-3 column. Conditions: isopropanol/*n*-hexane = 2.5:97.5, flow rate = 0.3 mL/min, oven temperature = 40 °C, UV-vis detection at λ = 210 nm, t_{R1} = 16.153 min (minor), t_{R2} = 18.673 min (major).

^{1}H NMR (400 MHz, $CDCl_3$) δ 7.46 – 7.39 (m, 2H), 7.37 – 7.29 (m, 2H), 7.25 – 7.19 (m, 1H), 1.86 – 1.72 (m, 2H), 1.70 (brs, 1H), 1.54 (s, 3H), 1.31 – 1.16 (m, 10H), 0.84 (t, *J* = 7.0 Hz, 3H).

^{13}C NMR (100 MHz, $CDCl_3$) δ 148.2, 128.2, 126.6, 124.9, 74.9, 44.3, 31.9, 30.3, 30.1, 29.3, 24.1, 22.8, 14.2.

HRMS m/z (EI): calculated for $C_{15}H_{24}O$ $[M]^+$: 220.18217, found 220.18269.

(*S*)-7-chloro-2-phenylheptan-2-ol (2i)

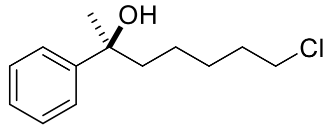

0.1 mmol scale, −60 °C for 5 days, 14.2 mg, 63%, colorless oil.

e.r. = 4:96

The enantiomeric excess was determined by chiral HPLC analysis on IJ-3 column. Conditions: isopropanol/*n*-hexane = 20:80, flow rate = 0.3 mL/min, oven temperature = 40 °C, UV-vis detection at λ = 210 nm, t_{R1} = 15.657 min (minor), t_{R2} = 21.593 min (major).

^{1}H NMR (400 MHz, $CDCl_3$) δ = 7.42 (d, *J* = 7.7 Hz, 2H), 7.34 (t, *J* = 7.4 Hz, 2H), 7.25 (t, *J* = 6.9 Hz, 1H), 3.47 (t, *J* = 6.7 Hz, 2H), 1.85 – 1.78 (m, 2H), 1.76 – 1.68 (m, 3H), 1.56 (s, 3H), 1.43 – 1.23 (m, 4H).

^{13}C NMR (100 MHz, $CDCl_3$) δ = 147.9, 128.3, 126.7, 124.9, 74.8, 45.2, 44.1, 32.6, 30.4, 27.3, 23.4.

HRMS m/z (EI): calculated for $C_{13}H_{19}ClO$ $[M]^+$: 226.11189, found 226.11234.

(*S*)-7-methoxy-2-phenylheptan-2-ol (2j)

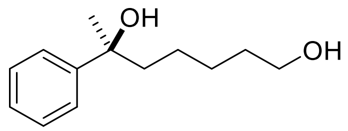

0.1 mmol scale, −60 °C for 5 days, 12.0 mg, 58%, a colorless oil.

e.r. = 96:4

The enantiomeric excess was determined by chiral HPLC analysis on IA-3 column. Conditions: isopropanol/*n*-hexane = 8:92, flow rate = 1.0 mL/min, oven temperature = 40 °C, UV-vis detection at λ = 210 nm, t_{R1} = 20.933 min (minor), t_{R2} = 15.893 min (major).

^{1}H NMR (400 MHz, $CDCl_3$) δ = 7.44 – 7.41 (m, 2H), 7.34 (t, *J* = 7.3 Hz, 2H), 7.25 – 7.19 (m, 1H), 3.58 (t, *J* = 6.6 Hz, 2H), 1.88 – 1.75 (m, 3H), 1.56 (s, 3H), 1.53 – 1.48 (m, 2H), 1.40 – 1.24 (m, 4H), 1.21 – 1.13 (m, 1H).

^{13}C NMR (100 MHz, $CDCl_3$) δ 148.1, 128.3, 126.7, 124.9, 74.8, 63.0, 44.2, 32.7, 30.4, 26.1, 23.9.

HRMS m/z (ESI): calculated for $C_{13}H_{20}O_2Na$ $[M+Na]^+$: 231.13555, found 231.13585.

(*S*)-7-methoxy-2-phenylheptan-2-ol (2k)

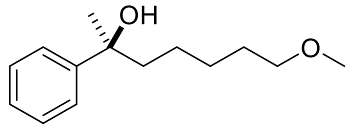

0.1 mmol scale, −60 °C for 5 days, 19.8 mg, 89%, colorless oil.

e.r. = 3:97

The enantiomeric excess was determined by chiral HPLC analysis on IJ-3 column. Conditions: isopropanol/*n*-hexane = 6:94, flow rate = 0.5 mL/min, oven temperature = 40 °C, UV-vis detection at λ = 210 nm, t_{R1} = 11.560 min (minor), t_{R2} = 15.057 min (major).

^{1}H NMR (400 MHz, $CDCl_3$) δ 7.45 – 7.39 (m, 2H), 7.37 – 7.30 (m, 2H), 7.27 – 7.20 (m, 1H), 3.31 (d, *J* = 6.6 Hz, 2H), 3.29 (s, 3H), 1.85 – 1.76 (m, 3H), 1.55 (s, 3H), 1.51 (dt, *J* = 6.5, 8.1 Hz, 2H), 1.36 – 1.25 (m, 3H), 1.20 – 1.09 (m, 1H).

^{13}C NMR (100 MHz, $CDCl_3$) δ 148.1, 128.2, 126.6, 124.9, 74.8, 72.9, 58.6, 44.2, 30.3, 29.6, 26.6, 23.9.

HRMS m/z (EI): calculated for $C_{14}H_{22}O_2$ $[M]^+$: 222.16143, found 222.16202.

(*S*)-6-methyl-2-(p-tolyl)hept-5-en-2-ol/(*S*)-(-)-gossonorol (2l)

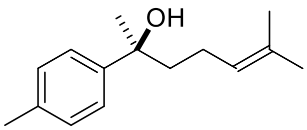

0.1 mmol scale, −70 °C for 5 days, 12.1 mg, 55%, colorless oil.

e.r. = 96.5:3.5

The enantiomeric excess was determined by chiral HPLC analysis on IJ-3 column. Conditions: isopropanol/*n*-hexane = 2.5:97.5, flow rate = 0.3 mL/min, oven temperature = 40 °C, UV-vis detection at λ = 210 nm, t_{R1} = 18.270 min (minor), t_{R2} = 24.387 min (major).

^{1}H NMR (400 MHz, $CDCl_3$) δ 7.34 – 7.29 (m, 2H), 7.19 – 7.10 (m, 2H), 5.14 – 5.05 (m, 1H), 2.34 (s, 3H), 2.05 – 1.77 (m, 5H), 1.65 (s, 3H), 1.53 (s, 3H), 1.49 (s, 3H).

^{13}C NMR (100 MHz, $CDCl_3$) δ 145.1, 136.1, 132.3, 129.0, 124.8, 124.4, 75.0, 43.8, 30.7, 25.8, 23.1, 21.1, 17.8.

HRMS m/z (EI): calculated for $C_{15}H_{22}O$ $[M]^+$: 218.16652, found 218.16703.

$[\alpha]_D^{23}$ = −10.6 (*c* = 0.83 g/L, $CHCl_3$); Lit. (for (*S*)-Gossonorol) **$[\alpha]_D^{20}$** = −11.9 (*c* = 1.0 g/L, $CHCl_3$)

Analytical data were in accordance with literature reported results and the absolute configuration is determined based on them.[15]

2-(cyclohex-1-en-1-yl)octan-2-ol (2m)

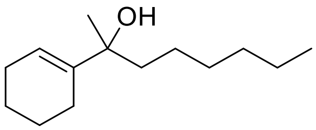

9.0 μmol scale, −80 °C for 18 hours, quenched with TEA solution (in *n*-BuCl, 0.14 M, 100 μL), solvent ratio = *n*-BuCl:PFTB = 1:1, 11% NMR yield, 93:7 e.r. (NMR yield was determined with 2.00 μL of mesitylene (14.4 μmol) as an internal standard)

GC condition: Injector temperature: 250 °C, Temperature: 100 °C for 20 min, 3 °C/min gradient increment to 130 °C, carrier gas N2, column flow 1.56 mL/min, t_{minor} = 95.75 min, t_{major} = 98.65 min.

^{1}H NMR (400 MHz, $CDCl_3$) δ = 5.62 - 5.76 (m, 1H), 1.99 - 2.08 (m, 2H), 1.88 - 1.98 (m, 2H), 1.46 - 1.69 (m, 14H), 1.11 - 1.40 (m, apparent 29H, overlap with hexane), 0.77 - 0.93 (m, apparent 9H, overlap with hexane).

^{13}C NMR (100 MHz, CDCl$_3$)δ = 142.48, 119.73, 75.28, 40.54, 31.93, 29.80, 27.63, 25.20, 24.84, 23.98, 23.19, 22.74, 22.46, 14.18.

HRMS m/z (EI): calculated for $C_{14}H_{26}O$ $[M]^+$: 210.19782; found 210.19811.

2-cyclohexylhexan-2-ol (2n)

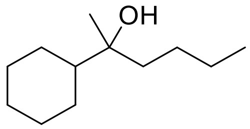

9.0 µmol scale, −40 °C for 18 hours, solvent ratio = *n*-BuCl:PFTB = 1:9, 17% NMR yield, 61:39 e.r. (NMR yield was determined with 2.00 µL of mesitylene (14.4 µmol) as an internal standard)

GC condition: Injector temperature: 250 °C, Temperature: 100 °C for 20 min, 3 °C/min gradient increment to 120 °C, carrier gas N2, column flow 1.56 mL/min, t_{major} = 56.78 min, t_{minor} = 58.29 min.

^{1}H NMR (400 MHz, CDCl$_3$) δ 1.85 - 1.57 (m, 5H), 1.48 - 1.36 (m, 2H), 1.34 - 1.08 (m, 8H), 1.08 - 0.93 (m, 5H), 0.92 - 0.78 (m, 3H).

^{13}C NMR (100 MHz, CDCl$_3$) δ 74.54, 47.26, 39.70, 27.59, 26.93, 26.92, 26.87, 26.65, 25.59, 24.13, 23.45, 14.27.

HRMS m/z (EI): calculated for $C_{11}H_{21}O[M-CH_3]^+$: 169.15869 found 169.15895.

(2-methoxyhexan-2-yl)benzene

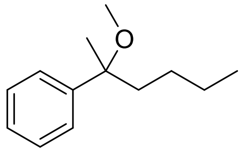

9.0 µmol scale, −60 °C for 18 h, 16% NMR yield, 12% yield, colorless oil.

e.r. = 56:44

The enantiomeric excess was determined by chiral UHPLC analysis on IB-U column. Conditions: isopropanol/water = 50:50, flow rate = 0.1 mL/min, oven temperature = 35 °C, UV-vis detection at λ = 210 nm, t_{R1} = 24.907 min (minor), t_{R2} = 27.523 min (major).

^{1}H NMR (400 MHz, CDCl$_3$) δ = 7.30 – 7.40 (m, 4H), 7.22 – 7.26 (m, 1H), 3.07 (s, 3H), 1.69 – 1.80 (m, 2H), 1.52 (s, 3H), 1.08 – 1.35 (m, 4H), 0.84 (t, J = 7.3 Hz, 3H).

^{13}C NMR (100 MHz, $CDCl_3$) δ = 145.5, 128.2, 126.8, 126.3, 79.2, 50.4, 42.7, 26.2, 23.3, 23.1, 14.2.

Scale-up synthesis of 2a

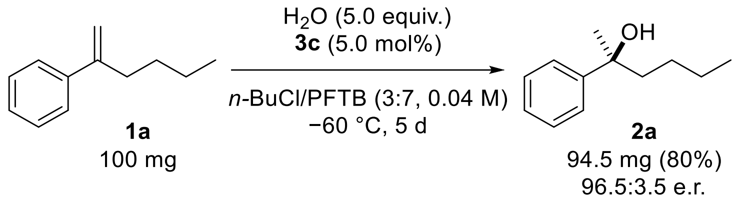

To IDPi (84.5 mg, 31.2 µmol, 0.05 equiv.) in a 100 mL Schlenk flask were added 1-chlorobutane (1.54 mL), nonafluoro-*tert*-butyl alcohol (10.8 mL) and water (56.2 µL, 5.0 equiv.) at room temperature. The mixture was cooled to -60 °C and stirred for 10 min. Next, alkene solution (3.10 mL, 0.624 mmol, 0.2 M in 1-chlorobutane, 1.0 equiv.) was added dropwise to the reaction mixture. After stirring for 5 days, the mixture was quenched with triethylamine (300 µL) and stirred at the same temperature for 10 min. Then the mixture was allowed to warm to room temperature, after which dibromomethane (42.0 µL, 0.60 mmol) was added. An aliquot (100 µL) was taken and dissolved in $CDCl_3$ (500 µL) for conversion determination by ^{1}H NMR. Solvents were removed under reduced pressure. Purification by column chromatography with silica gel (Rening 10 g) using 0-15% Et_2O/hexane as an eluent afforded the product as a colorless oil (94.5 mg). Subsequently, NMR was measured with mesitylene (80 µL, 576 µmol) as an internal standard. As a small amount of Et_2O (7.07 µmol, 5.24 mg) remained, the net yield was 89.2 mg (500.5 µmol, 80%). The enantiomeric ratio was 96.5:3.5.

Temperature-triggered deracemization of tertiary alcohols

General procedure for formal deracemization

To IDPi (1.80 mg, 0.67 µmol, 0.05 equiv.) in a vial under nitrogen were added substrate alcohol solution (100 µL, 13.3 µmol, 0.133 M in dry 1-chlorobutane, 1.0 equiv.). The mixture was warmed to 30 °C and stirred for 18 hours. Subsequently, the mixture was cooled to –60 °C, and then a solution of water in PFTB (235 µL, 5.0 equiv of H_2O, 0.283 M) was added to give a final concentration of 0.04 M. After stirring for 3 days, the reaction was quenched with triethylamine (2.0 µL) and stirred at the same temperature for 10 min. The solvents were removed under reduced pressure. The residue was purified on SiO_2 using 0-15% Et_2O/hexane, and the volatiles were removed *in vacuo*. $CDCl_3$ and mesitylene (2.0 µL, 14.4 µmol) as an internal standard were added, and the chemical yield was calculated from ^{1}H NMR. The volatiles were then removed, and the residue was dissolved in hexane for HPLC analysis to determine the enantioselectivity.

The analytical data for all compounds were consistent with those of the hydration products.

Catalyst preparation

The catalyst **3a** and **3b** was prepared according to the reported procedure.[16, 17]

(*S*,*S*)-3,3'- di(spiro[cyclopentane-1,9'-fluoren]-2'-yl)-$C_{10}F_{21}$-IDPi (3c)

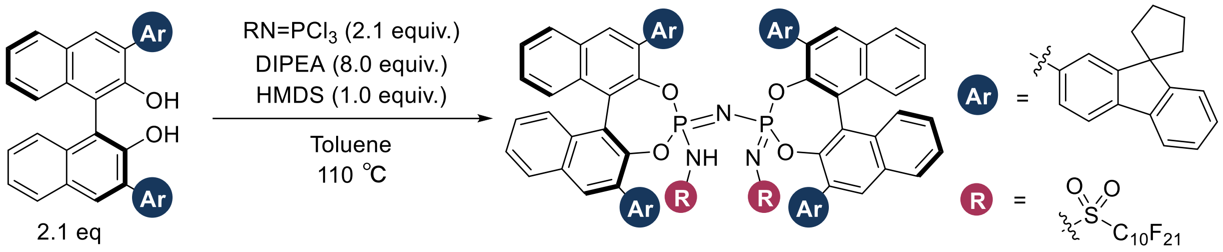

In a flame-dried 35 mL J-Young tube, (*S*)-3,3'-di(spiro[cyclopentane-1,9'-fluoren]-2'-yl)-[1,1'-binaphthalene]-2,2'-diol (0.59 g, 0.82 mmol, 2.1 equiv.) (ref: *J. Am. Chem. Soc.* **2018**, *140*, 12671−12676), ((perfluorodecyl)sulfonyl)phosphorimidoyl trichloride (0.60 g, 0.82 mmol, 2.1 equiv.), and toluene (4.0 mL) were added. *N*-ethyldiisopropylamine (0.54 mL, 3.1 mmol, 8.0 equiv.) was added and the mixture was stirred at room temperature for 40 minutes, showing white precipitate in the reaction. Next, the mixture was stirred at 60 °C for another 20 minutes. Finally, hexamethyldisilazane (82.0 µL, 1.0 mmol, 1.0 equiv.) was added, and then the mixture was heated

to 110 °C for 3 days. The reaction mixture was then cooled to room temperature, and all the volatiles were removed under reduced pressure. The mixture was dissolved in $CHCl_3$, washed with 1 M aqueous HCl solution. The two phases were separated, and the aqueous layer was washed twice with $CHCl_3$ (15 mL each). The combined organic layer was dried over Na_2SO_4. The resulting mixture was filtrated, dried *in vacuo*, and purified by column chromatography on SiO_2 to give the desired product using 0-8% ethyl acetate/hexane and 10% $CHCl_3$ as a constant. The material was subsequently acidified in $CHCl_3$ with 6 M aqueous HCl solution. The organic layer was collected and concentrated under reduced pressure to furnish the (*S,S*)-3,3'- di(spiro[cyclopentane-1,9'-fluoren]-2'-yl)-$C_{10}F_{21}$-*IDPi* **(3c)** as white solid (0.37 g, 35%).

^{1}H NMR (400 MHz, $CDCl_3$) δ = 8.04 (d, *J* = 5.5 Hz, 4H), 7.97 (d, *J* = 8.2 Hz, 2H), 7.83 (t, *J* = 7.6 Hz, 2H), 7.78 (d, *J* = 8.5 Hz, 2H), 7.66 (t, *J* = 7.6 Hz, 2H), 7.58 – 7.50 (m, 6H), 7.41 (t, *J* = 8.4 Hz, 4H), 7.35 – 7.14 (m, 18H), 6.98 (s, 2H), 6.63 (d, *J* = 7.8 Hz, 2H), 6.56 (d, *J* = 8.0 Hz, 2H), 6.45 (d, *J* = 8.0 Hz, 2H), 2.16 – 1.97 (m, 24H), 1.81 – 1.59 (m, 8H).

^{13}C NMR (100 MHz, $CDCl_3$) δ 155.0, 154.8, 154.4, 153.9, 144.3 (t, *J* = 4.8 Hz), 143.1 (t, *J* = 4.8 Hz), 140.0, 139.3, 139.2, 138.7, 135.0, 135.0, 134.3, 134.0, 132.3, 132.2, 131.8, 131.8, 131.7, 131.3, 129.5, 128.9, 128.6, 127.9, 127.3, 127.2, 127.1, 127.0, 126.8, 126.6, 126.5, 126.3, 124.0, 123.6, 123.5, 122.9, 122.6, 121.9, 119.9, 119.3, 118.7, 118.5, 113.5, 110.8, 110.3, 58.1, 57.9, 39.7, 39.3, 39.0, 27.1, 26.7, 26.6, 26.5

^{31}P NMR (162 MHz, $CDCl_3$) δ −15.93.

^{19}F NMR (376 MHz, $CDCl_3$) δ −80.6 (t, *J* = 9.7 Hz, 6F), −111.4 (dd, *J* = 394.0, 257.7 Hz, 4F), −119.9 (s, 4F), −121.3– −121.7 (m, 20F), −122.6 (s, 4F), −126.0 (s, 4F).

HRMS m/z (ESI): calculated for $C_{128}H_{80}O_8N_3F_{42}P_2S_2$ [M-H]$^-$: 2710.41969, found 2710.41654.

NMR spectra

1-chloro-4-(hex-1-en-2-yl)benzene (1d)

^{1}H NMR

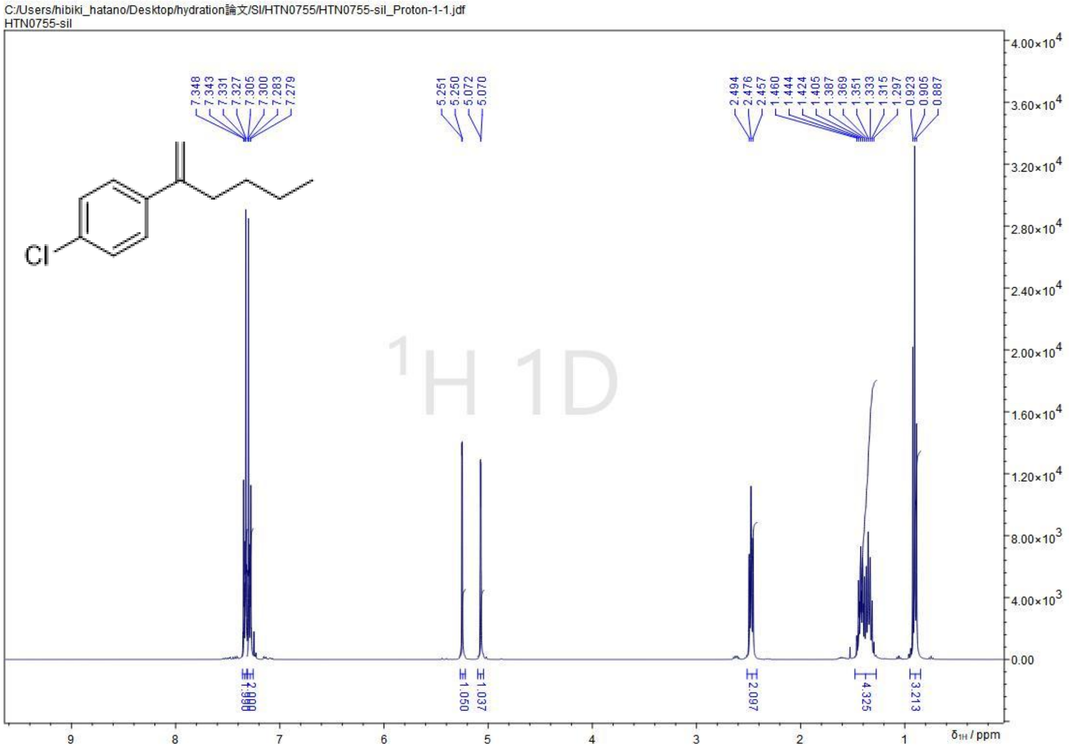

^{13}C NMR

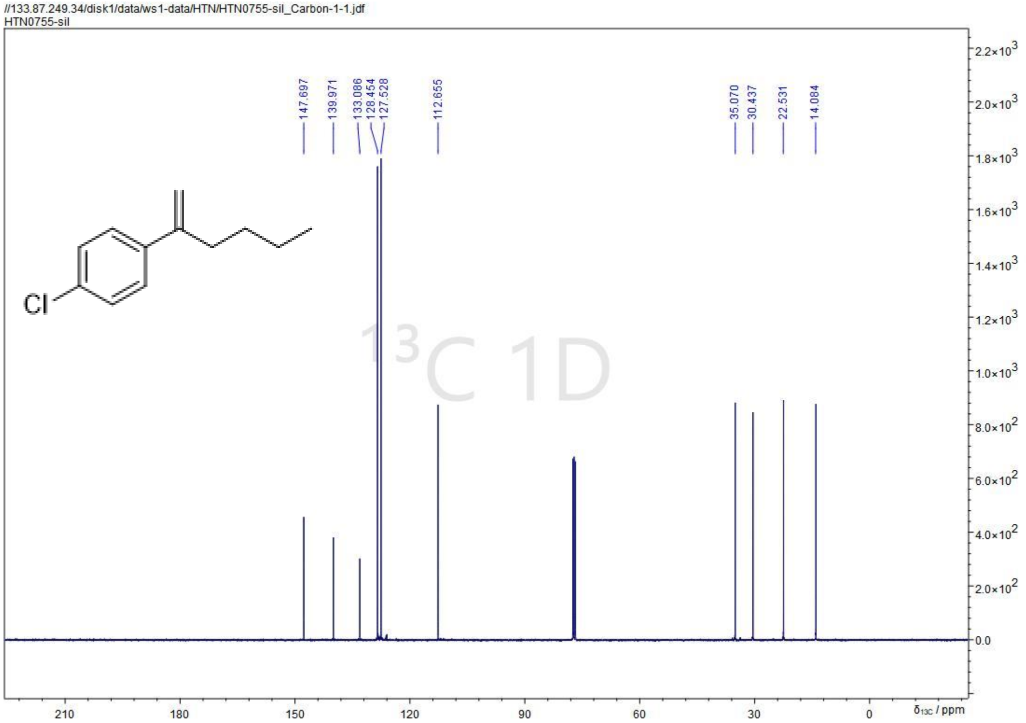

(7-chlorohept-1-en-2-yl)benzene (1i)

^{1}H NMR

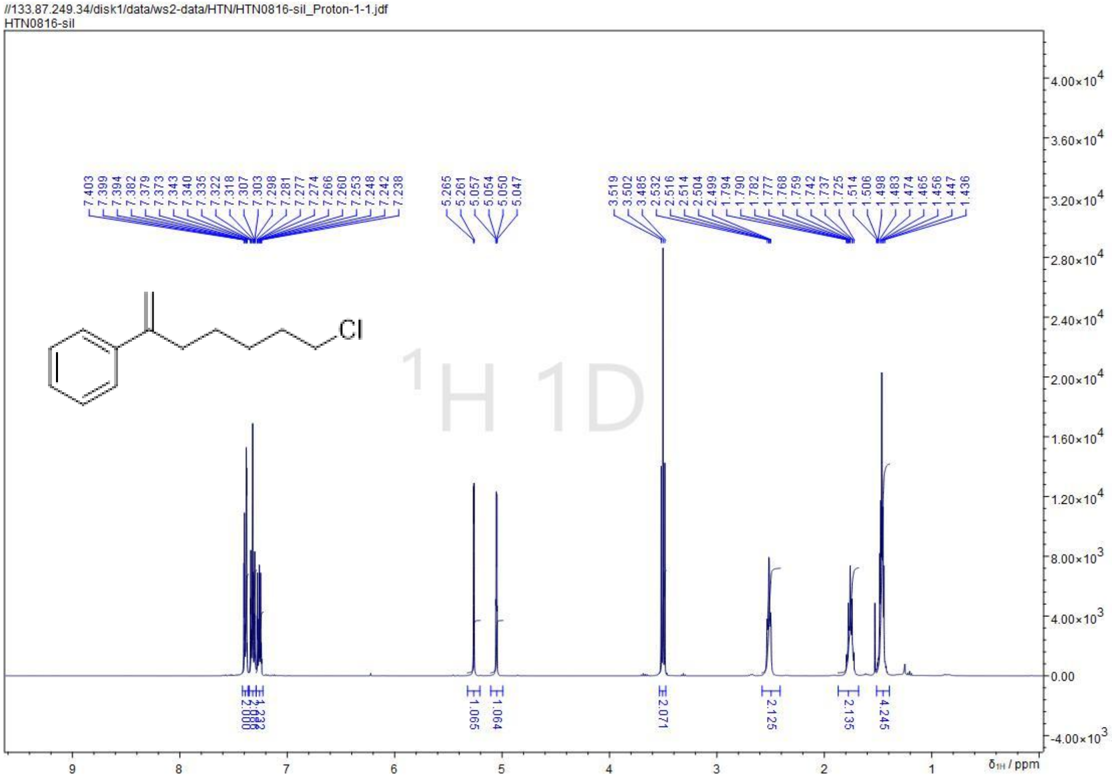

^{13}C NMR

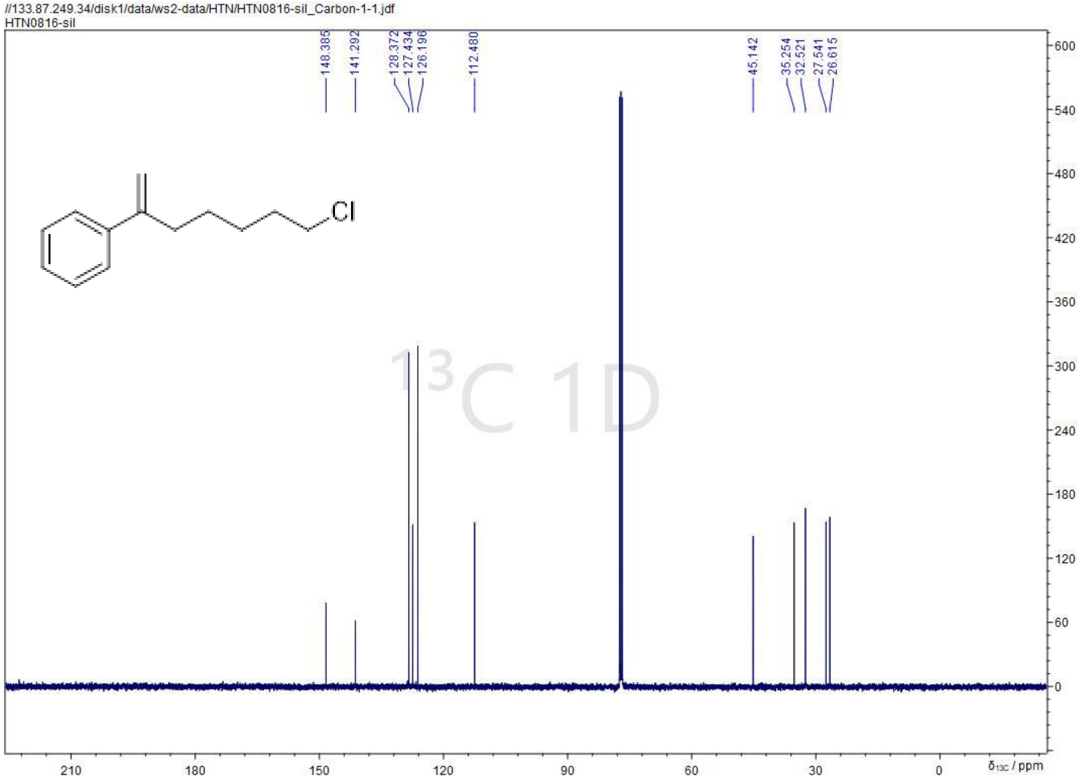

6-phenylhept-6-en-1-ol (1j)

^{1}H NMR

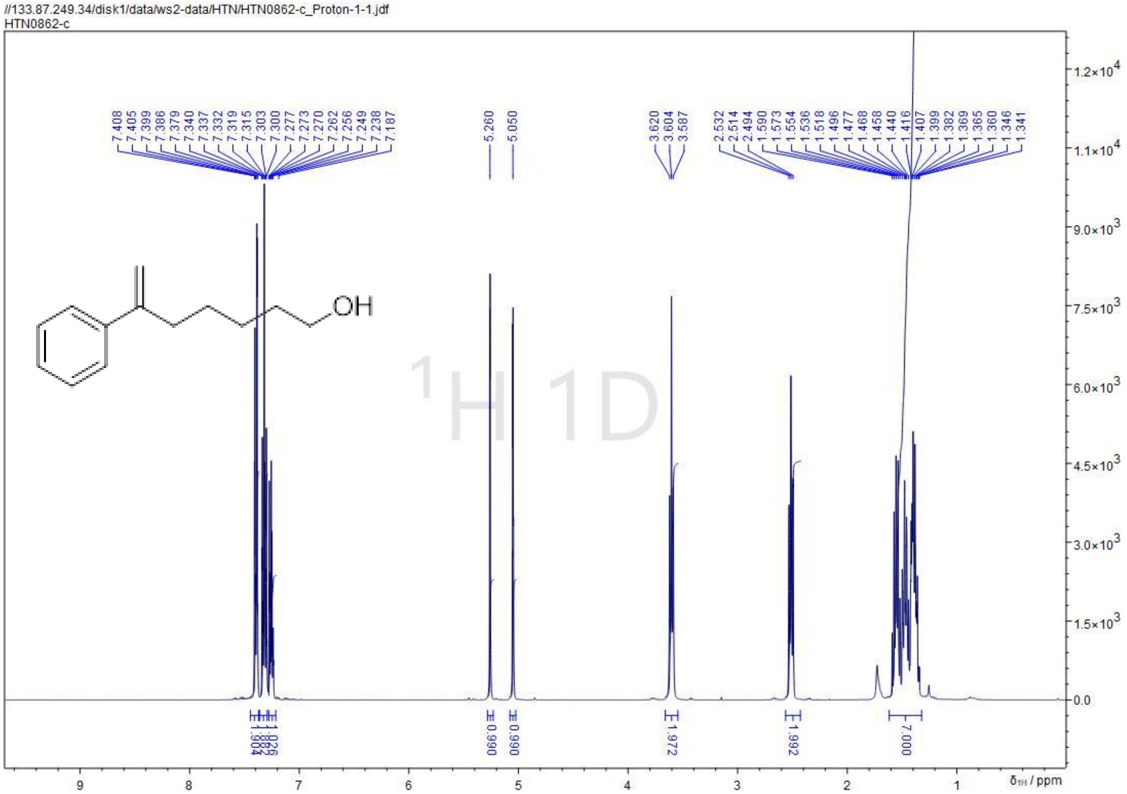

^{13}C NMR

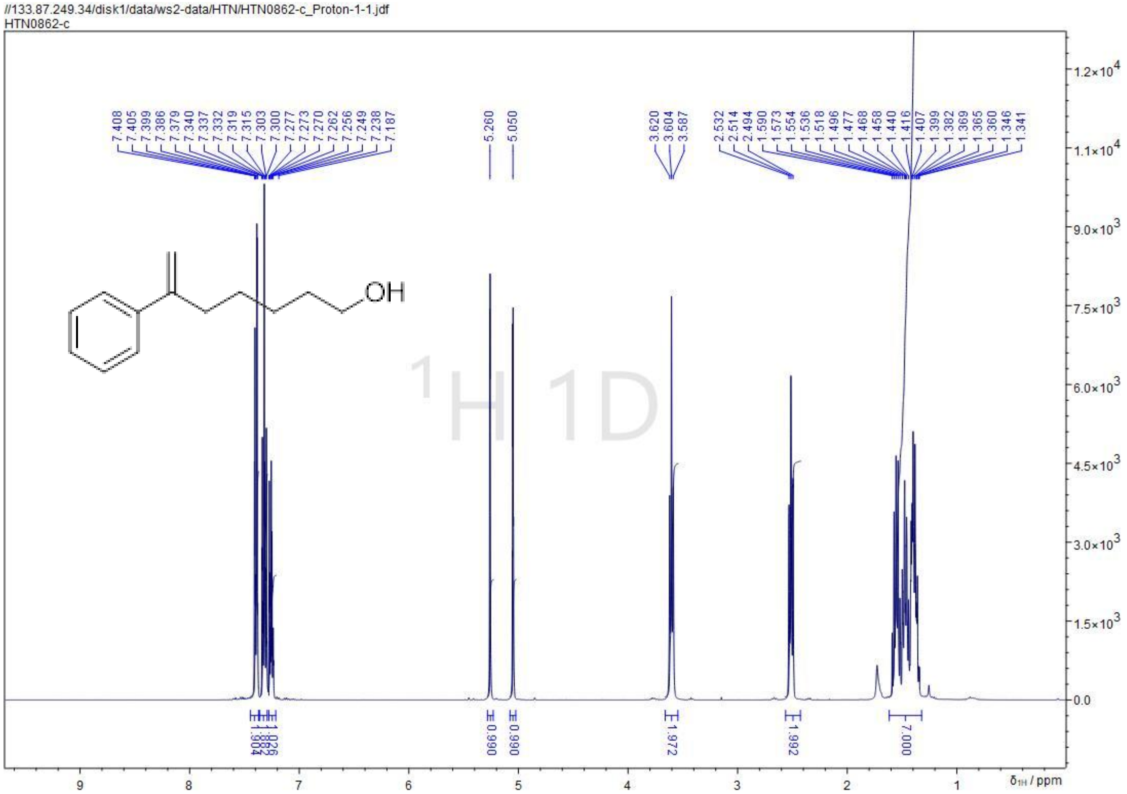

(7-methoxyhept-1-en-2-yl)benzene (1k)

^{1}H NMR

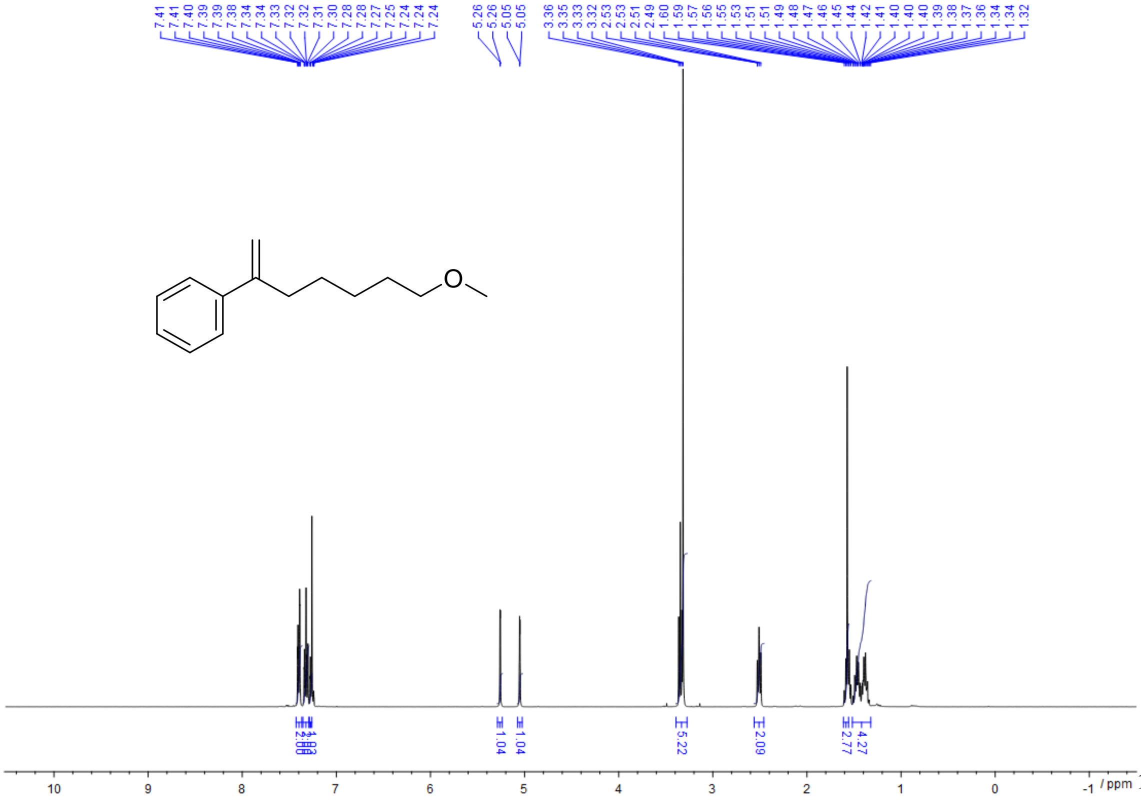

^{13}C NMR

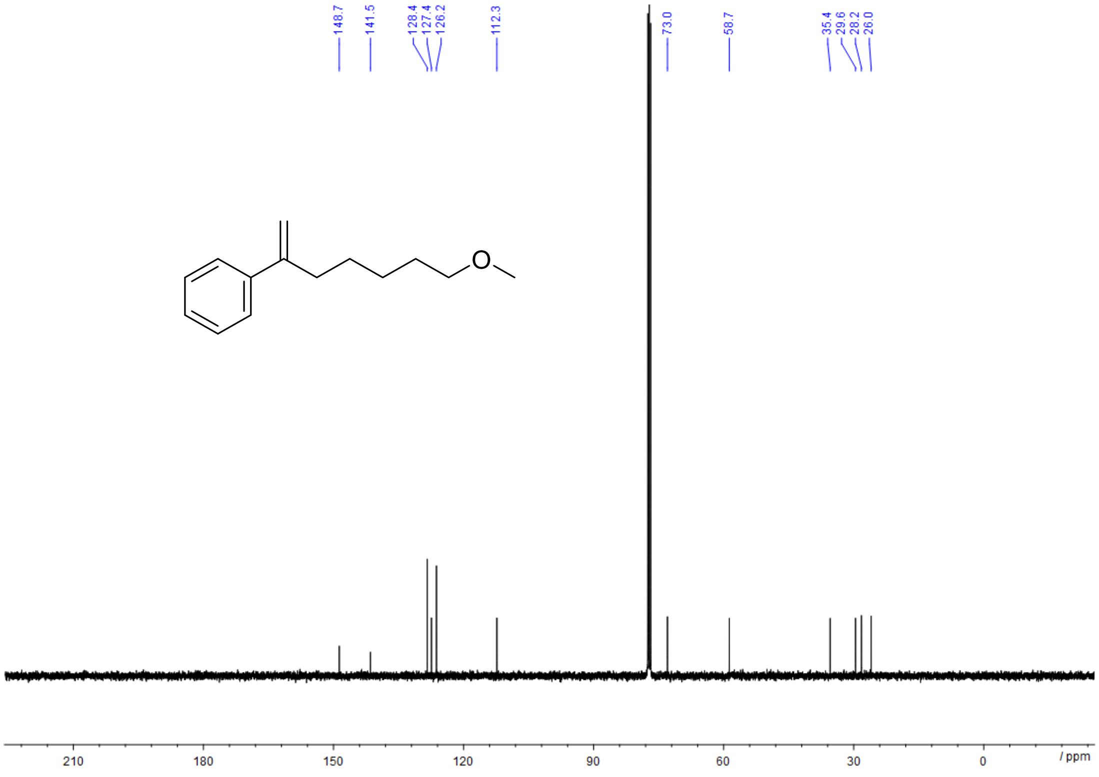

(*S*)-2-phenylhexan-2-ol (2a)

^{1}H NMR

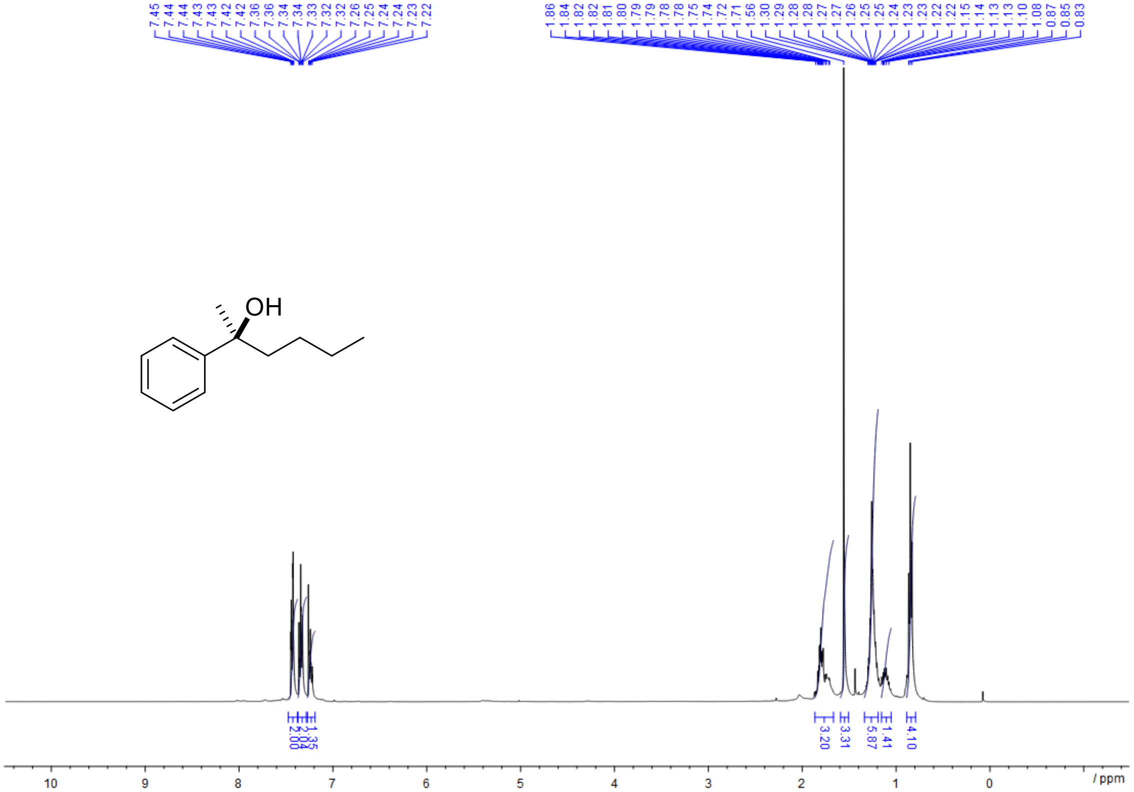

^{13}C NMR

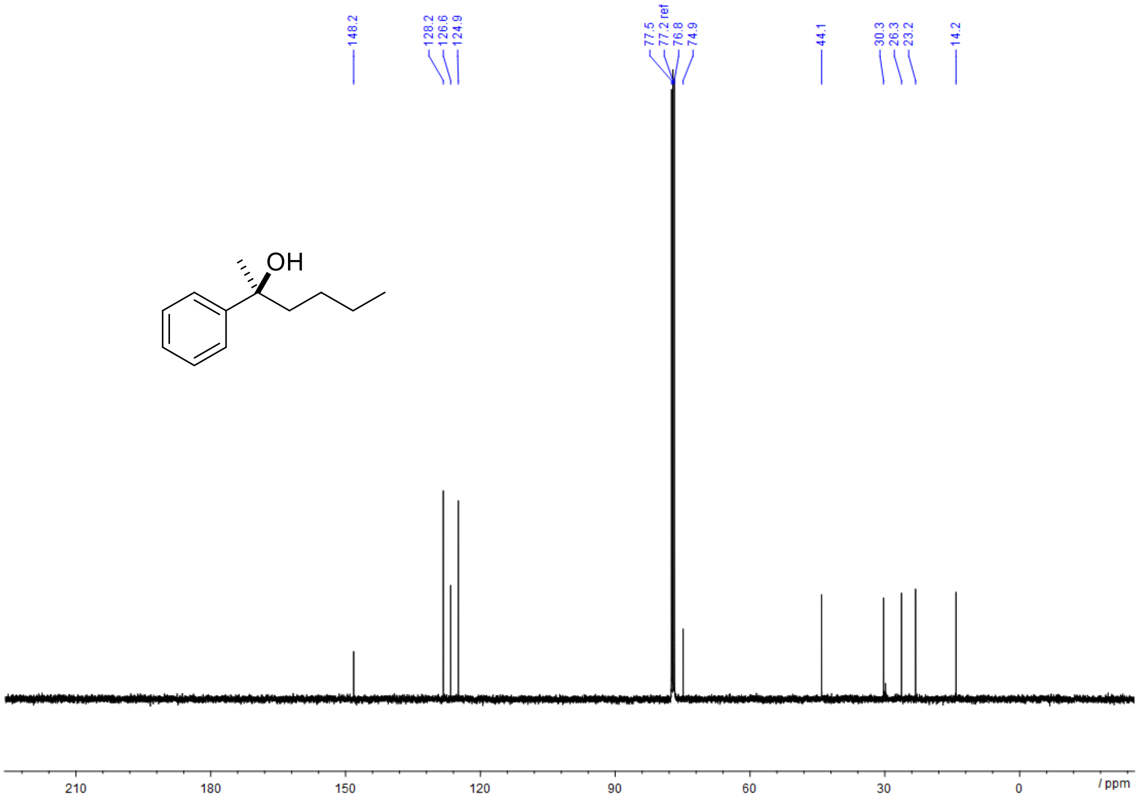

(S)-2-(p-tolyl)hexan-2-ol (2b)

^{1}H NMR

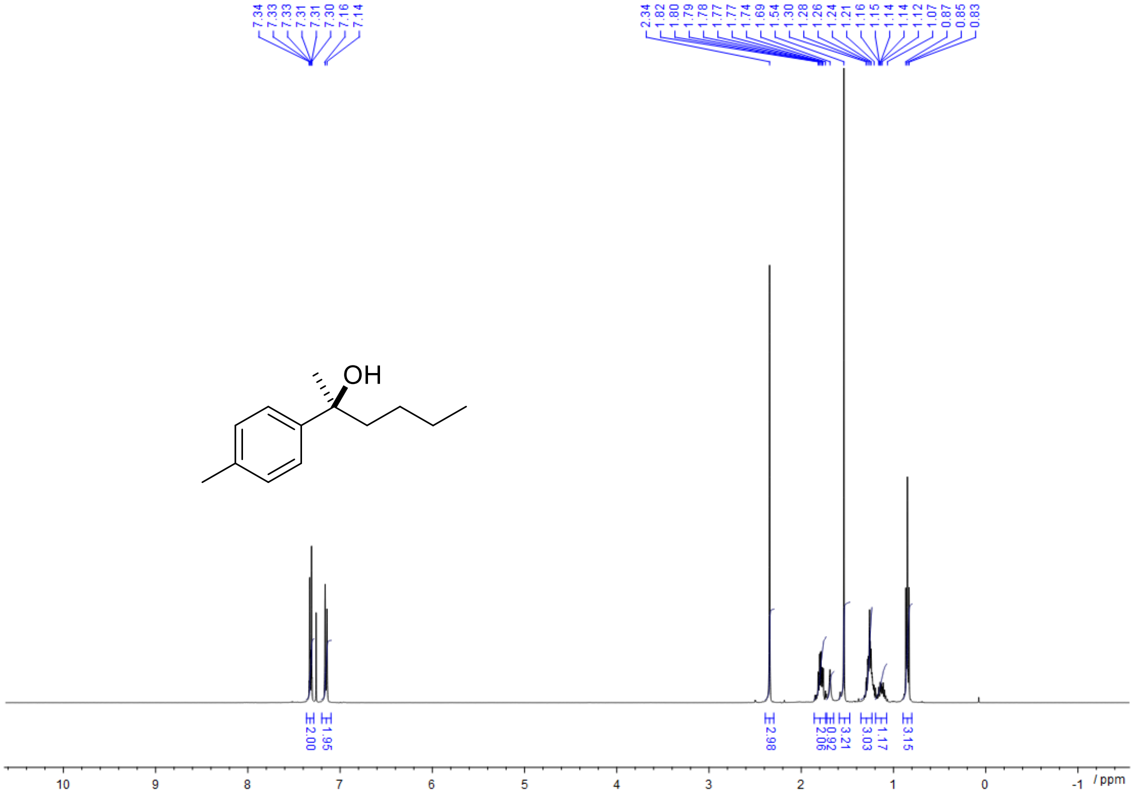

^{13}C NMR

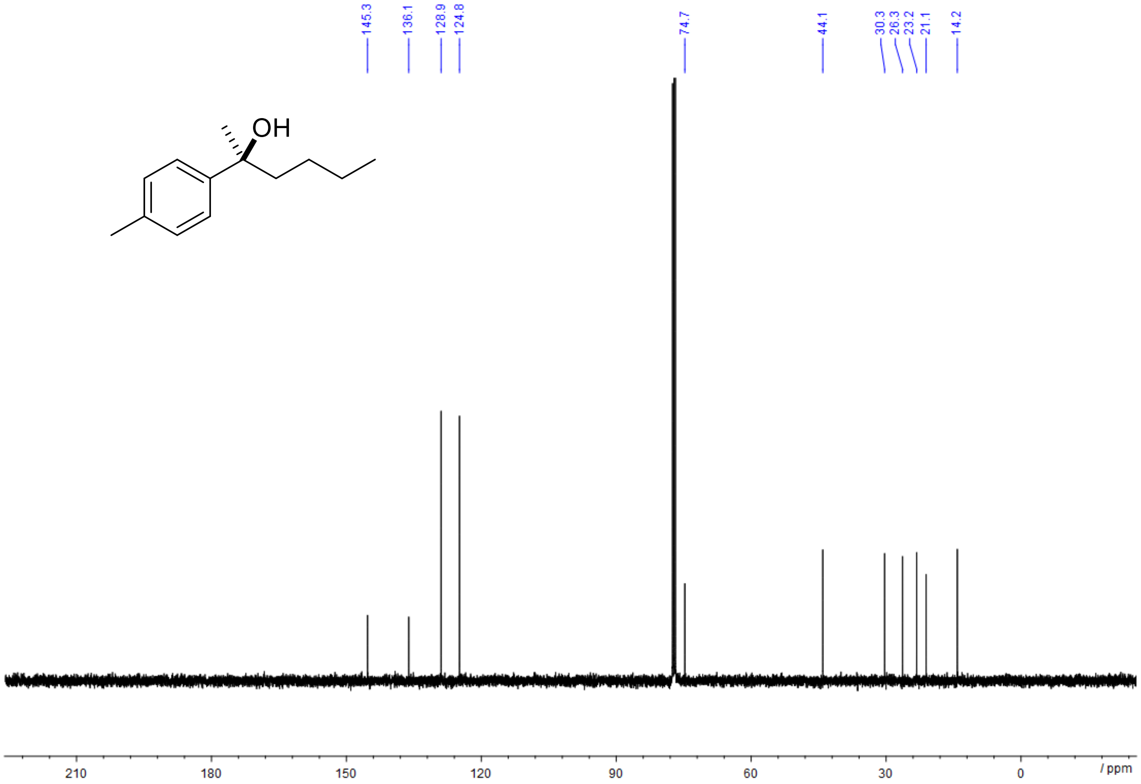

(*S*)-2-(3-methoxyphenyl)hexan-2-ol (2c)

^{1}H NMR

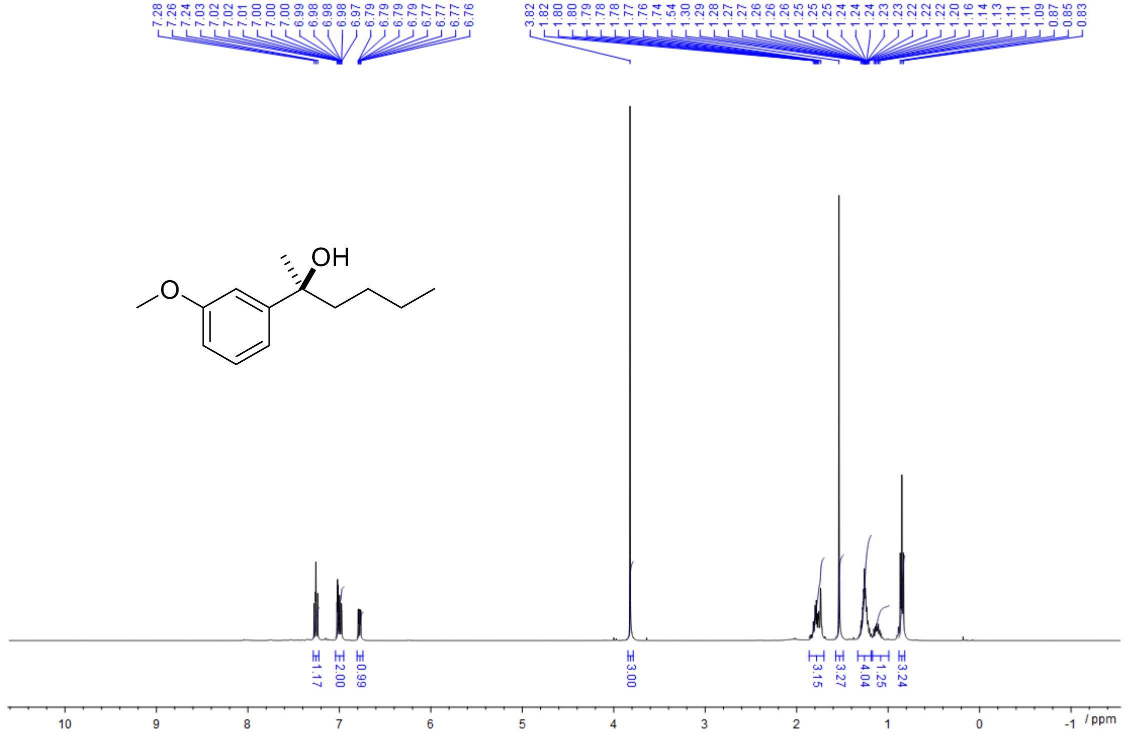

^{13}C NMR

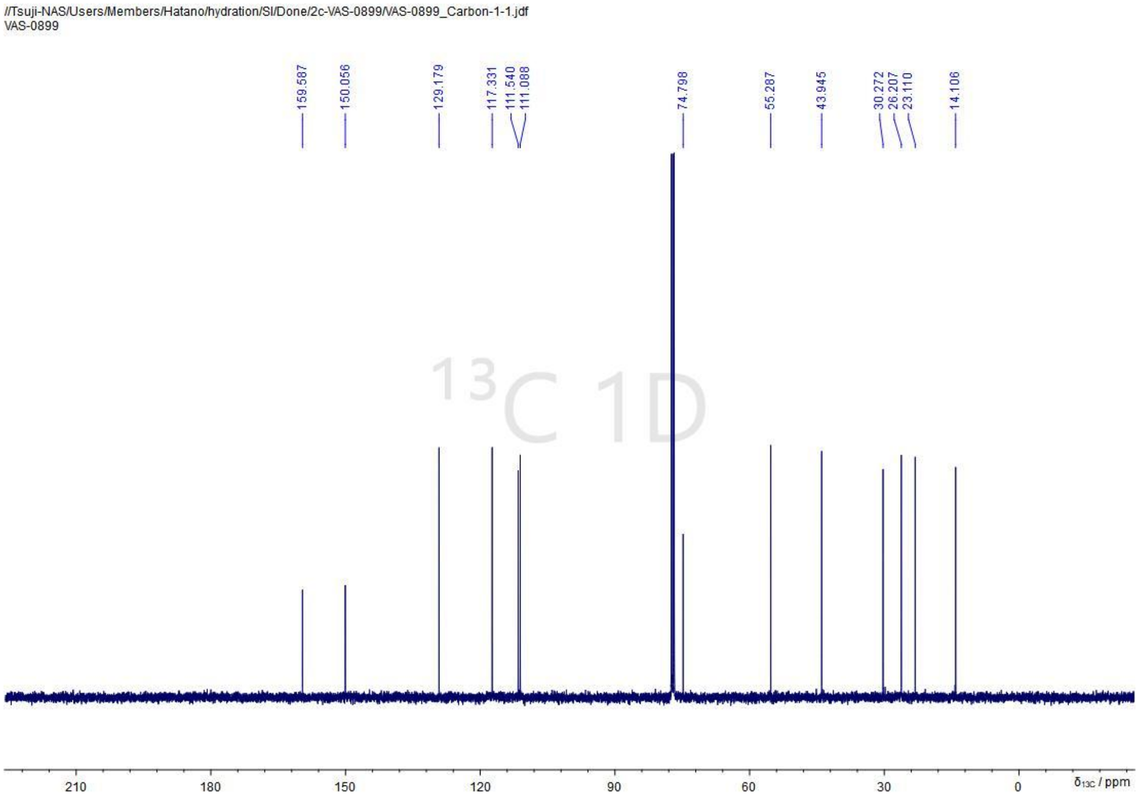

(*S*)-2-(4-bromophenyl)hexan-2-ol (2d)

^{1}H NMR

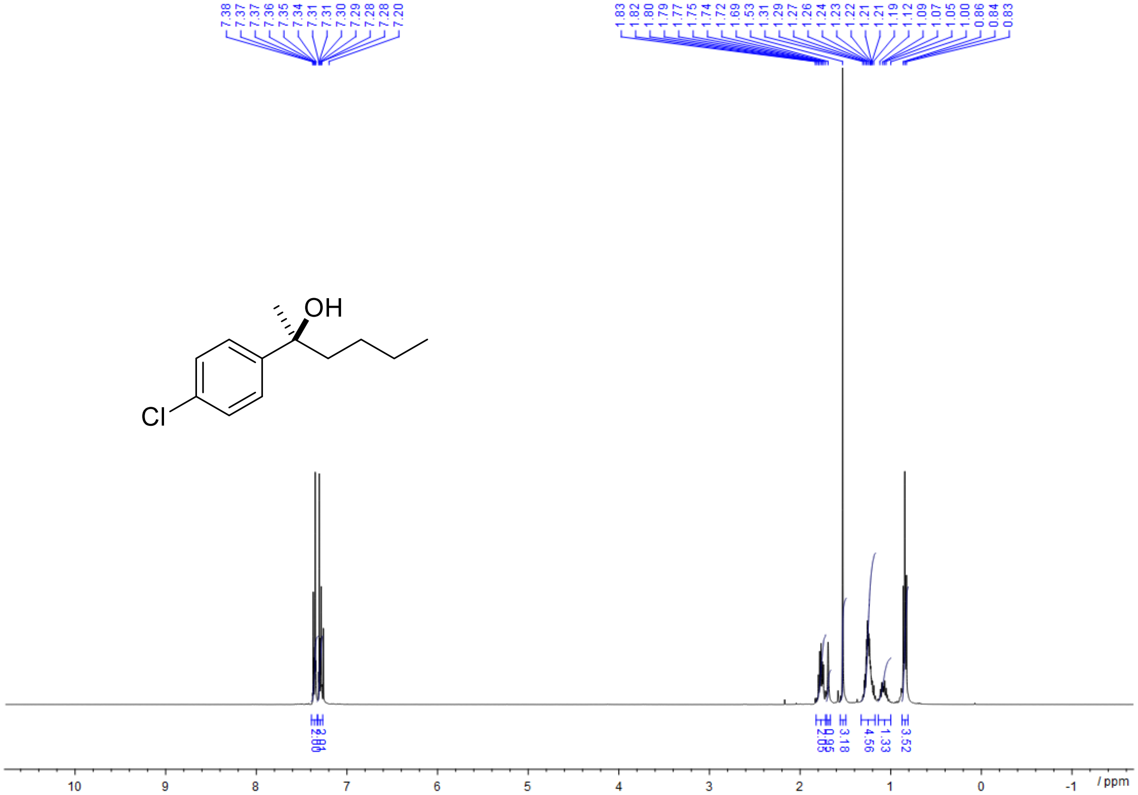

^{13}C NMR

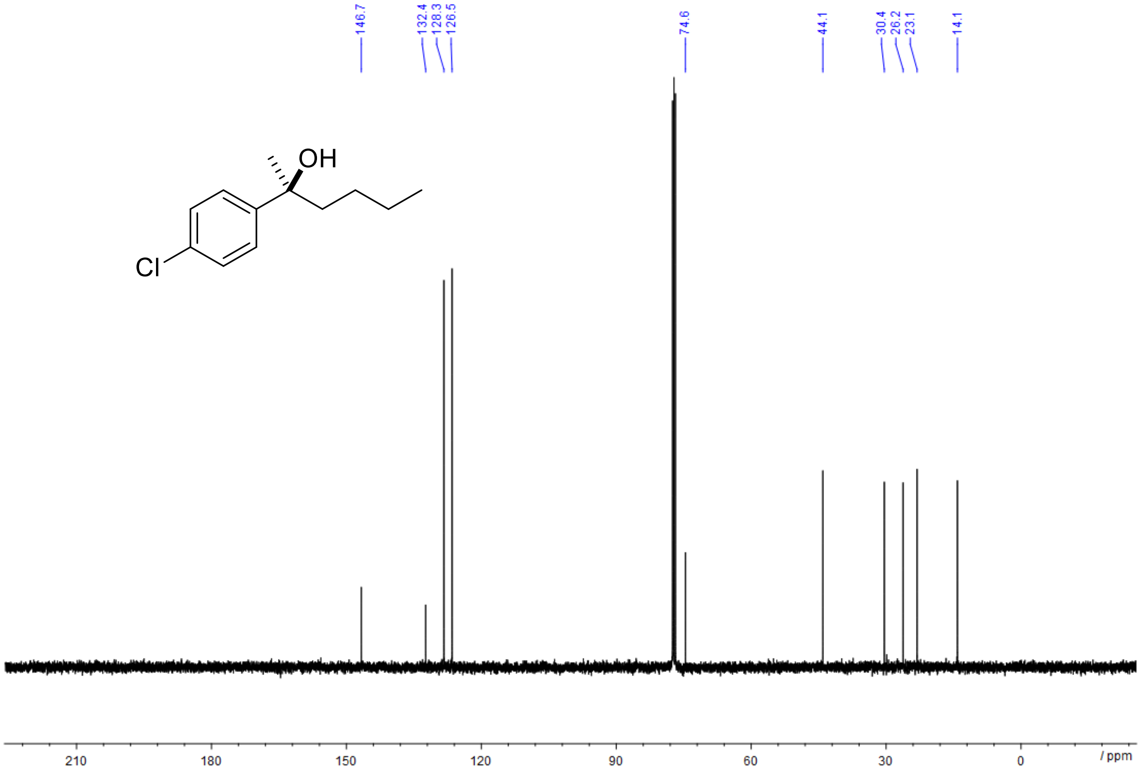

(*S*)-2-(4-bromophenyl)hexan-2-ol (2e)

^{1}H NMR

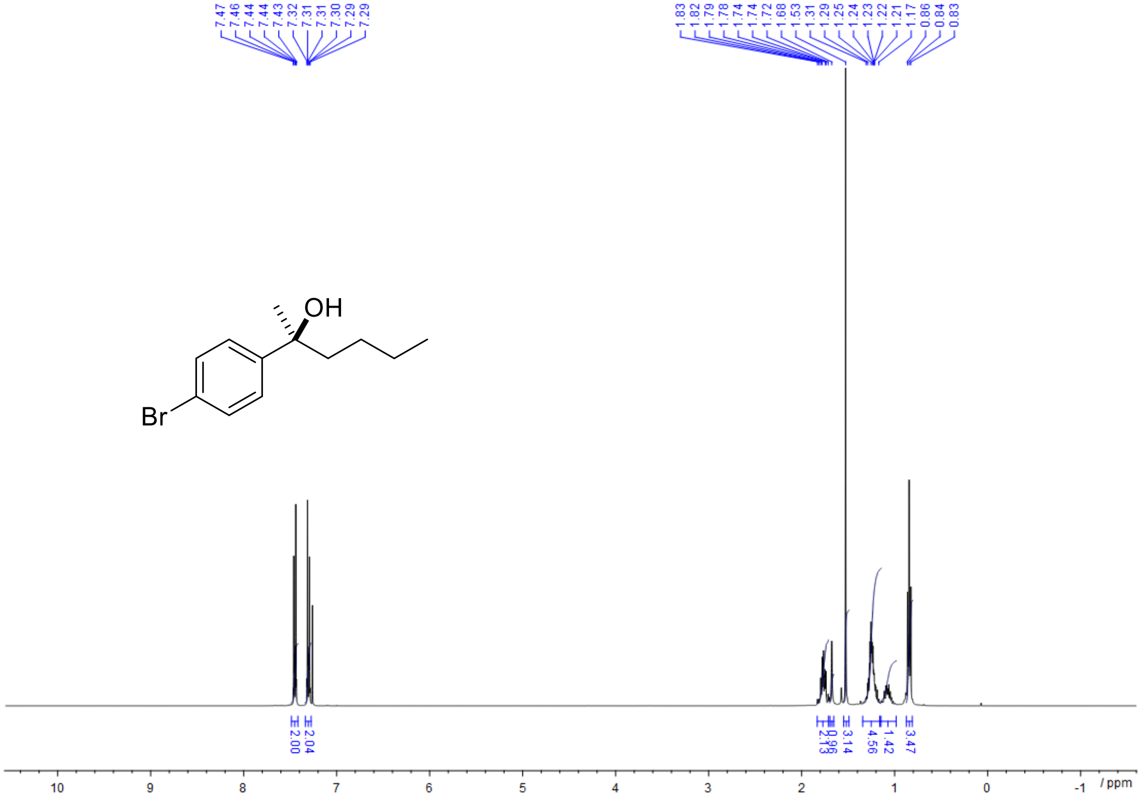

^{13}C NMR

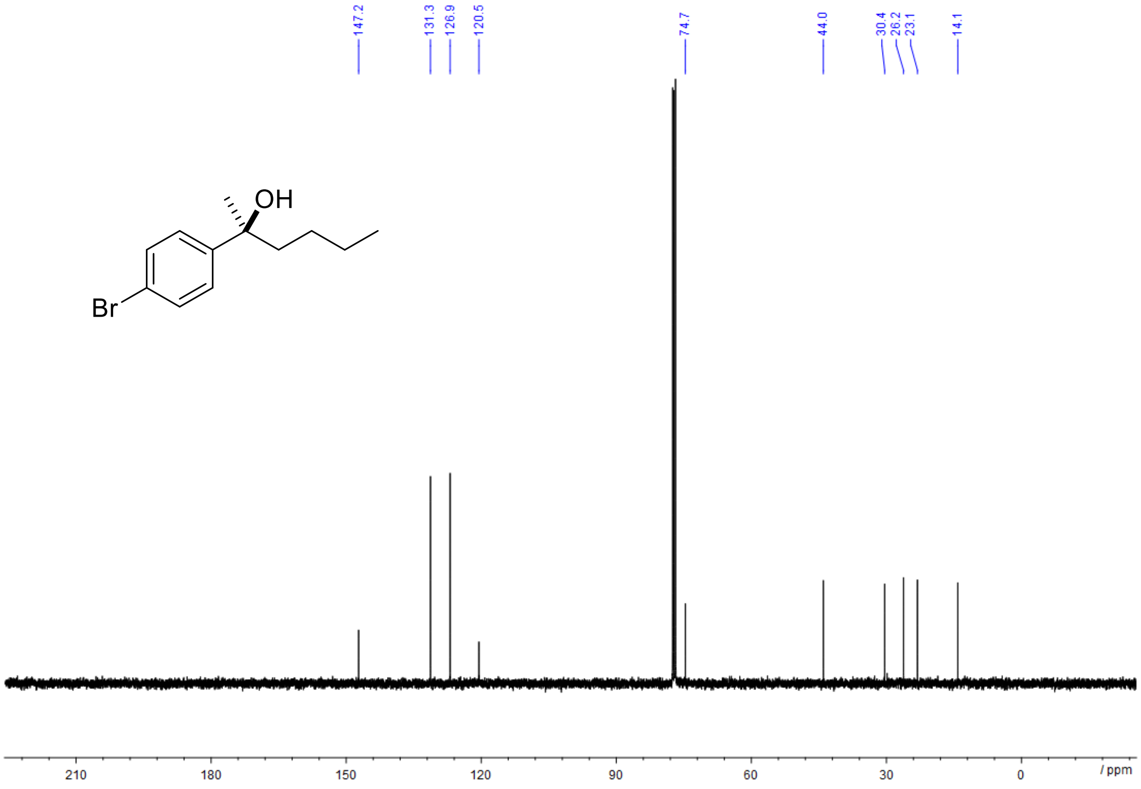

(*S*)- 2-phenylbutan-2-ol (2f)

^{1}H NMR

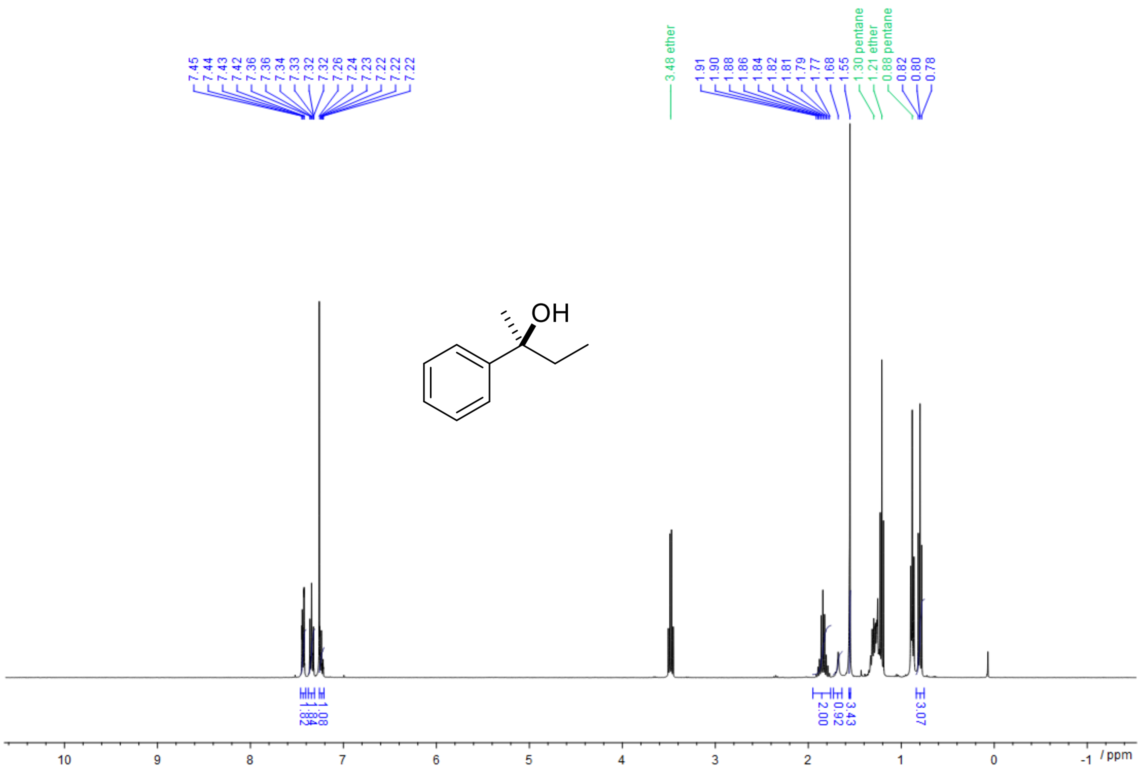

^{13}C NMR

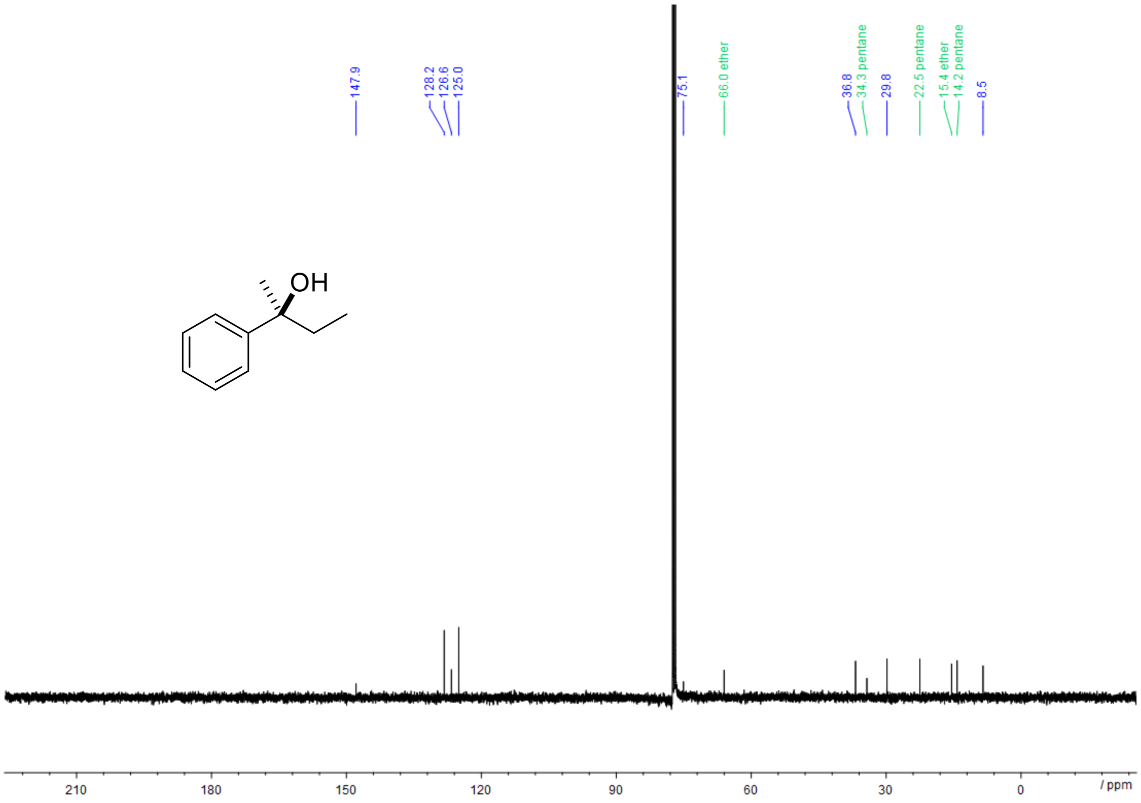

(*S*)-2-phenylpentan-2-ol (2g)

^{1}H NMR

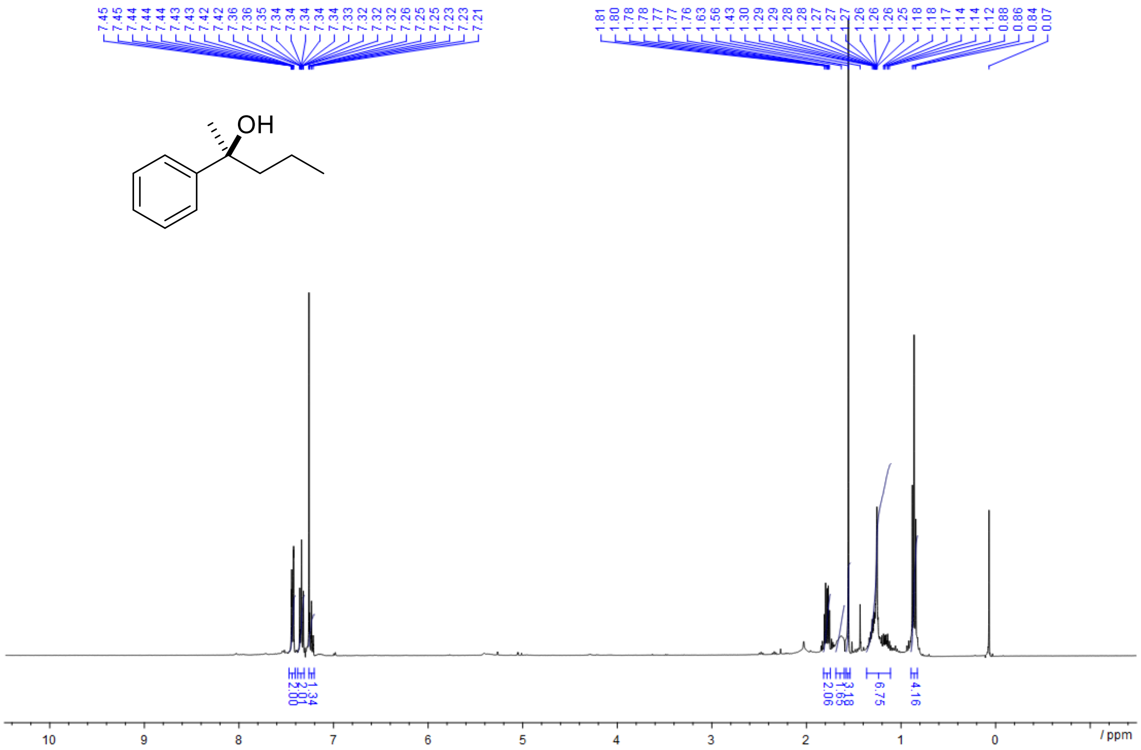

^{13}C NMR

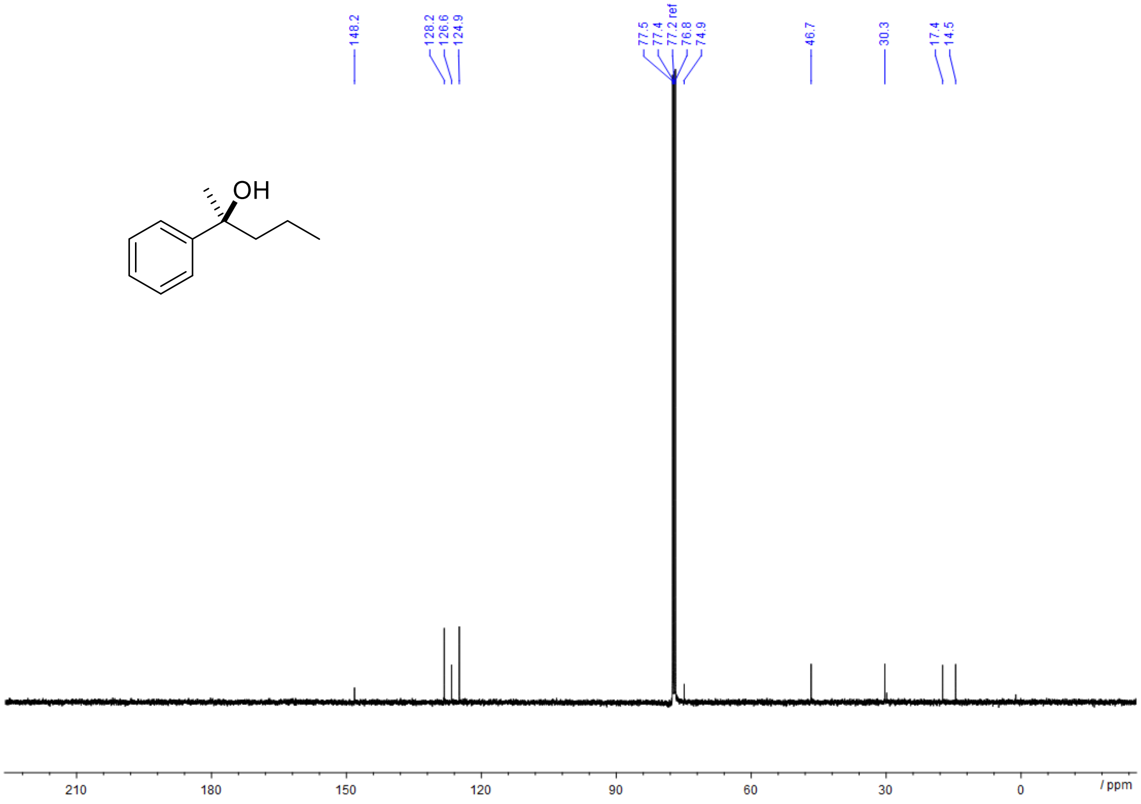

(*S*)-2-phenylnonan-2-ol (2h)

^{1}H NMR

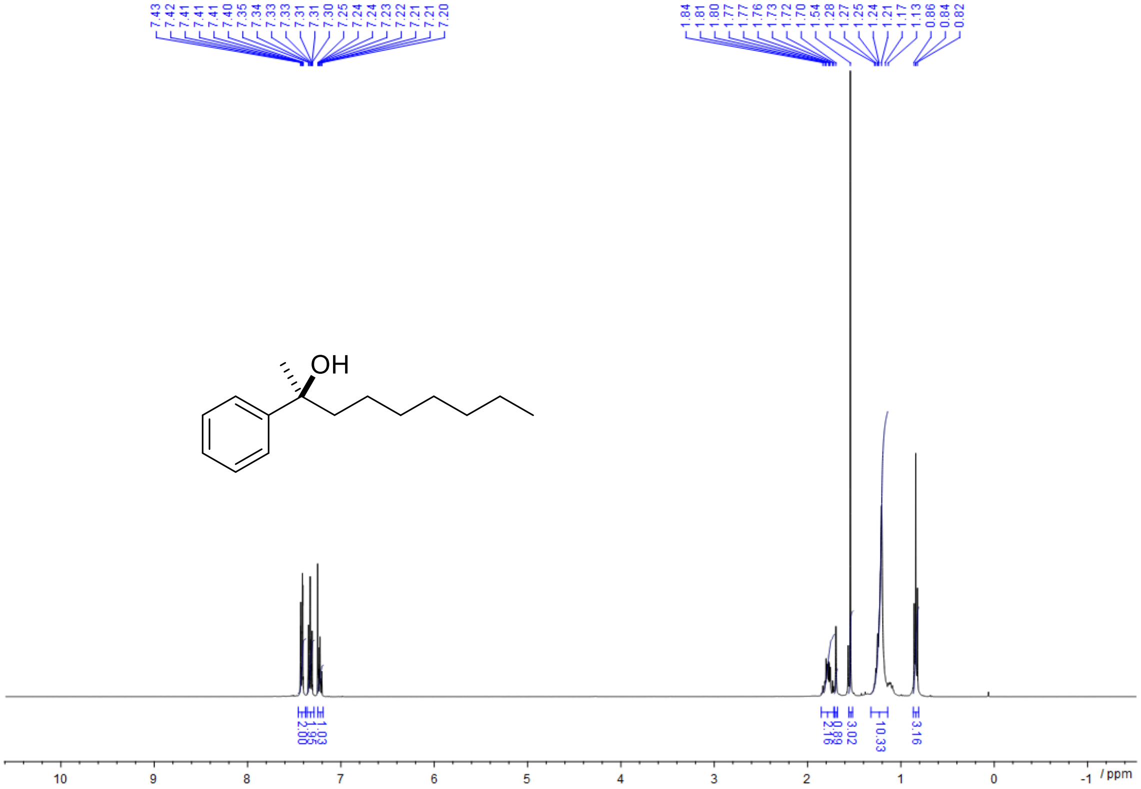

^{13}C NMR

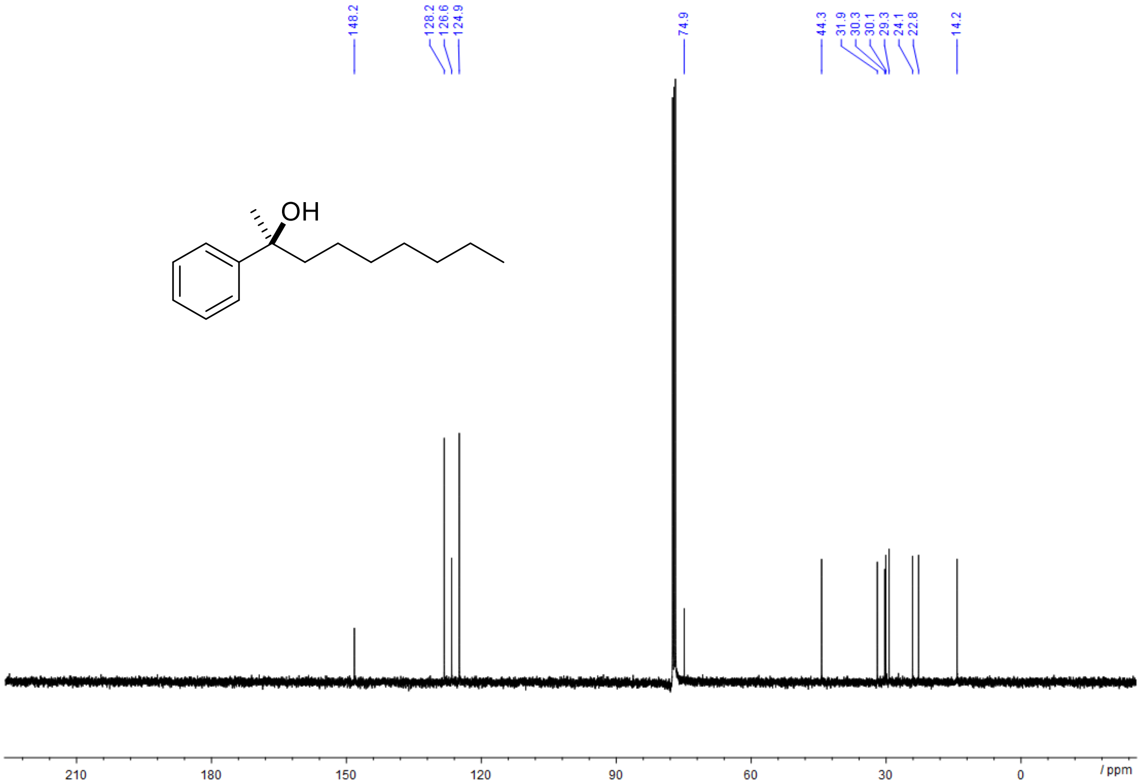

(*S*)-7-chloro-2-phenylheptan-2-ol (2i)

^{1}H NMR

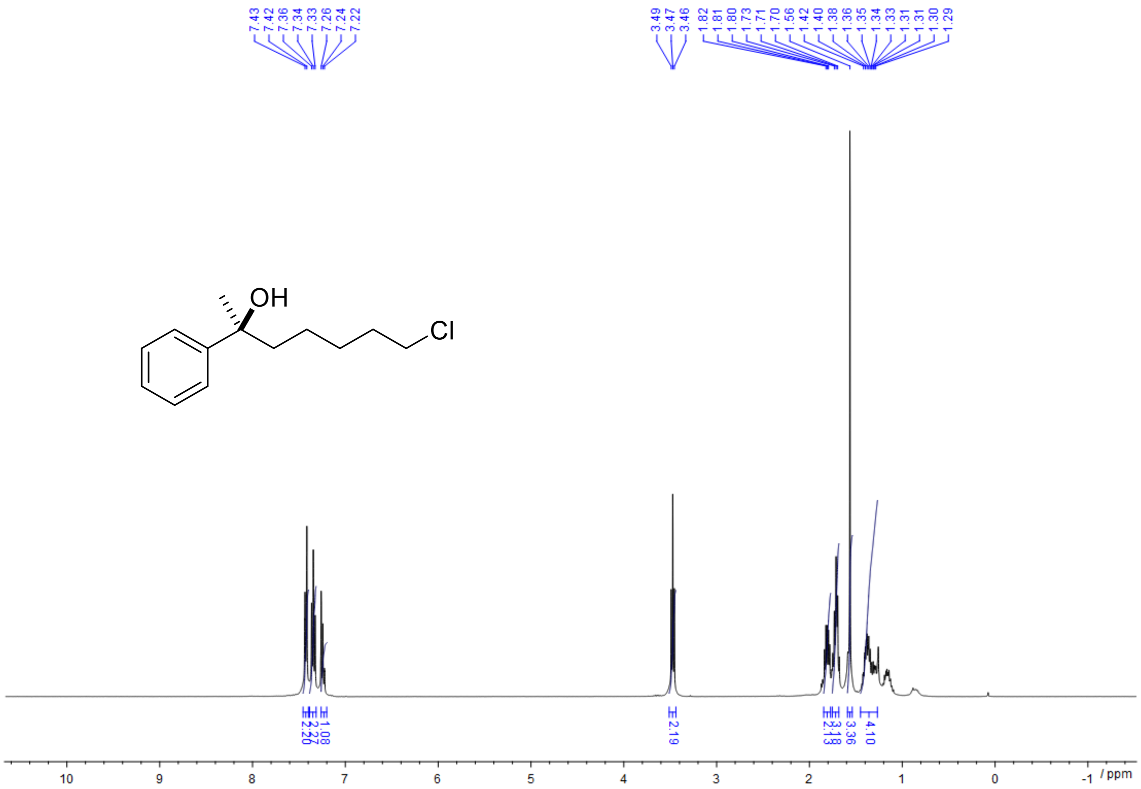

^{13}C NMR

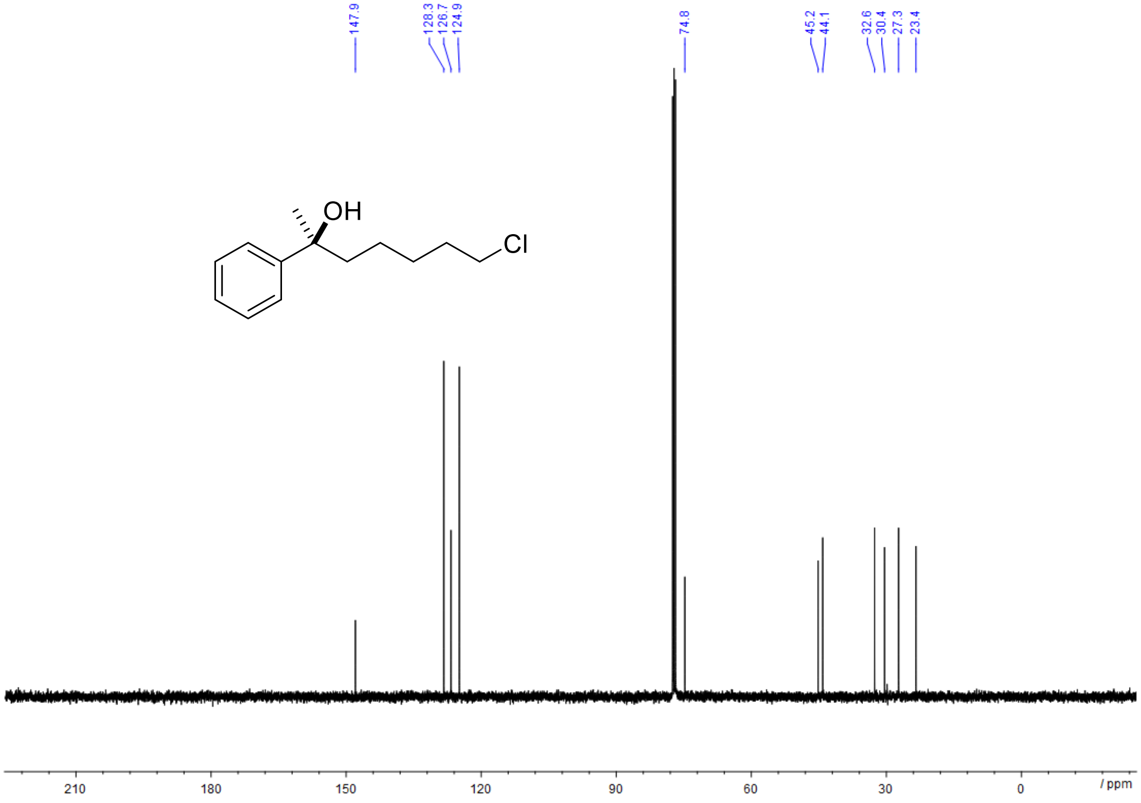

(*S*)-7-methoxy-2-phenylheptan-2-ol (2j)

^{1}H NMR

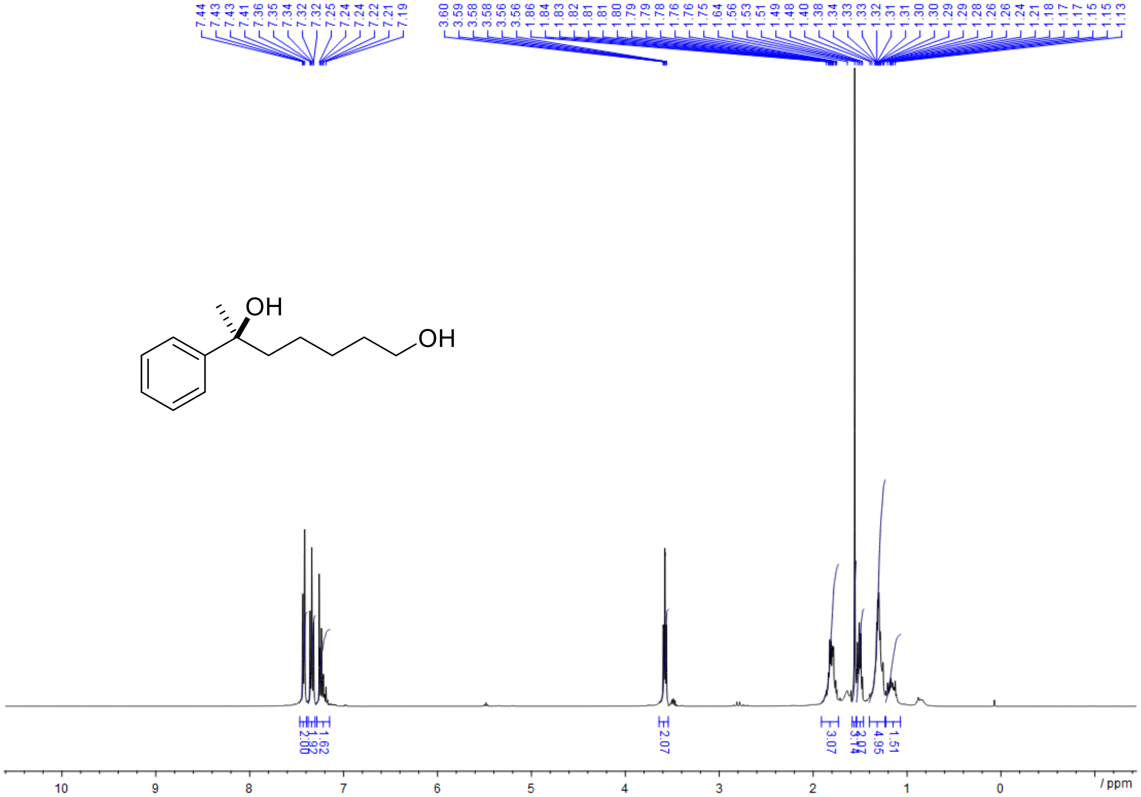

^{13}C NMR

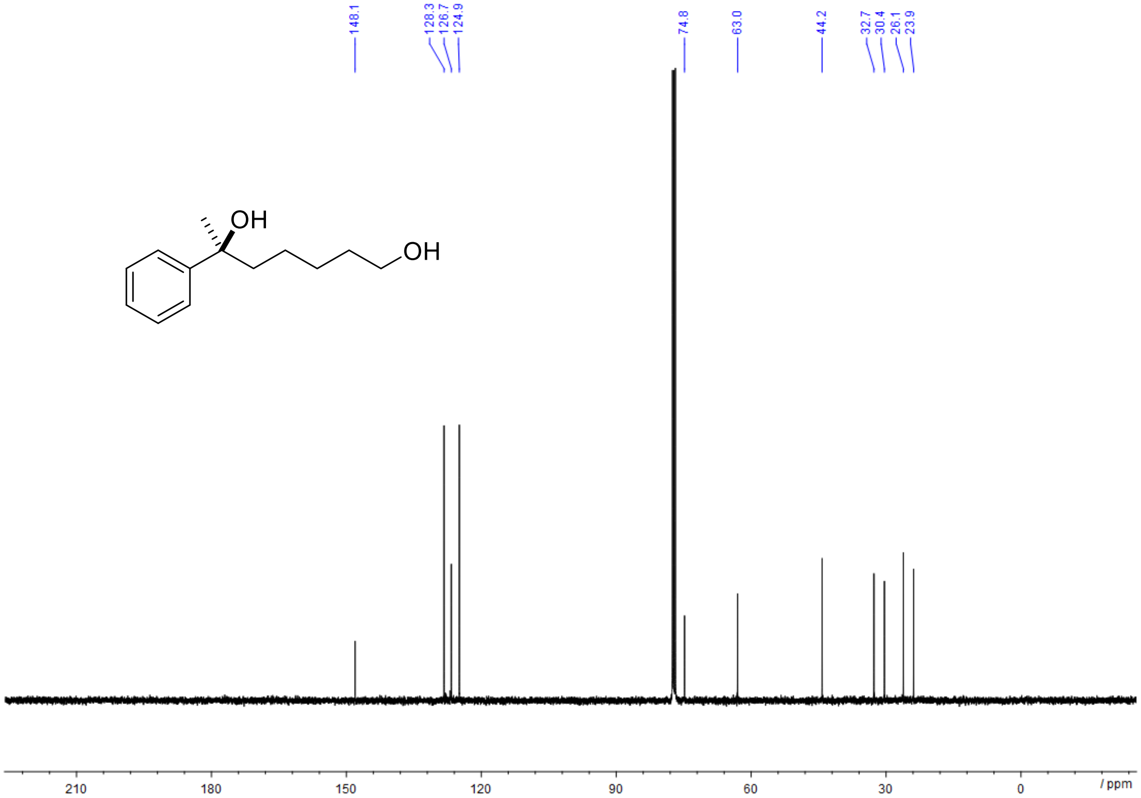

(*S*)-7-methoxy-2-phenylheptan-2-ol (2k)

^{1}H NMR

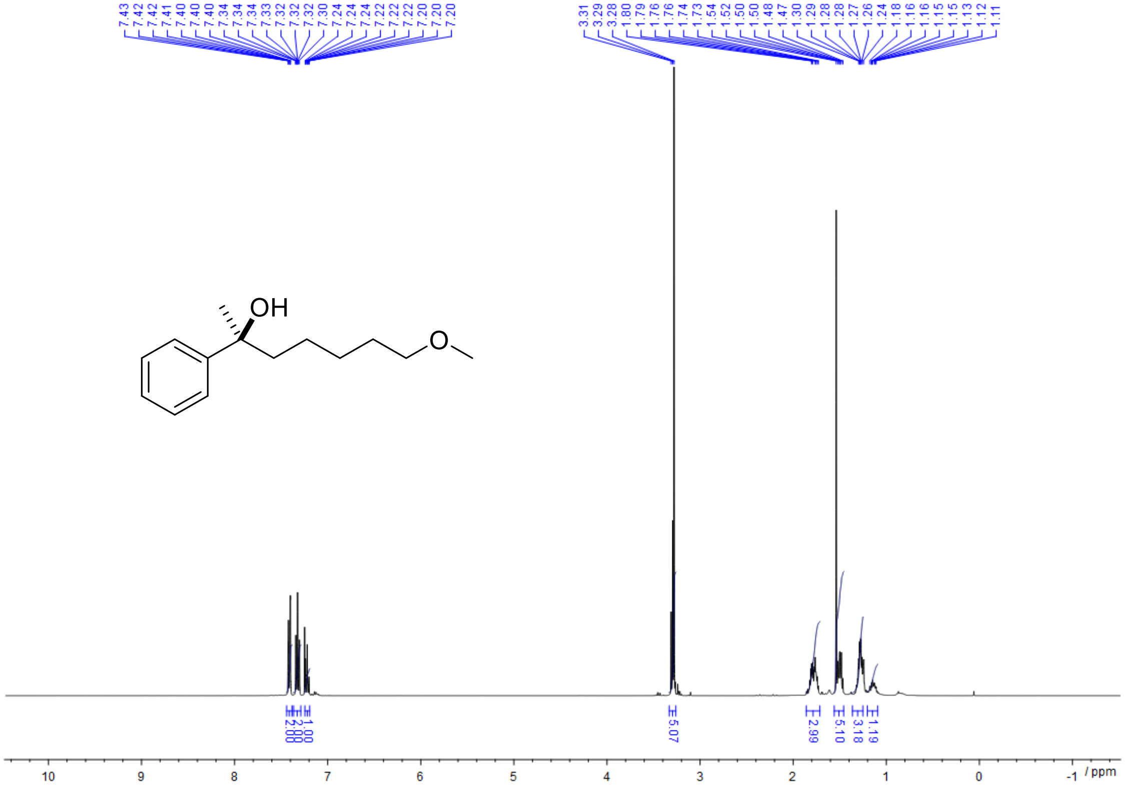

^{13}C NMR

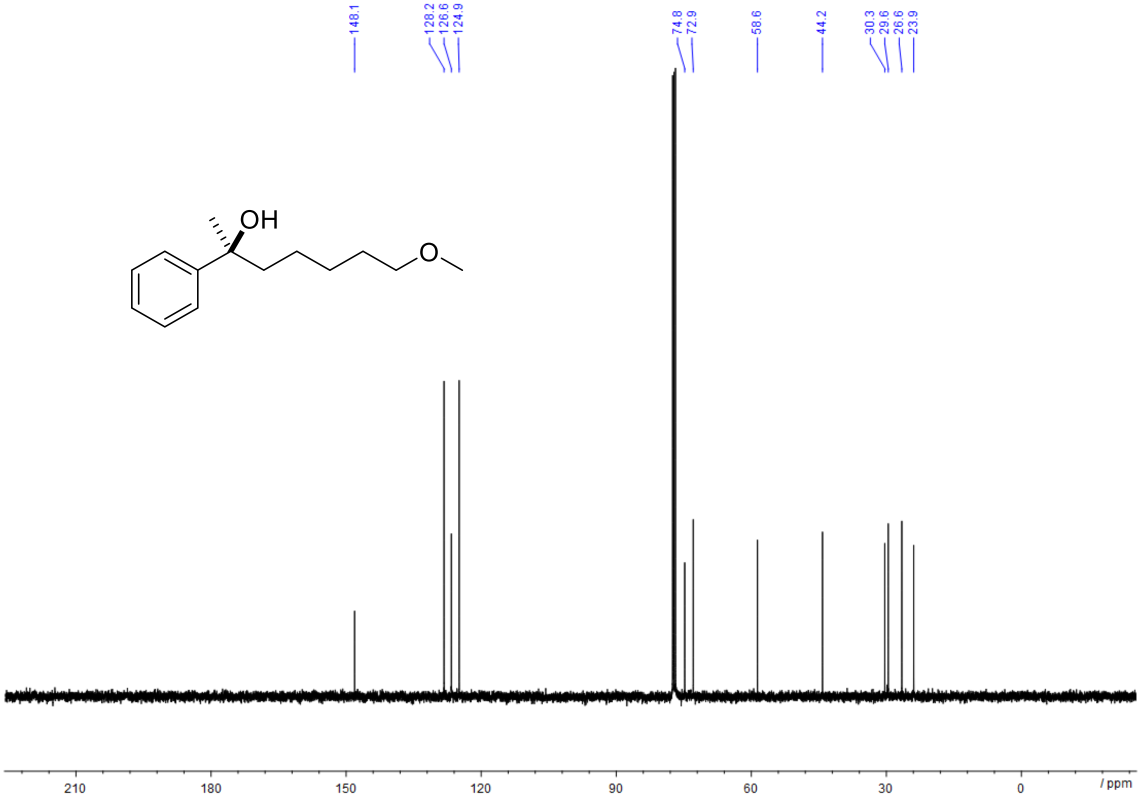

(*S*)-6-methyl-2-(p-tolyl)hept-5-en-2-ol (2l)

^{1}H NMR

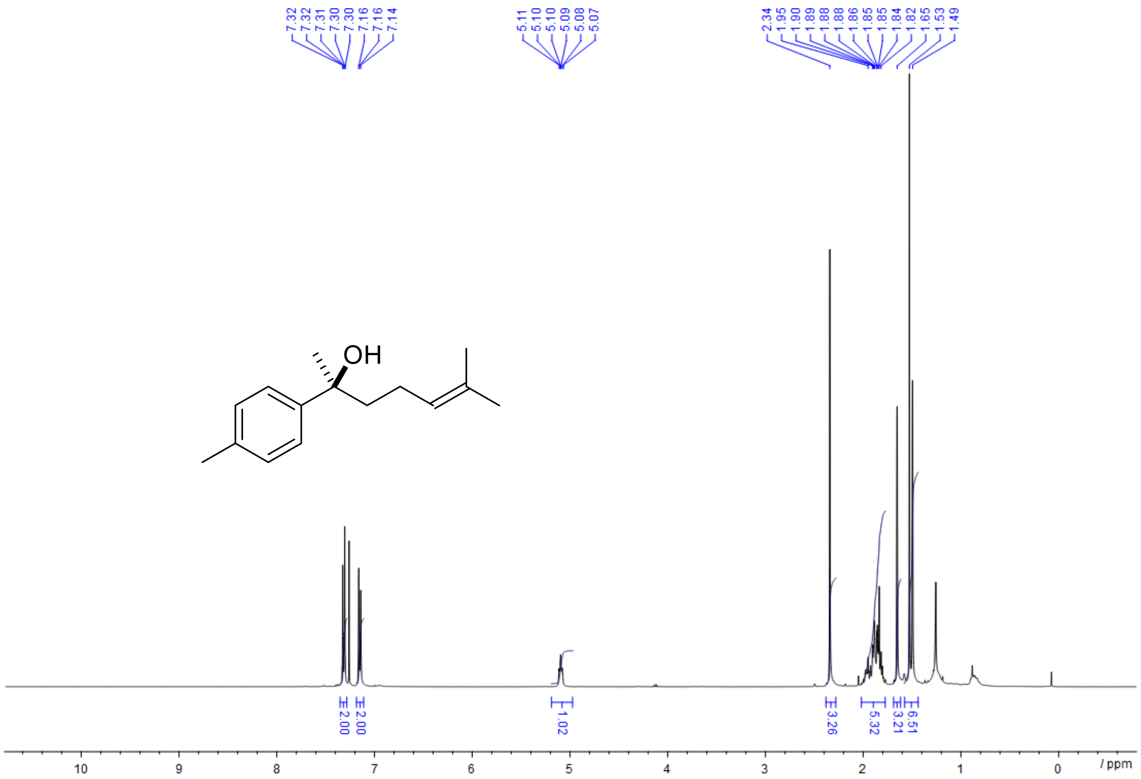

^{13}C NMR

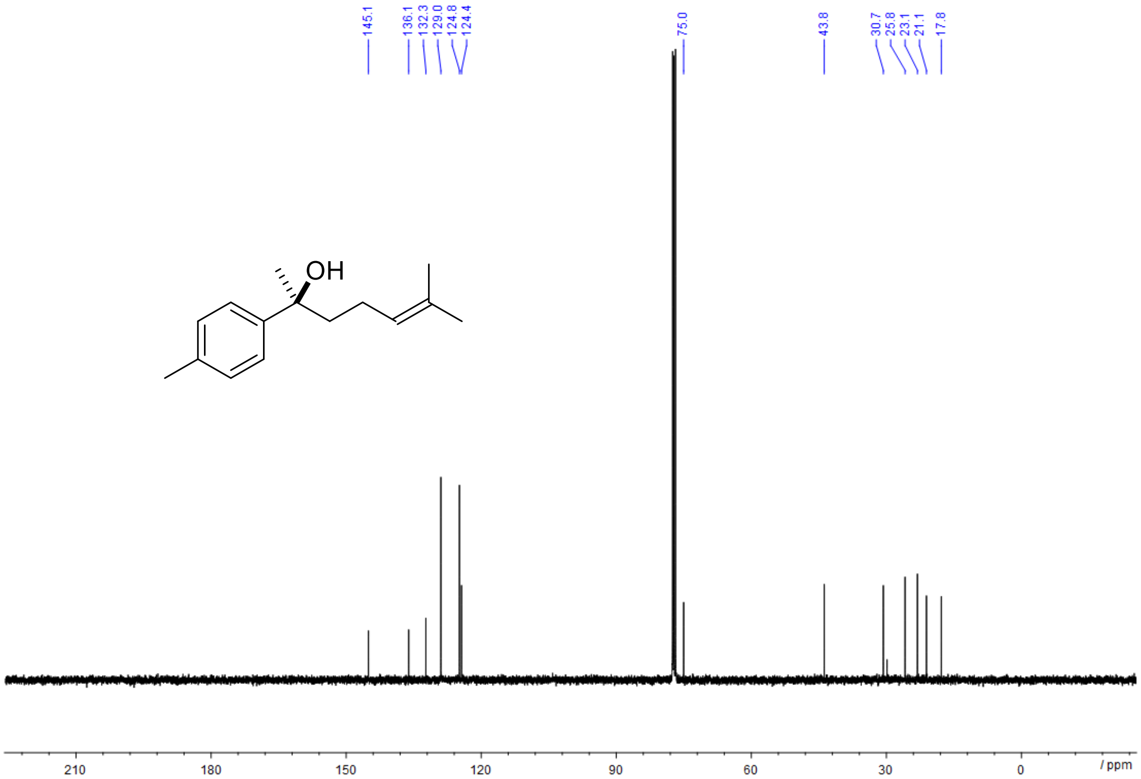

2-(cyclohex-1-en-1-yl)octan-2-ol (2m)

^{1}H NMR

//133.87.249.34/disk1/data/ws1-data/HTN/HTN1201-sil_Proton-1-1.jdf
HTN1201-sil

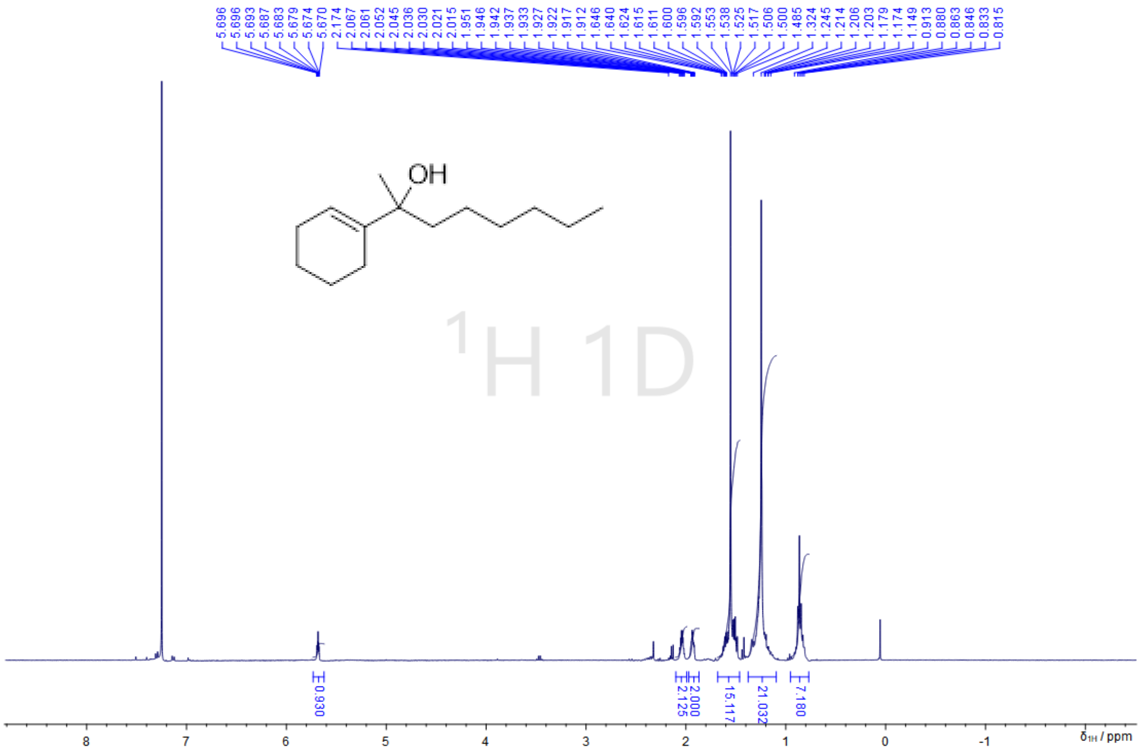

^{13}C NMR

//133.87.249.34/disk1/data/ws2-data/HTN/HTN1231-sil1-c2_Carbon-1-1.jdf
HTN1231-sil1-c2

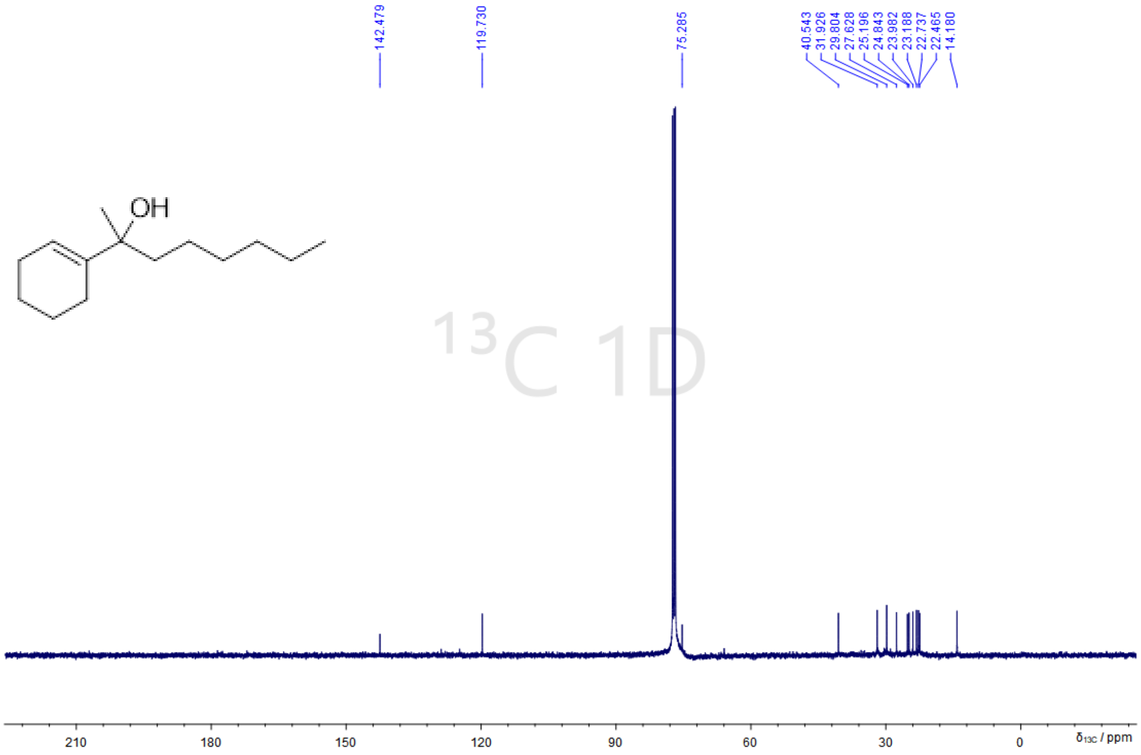

2-cyclohexylhexan-2-ol (2n)

^{1}H NMR

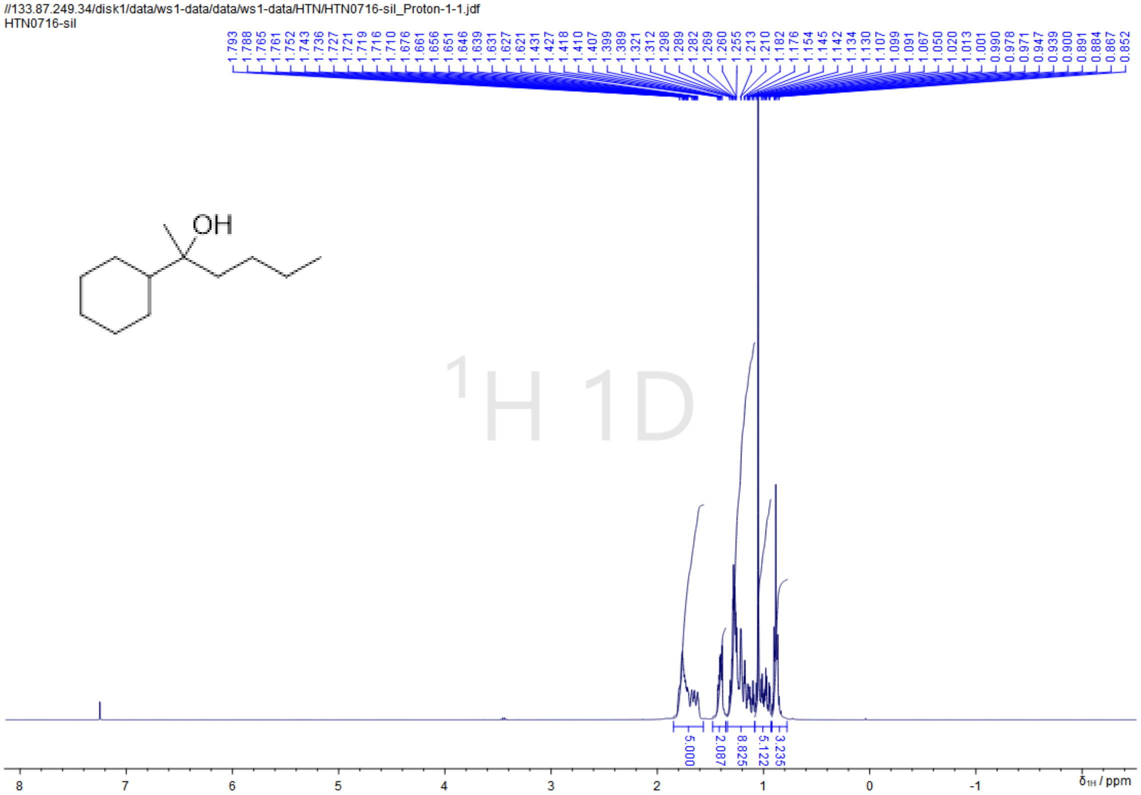

^{13}C NMR

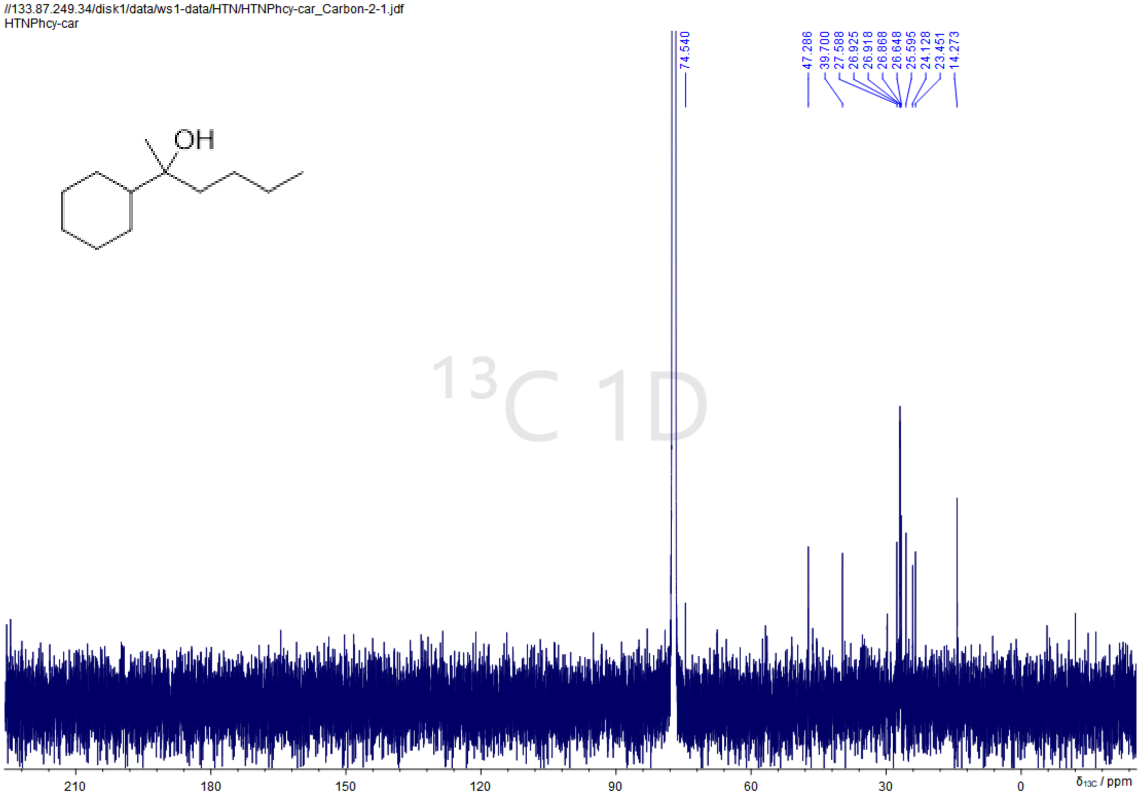

(2-methoxyhexan-2-yl)benzene

^{1}H NMR

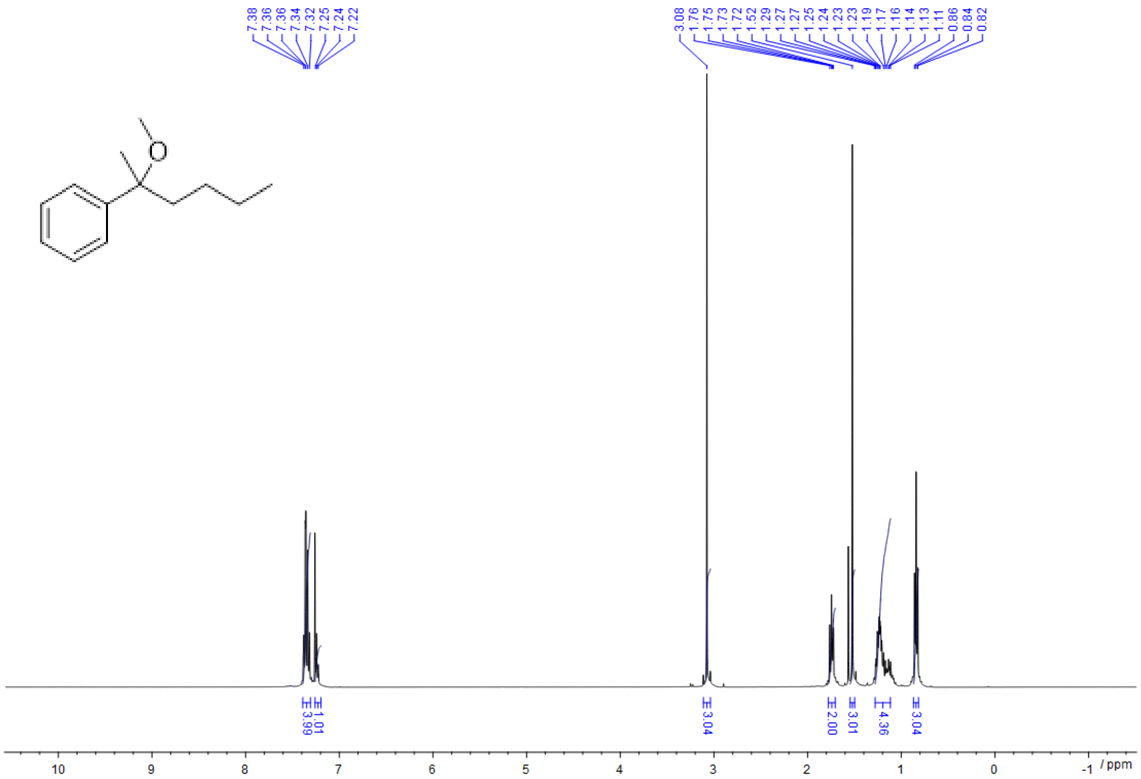

^{13}C NMR

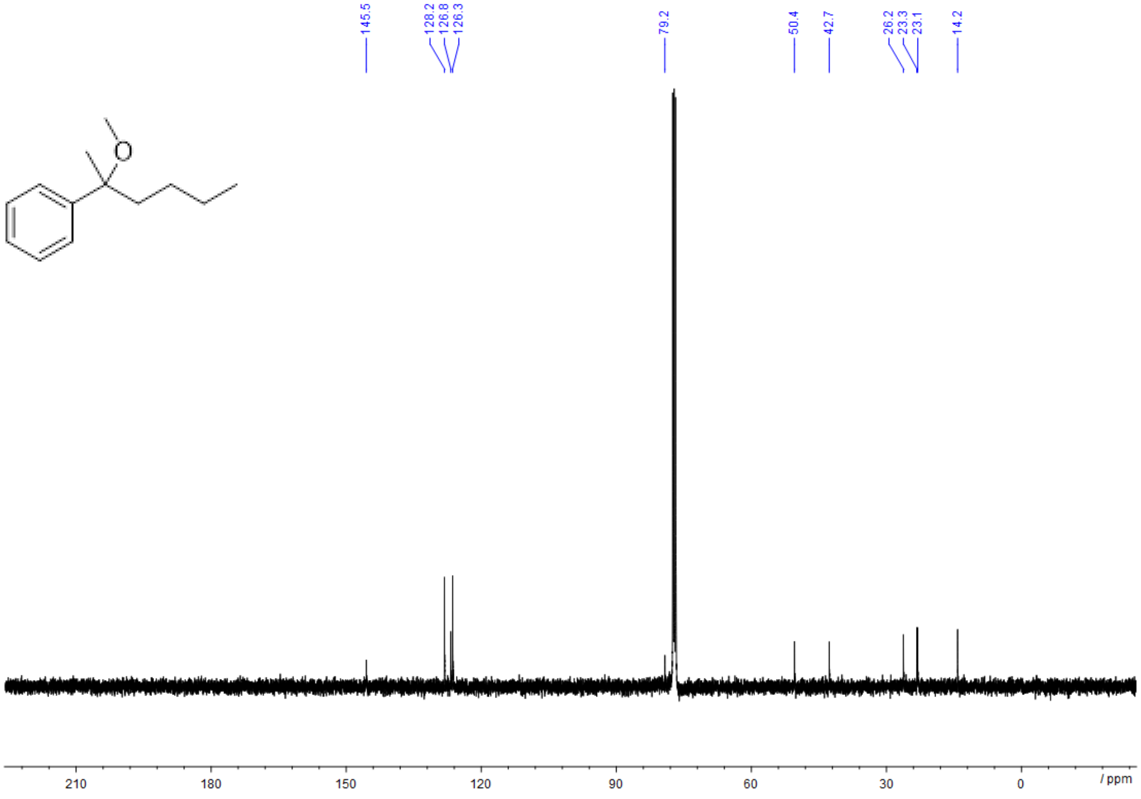

(*S*,*S*)-3,3'- di(spiro[cyclopentane-1,9'-fluoren]-2'-yl)-$C_{10}F_{21}$-IDPi (3c)

^{1}H NMR

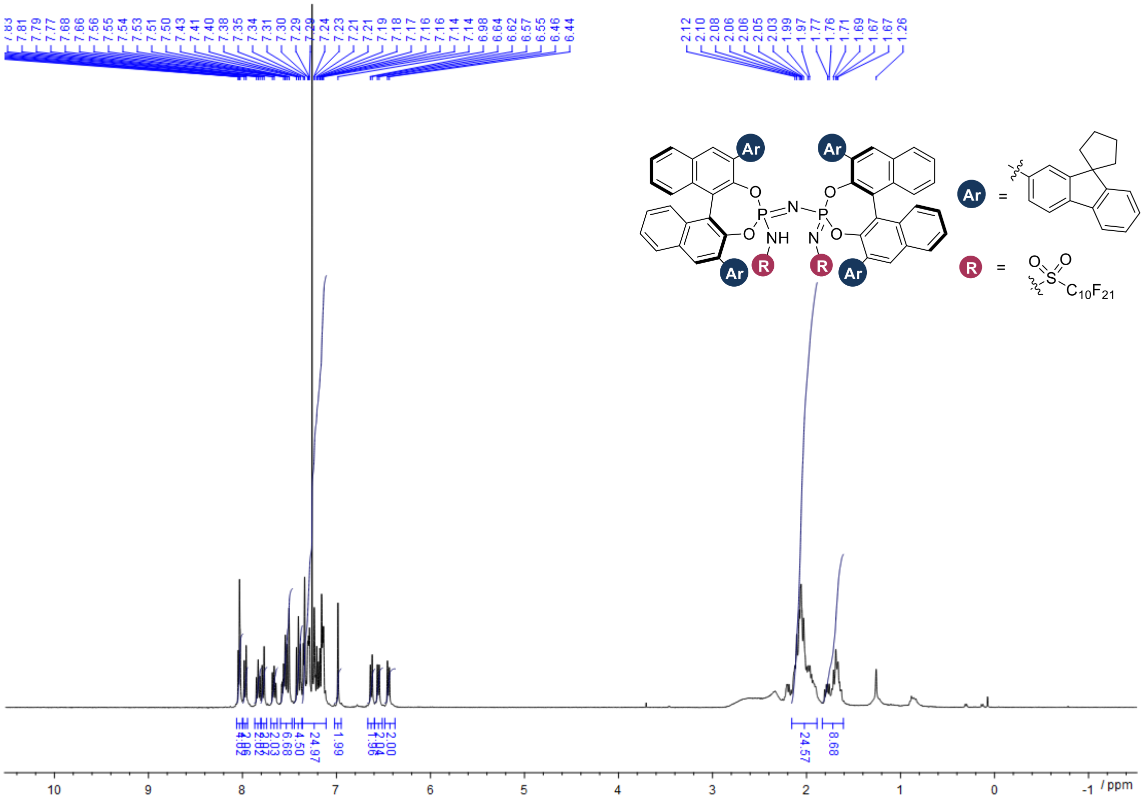

^{13}C NMR

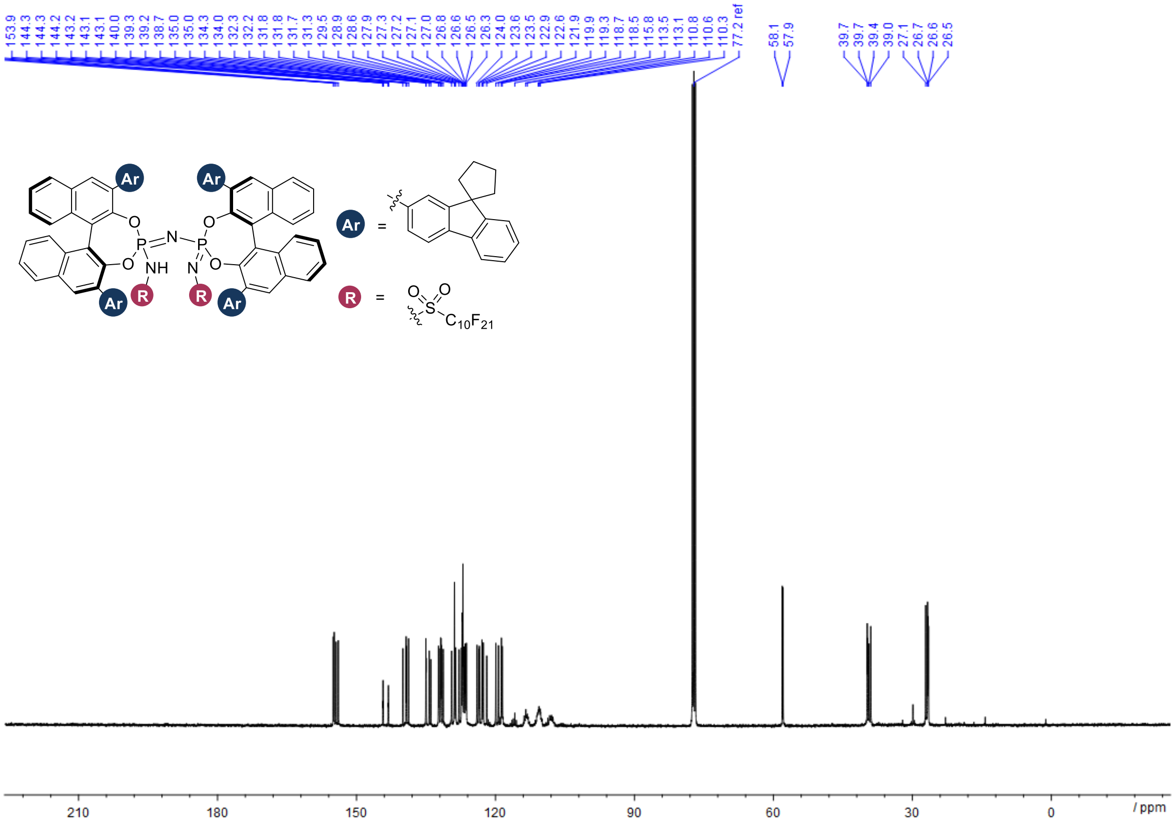

^{31}P NMR

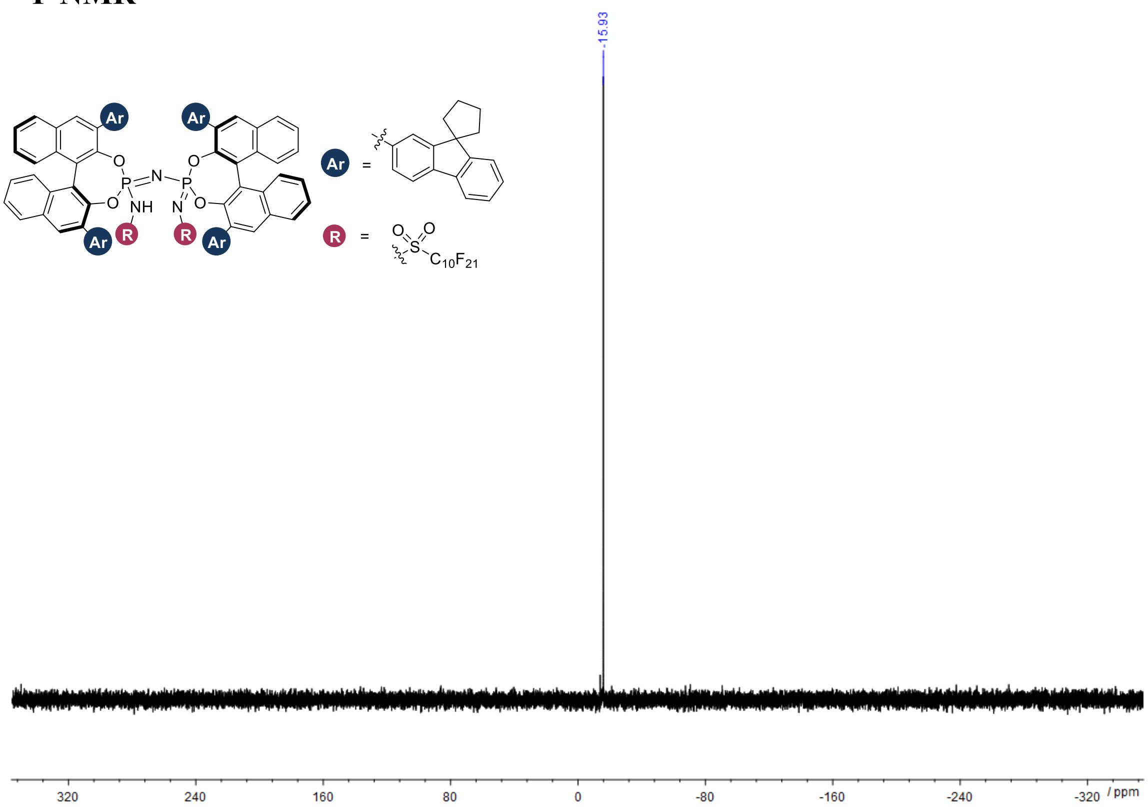

^{19}F NMR

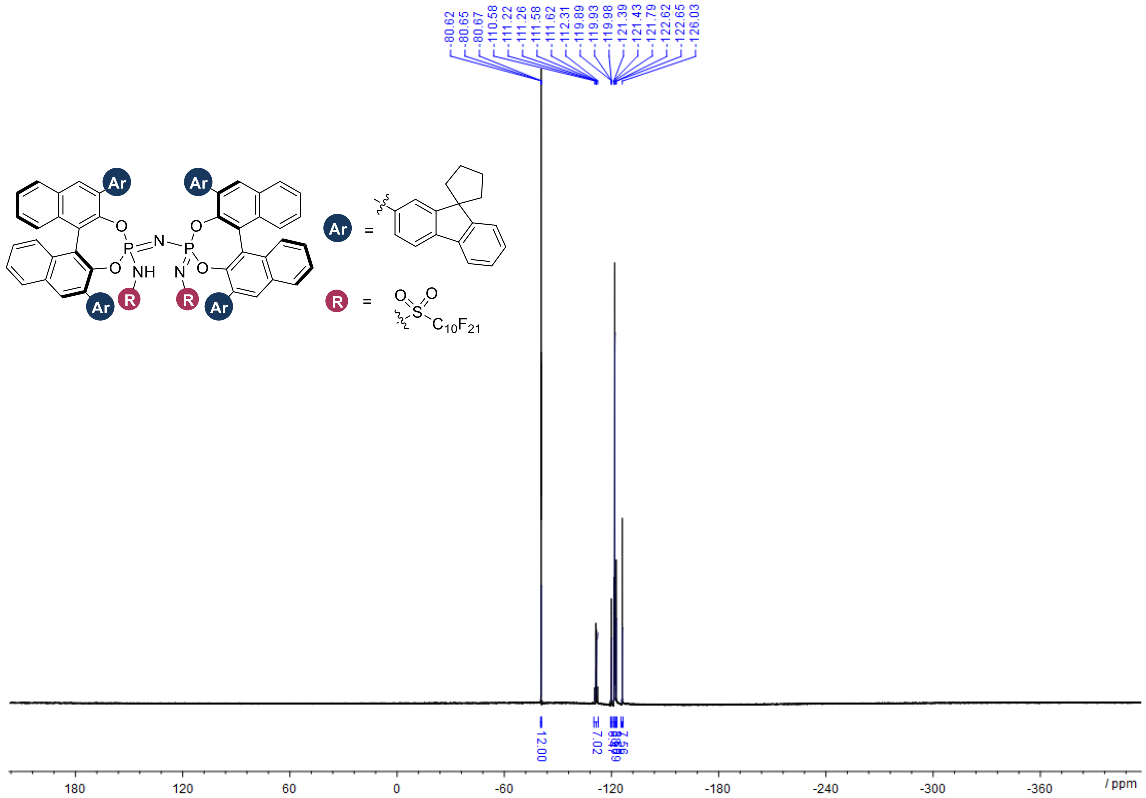

HPLC traces

(*rac*)- and (*S*)-2-phenylhexan-2-ol (2a)

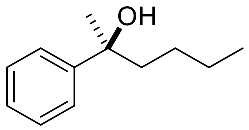

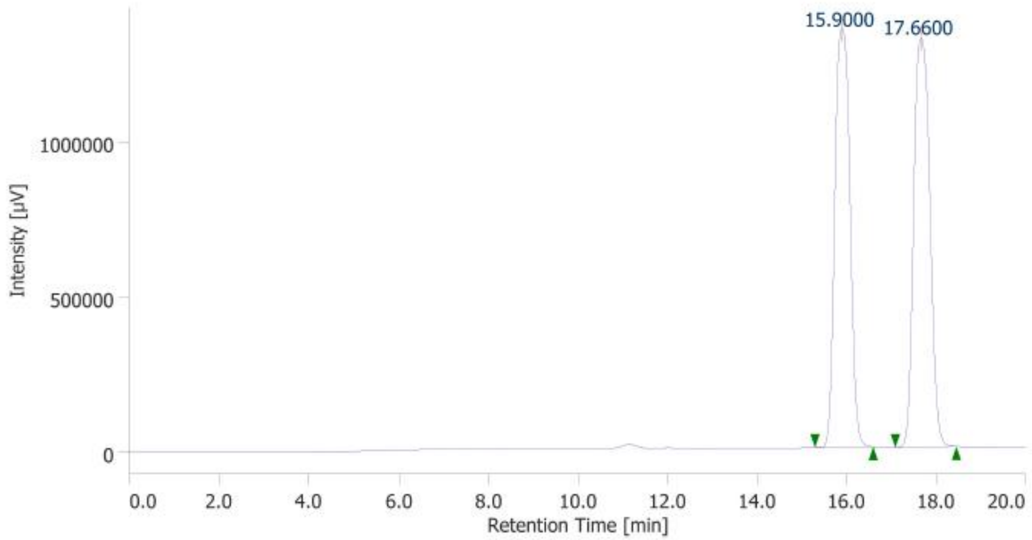

Peak Information

#	tR [min]	Area [µV·sec]	Area%
1	15.900	31568795	48.865
2	17.660	33034855	51.135

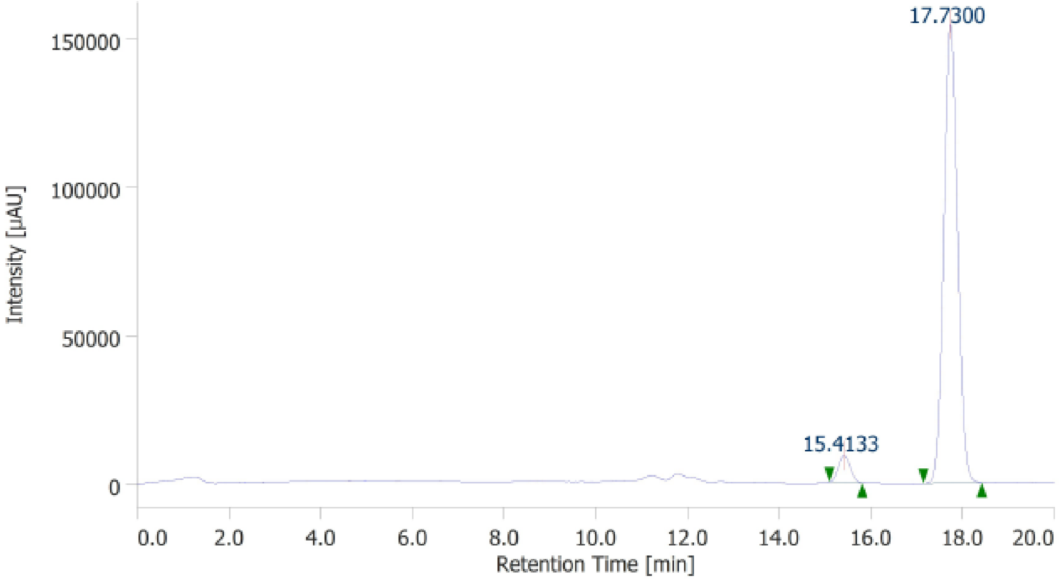

Peak Information

#	tR [min]	Area [µV·sec]	Area%
1	15.413	163274	4.837
2	17.730	3212529	95.163

(*rac*)- and (*S*)-2-(*p*-tolyl)hexan-2-ol (2b)

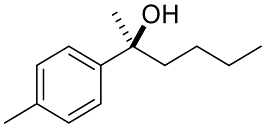

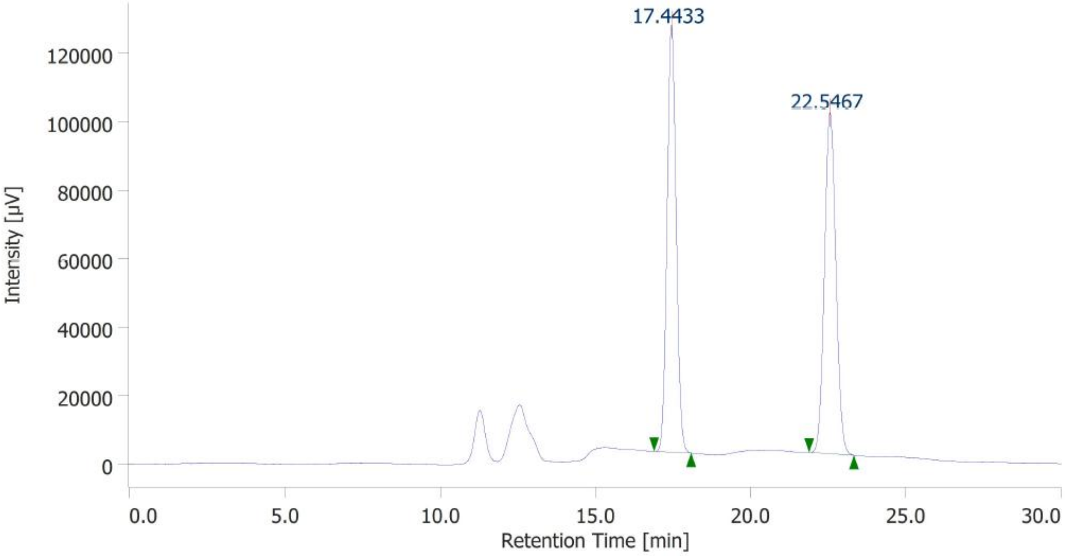

Peak Information

#	tR [min]	Area [μV·sec]	Area%
1	17.443	2544274	50.026
2	22.547	2541638	49.974

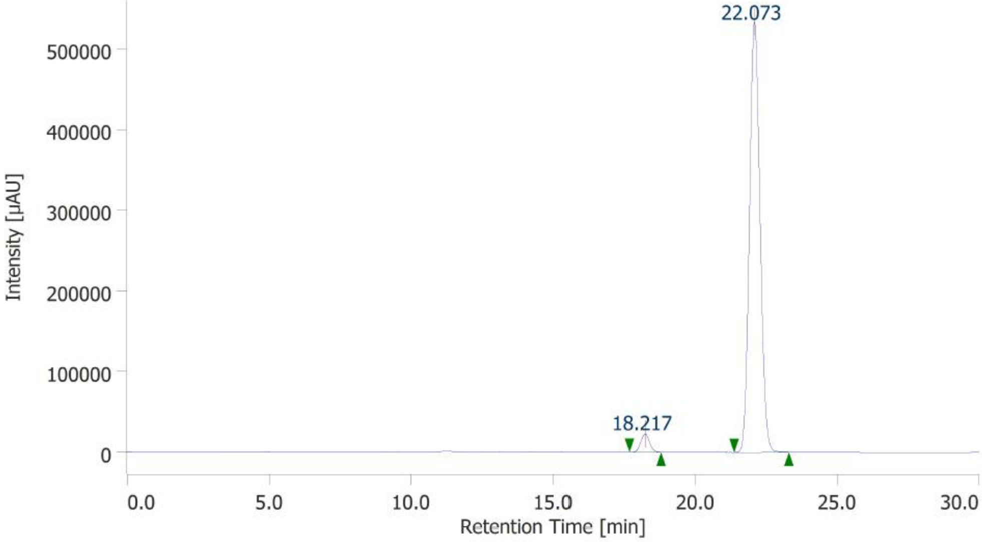

Peak Information

#	tR [min]	Area [μV·sec]	Area%
1	18.217	484376	3.415
2	22.073	13699987	96.585

(*rac*)- and (*S*)-2-(3-methoxyphenyl)hexan-2-ol (2c)

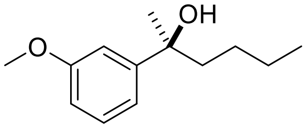

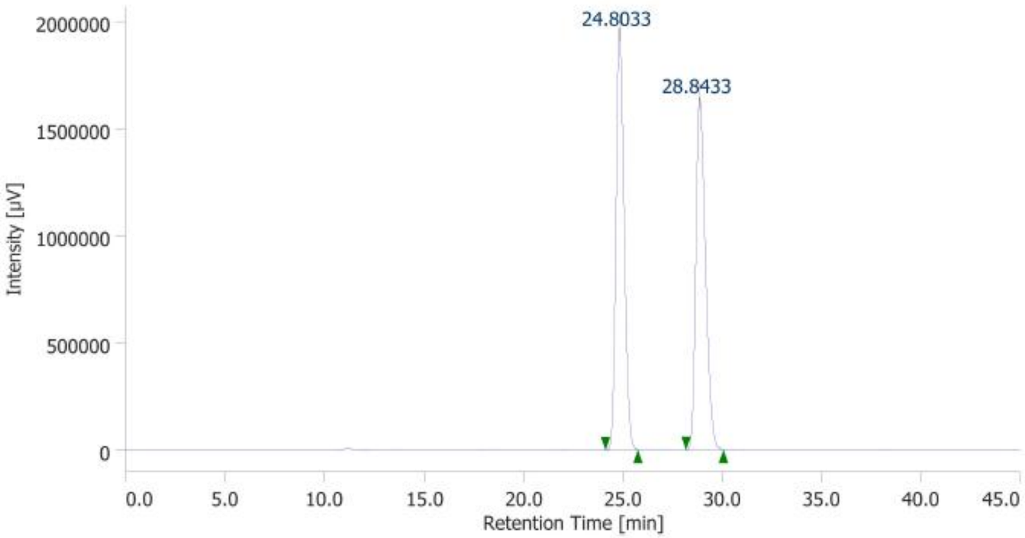

Peak Information

#	tR [min]	Area [µV·sec]	Area%
1	24.803	55617925	50.128
2	28.843	55333353	49.872

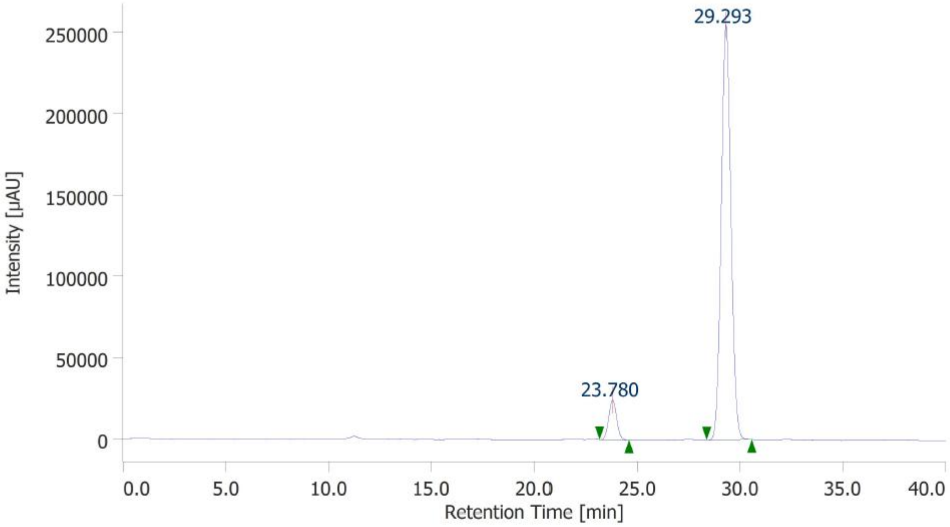

Peak Information

#	tR [min]	Area [µV·sec]	Area%
1	23.780	661344	7.301
2	29.293	8396871	92.699

(*rac*)- and (*S*)-2-(4-chlorophenyl)hexan-2-ol (2d)

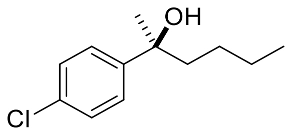

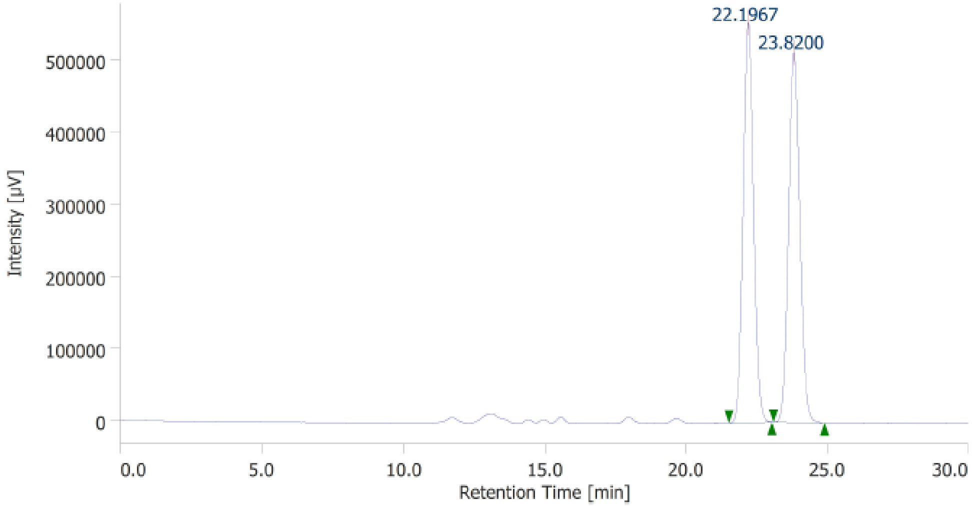

Peak Information

#	tR [min]	Area [µV·sec]	Area%
1	22.197	14261572	50.078
2	23.820	14217398	49.922

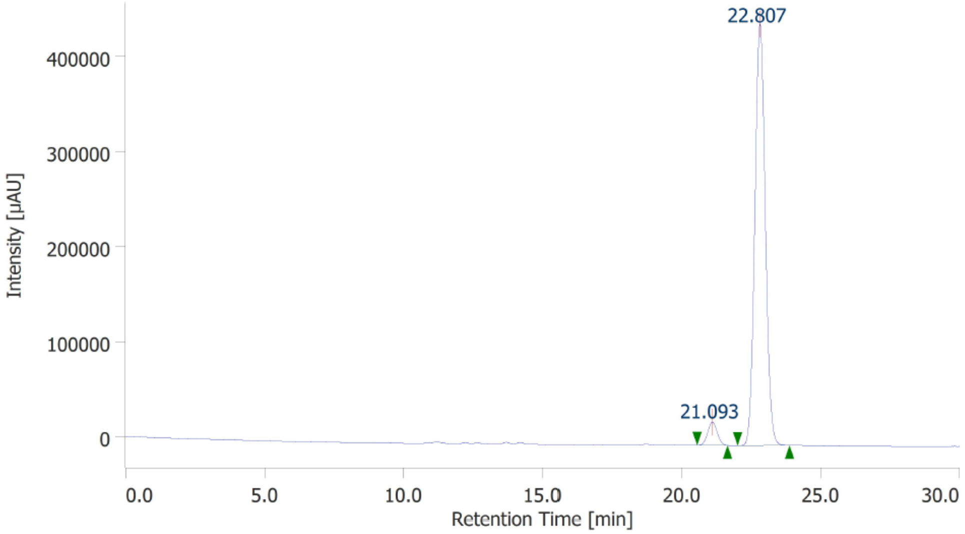

Peak Information

#	tR [min]	Area [µV·sec]	Area%
1	21.093	591176	4.847
2	22.807	11605551	95.153

(*rac*)- and (*S*)-2-(4-bromophenyl)hexan-2-ol (2e)

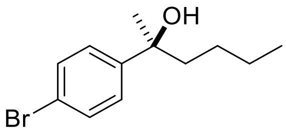

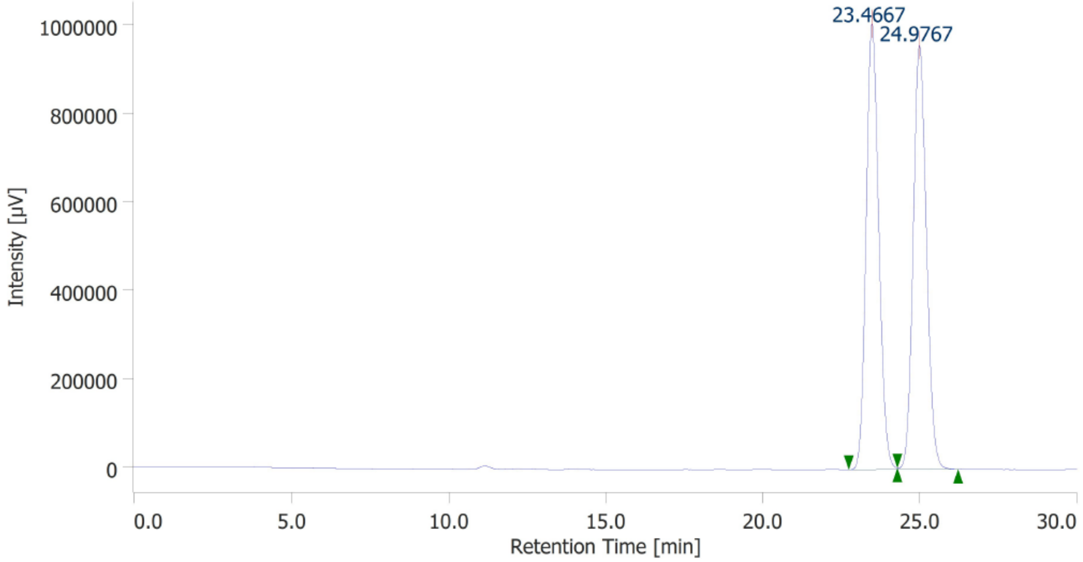

Peak Information

#	tR [min]	Area [µV·sec]	Area%
1	23.467	28182035	50.037
2	24.977	28139922	49.963

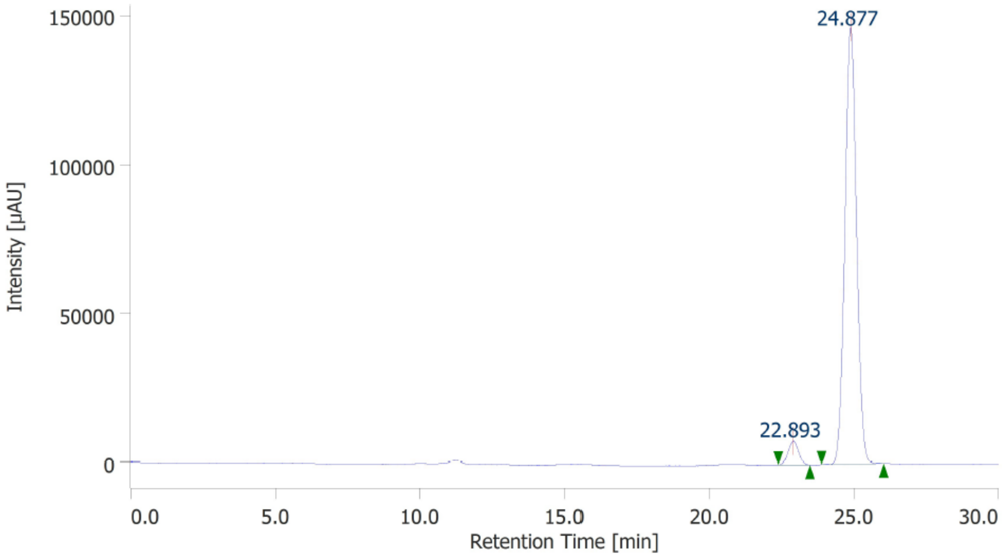

Peak Information

#	tR [min]	Area [µV·sec]	Area%
1	22.893	206599	4.767
2	24.877	4127138	95.233

(*rac*)- and (*S*)- 2-phenylbutan-2-ol (2f)

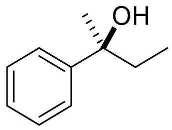

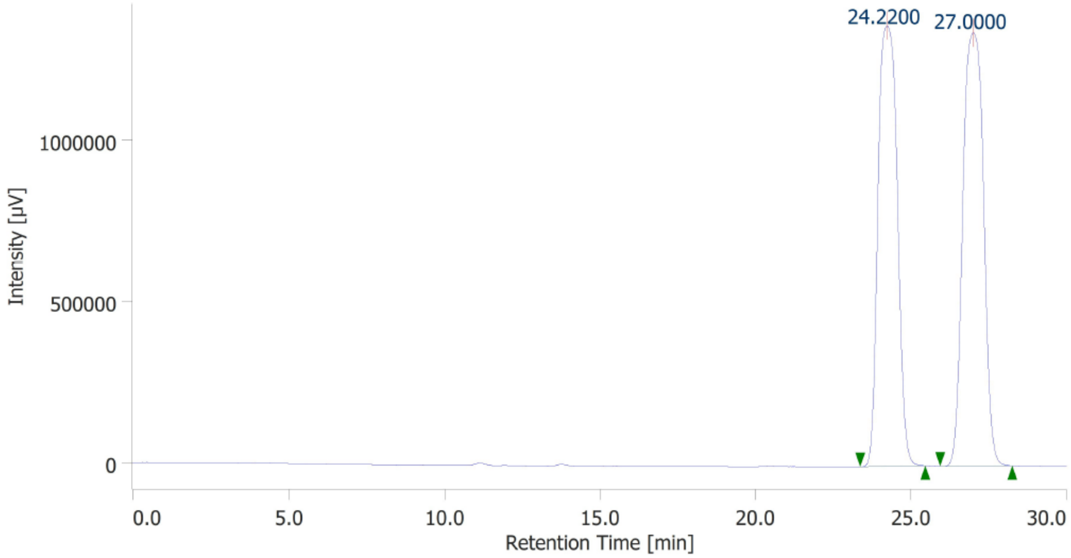

Peak Information

#	tR [min]	Area [μV·sec]	Area%
1	24.220	58508696	48.602
2	27.000	61874974	51.398

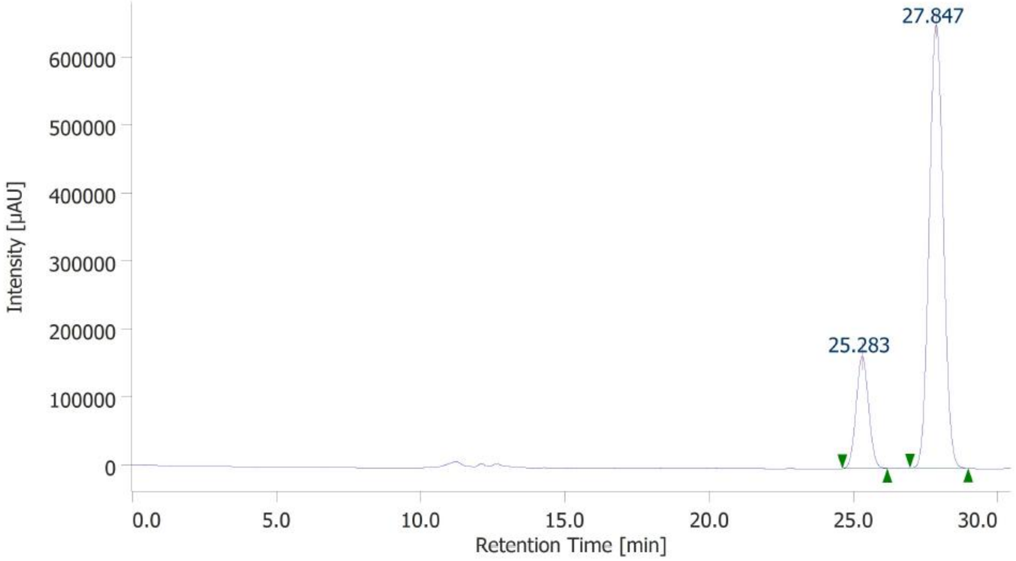

Peak Information

#	tR [min]	Area [μV·sec]	Area%
1	25.283	4972110	18.283
2	27.847	22223600	81.717

(*rac*)- and (*S*)-2-phenylpentan-2-ol (2g)

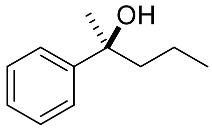

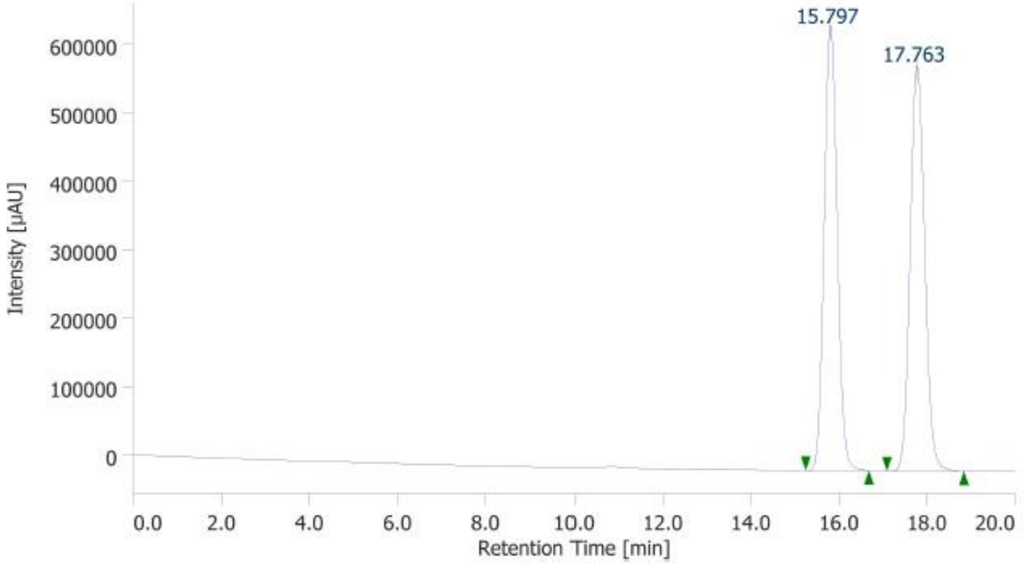

Peak Information

#	tR [min]	Area [µV·sec]	Area%
1	15.797	13377913	49.671
2	17.763	13555386	50.329

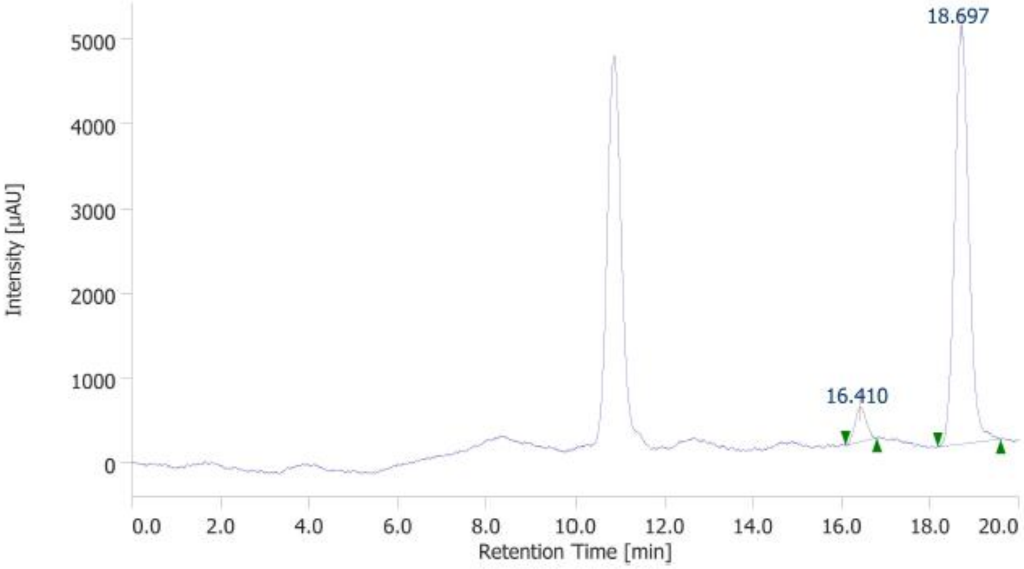

Peak Information

#	tR [min]	Area [µV·sec]	Area%
1	16.410	7225	6.160
2	18.697	110070	93.840

(*rac*)- and (*S*)-2-phenylnonan-2-ol (2h)

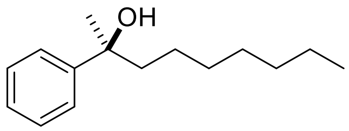

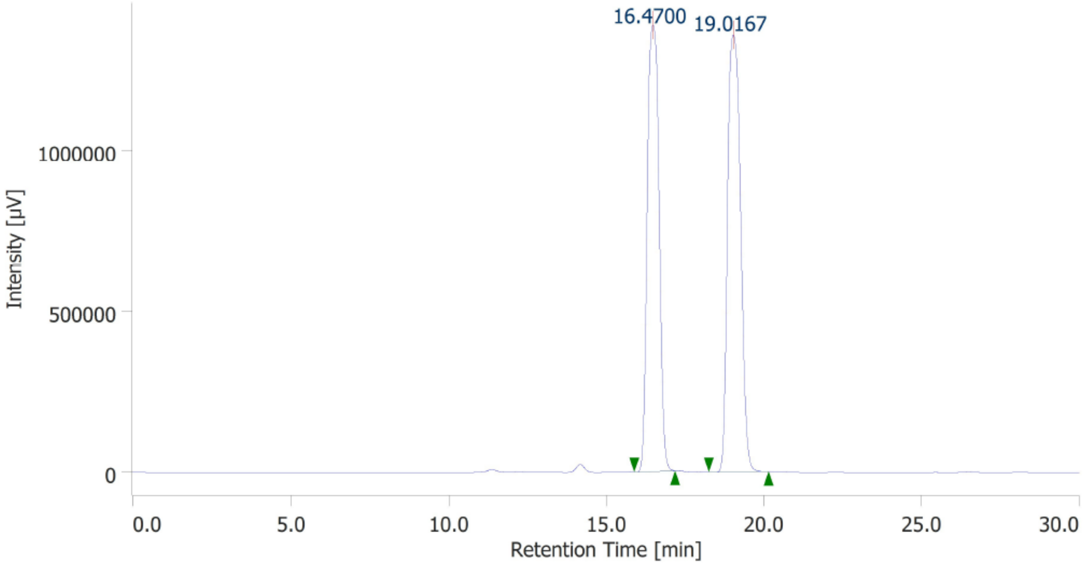

Peak Information

#	tR [min]	Area [µV·sec]	Area%
1	16.470	35863996	48.044
2	19.017	38784344	51.956

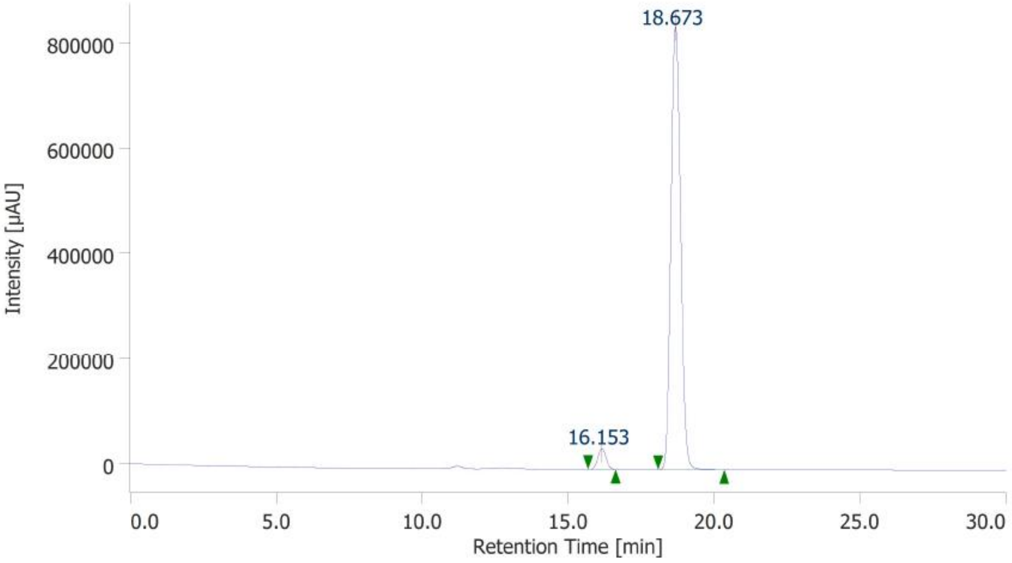

Peak Information

#	tR [min]	Area [µV·sec]	Area%
1	16.153	770316	3.856
2	18.673	19209149	96.144

(*rac*)- and (*S*)-7-chloro-2-phenylheptan-2-ol (2i)

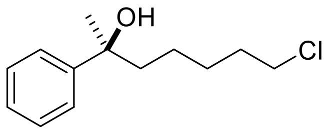

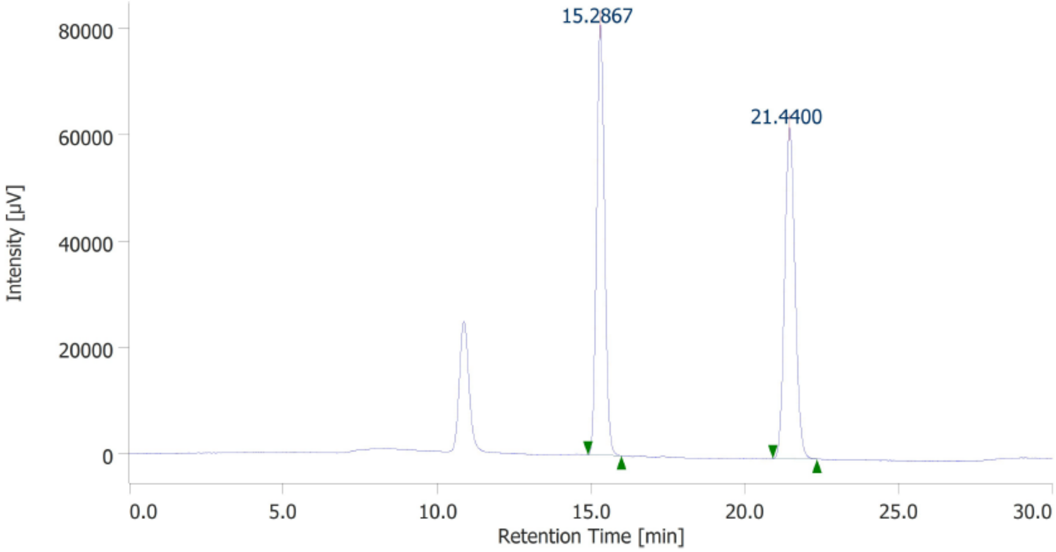

Peak Information

#	tR [min]	Area [µV·sec]	Area%
1	15.287	1455862	49.808
2	21.440	1467073	50.192

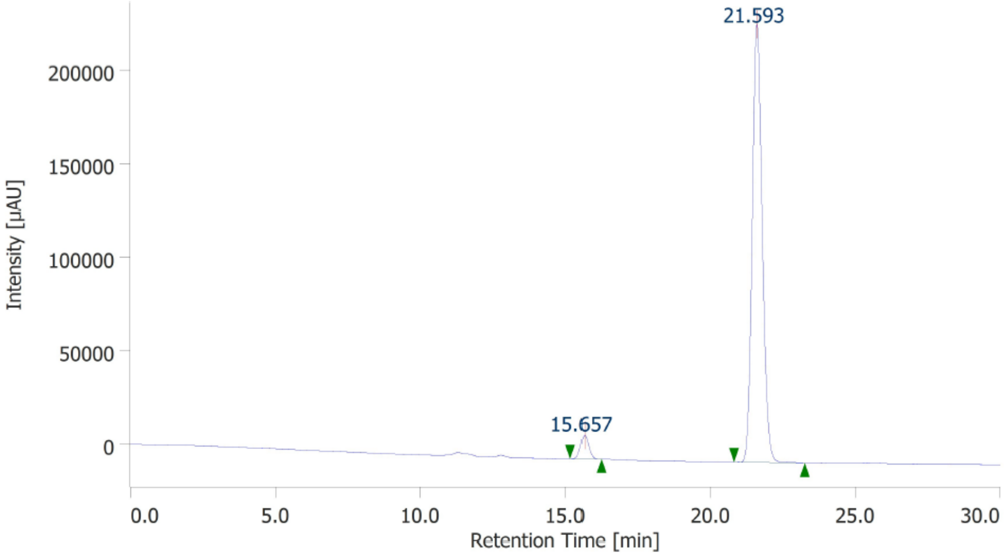

Peak Information

#	tR [min]	Area [µV·sec]	Area%
1	15.657	234318	3.978
2	21.593	5655290	96.022

(*rac*)- and (*S*)-7-methoxy-2-phenylheptan-2-ol (2j)

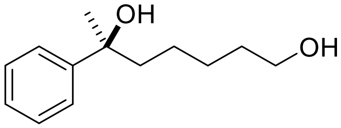

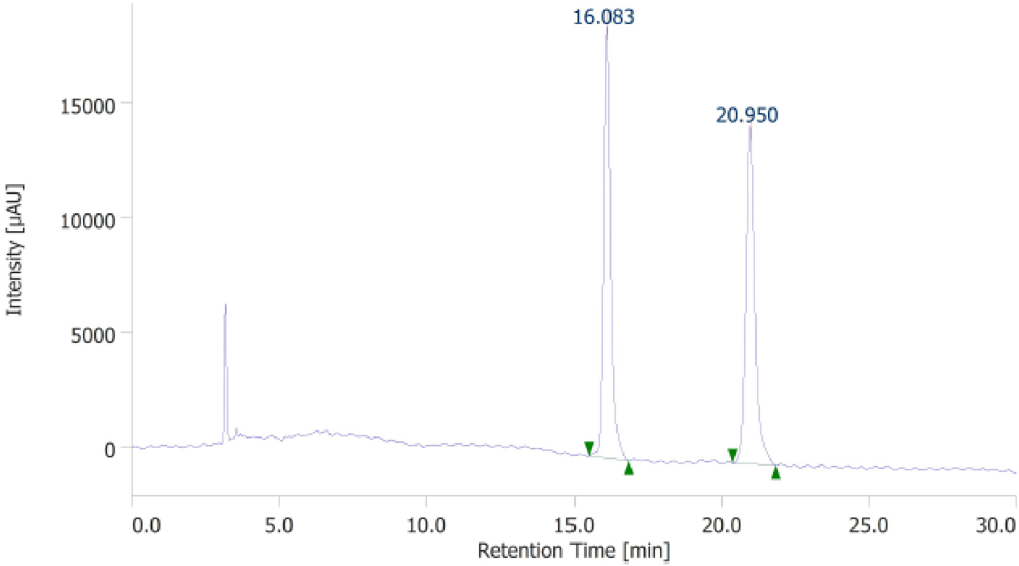

Peak Information

#	tR [min]	Area [µV·sec]	Area%
1	16.083	309827	49.679
2	20.950	313828	50.321

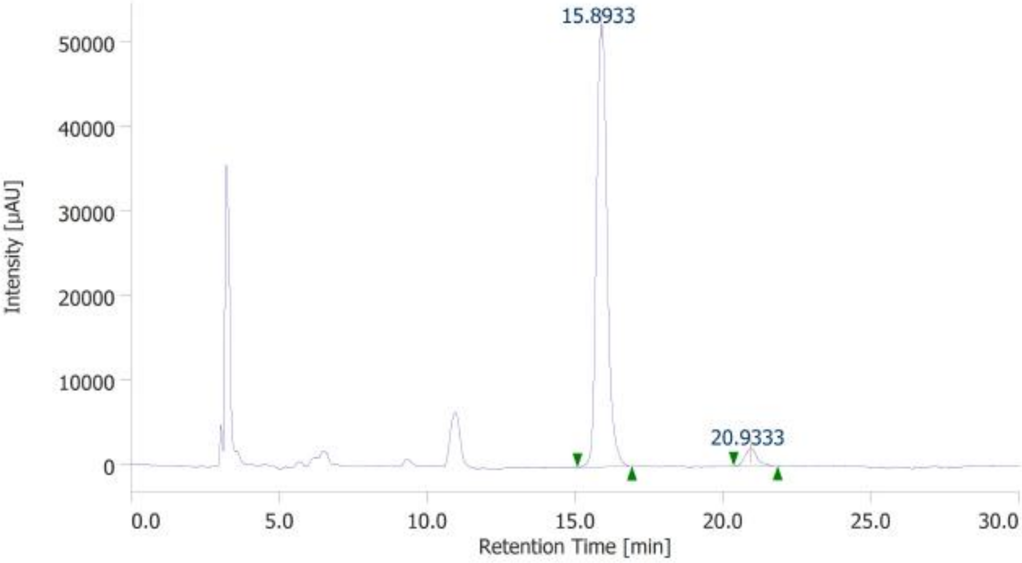

Peak Information

#	tR [min]	Area [µV·sec]	Area%
1	15.893	1318346	95.758
2	20.933	58403	4.242

(*rac*)- and (*S*)-7-methoxy-2-phenylheptan-2-ol (2k)

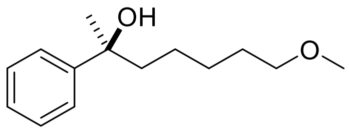

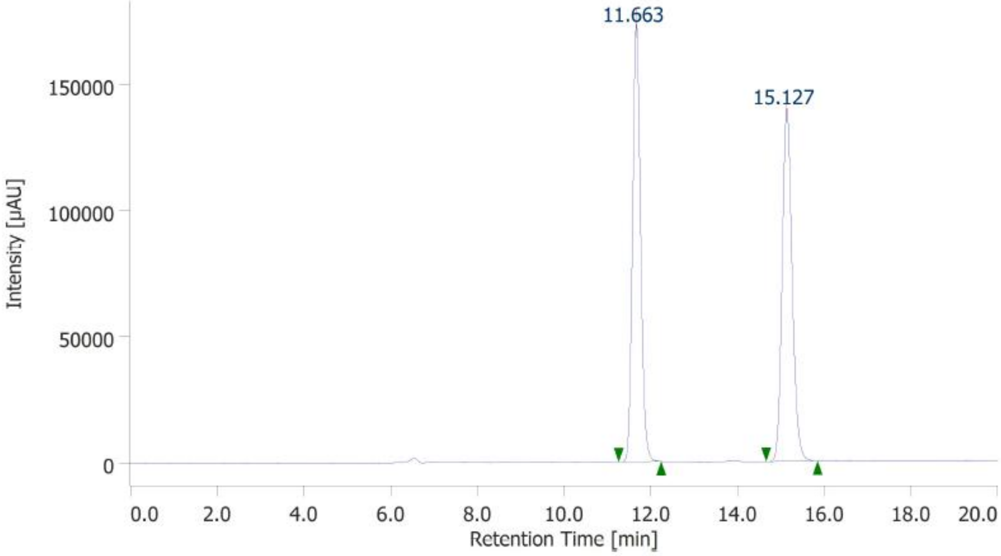

Peak Information

#	tR [min]	Area [µV·sec]	Area%
1	11.663	2215657	49.813
2	15.127	2232288	50.187

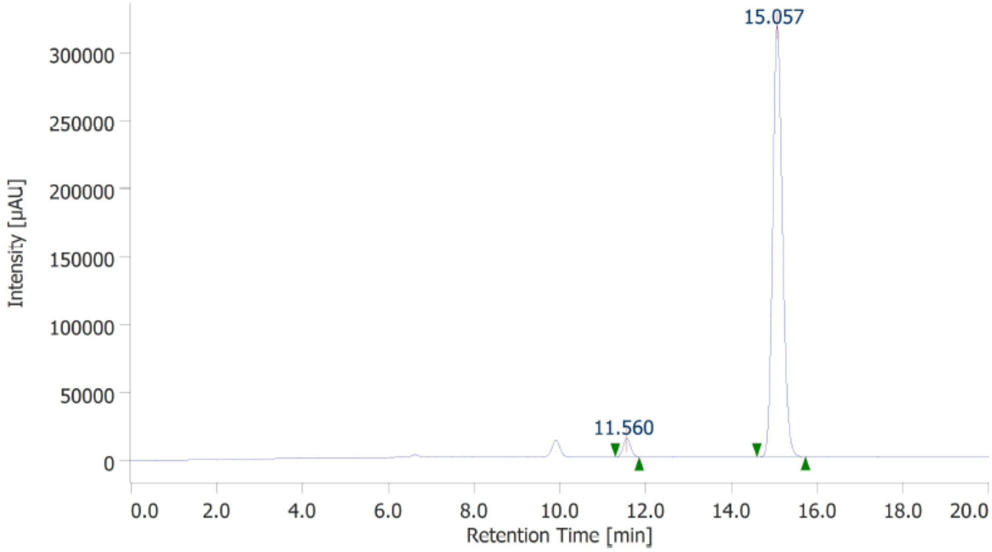

Peak Information

#	tR [min]	Area [µV·sec]	Area%
1	11.560	167103	3.240
2	15.057	4989847	96.760

(*rac*)- and (*S*)-6-methyl-2-(p-tolyl)hept-5-en-2-ol (2l)

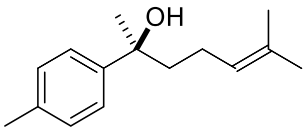

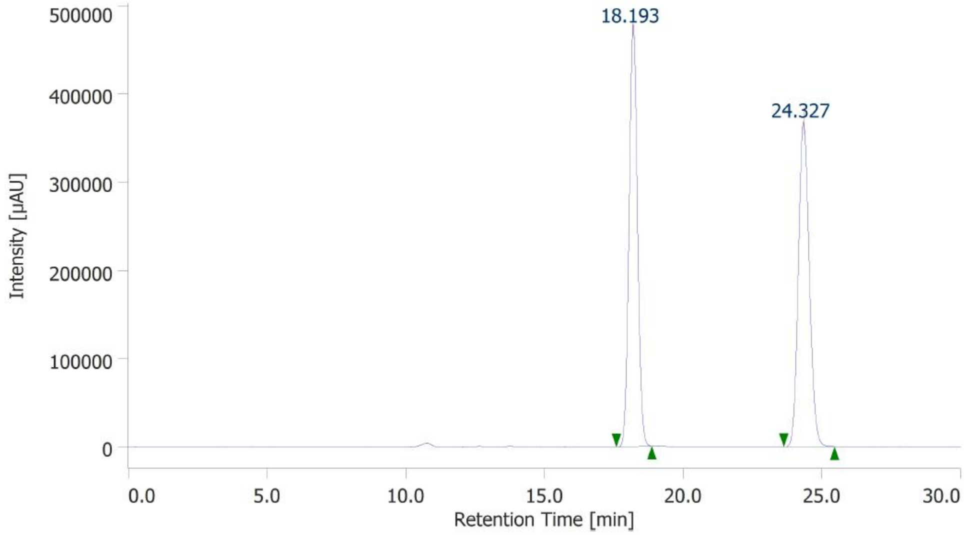

Peak Information

#	tR [min]	Area [µV·sec]	Area%
1	18.193	10049037	49.848
2	24.327	10110393	50.152

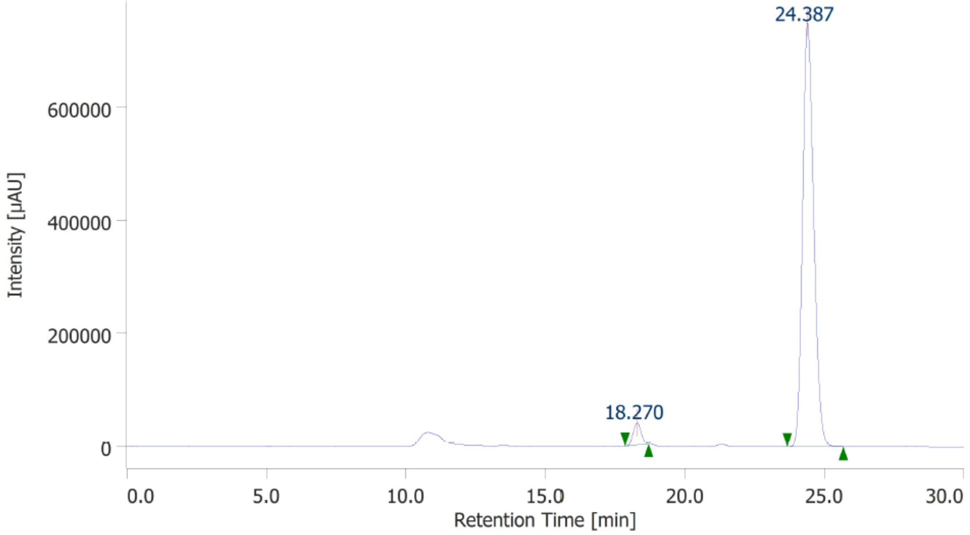

Peak Information

#	tR [min]	Area [µV·sec]	Area%
1	18.270	757525	3.519
2	24.387	20769707	96.481

(*rac*)-2-cyclohexylhexan-2-ol ((*rac*)-2m)

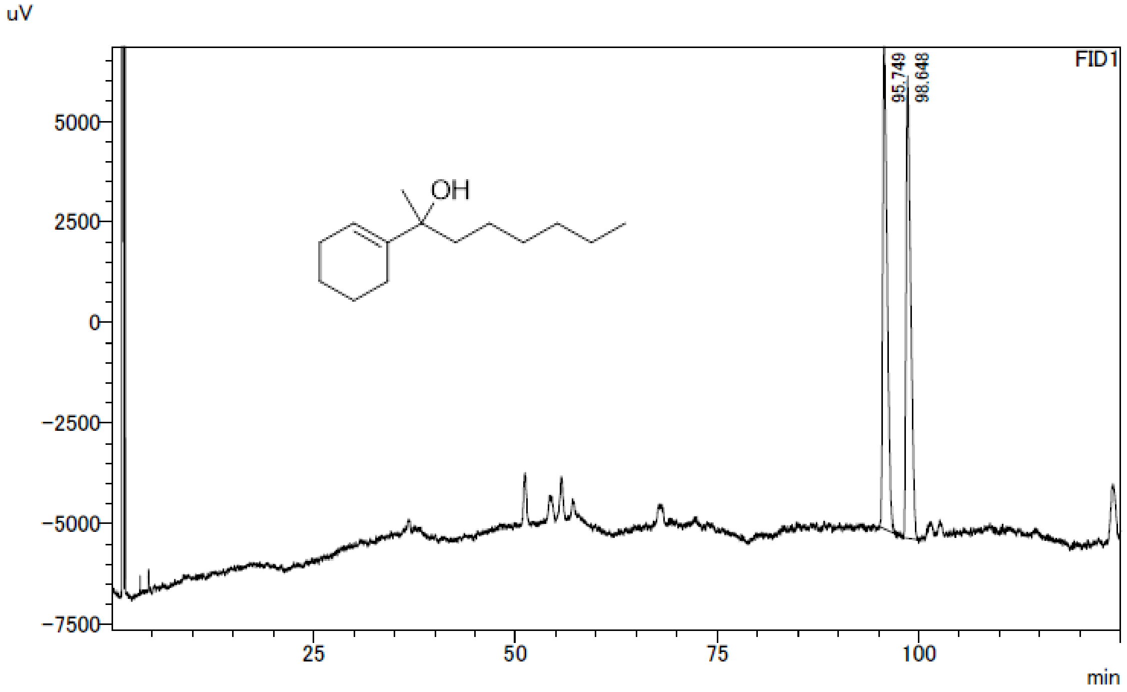

FID1

ピーク#	保持時間	Height%	面積	Area%
1	95.749	51.395	463519	50.159
2	98.648	48.605	460589	49.841
Total		100.000	924107	100.000

2-cyclohexylhexan-2- ol (2m)

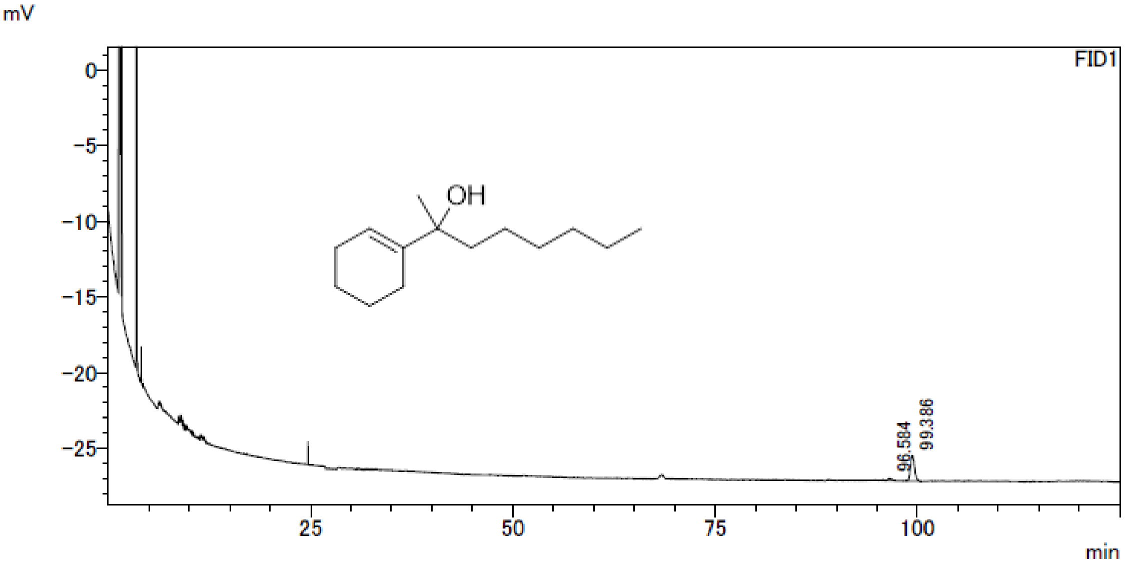

FID1

ピーク#	保持時間	面積	高さ	Area%	Height%
1	96.584	4329	164	6.858	8.807
2	99.386	58788	1693	93.142	91.193
Total		63116	1857	100.000	100.000

(*rac*)-2-cyclohexylhexan-2-ol ((*rac*)-2n)

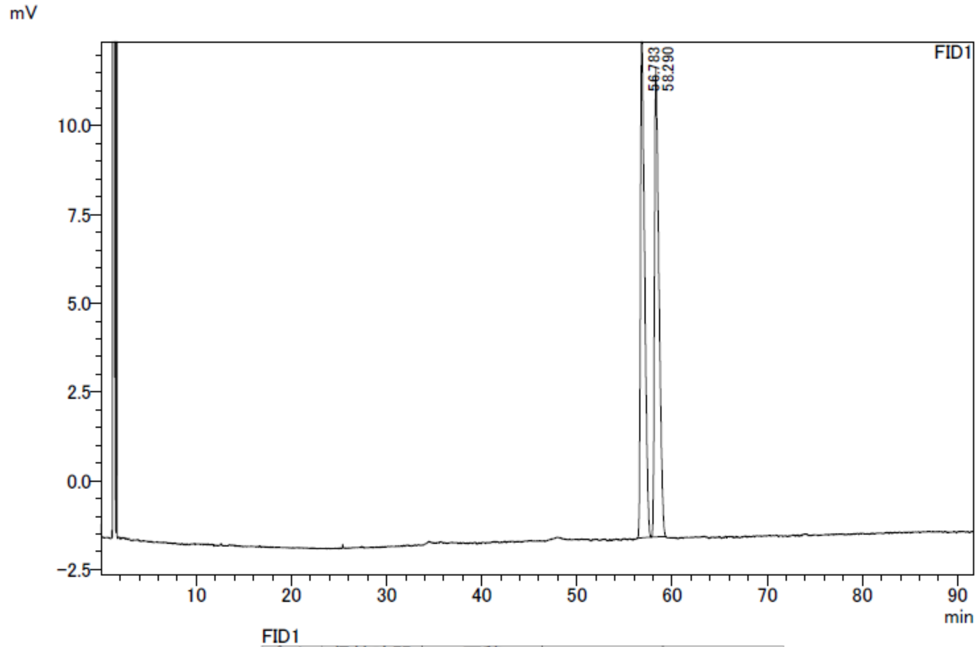

FID1

ピーク#	保持時間	面積	Area%	Height%
1	56.783	432665	50.107	52.322
2	58.290	430820	49.893	47.678
Total		863486	100.000	100.000

2-cyclohexylhexan-2-ol (2n)

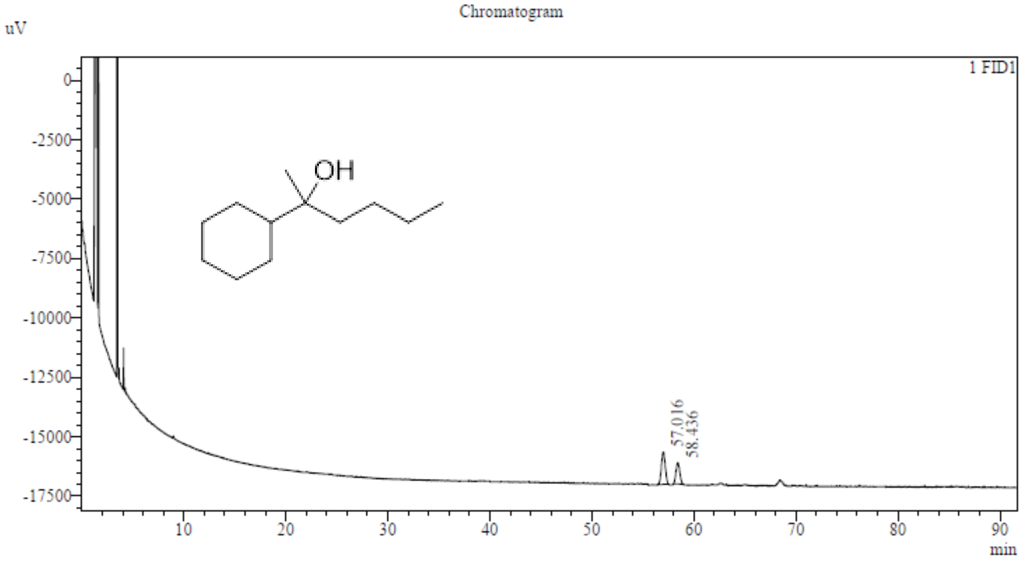

FID1

ピーク#	保持時間	面積	Area%	Height%
1	57.016	35602	60.886	60.512
2	58.436	22871	39.114	39.488
Total		58473	100.000	100.000

(*rac*)-(2-methoxyhexan-2-yl)benzene

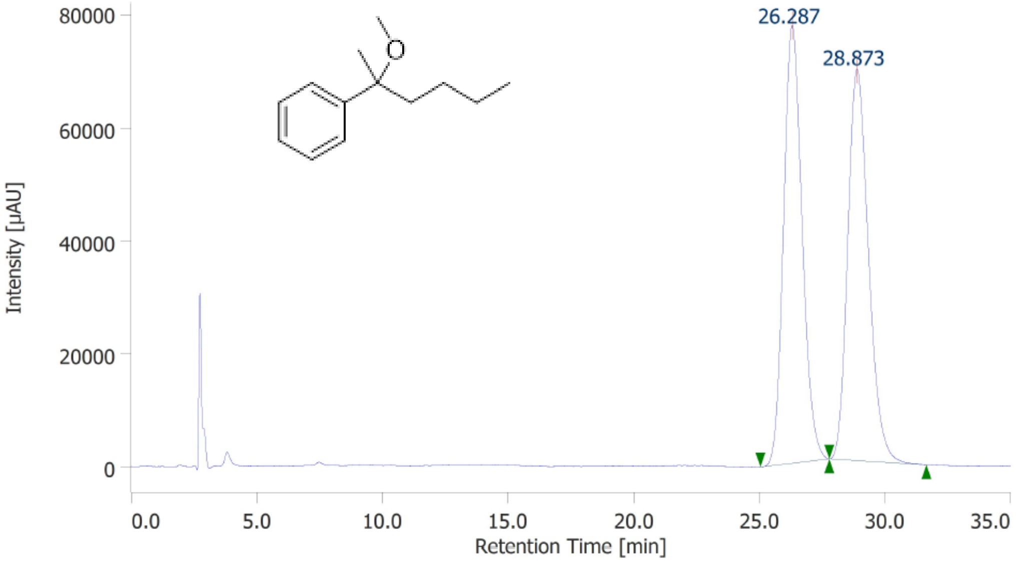

Peak Information

#	tR [min]	Area [µV·sec]	Area%
1	26.287	3985595	49.988
2	28.873	3987492	50.012

(2-methoxyhexan-2-yl)benzene

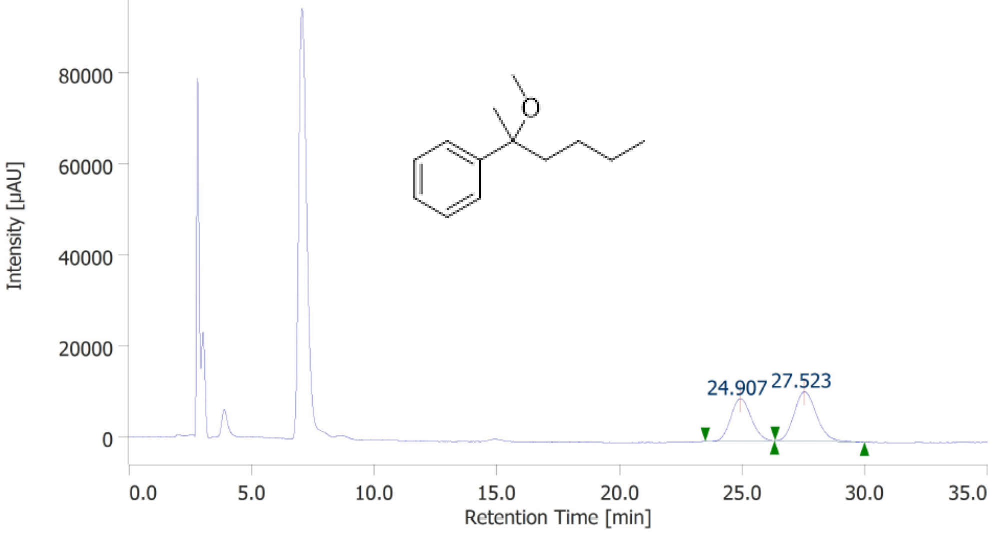

Peak Information

#	tR [min]	Area [µV·sec]	Area%
1	24.907	537078	43.798
2	27.523	689175	56.202

Single-crystal X-ray diffraction

Single-crystal X-ray diffraction (XRD) data were collected on a Rigaku XtaLAB Synergy Custom diffractometer equipped with a HyPix-Arc 150° X-ray detector, using graphite-monochromated Cu-Kα radiation. The structures were solved with the SHELXT structure solution program[18] using Intrinsic Phasing incorporated in the OLEX2 program package[19] and refined with the SHELXL package and olex2 refinement package using Levenberg-Marquardt minimization[20]. Hydrogen atoms were placed in calculated positions and refined using a riding model.

Crystallization of the IDPi–PFTB complex: The IDPi catalyst (ca. 5 mg) was first mixed with 3-methylpyridine in a chloroform/hexane (1:1, v/v, 1 mL) solution to generate the corresponding acid–base pair. A hexane solution of PFTB (2 mL) was then added dropwise. Upon addition, a microcrystalline precipitate formed rapidly.

Solvent disorder and occupancies: The solvent molecules exhibit partial occupancies in the crystal structure: chloroform was refined with an occupancy of 0.5, while PFTB and hexane were each refined with occupancies of 0.25.

Table S2. Crystal and structure refinement data

Identification code	IDPi_PFTB
CCDC NO.	2525386
Empirical formula	$C_{119}H_{92.15}Cl_{1.5}F_{8.25}N_4O_{8.25}P_2S_2$
Formula weight	2046.10
Temperature/K	100.15
Crystal system	trigonal
Space group	$P3_1$
a/Å	21.8600(3)
b/Å	21.8600(3)
c/Å	18.9832(3)
α/°	90
β/°	90
γ/°	120
Volume/Å^3	7856.0(2)
Z	3
ρ_{calc}g/cm^3	1.297
μ/mm^{-1}	1.723
F(000)	3186.0
Crystal size/mm^3	0.11 × 0.09 × 0.06
Radiation	CuKα (λ = 1.54184)
2Θ range for data collection/°	4.668 to 136.43
Index ranges	$-26 \le h \le 26, -26 \le k \le 26, -22 \le l \le 22$
Reflections collected	113594
Independent reflections	18976 [R_{int} = 0.0201, R_{sigma} = 0.0147]
Data/restraints/parameters	18976/662/1417
Goodness-of-fit on F^2	1.028

Final R indexes [I>=2σ (I)]	R_1 = 0.0820, wR_2 = 0.2204
Final R indexes [all data]	R_1 = 0.0834, wR_2 = 0.2229
Largest diff. peak/hole / e Å^{-3}	2.33/-0.56
Flack parameter	0.027(4)

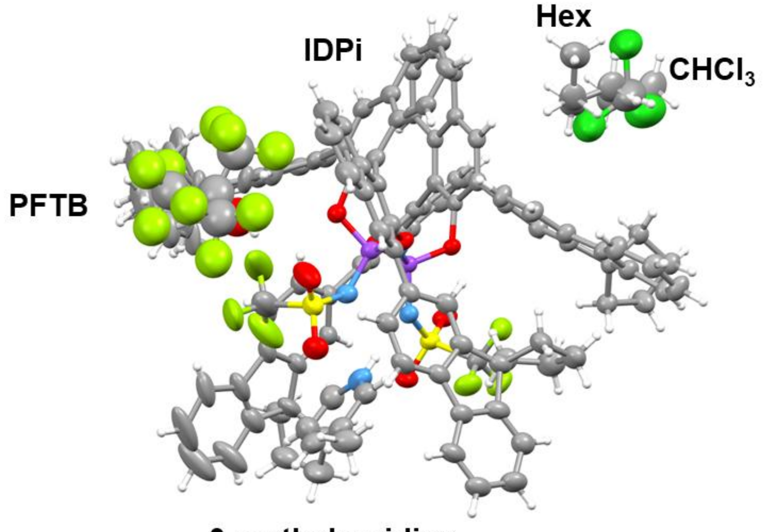

Figure S11. X-ray crystal structure of the IDPi–PFTB complex. Thermal ellipsoids are drawn at the 50% probability level.

FTIR spectroscopy:

Fourier-transform infrared (FTIR) spectra were recorded on a JASCO FT/IR-4X spectrometer. Measurements were performed on ground crystalline powder samples.

Figure S12 compares the FTIR spectra of PFTB, IDPi, IDPi with 3-methylpyridine, and the IDPi–PFTB complex. Neat PFTB exhibits a sharp O–H stretching vibration at high wavenumber, characteristic of a non- or very weakly hydrogen-bonded hydroxyl group. Upon formation of the IDPi–PFTB complex, this band becomes broadened and slightly shifted, while remaining in the high-frequency region (ca. 3600–3700 cm^{-1}), indicating the presence of an intermolecular hydrogen-bonding interaction. Consistent with this observation, single-crystal X-ray diffraction analysis reveals an intermolecular O–H···O=S hydrogen bond between PFTB and the sulfonyl oxygen atom of IDPi, with an H···O distance of approximately 2.1 Å. The persistence of a high O–H stretching frequency together with pronounced band broadening is attributed to the sterically hindered nature of the hydroxyl group and the constrained hydrogen-bond geometry in the solid state. In addition, the relatively low crystallographic occupancy of PFTB (0.25 per asymmetric unit) likely contributes to the reduced intensity of the corresponding O–H stretching band in the IR spectrum.

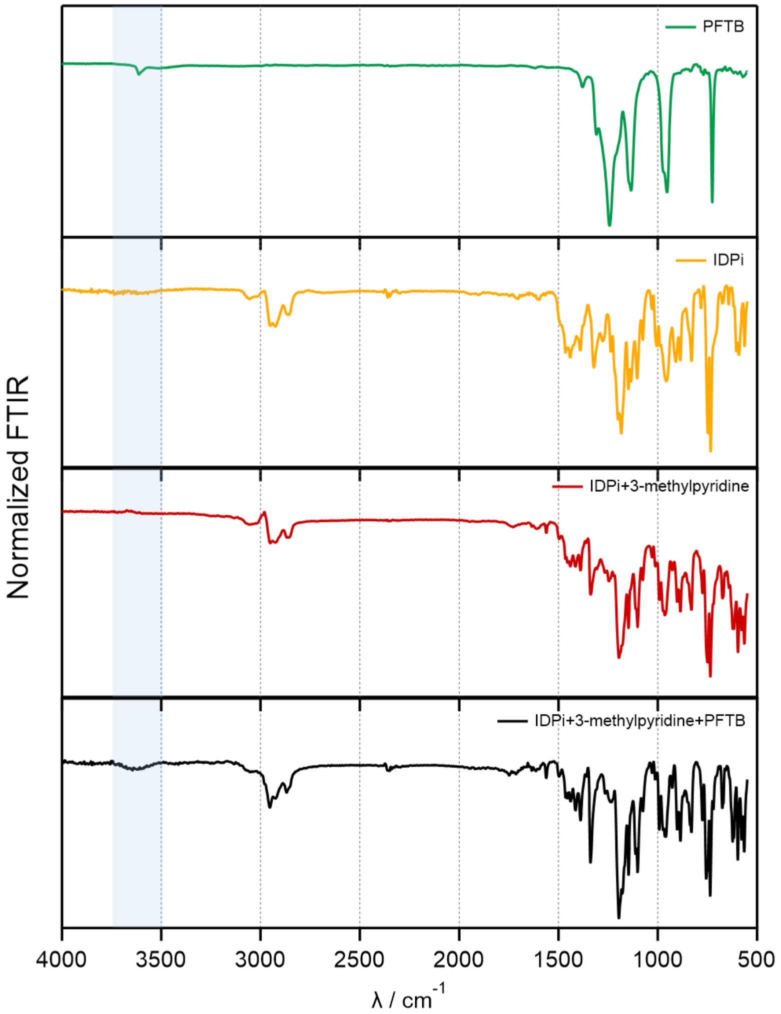

Figure S12. Solid-state FTIR spectra of PFTB, IDPi, IDPi with 3-methylpyridine, and IDPi–PFTB complex. The highlighted region shows the O–H stretching vibrations.

Computational studies

Method:

Preliminary mechanistic investigation was done using the NNP-AFIR method.[21-23] Then, all subsequent transition-state (TS) searches, optimizations, and energetics were performed using the protocol described below. Possible TS conformations were explored by the artificial force induced reaction (AFIR) method[24] implemented in the global route reaction mapping (GRRM) program.[25] An extensive conformational search was performed on possible catalyst-substrate orientations of reactant using AIMNet2 (model: aimnet2_wb97m_0.jpt)[26] via Orca 6.1.1 program[27, 28] using SC-AFIR for both enantiomers. The obtained approximately 1000 structures were sorted by energy. The energies of the lowest 100 structures were re-evaluated at g-xTB.[29] The lowest ten geometries were then further optimized at r^2SCAN-3c[30] level of theory, and the most stable conformers were identified. The reaction paths were subsequently generated from the corresponding conformers, and the transition state structures were optimized. All geometries were optimized at the r^2SCAN-3c level of theory using Orca 6.1.1 program. Thermal free energy corrections have been performed at the same level of theory using Orca 6.1.1 program and the temperature was set at 213.15K. Transition state structures were verified by the presence of a single imaginary vibrational frequency and the corresponding intrinsic reaction coordinates (IRC). Solvation effect has been accounted by using SMD(2-chlorobutane) solvation model[31] as implemented in Orca 6.1.1 program. All single point energy is calculated at SMD(2-chlorobutane)-ωB97M-V/def2-TZVPP level of theory.[32, 33] The visualizations of the molecular geometries were generated using the ChimeraX[34] version 1.9 followed by rendering with Blender version 4.3.[35] Conversion of enantiomeric ratio and ΔΔG was performed based on the Boltzmann distribution as follows: $\Delta\Delta G = RT\ln(\mathrm{pdt}(R)/\mathrm{pdt}(S))$.[36]

Results and discussion

The computational studies were performed using **1a**, **3d**, water, and two explicit molecules of PFTB (Figure S13). The protonation step is calculated with and without PFTB molecules (**TS0** and **TS0'**). As a result, the association of PFTB molecules lowers the barrier for protonation by 5.4 kcal/mol, which is in good agreement with experimental results. The subsequent addition of water to the carbocation intermediate seems to be the enantiodetermining step, which is discussed in the main text.

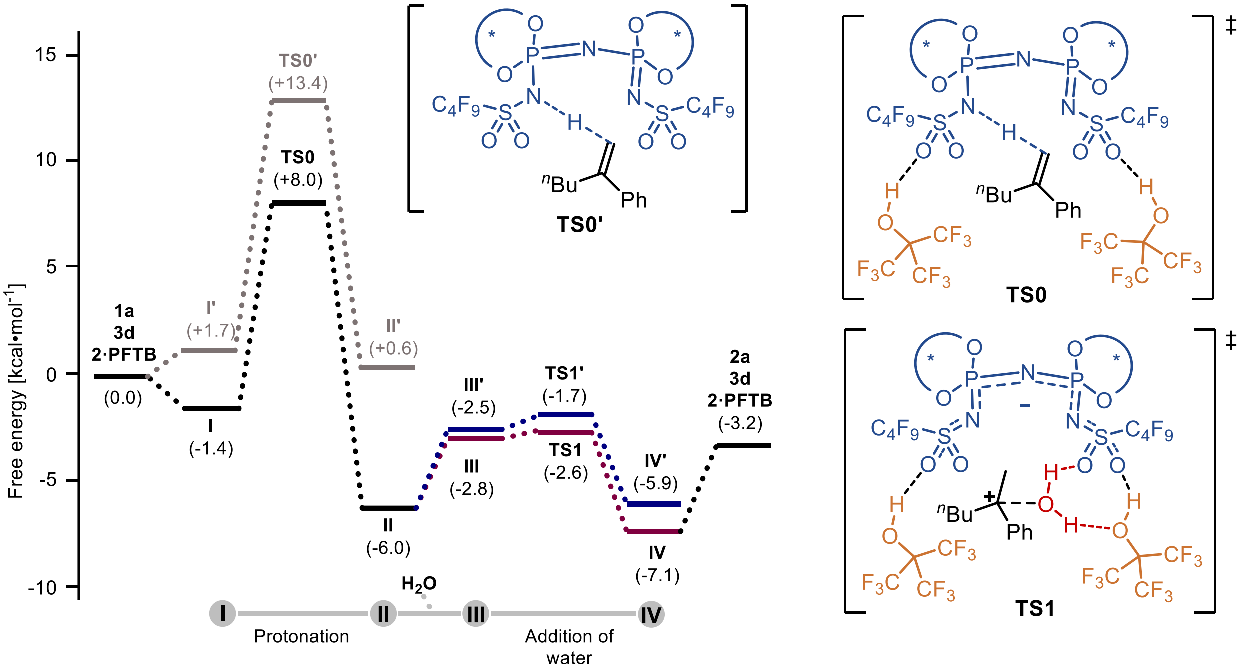

Figure S13. Energy diagram of the asymmetric hydration of alkene **1a** with **3d**. **TS0** represents the protonation transition state with two molecules of PFTB, whereas **TS0'** represents the one without them. The reaction pathway via **TS1** leading to the major enantiomer is depicted in red, and the one via **TS1'** leading to the minor enantiomer is depicted in blue.

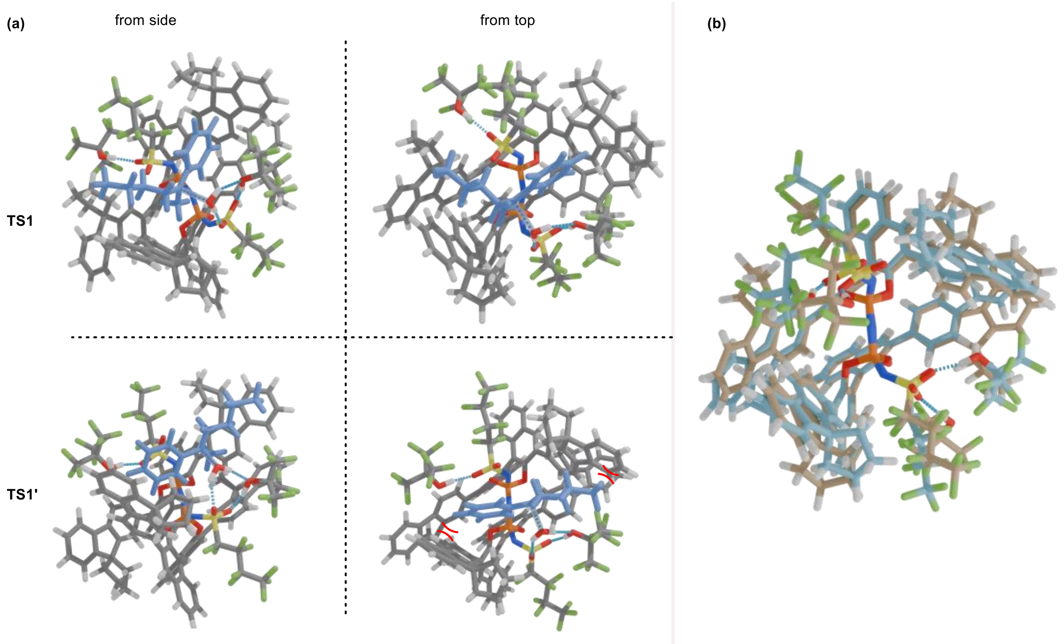

Figure S14. Visualization of transition states (a) Visualization of the transition states from side and top. Substrate is depicted in blue for clarity. Steric repulsions are depicted in red for **TS1'**from topview. (b) Overlaid image of counteanion of the transition states.

Further visualizations of the transition state structures are provided in Fig. S14. While the alkyl group can rotate to minimize the steric demands within the pocket in **TS1**, the substituents in **TS1'** have no analogus means of relief and instead induce deformation of the chiral counteranion.
To shed light on the origin of enantioselectivity, a distortion-interaction analysis was conducted following the Houk-Bickelhaupt protocol[37] (Table S3). Gas phase single point energies of **TS1** and **TS1'** were calculated at the ωB97M-V/def2-TZVPP level of theory. The energies were decomposed into the contributions from the catalyst fragment (chiral counteranion and PFTB) and the substrate fragment (carbocation and water). The results indicate that distortion of catalyst and substrate is the predominant factor controlling enantioselectivity, which is consistent with the structural features shown in Fig S14.

Table S3. Summary of the distortion-interaction analysis of **TS1** and **TS1'**

TS	ωB97M-V/def2-TZVPP (in Hartree)	Relative energy ΔΔE (kcal/mol)
**TS1**	-11309.96146	-1.87
**TS1'**	-11309.95849	
Substrate fragment		
subst- **TS1**	-543.6331972	-1.66
subst- **TS1'**	-543.6305547	
Catalyst fragment		
cat- **TS1**	-10766.14559	-2.51
cat- **TS1'**	-10766.14159	
Total distortion	(predominant factor)	-4.17
Total interaction		+2.30

Table S4. Energy table of the optimized structures at SMD(2-chlorobutane)-ωB97M-V/def2-TZVPP level of theory at 213.15K. Energies are given in Hartree. Computed single point energies (E), Gibbs free energy corrections (Gcorr), Gibbs free energies (G), and imaginary frequencies for transition states are provided.

Structures	E (solv) (in Hartree)	Gcorr (in Hartree)	G (solv) (in Hartree)	Frequency
**1a**	-466.8630771	0.22180372	-466.6412733	-
PFTB	-1127.10146	0.04089402	-1127.060566	-
H_2O	-76.43997343	0.00971292	-76.43026051	-
**3d**	-8512.487786	1.6385955	-8510.84919	-
**I**	-11233.61413	2.00028167	-11231.61385	-
**I'**	-8979.368567	1.88087031	-8977.487696	-
**TS0**	-11233.59432	1.99540284	-11231.59892	-1229.38
**TS0'**	-8979.345700	1.87654543	-8977.469155	-941.09
**II**	-11233.61992	1.99879272	-11231.62112	-
**II'**	-8979.370455	1.8809666	-8977.489489	-
**III**	-11310.06905	2.02276549	-11308.04628	-
**III'**	-11310.06624	2.02041109	-11308.04583	-
**TS1**	-11310.06881	2.02288426	-11308.04593	-19.81
**TS1'**	-11310.06658	2.02201983	-11308.04456	-68.38
**IV**	-11310.07934	2.02624259	-11308.0531	-
**IV'**	-11310.07411	2.02291811	-11308.05119	-
**2a**	-543.3246754	0.248069	-543.0766064	-

Table S5. Energy table of the optimized structures at the SMD(Chloroform)-ωB97M-V/def2-TZVPP/r^{2}SCAN-3c level of theory. Energies are given in Hartree. Computed single point energies (E), Gibbs free energy corrections (Gcorr), Gibbs free energies (G), and imaginary frequencies for transition states are provided.

Structures	E (solv) (in Hartree)	Gcorr (in Hartree)	G (solv) (in Hartree)
**1a** (298.15 K)	-466.8625054	0.20882823	-466.6536771
**1a** (213.15 K)	-466.8625054	0.22180372	-466.6407016
**water** (298.15 K)	-76.44082026	0.00377584	-76.43704442
**water** (213.15 K)	-76.44082026	0.00971292	-76.43110734
**2a** (298.15 K)	-543.3249327	0.23430794	-543.0906248
**2a** (213.15 K)	-543.3249327	0.248069	-543.0768637

Cartesian coordinates of the optimized structures

1a

C	1.80916447751675	1.92112447406783	-1.10858511736166
C	2.93545899656771	2.30022884524031	-0.38859342571415
C	3.09811878673874	1.91913740167586	0.94995513916289
C	2.10424043558716	1.12270711456064	1.53462253845638
C	0.97566780436389	0.74698845517972	0.81605168882089
C	0.82179355909884	1.14473698213629	-0.50879777636929
H	1.70770503694829	2.22318015302828	-2.14720807308543
H	3.71679576019003	2.88124990833803	-0.87057319262844
H	2.20388059254958	0.80920369180697	2.56972043698740
H	0.21191945801799	0.13984470224102	1.29387736713136
H	-0.05700707973674	0.84459024440602	-1.07196551549070
C	4.29379605143281	2.34263915348809	1.71733727902321
C	4.80814516007088	3.56886174718032	1.56919389981059
H	4.34261768135064	4.30856886204353	0.92464335578507
H	5.69898475991682	3.87150162200649	2.11176787211211
C	4.91554534546844	1.33608858568505	2.65601378771610
H	5.78050055105104	1.79957172464209	3.14553789355969
H	4.20424346663358	1.08876538990200	3.45623751413472
C	5.35064198888600	0.03957843090167	1.95571862239887
H	4.47029372976077	-0.46169962915999	1.53127653355505
H	5.76664452666277	-0.64392797032675	2.70852443975646
C	6.37983539159391	0.27049636830557	0.85142063703173
H	5.95078149450243	0.93623006952580	0.09108172913872
H	7.24598810538507	0.80366062531625	1.26860129095866
C	6.83579026441174	-1.03169632940632	0.19756920401458
H	5.98644607370895	-1.56329499653598	-0.24627974453338
H	7.56680200326837	-0.84767743964703	-0.59625576148247
H	7.29939774800051	-1.70134974662877	0.93107097710701

PFTB

C	0.27797958692282	-0.92632337866945	0.00709551491763
C	0.78126009293167	-0.16695383390050	1.28349062727717
C	-1.28903042573428	-0.96541606078757	-0.01721181861745
C	0.85781511075993	-2.37692616124938	-0.02361188456346
O	0.75981713400097	-0.30040056887168	-1.14063794908756
H	0.46759544185383	0.62184782480536	-1.14515935395423
F	0.73666883832871	-2.95822869790128	1.18263252203608
F	0.20197749983601	-3.13010413425051	-0.92040691782327
F	2.14947277503269	-2.36427830032746	-0.36287280357684
F	2.07692967964147	-0.41812899208303	1.51271248713074
F	0.08338354063918	-0.48924129034902	2.38001545289734
F	0.65792654369323	1.16410374427148	1.08338746006855
F	-1.77176201584888	0.26922632376427	0.25249264166181
F	-1.79522298725247	-1.82184526918041	0.87941656060866

F	-1.73112833480490	-1.30766289527081	-1.23186995897516

H_2O

O	-1.43972364254517	-0.63892632702788	0.00000000000000
H	-0.47904523182409	-0.59319055530109	0.00000000000000
H	-1.71730430563073	0.28192906232897	0.00000000000000

3d

O	-3.75703409092896	-1.55570239600690	-0.85418211967351
O	-1.80621784515262	-3.19481854951271	-0.81913979983875
O	-0.67514140967005	-2.40260763850137	3.11204324946928
O	-1.87865509355966	-0.13383223761898	3.18961096171293
P	-0.99295626254915	-1.07701550678609	2.23010088278415
P	-2.21825856666906	-1.69024597605428	-0.44801287448900
S	1.41065259817110	-0.58803817012587	0.76370176272260
S	-2.08317905993804	0.59075936403384	-2.51296942249778
O	0.84519208033902	-1.37218817715084	-0.36733603211931
O	2.15874814645137	0.62446974600365	0.43732872223066
O	-3.33218970382888	1.09203253599129	-1.95722881209070
O	-0.92870814475617	1.42317660622903	-2.82259275364300
N	-1.48629044567657	-0.68915666861079	-1.59608741180535
N	0.39579853031167	-0.33230836589278	1.96684398863704
N	-1.98772013436263	-1.42468886282233	1.05078676511374
H	-0.45708386085579	-0.69507086405655	-1.51870965190079
C	2.68358295160346	-1.80837377511229	1.48293474211910
F	2.08269053602258	-3.01277545755508	1.57117069056112
F	3.69235701161705	-1.87649265649574	0.59131144875834
C	3.25207015206350	-1.41940386266883	2.88470214611714
F	2.36660281459966	-1.80122323733227	3.83533515700778
F	3.42087452230293	-0.08356457240624	2.95315404533086
C	4.63180733336224	-2.09767097971230	3.19176447612727
F	4.60703928952940	-3.38628938510607	2.76852821516538
F	5.60517180707923	-1.43785458197971	2.53352968700162
C	5.01400641882555	-2.11694778175733	4.70746318632881
F	4.25417218944863	-2.98398658635698	5.38705145584012
F	6.29519722400993	-2.49698080300926	4.82555493319761
F	4.87506119743458	-0.89750410141032	5.24307764085421
C	-2.57834979182471	-0.30682253117080	-4.13225962925549
F	-1.45098143453196	-0.77251292784402	-4.70399648423245
F	-3.37631361348499	-1.33094633267336	-3.80492943580376
C	-3.32422787784544	0.64025675049232	-5.12326513375531
F	-2.68747308953514	1.83004900878815	-5.15583655315377
F	-4.58349915517024	0.82322666537284	-4.67239150403348
C	-3.38487212349177	0.06785723774303	-6.58155711010029
F	-2.18361575240658	0.24727441298246	-7.16930731557304
F	-3.65952191409056	-1.25673465403156	-6.53216480726464

C	-4.46634622045084	0.74113718260299	-7.48754430440065
F	-4.36239950979835	2.07537363558646	-7.42322878724872
F	-4.27031260300360	0.35193035943321	-8.75453496695580
F	-5.69726651120844	0.37704559877409	-7.11616971895123
C	-2.24320716908504	-3.67061387617813	-2.07005231127721
C	-4.57362312339926	-2.60890607836325	-0.39246244949349
C	-1.26260379780533	-3.83469713538194	-3.07683825089246
C	-5.34859062223547	-2.39728459040200	0.77486356701502
C	-5.81467596052742	-4.64063271033816	-5.16839327616889
C	-5.41427275328235	-4.37655302135129	-3.88079627419781
C	-4.04153410531011	-4.24262576700520	-3.56295166054106
C	-3.07944354899832	-4.35683680044721	-4.61624077362615
C	-3.52729585135225	-4.64476539921899	-5.92786500479903
C	-4.86500370630510	-4.78587571505969	-6.20030293593775
H	-6.87293285020987	-4.73015471149828	-5.39494605865481
H	-6.15308778253941	-4.25318760131610	-3.09564506255584
C	-3.58914359177374	-3.91831919994958	-2.24837557417515
C	-1.70687865874829	-4.17464867322576	-4.33534113493503
H	-2.78905305631051	-4.73655984393330	-6.72039195575809
H	-0.97865797437648	-4.31305166962586	-5.13102356619446
C	-6.72952157542416	-7.05991253620773	0.46082537389670
C	-6.81039831536051	-5.82680087772193	1.05703331930599
C	-6.07650088343209	-4.72502054731078	0.55168819716083
C	-5.27036521898782	-4.90514126172239	-0.61603854969640
C	-5.19506136771280	-6.19414641958840	-1.19667657433301
C	-5.90646420283555	-7.24487730802805	-0.66939818834153
H	-6.73310671772527	-3.35819643447870	2.08021734210547
H	-7.43059627157577	-5.67668672528566	1.93719879599121
C	-6.10272532895329	-3.47459348732023	1.20257802410301
C	-4.51597979714282	-3.79297404172178	-1.09706536302482
H	-4.56264318956705	-6.34780897126035	-2.06461619636965
H	-5.83141398807332	-8.22782544640810	-1.12479254577118
H	-5.19632687296479	-4.99700346910198	-7.21264442298931
H	-7.29050513390133	-7.89852754853629	0.86233554861080
C	-1.76573864667788	-2.99311859123460	3.76466135511741
C	-1.53339737361954	0.00051463437131	4.53683626021723
C	-1.21185951348698	1.29262880399632	5.02310611324723
C	-2.26572224721117	-4.22628291061925	3.27848544210870
C	-5.39352819129307	-2.63151697762380	6.94197912719228
C	-4.19983789816427	-2.15010063300293	6.45968206515542
C	-3.50402910554731	-2.83287247468256	5.43278842988115
C	-4.08228173157880	-4.02467611316200	4.89639729717472
C	-5.30562046872822	-4.50162644601307	5.42665604728285
C	-5.95059763799065	-3.82077412829151	6.42859954868622
H	-5.91594338455645	-2.08697154845246	7.72309056735928
H	-3.78796661722124	-1.22860238010613	6.85701757542878

C	-2.29494816848421	-2.33570472512958	4.85326411724039
C	-3.42442203745601	-4.70607483965086	3.85182998107673
H	-5.72820982695428	-5.41553302556599	5.01615836327920
H	-3.82957620736600	-5.65255686376708	3.50165248139627
C	-0.29282024040710	-0.72671530287547	9.35737795257434
C	-0.33220022909604	0.37474416603601	8.53945606630669
C	-0.78350234713013	0.26788761097784	7.20169725680577
C	-1.21428839695478	-1.00242963139593	6.71222918063553
C	-1.15284899445276	-2.12101649275552	7.57734807865504
C	-0.70016799115351	-1.98498337593149	8.86760166711236
H	-0.58942494724101	2.36954038418265	6.74780686993378
H	-0.00984552923240	1.34675292902543	8.90393079077366
H	-1.45569352828321	-3.09484931192435	7.20613407126698
H	-0.64836910416863	-2.85581041173394	9.51447618377191
C	-0.84540813791936	1.39074605612038	6.35008138899149
C	-1.66093853151131	-1.09969930610834	5.36019637683770
H	-6.89207386306162	-4.19058966891247	6.82368021367306
H	0.06041420804966	-0.63483855992285	10.38016160168955
C	-5.35157200600650	-1.13074609697764	1.53599022785767
C	-5.38606750214074	0.12497355204695	0.90654603554156
C	-5.37927584044806	-1.19411710180800	2.93935530299734
C	-5.48056778999406	-0.04819686876415	3.71494709154846
C	-5.56086137933738	1.18778776422827	3.07949384830480
C	-5.49390223944222	1.26831557040648	1.67912172198798
C	-5.75623118978874	2.53192741119812	3.61095361920564
C	-5.79783092180313	3.43791519312847	2.53373352041269
C	-5.58090176883021	2.70786392984181	1.21976029279799
C	-5.92679539792191	2.96775461439437	4.92240341759480
C	-6.14993503979285	4.32160977228396	5.15514616515695
C	-6.19020104913652	5.22279612657773	4.09102223173769
C	-6.00706949067325	4.78813039200755	2.77629487891450
C	-6.67165482672389	2.97217403521790	0.15533667798198
C	-6.26001551378913	4.27792400877379	-0.54957201226707
C	-4.72749676722159	4.41222027487769	-0.34120096538071
C	-4.29904288828750	3.19124110960471	0.49220347567488
H	-5.35792271753389	0.19466029320399	-0.17675738634393
H	-5.30449570233835	-2.16050203667355	3.42811997263786
H	-5.50181430222417	-0.13261219620796	4.79812789623952
H	-5.89599834573719	2.26412030661069	5.75067612405399
H	-6.29616501326489	4.67979629548053	6.17046944519328
H	-6.02898526080588	5.50988346327681	1.96618165460406
H	-6.65087794651533	2.14113796930756	-0.55973526382618
H	-7.67437322244870	3.00762089689509	0.59388448827528
H	-6.78767880698803	5.13494557892654	-0.11769789084315
H	-6.53152942448791	4.25142853288485	-1.60981635064283
H	-4.18632624721117	4.43860298898204	-1.29252220335582

H	-4.48316185406335	5.34366664359656	0.17872607940582
H	-3.49847442020897	3.41193428261396	1.20291188029823
H	-3.95450837289100	2.38617751543558	-0.16033924031372
H	-6.36807042382890	6.27698007184093	4.28683054177587
C	0.17657209805100	-3.70637479375929	-2.75763129336542
C	0.99752259180840	-2.78639505558519	-3.42363416475666
C	0.72004643349752	-4.56444456189562	-1.79592830944630
C	2.07656002483051	-4.54922722948022	-1.51378619048442
C	2.88556943898996	-3.62392292739695	-2.16474599602678
C	2.34534353959781	-2.72308567454666	-3.10094923200114
C	4.31927728454506	-3.38534638280558	-2.05625353529794
C	4.65702834198944	-2.32567130895957	-2.91203262368580
C	3.42661578773643	-1.78161226515405	-3.60684453411780
C	5.28754092755787	-4.01124955478701	-1.27522673148695
C	6.60352247636910	-3.56802253539118	-1.36321172043016
C	6.94298679146609	-2.51719285072451	-2.21746541025918
C	5.96963019243203	-1.88786579658458	-2.99495098497544
C	3.14260666127204	-0.30272473537758	-3.22932219469460
C	2.16227481431925	0.19631443605196	-4.29900773952582
C	2.46713289128012	-0.64151218366890	-5.57059343416354
C	3.51577579117822	-1.69543006252021	-5.14787916703741
H	0.55851677199508	-2.12998195207923	-4.16712720602247
H	0.07026606133754	-5.26909221507108	-1.28972838983664
H	2.48985601243974	-5.25962611706619	-0.80305618701628
H	5.02224805251467	-4.82553811526416	-0.60507730422688
H	7.37441722474359	-4.04093563881372	-0.76156614803660
H	6.24600095805863	-1.06423935973335	-3.64936103068785
H	2.77074922036877	-0.19766898403007	-2.20575670981549
H	4.09055371761820	0.24573726900872	-3.29631324983580
H	2.26793121345052	1.27203422907499	-4.47045398496824
H	1.13043085778575	0.04131617495087	-3.97004164955696
H	1.55769932958760	-1.12136615830771	-5.94861293795043
H	2.84893529741422	-0.01966959614378	-6.38646808769256
H	4.52240719159805	-1.35063010932756	-5.41226974668004
H	3.36880221827560	-2.67137314241754	-5.62130026510698
H	7.97509684634132	-2.18349264217950	-2.27383039724317
C	-1.55966368597463	-5.05747196076200	2.28190755477672
C	-0.22937736959680	-5.44597359200308	2.51299752054432
C	-2.26837775804928	-5.60032560278605	1.20557881846146
C	-1.66796660519756	-6.50301105366473	0.33798311535768
C	-0.35605888038958	-6.89776746359729	0.58228107003238
C	0.35527719628100	-6.39395455126322	1.68979417612249
C	0.51956081570267	-7.79221419288294	-0.16742105577440
C	1.74666298397383	-7.88044314557427	0.51030318124238
C	1.70455808249977	-7.09063879608548	1.80047345698574
C	0.31958346527118	-8.46385378987258	-1.37093499265599

C	1.36110441696954	-9.22680143135086	-1.89071106594120
C	2.57910635509550	-9.31940197914625	-1.21605602496238
C	2.77667972675428	-8.64976318315138	-0.00736558638048
C	1.74283413471727	-8.02726419272591	3.04319891311457
C	2.09390641875313	-7.10768923961865	4.22014205445813
C	2.92933093266667	-5.95111165524466	3.60848501333102
C	2.91338222667127	-6.17516333385981	2.07996164744702
H	0.30389084343205	-5.02390144686942	3.35875702960896
H	-3.29634215769568	-5.29149040679411	1.04195544838465
H	-2.22379584733505	-6.89074249744683	-0.51225131318834
H	-0.62478521260315	-8.38633677627137	-1.90359875324037
H	1.22846689476930	-9.75061694917859	-2.83281221844663
H	3.73023856064941	-8.73040085807656	0.50936095992828
H	0.80434645199159	-8.57552215617652	3.17332579906276
H	2.54086367946750	-8.76316244712334	2.88195299298248
H	2.63901398492091	-7.64764433258615	5.00064867018495
H	1.18041929028024	-6.72143713734614	4.68464789012043
H	2.49719324802606	-4.97826181866018	3.86431753093959
H	3.95457343642895	-5.94045273295116	3.99036560937087
H	3.82153273214355	-6.70662298111363	1.76974328544984
H	2.86935551861856	-5.24380778384903	1.51321032113272
H	3.38312850684189	-9.91464519115413	-1.63888406792661
C	-1.43802996434214	2.52616356514854	4.23515429210810
C	-0.82218398992990	2.79292584719867	3.00283664740758
C	-2.33426082122944	3.45674657820608	4.78300089635156
C	-2.65195961813408	4.63174850054978	4.11726617586072
C	-2.05290819821793	4.88128585876200	2.88973635830427
C	-1.12408239706869	3.97490644479261	2.34520764880195
C	-2.26381769318701	5.96354816264626	1.93573636787087
C	-1.45438996675550	5.72334679848505	0.81364210012039
C	-0.58083906474568	4.50429728049057	1.02922940492185
C	-3.11841045054508	7.06286333406063	1.97729094017257
C	-3.16494227862843	7.91434662359613	0.87642457407770
C	-2.36980155679728	7.66921793850586	-0.24480308955583
C	-1.50346007522073	6.57507678467556	-0.27823183995365
C	-0.57519376381142	3.46272566624591	-0.13687530272319
C	0.84425174444663	3.48231497050818	-0.72479266595291
C	1.71913924193245	3.82557141470569	0.48159122232139
C	0.92589450586636	4.93565706008416	1.16602817117976
H	-0.11958686324882	2.07780504601421	2.58325349394650
H	-2.82446007124635	3.22291310853627	5.72379255179337
H	-3.38831587062284	5.31148093939136	4.53492425207719
H	-3.74438570471357	7.24745425943347	2.84666204969352
H	-3.82880021103474	8.77422676360102	0.88626139387580
H	-0.88353742741752	6.39474544135042	-1.15392582372558
H	-0.79399953141100	2.46778773540210	0.27115173201480

H	-1.35088076669380	3.68391521946869	-0.87626598800359
H	0.92899595934537	4.27320304003315	-1.48272424969381
H	1.11355486970332	2.53418192536315	-1.19758118138826
H	1.80692079165811	2.94940965663665	1.13295232644212
H	2.73320623184224	4.13784611840543	0.20923989901796
H	1.06802325539431	5.87892562659825	0.62510757649816
H	1.19925239793964	5.10703284521489	2.21165706070496
H	-2.42534315125376	8.33868560969467	-1.09848975369777

I

H	4.30966207597030	1.00277793011088	0.64823479250509
O	4.86589341327653	1.17942800915191	-0.14496258540376
C	6.12904577552675	1.61020902706998	0.21380961195866
C	6.07191590383500	2.69045598796681	1.35052500025745
F	5.90880749718444	2.10315753470483	2.55021745703945
F	7.19466167495174	3.43172599827399	1.40342764944960
F	5.03551759281126	3.51404054037089	1.15096845359848
C	6.73953253372069	2.24321374122578	-1.07791409698148
F	8.07328081232161	2.38780960903270	-0.97719294929845
F	6.48190499753602	1.48356734266309	-2.14877303077129
F	6.20340878797162	3.45800608906699	-1.30543334608911
C	7.00311982192387	0.39526153027936	0.68392343838640
F	6.28516886887078	-0.39429164775451	1.49680933903626
F	7.38397249070471	-0.35489411086556	-0.37023127506144
F	8.11346713044873	0.77872924439060	1.33777108904948
H	-1.80616142181357	3.33950604149875	-3.85284748079161
O	-2.44185300350053	3.99788760512231	-4.18947821542381
C	-1.82652782334846	4.89386111548021	-5.04951392076648
C	-0.77061770581998	5.75979314296367	-4.27624876883284
F	-0.39653577394647	6.84730385924337	-4.97151844837169
F	-1.26431978227126	6.16137871297895	-3.10024214023776
F	0.32625407058033	5.01821484301142	-4.02634418764502
C	-2.97389573661364	5.80232721244883	-5.59710997017529
F	-2.56839806944488	6.49730683537559	-6.67479530950389
F	-4.03603580984707	5.06700799807762	-5.94537025132476
F	-3.37438035419150	6.67863286138750	-4.66068390912914
C	-1.13388571890305	4.12820912171445	-6.23038886205997
F	-0.46422185769506	3.06454708770660	-5.74314621621291
F	-2.04440048148960	3.66264136482728	-7.10178722355300
F	-0.26414989000927	4.89649830069272	-6.90318632789756
C	2.02151487182872	1.66358069650264	-1.66508710287760
H	1.93883887899505	-0.39528341434732	-1.16121106470939
H	1.07201799222977	0.76865523193998	0.00098618178961
C	1.60870892490751	0.61476752898946	-0.93066577931874
C	1.65765280521872	3.04577828958256	-1.28202582358879
C	0.45520490731912	3.31985415245375	-0.61219294279998

C	2.52071348249714	4.11761319636196	-1.54865612209027
C	2.20289396857618	5.40888773169035	-1.14260112909955
C	1.00899584149963	5.66318922653137	-0.47695356171566
C	0.13663618958734	4.60986023713501	-0.21499325356845
H	-0.24511919408723	2.51410832576790	-0.40945536805780
H	3.46375552010853	3.93913509529789	-2.05552860706612
H	2.89460299878203	6.22087574694128	-1.34929332620820
H	0.75429455306815	6.66981251875624	-0.15921655549976
H	-0.79791538486273	4.80045492236916	0.30278460275323
C	2.92047612273009	1.45251597277822	-2.85242620861886
H	3.87899106045109	1.95134753057317	-2.65499811849554
H	3.15790558226794	0.38522486214607	-2.93298959142593
C	2.35178145822304	1.95995126352525	-4.18090028510462
H	1.99413463902402	2.99103833545079	-4.06912552401260
H	1.46933228427553	1.36009330734999	-4.44775587767890
C	3.38576970734838	1.89794544673647	-5.30328271747710
H	4.23081749040288	2.55144560406446	-5.04442012659802
H	3.80033416186836	0.88202175093142	-5.36349844661405
C	2.81343359968778	2.31132661232924	-6.65638815407288
H	2.42759414277829	3.33671524148842	-6.62317819909387
H	1.98203010821279	1.65888148103998	-6.94952350373473
H	3.57171174389340	2.26357381338227	-7.44527508473543
O	-3.21515652392034	-1.31144674571910	-0.45450110226517
O	-1.19073084557024	-2.82170260901405	-0.48328016063715
O	-0.23946101417307	-2.17294698928187	3.10963976282534
O	-1.59229262517890	0.01424413536305	3.29686646334977
P	-0.42686910602969	-0.70187856416108	2.45652189305122
P	-1.63338324669507	-1.29976390548536	-0.28728277784602
S	2.38544779058481	-0.36043592409204	2.28819816707917
S	-2.07169554179675	0.54463578786251	-2.72436316044662
O	2.43778995238743	-1.44095274658119	1.28702386736064
O	3.24392967472623	0.82561870708204	2.08983165393932
O	-3.46794604443633	0.68599229106459	-2.34042710404850
O	-1.21012708352285	1.68842332128560	-3.03277938107161
N	-1.17685578225480	-0.37546085088740	-1.63105347850518
N	0.92477772618959	0.11245318658244	2.70909936078729
N	-0.98634738406203	-0.70858856521231	0.97312595960162
H	-0.20874438514522	0.01519413773064	-1.56006527756345
C	3.14041522661633	-1.20065027856387	3.84874799673373
F	2.71388086714274	-2.48083286035437	3.87242507497833
F	4.47871278189459	-1.18942762960990	3.67083839159426
C	2.79726019091548	-0.54114335714416	5.21307100035295
F	1.53902599184600	-0.89957608768468	5.55554578948512
F	2.85923581584467	0.80334296781664	5.09274343594977
C	3.76297879295370	-0.96573855312395	6.37326952205027
F	4.05357633194960	-2.28436082347403	6.26429056237356

F	4.90456581345827	-0.25453134468239	6.27938737515453
C	3.17097735798907	-0.73912154546217	7.80225470179696
F	2.21158301778789	-1.62931127030845	8.06778585592166
F	4.14914350206635	-0.88537064103377	8.70784626887174
F	2.66861366601880	0.50072582717337	7.91055400842653
C	-2.05205107237626	-0.58432173932310	-4.26968437469478
F	-0.78484905597582	-0.62769765814552	-4.71344119938681
F	-2.44311959761714	-1.80575594996142	-3.88659068306300
C	-3.01265492284372	-0.04957845515482	-5.38178298747042
F	-2.92598141944744	1.29633091961819	-5.42268895347175
F	-4.27339509510852	-0.40166020004666	-5.05713707202298
C	-2.67149109342678	-0.61611345737912	-6.79989795291779
F	-1.56386342053940	-0.00086385919950	-7.26082115708378
F	-2.43059849164397	-1.94633085721934	-6.69137248124923
C	-3.80291781110422	-0.42308467647157	-7.86096978232148
F	-4.19608158091410	0.85561575044406	-7.89136158657379
F	-3.32298880128413	-0.76125247868600	-9.06483894397098
F	-4.85395791415344	-1.20221074899085	-7.58669841854690
C	-1.73140892777506	-3.56984882128193	-1.54275961006197
C	-3.98015835059681	-2.30846856763247	0.18169923712744
C	-0.83958570760317	-3.95827195759156	-2.57566311052050
C	-4.81225659960618	-1.90597716216752	1.25847271899823
C	-5.50688027506776	-5.44011323803627	-3.90599008595493
C	-5.02102901608865	-4.76549750837148	-2.81202685204043
C	-3.62903660975143	-4.57987758436563	-2.63537719640830
C	-2.74453169882083	-5.05962275291070	-3.64792323899152
C	-3.27555109223336	-5.77880340895504	-4.74752329109041
C	-4.62795924436272	-5.97076226061868	-4.87281057360200
H	-6.57891948212696	-5.55994870883394	-4.03011121431694
H	-5.70913695892890	-4.35870404344182	-2.07911502311778
C	-3.07172192011830	-3.90557393743519	-1.50220186682728
C	-1.37017567172259	-4.76458825900901	-3.56625963665384
H	-2.59037873262014	-6.15280277239920	-5.50396734656636
H	-0.71234715109417	-5.15039233067856	-4.34003061347709
C	-6.27456754869673	-6.53473084966810	1.64249709580283
C	-6.35439423578948	-5.21892108554037	2.02015889810626
C	-5.55183195360596	-4.23280746097007	1.39507683625761
C	-4.68755836491134	-4.60901960065364	0.32279242462169
C	-4.60814504662144	-5.98129340435408	-0.02161948265469
C	-5.37640449190591	-6.91868564432603	0.62579292244017
H	-6.28124007190197	-2.62871885737725	2.62465521696872
H	-7.02119557186102	-4.91056989294383	2.82072165874222
C	-5.59570002611732	-2.89314522213607	1.82452356886833
C	-3.91602401350946	-3.58963643225119	-0.32679110469829
H	-3.92603758094293	-6.29781291339609	-0.80247382815756
H	-5.29110028299883	-7.96498530351307	0.34808947892468

H	-5.02647234559159	-6.51114860706890	-5.72618013809766
H	-6.89041538462546	-7.28375909799162	2.13170451527654
C	-1.31868567461117	-2.90415546510568	3.61069008403055
C	-1.40187887982334	0.02579603025672	4.68842135634551
C	-1.00280248376191	1.23823718137807	5.29687580277227
C	-1.55441959851107	-4.19275530468688	3.05940018148365
C	-5.27374260290234	-3.20071219494608	6.37622268779628
C	-4.14012163212510	-2.51423166249783	6.01302202075132
C	-3.23264971457620	-3.06113811773030	5.07287285673005
C	-3.53229019222196	-4.32974144482022	4.49203603530861
C	-4.69118243153572	-5.02590832598671	4.91364275539171
C	-5.54643361965814	-4.47460874010020	5.83429899904838
H	-5.96357268440566	-2.76050364653842	7.09027825713548
H	-3.93481691624668	-1.53680761454223	6.43777171614866
C	-2.05030855841578	-2.37599469653847	4.65960877943019
C	-2.66129843217932	-4.87522429477866	3.52822276249049
H	-4.89738220149560	-5.99903462560067	4.47674443336340
H	-2.86252460134152	-5.87516728686600	3.15195605757312
C	-0.69589230886281	-1.14934504483787	9.52299772481094
C	-0.60779868400554	0.01122600198855	8.79705519911878
C	-0.88663239225642	0.02300303006862	7.40920222898395
C	-1.29998093502402	-1.18689562899995	6.76861780540906
C	-1.34266734394232	-2.37411106185321	7.53924570665936
C	-1.04832182165412	-2.35358570680904	8.88134649987499
H	-0.46441631505386	2.12695986368095	7.15216804872632
H	-0.30790980614018	0.94099079425762	9.27366924673852
H	-1.60347088444196	-3.31108201244674	7.05965648496595
H	-1.08192114262664	-3.27709566649557	9.45186602621314
C	-0.75822583405818	1.20743978712425	6.65157149662805
C	-1.59872427314043	-1.15499382035573	5.36918022797786
H	-6.43585791516974	-5.01425633236964	6.14682265033355
H	-0.47373599918791	-1.14780702410795	10.58597544136734
C	-4.89950494742125	-0.51711103605771	1.75093353698784
C	-4.86290159168629	0.58251175486094	0.87849655369504
C	-5.09186107163462	-0.29477857658862	3.12599131153823
C	-5.28146329221811	0.98366144346086	3.63093758648473
C	-5.25654282008732	2.06555369248789	2.75278025886748
C	-5.02407299288321	1.85758409622862	1.38509801656626
C	-5.40301779572729	3.49868579803808	2.98110775724637
C	-5.22990641478539	4.16696625721562	1.75228269410261
C	-4.89479577651948	3.17372112994973	0.65365906359918
C	-5.65657217503327	4.20552274509590	4.15334917870430
C	-5.75288073472338	5.59256534149841	4.09196532324443
C	-5.61319558647495	6.25662628795389	2.87360197550092
C	-5.35467188868218	5.54846348999296	1.69711094321855
C	-5.71821806678469	3.34632163980829	-0.64274725279854

C	-4.95521802870812	4.38893244601152	-1.49917345410500
C	-3.54003356452983	4.51204204626088	-0.87827084227832
C	-3.44298945624880	3.36401413829314	0.12711467784822
H	-4.72553253413401	0.43602473234569	-0.18646332603219
H	-5.07138640827905	-1.13909058525858	3.80921083104111
H	-5.42841408024987	1.12840579040213	4.69842694057688
H	-5.77581103778785	3.68768958619822	5.10151271471447
H	-5.94556315881348	6.16182996803830	4.99642523067342
H	-5.26161834274868	6.08518190430835	0.75829386584105
H	-5.74646789018227	2.38308711961092	-1.16410559158656
H	-6.75182146062563	3.63550697454245	-0.42832755268456
H	-5.46911901114088	5.35591352342694	-1.50275628039826
H	-4.89796359906599	4.06256484104146	-2.54209502332187
H	-2.75081785697800	4.46083925179463	-1.63176960596208
H	-3.41765105435405	5.47386925592110	-0.36706165526907
H	-2.73118198827437	3.53074575483985	0.94129545417879
H	-3.15826641923331	2.43655328359338	-0.38412531591109
H	-5.70766762295496	7.33820412949814	2.83804949578588
C	0.55521824023168	-3.48600650558254	-2.68548473817909
C	1.03871755691489	-3.22119415405887	-3.98300147955033
C	1.41494405001451	-3.30631046871319	-1.59009813180867
C	2.72350433988176	-2.87459298601242	-1.77572307653880
C	3.18876480056319	-2.63077338589232	-3.06397164127290
C	2.34421672344287	-2.81665453063958	-4.17419923785142
C	4.47696077328774	-2.13790542233248	-3.53819999496727
C	4.42069886494552	-2.02791334194562	-4.93774361793892
C	3.07743317806815	-2.48588206888934	-5.46326242042796
C	5.62773033092893	-1.78400540424749	-2.83883306638925
C	6.72725602287479	-1.32494801680275	-3.55724458643274
C	6.67432694294034	-1.21678302644422	-4.94758105584619
C	5.51938907061324	-1.56988613180875	-5.64707445383153
C	2.32984646945876	-1.46451198969266	-6.35302810399948
C	1.33572222223868	-2.28918154150570	-7.19878419180690
C	1.85345454390164	-3.75300438835255	-7.16954438009359
C	3.19002755587829	-3.70550377278217	-6.41442277842746
H	0.35614047975967	-3.30223698173728	-4.82191377318652
H	1.07400081499972	-3.51610382239828	-0.58381970279251
H	3.35974210270711	-2.71937832808351	-0.90990845363690
H	5.66593149042875	-1.85098341624637	-1.75539090781783
H	7.63188131922956	-1.03711398200699	-3.03078942860733
H	5.48954417628160	-1.47675827222023	-6.73055479761345
H	1.84848832327981	-0.68499239787897	-5.75492099280735
H	3.06120904105655	-0.97050507200179	-7.00367974078731
H	1.27549415307800	-1.89773151231901	-8.21914276545700
H	0.32429188543074	-2.22736421049816	-6.78615631950255
H	1.14409201649038	-4.40337411036093	-6.64577255575568

H	1.97573187642915	-4.17048938339323	-8.17390237969981
H	4.01365102098761	-3.51285573269647	-7.11308669298133
H	3.42266537645550	-4.62745503380754	-5.87187121696783
H	7.54068499030579	-0.84965223333686	-5.49016348536831
C	-0.66969899945796	-4.83643610639731	2.06217331842067
C	0.73351772716782	-4.83179464851870	2.17646295757038
C	-1.26644481050772	-5.55150893292892	1.01572141007367
C	-0.50914021084460	-6.20275194026710	0.05335914978647
C	0.87538752251153	-6.17872239134743	0.16459886185365
C	1.49348739925456	-5.52917315124562	1.25098425136755
C	1.90868392154798	-6.71002507499464	-0.71442894823815
C	3.15733175975418	-6.45028461124187	-0.12912908178309
C	2.99700832694078	-5.76995755457119	1.21432453630437
C	1.81736345302907	-7.31939968536904	-1.96339032454333
C	2.99309194741755	-7.66858173808988	-2.62145459385153
C	4.23593191136409	-7.41585229253537	-2.03769973194926
C	4.32421515140125	-6.80850951009368	-0.78429073318959
C	3.42048165545599	-6.71797549045038	2.39008822220404
C	3.96812974632421	-5.78182351320747	3.46665696893115
C	4.77252776127688	-4.77271177644788	2.64449637690178
C	3.88246330176276	-4.49922020409105	1.41842793399436
H	1.20587284245320	-4.29605529897736	2.99333025119530
H	-2.34681195879643	-5.55496199693299	0.93847797867722
H	-0.99643298736363	-6.70375698831508	-0.77978649937322
H	0.85017502508764	-7.50500913133575	-2.42425150880981
H	2.94578058168894	-8.13703383371463	-3.60035538210079
H	5.29747363650293	-6.60704254012272	-0.34172153048036
H	2.59015263202504	-7.35080569833607	2.71805745441138
H	4.22460537261330	-7.37605305802687	2.03743482859983
H	4.56826778998397	-6.30818996683040	4.21679563378166
H	3.14789318360468	-5.27814649331199	3.99435394837214
H	5.01950633344429	-3.85953013127566	3.19387351651789
H	5.71832928309819	-5.23550139252459	2.33259957495343
H	4.47279310955788	-4.28761261426116	0.52201277018206
H	3.24191766879768	-3.63232361142668	1.59128375195196
H	5.14340588086892	-7.68854206526005	-2.56859348572255
C	-0.90716897171979	2.51409844002088	4.55014791347191
C	0.30742398615634	3.21158083387309	4.48386367326443
C	-2.06963065951657	3.07552529635994	4.01256962620603
C	-2.04605779305695	4.33737561709829	3.43200901166397
C	-0.83855206752857	5.02335262071058	3.36733330106164
C	0.34157553556698	4.46478636578298	3.89117454544816
C	-0.52900648525263	6.32930596371950	2.79798731085312
C	0.84232930927971	6.57196459968410	2.97194612024221
C	1.50894634895707	5.41809123237026	3.68968673578866
C	-1.34397949880205	7.25407233563067	2.14860280413238

C	-0.77175553806413	8.43590623776082	1.68471551518794
C	0.58955044412702	8.68650186589822	1.87052539378806
C	1.40408254208914	7.75428511189449	2.51578370354937
C	2.69342477322970	4.77859034499457	2.92014408487766
C	3.59148846066050	4.12004905857168	3.99292974753157
C	3.15251575245224	4.70780478350259	5.35898576797106
C	2.17909434353983	5.84424734316134	5.02057228688749
H	1.20262508839165	2.74257940482675	4.87729261071488
H	-3.00326234731985	2.52614640767680	4.07784776215678
H	-2.96291379301413	4.78202375930585	3.05565024744711
H	-2.40532269988094	7.05914883940201	2.00928147489320
H	-1.38793946516027	9.17092699309527	1.17463592706607
H	2.46505937860189	7.95626140031878	2.64440292131385
H	2.34719629612173	4.07300494605256	2.15896947184044
H	3.23754046675212	5.57499623796445	2.39950923823134
H	4.64535308443866	4.33317997513543	3.79150297128407
H	3.48849449960395	3.03194168542598	3.97270764816391
H	2.65297045237693	3.94666297123370	5.96821734312611
H	4.00117640872070	5.06908406037655	5.94887665756950
H	2.73409412395145	6.77311495490433	4.83732875171925
H	1.44296077391729	6.04703453870248	5.80559695031950
H	1.01908222850804	9.61453445378044	1.50394586922828

I’

C	1.75247692667825	3.00751388884615	-2.27689938981714
H	2.36691422372733	1.08414078527682	-1.62545185738921
H	1.38419998901603	2.07174465415633	-0.40498855150074
C	1.80266882592274	1.98591984568531	-1.40381152265983
C	1.04304446890650	4.26448239491567	-1.94411388258187
C	-0.03398987656178	4.27822471617413	-1.04511447746675
C	1.45137528851240	5.48783517368147	-2.49373776855736
C	0.82864273992249	6.67957734634313	-2.13823278409042
C	-0.22667107329595	6.67582895042524	-1.23373447459298
C	-0.65379469289461	5.46682803953675	-0.69183483921419
H	-0.39066108938016	3.34645980335517	-0.61389768675243
H	2.28088373690890	5.51716798116449	-3.19314381833000
H	1.17625115195905	7.61538173429026	-2.56703406277174
H	-0.70726900259920	7.60293585586595	-0.93662071348121
H	-1.46857106611367	5.46002178229603	0.02335245748628
C	2.48885854442564	2.92578435386321	-3.59067707653650
H	3.25998727842046	3.71028232173846	-3.60521780606288
H	3.03488983881575	1.97663386072287	-3.63890185554953
C	1.60589481362814	3.07711872292230	-4.83622827636630
H	0.87802924249780	3.88548558260181	-4.69077120236595
H	1.01288800812055	2.16145252146661	-4.96694835774586
C	2.42821724410176	3.34863207831210	-6.09394927917911

H	2.95018585272474	4.30904827838407	-5.97597044072603
H	3.21630806534054	2.58801811491153	-6.18769640273540
C	1.57609627764999	3.38077940787579	-7.36003553170924
H	0.80315581669448	4.15493647410180	-7.29251367212831
H	1.06674527200096	2.42266248224890	-7.51777669464122
H	2.18169560677410	3.58717248202684	-8.24887166655285
O	-2.63382199084656	-0.86614698081093	-0.75902786811708
O	-0.42745905772336	-2.05196095791018	-1.01686164883897
O	0.58991964810717	-1.51248408883649	2.53705095750898
O	-1.05807398288775	0.42909415697677	2.89887057612983
P	0.19181522376435	-0.03296565500013	2.00038411494268
P	-1.06246001964762	-0.61632943165035	-0.70897502438835
S	2.91904829820994	0.77265101668252	1.81784300330251
S	-1.99008345204082	1.15754711642757	-3.06972362460031
O	3.08283676750739	-0.18917905653604	0.70969043925112
O	3.57508829498116	2.07964621773118	1.74339450511067
O	-3.35655651438485	1.02551267480565	-2.58828626376445
O	-1.39663761096302	2.42850019329142	-3.46214554301693
N	-0.87462008362843	0.44126469683861	-2.00831872522466
N	1.40837592946231	0.94540568812519	2.31360436524381
N	-0.37928099569051	0.00041314191805	0.52069431745110
H	0.00876957999146	0.99025865072293	-1.98813802372989
C	3.81925351389806	-0.11043220005066	3.27205912902552
F	3.56712129252783	-1.43589521385292	3.19174613827750
F	5.14231721736105	0.08987258829442	3.07384353744589
C	3.43633905863647	0.37675628573630	4.69825395249696
F	2.24341945016744	-0.16797845096232	5.03226313606882
F	3.32454633580751	1.72360615171711	4.70248995654794
C	4.47819523453166	-0.02061629408375	5.79994174279343
F	4.91105896134909	-1.28602907608573	5.58275098041005
F	5.53150561065539	0.82006768477410	5.73666500344946
C	3.91313500363936	0.03108535699166	7.25676474460495
F	3.06229656273780	-0.97436745409124	7.47785877100483
F	4.93244911140464	-0.07786236967171	8.12295585583587
F	3.28776386191898	1.19707886094360	7.47675317542385
C	-1.82160503725522	-0.00133090243957	-4.58249818221812
F	-0.51946336237386	-0.28628280744180	-4.73267020118839
F	-2.51094302072425	-1.12336578441646	-4.31172848922408
C	-2.36242028292739	0.65135634177187	-5.88996327740669
F	-1.45453987400796	1.54782689265801	-6.33176361411208
F	-3.52009806313423	1.28878798407819	-5.61537100794686
C	-2.62798862199145	-0.40202814912861	-7.02299990394603
F	-1.63979514260262	-1.33098177031132	-7.01302524726506
F	-3.80533236549687	-1.01214908483937	-6.78750317858532
C	-2.67889784632454	0.21269172135082	-8.45923706235290
F	-1.45855821565125	0.57716501393603	-8.86643873893202

F	-3.15467346103075	-0.70745333563013	-9.30861575281249
F	-3.48847666115979	1.27973148262285	-8.47755480232102
C	-0.88890577168207	-2.79725418589438	-2.10936984269743
C	-3.20055325641966	-2.01848828902084	-0.19070800000868
C	0.01929974249747	-3.00059147606857	-3.18460121040453
C	-4.01059268170485	-1.83749926294693	0.96089809599973
C	-4.46544702571157	-4.99140738646221	-4.49823066994781
C	-4.02977169662011	-4.35086384969315	-3.36324918472658
C	-2.67047771240214	-3.98743199951233	-3.21423656932353
C	-1.77033956529215	-4.25812484460489	-4.28854652061383
C	-2.24520897775726	-4.94781321081886	-5.43187529476677
C	-3.56414008597611	-5.31059090896898	-5.53492181367444
H	-5.51557757161006	-5.24803524124789	-4.60043489252537
H	-4.73422387430175	-4.10561469818697	-2.57572458039894
C	-2.16613603750205	-3.32427641141296	-2.05124235886066
C	-0.44222420415461	-3.79543783448325	-4.21919031826981
H	-1.54758453404352	-5.15944574930747	-6.23824647789034
H	0.22949238029844	-4.04583963884880	-5.03509995472404
C	-4.73260674049001	-6.65039026327340	1.01997016471640
C	-4.97367167043756	-5.39691232375342	1.52103835677841
C	-4.37950471530854	-4.25211351545831	0.93521654017337
C	-3.55737720464065	-4.40223163319993	-0.22293850611875
C	-3.30689458131867	-5.71437505497584	-0.69605076929823
C	-3.87503354624491	-6.80771765535417	-0.08771480346730
H	-5.24351779307241	-2.88272696058598	2.35068771239521
H	-5.60973849323316	-5.25842352284030	2.39096586224711
C	-4.58979564670527	-2.97431951751813	1.48757591151311
C	-2.98497237000706	-3.22872453453901	-0.81864927271162
H	-2.64885928133743	-5.86025565399529	-1.54484498232138
H	-3.65951406625668	-7.80247459197451	-0.46617455292860
H	-3.92168530985087	-5.82491684797731	-6.42191525061066
H	-5.18878611478369	-7.52211465626667	1.47995014607320
C	-0.33879858994987	-2.41554923414565	3.04979887832946
C	-0.82490294141171	0.41011906770677	4.28069323340199
C	-0.62283733201233	1.64666197503966	4.93232680658365
C	-0.40529642774232	-3.69895813890749	2.44023856422279
C	-4.03290089041050	-3.47993575662795	5.98818927674549
C	-3.04762007444979	-2.60178654149227	5.60524669424058
C	-2.11648913078740	-2.95007815618392	4.59572539594905
C	-2.24405412076370	-4.22285392055170	3.96317537615747
C	-3.24958597611321	-5.11756765563232	4.40405709367256
C	-4.12720804812712	-4.75660273467956	5.39527081170727
H	-4.74310081870948	-3.18939439032662	6.75677514681001
H	-2.98195707904845	-1.62325379668171	6.06965974257072
C	-1.08461852236633	-2.06154731822618	4.16225653991144
C	-1.35852716304944	-4.57324884633126	2.92533052776891

H	-3.32020736390088	-6.09104931821066	3.92651771476854
H	-1.42388701727352	-5.57154532562585	2.49972913731630
C	0.17973114209449	-0.81132771189594	9.04877951582701
C	0.04334285247119	0.37419927620067	8.37190964588313
C	-0.26667941882066	0.39127491079754	6.99083499445544
C	-0.47061365150143	-0.84649292349191	6.30414327674043
C	-0.28256335834347	-2.05158664693670	7.02387205013402
C	0.03246733732174	-2.03247781416318	8.36100077304390
H	-0.21313751977868	2.54643254726567	6.81475924691668
H	0.18584551233587	1.32307739369470	8.88261273130520
H	-0.38157429384674	-2.99991484347967	6.50743126095655
H	0.17830221348630	-2.96925539735168	8.89081555674976
C	-0.36149417638605	1.61037053968606	6.28197132769706
C	-0.80614547492213	-0.81382334980032	4.91277299783148
H	-4.89835923757414	-5.44830776023204	5.72200594748688
H	0.42364763625602	-0.81233084001119	10.10699282284947
C	-4.27837359094444	-0.51664121984195	1.56375669991328
C	-4.50179566131701	0.61902763458102	0.76991978846330
C	-4.38721956564717	-0.40861967479494	2.96069552350516
C	-4.73814971969852	0.78966984315062	3.56743562535181
C	-4.97017173326131	1.90721550935619	2.76768819985942
C	-4.82888045458808	1.81604215784603	1.37562745141877
C	-5.34227085760843	3.27827184184608	3.10041679410032
C	-5.39425856121512	4.03097668317426	1.90966078192043
C	-5.01584562911694	3.16390476899543	0.72127622426133
C	-5.61424953748830	3.86604317372147	4.33290027641942
C	-5.94484790193816	5.21752170854170	4.37365599488782
C	-6.01124629470715	5.96348494210865	3.19721207969096
C	-5.73895086155103	5.37456864622060	1.95954921604320
C	-6.01396458216923	3.21357925456346	-0.45690996371874
C	-5.59729295767063	4.43158323212248	-1.31223093329347
C	-4.13283041716214	4.76152332862158	-0.91558247544448
C	-3.70406551951287	3.64113104632621	0.03994574829467
H	-4.43704618866178	0.55537463724234	-0.31064656530118
H	-4.17492880352764	-1.27669394234878	3.57794378221427
H	-4.81627083383461	0.84522580364775	4.65051738082393
H	-5.56844845993196	3.28409399283149	5.25000202801045
H	-6.15622953416959	5.69408820609137	5.32637883607365
H	-5.80330340866151	5.97458515693017	1.05730355665808
H	-5.89630792459257	2.29290311281381	-1.03937740759092
H	-7.05186350147587	3.25854600896101	-0.11219193469471
H	-6.25494058881473	5.28730932259492	-1.12649565004973
H	-5.68256982128327	4.19994097068153	-2.37833872582339
H	-3.46715208486031	4.81765341782018	-1.78211822240604
H	-4.07464305969441	5.73479162592413	-0.41512918519288
H	-2.95308676261609	3.93502052307555	0.78014155700967

H	-3.30113506065831	2.79679564035433	-0.53210013740924
H	-6.27993711586037	7.01506048795696	3.24278917580417
C	1.34854089393734	-2.35896634469808	-3.27511803257837
C	1.82507177255125	-2.03181488290260	-4.56177250105638
C	2.13953386912856	-2.02385259869205	-2.16269952168280
C	3.33338448608776	-1.32813034652419	-2.31434581733170
C	3.76344886991638	-0.97863014190835	-3.58820338307967
C	3.02539776565600	-1.37186142258720	-4.71833393577833
C	4.87593401156914	-0.14501973556997	-4.02779719879966
C	4.82905457019379	-0.04782081931748	-5.42962879476049
C	3.70335253568469	-0.89298830023187	-5.98962307745713
C	5.83731495622393	0.54603023003797	-3.29411859934796
C	6.75701895112796	1.33434333621956	-3.97975699036255
C	6.71710307049478	1.42668892394596	-5.37197428135155
C	5.75368544390276	0.73174779565750	-6.10580541840503
C	2.74263674279589	-0.18998867714096	-6.97590509283728
C	2.13507074429664	-1.30778336322191	-7.85936128340287
C	3.00058493115242	-2.57184683064420	-7.61690536719913
C	4.22795262201465	-2.07858046282038	-6.84361648840738
H	1.20124595386506	-2.23328502098951	-5.42520022152617
H	1.82984609825343	-2.29634898427973	-1.16308689919546
H	3.89040393895925	-1.03557770091129	-1.43082998853017
H	5.85925514058450	0.48645040069020	-2.20899934117996
H	7.50966275667860	1.88834858255402	-3.42636747961884
H	5.73082219986525	0.81618475131093	-7.19009833615825
H	1.98612431397119	0.39503905762109	-6.44465655993910
H	3.31706157472183	0.50904741437833	-7.59440819714690
H	2.14793298789927	-1.01147616792009	-8.91299778249006
H	1.08590636370151	-1.49202712740495	-7.60480878092261
H	2.45268788690161	-3.30575327688596	-7.01510641136020
H	3.27970812855764	-3.07300442932751	-8.54915047930048
H	4.98582318336281	-1.69040816490232	-7.53638178180703
H	4.69908770387572	-2.84990159384648	-6.22558932155074
H	7.43991968028242	2.05097542857208	-5.88928826247732
C	0.50135479742793	-4.13946371863354	1.35567031283152
C	1.89206929010254	-3.92673495719266	1.39720112152417
C	-0.04701756236329	-4.86740845396906	0.29197244366411
C	0.73672959106006	-5.32316055204270	-0.75803513082882
C	2.10470623855879	-5.08452436804426	-0.72249440127130
C	2.68698043200217	-4.42779952522067	0.37904434718752
C	3.14561725242589	-5.37632062515358	-1.69864282877837
C	4.37601839315633	-4.96601042947205	-1.16277120680464
C	4.20300590169037	-4.42240942466486	0.24052183953479
C	3.06493110378800	-5.89756227583231	-2.98757328022961
C	4.23326228836207	-6.00691435671398	-3.73636266298868
C	5.45913521417686	-5.60523136347234	-3.20221922554134

C	5.53727703205869	-5.08616370856341	-1.90888450361549
C	4.84583380557234	-5.37642140271498	1.30720676294802
C	5.31529480874577	-4.44743330177452	2.42574382158379
C	5.90028226073187	-3.26815409675023	1.64659379113786
C	4.89356604937370	-3.04584149919356	0.50313448531945
H	2.32771144286980	-3.38408974998689	2.22953674372794
H	-1.11675684690586	-5.03638200339075	0.27221832083047
H	0.27936980792781	-5.83764827244530	-1.59993661445639
H	2.10866404536112	-6.19991360532019	-3.40782058294661
H	4.19264975585998	-6.40390582027916	-4.74679456602695
H	6.49576377442574	-4.76722387160811	-1.50480585193670
H	4.14693265016019	-6.15540845104718	1.62712053705764
H	5.71544038530699	-5.87033817491379	0.85579876168261
H	6.03587795844437	-4.92692544482384	3.09724836627369
H	4.46459687097396	-4.11583792935356	3.03518743272812
H	6.04417537004104	-2.37111897188386	2.25605661825306
H	6.88049564182588	-3.55369964380759	1.24166924071805
H	5.37482182855943	-2.67259215265990	-0.40568703440506
H	4.14178984966086	-2.30790988917035	0.79148120385902
H	6.36044520282430	-5.69268125322473	-3.80216882776073
C	-0.73832017024283	2.94518724305548	4.21617466991857
C	0.40050187826855	3.65170662026912	3.81174890288360
C	-2.00969177889101	3.49320792432264	4.01757504811528
C	-2.16834807733318	4.75123780605340	3.44639838243201
C	-1.03116286938730	5.45686932694590	3.06364240666921
C	0.24780938950515	4.90099072651226	3.23304299269658
C	-0.88044277023667	6.79322322544702	2.49436829448666
C	0.49082786320192	7.04810438300658	2.32307626201414
C	1.32341605612826	5.85572425980144	2.75070922612010
C	-1.83453454646670	7.74184209071718	2.13416868199915
C	-1.40255983477587	8.95792177129693	1.60907190342839
C	-0.04040736815646	9.21538747126275	1.44426582564245
C	0.91315999363896	8.25893448901029	1.79825106496923
C	2.18793430358334	5.25468707517156	1.58134782896552
C	3.65174165166268	5.48722397974790	1.97228676608955
C	3.62518564039372	5.39493404982172	3.49808215459090
C	2.37189260810597	6.19769203352269	3.85993853861476
H	1.38368971613298	3.21148763131045	3.94433904878877
H	-2.88183990499022	2.93082264894979	4.33400516510163
H	-3.16396905430170	5.17028305659924	3.32491083438638
H	-2.89544339890088	7.54076308926277	2.26691468716192
H	-2.13027427903243	9.71422487333833	1.32803453139878
H	1.97153471838564	8.46390296700087	1.65305710787532
H	1.99916689580307	4.17870380257535	1.51311696953535
H	1.92079811524948	5.69783044982324	0.61840698774690
H	3.97787696598897	6.49198015121069	1.66884839443287

H	4.31837032196649	4.75575198695120	1.50573944627362
H	3.51712424820742	4.34476572008673	3.79733247385058
H	4.52819023192924	5.78698566352186	3.97906790696606
H	2.60079722704998	7.26937294532097	3.81199814241584
H	1.98091375650292	5.98924392844393	4.86087232658654
H	0.27992698016688	10.16875383160643	1.03321564328877

TS0

H	4.226626004740	1.002988203681	0.665508076609
O	4.737659018065	1.195590314193	-0.158008537663
C	6.006563292719	1.656511744073	0.140206895547
C	5.971462264000	2.763956326341	1.252095277823
F	5.857558926918	2.203097012925	2.468872344929
F	7.082670901935	3.523487630554	1.248322438557
F	4.915328040633	3.567447494674	1.068738841960
C	6.558781867157	2.266585190655	-1.187626282420
F	7.890851275487	2.436716879987	-1.140928914879
F	6.273902970601	1.477114602105	-2.230706662987
F	5.992050866402	3.466922536002	-1.425813580348
C	6.921262644361	0.470810150139	0.609557461453
F	6.248562766927	-0.311786373297	1.465565477729
F	7.283487489858	-0.295941175591	-0.438343688305
F	8.043792799848	0.896402597674	1.214390540903
H	-1.676479422232	3.275051433085	-3.832478745793
O	-2.322584540355	3.967318971375	-4.079448604014
C	-1.789296068613	4.897620148182	-4.952654555866
C	-0.676453335260	5.748522611516	-4.243990381719
F	-0.387806321110	6.878058223498	-4.911857056851
F	-1.053481320484	6.078001181043	-3.004211709458
F	0.457147998741	5.021511240806	-4.143209866536
C	-2.985205241007	5.818142910600	-5.360262102070
F	-2.674804897390	6.570004670988	-6.431850193455
F	-4.067000478087	5.090541942262	-5.655842584423
F	-3.310366865235	6.642948602405	-4.349530517434
C	-1.194890114039	4.192154523185	-6.220803770047
F	-0.476290365289	3.117011879231	-5.841347754583
F	-2.171299265898	3.757605896081	-7.032665080416
F	-0.389650194007	5.000501911865	-6.929397785385
C	1.891695618010	1.541986488336	-1.706941537535
H	1.889686474195	-0.503205696697	-1.155652890823
H	1.098265720397	0.653528461012	0.042240769484
C	1.416956903751	0.463327941304	-0.980116141698
C	1.578451998965	2.908262625452	-1.300436317799
C	0.446204393107	3.180923793378	-0.509889585016
C	2.423121733090	3.975854571368	-1.649070499563
C	2.156429793371	5.262764775772	-1.204914657162

C	1.026703704311	5.517533240732	-0.433708922391
C	0.170543951635	4.471415432745	-0.093980796160
H	-0.234127214836	2.378989792764	-0.238397556150
H	3.314378143681	3.793638039431	-2.240182864849
H	2.832209534638	6.071725077386	-1.466202090198
H	0.808011036421	6.524660112642	-0.091031141418
H	-0.709588162971	4.671004618484	0.507143901452
C	2.753205279790	1.300846506000	-2.902201399967
H	3.732499911671	1.751270548193	-2.683653912042
H	2.939957651861	0.226252003163	-3.003992825140
C	2.225315348286	1.869747975808	-4.228583939892
H	1.854639670068	2.890561467996	-4.086801066953
H	1.360357025978	1.275869490120	-4.551534190807
C	3.308913820656	1.858339141643	-5.305026282897
H	4.130513242841	2.517114776994	-4.989430237004
H	3.742688301107	0.851721858757	-5.378443202506
C	2.783015422546	2.303226644677	-6.666318415227
H	2.378331309267	3.320702087202	-6.619547546864
H	1.976287934878	1.645539665823	-7.010987853408
H	3.573886138442	2.290263653610	-7.423495125611
O	-3.192482757287	-1.276235986007	-0.495925705616
O	-1.162121595424	-2.782596480548	-0.515091101826
O	-0.242272648586	-2.165312506378	3.110745887964
O	-1.602278760083	0.018425354833	3.298681881086
P	-0.447217591662	-0.701472651377	2.442799828111
P	-1.600240737101	-1.247324032444	-0.345463347910
S	2.370470685151	-0.347831793516	2.299793430013
S	-1.917910523329	0.577956764857	-2.690190230098
O	2.453661247642	-1.424851007747	1.295559296490
O	3.220061354574	0.849505818191	2.097554972350
O	-3.304408739530	0.863266328515	-2.325152983414
O	-1.009657151659	1.673813579343	-3.078173647719
N	-1.075011395060	-0.330407680446	-1.624309541580
N	0.909304477447	0.117229010870	2.705105977603
N	-1.001628060942	-0.703322326334	0.970884441859
H	0.177950310099	0.141363658657	-1.448262263364
C	3.134554250505	-1.178513570384	3.860704516410
F	2.720096651022	-2.461922430596	3.882613372379
F	4.473234656147	-1.153481612041	3.684540397765
C	2.782395010281	-0.523334444109	5.224504840352
F	1.524698303370	-0.887176378929	5.560167653335
F	2.839698587390	0.822154001772	5.105877251390
C	3.745267645578	-0.944770007011	6.388385306536
F	4.046971715096	-2.260411160741	6.276627333837
F	4.882064290313	-0.224300451179	6.302678173191
C	3.143979702476	-0.727036862994	7.814887435110

F	2.188619794045	-1.623553576284	8.071818901240
F	4.118350950443	-0.870718513196	8.725052173812
F	2.634148575783	0.509752515000	7.924558626253
C	-2.013088092112	-0.562419708790	-4.222055711531
F	-0.764884591912	-0.679274471624	-4.718876705204
F	-2.446664769170	-1.764398501990	-3.813406716353
C	-2.982040933378	-0.014975393302	-5.317460952935
F	-2.839682283787	1.324160096972	-5.405204656072
F	-4.248495299164	-0.301302374773	-4.950359858513
C	-2.713115089816	-0.632434821026	-6.730719613482
F	-1.604277293185	-0.066588583320	-7.252502134295
F	-2.509673671387	-1.966766596575	-6.599584996089
C	-3.876198675727	-0.430401740636	-7.754855718906
F	-4.234416100730	0.858475395794	-7.803154928242
F	-3.450315032170	-0.809283065460	-8.968124260702
F	-4.939000636510	-1.171982137114	-7.427524890609
C	-1.701309079956	-3.541484854589	-1.562413460874
C	-3.947103643286	-2.267234676544	0.151184067097
C	-0.813753260436	-3.947273229595	-2.592237868020
C	-4.781800514378	-1.863757596834	1.227163491030
C	-5.484972760797	-5.409854776515	-3.918679644865
C	-4.995501661867	-4.732846012086	-2.827803643691
C	-3.602401670554	-4.554251485206	-2.651235745720
C	-2.721422263531	-5.043814705847	-3.661565515025
C	-3.255748176841	-5.765387021034	-4.757763617285
C	-4.609120051681	-5.950443771573	-4.882789184339
H	-6.557747848207	-5.523627258994	-4.042746565290
H	-5.681546579796	-4.317884642814	-2.097547650368
C	-3.042247822002	-3.876370271611	-1.521684792400
C	-1.345848560741	-4.755738806833	-3.579413078267
H	-2.572176411921	-6.146488730257	-5.512293041898
H	-0.688521717851	-5.149604297079	-4.349722055083
C	-6.265546406102	-6.486316280977	1.612413611227
C	-6.341901911651	-5.169524143311	1.987367180299
C	-5.530511523732	-4.188163911507	1.366484446137
C	-4.660823313533	-4.569057678109	0.300827723082
C	-4.584612268623	-5.942588795783	-0.040177076740
C	-5.361387631245	-6.875754263634	0.603215587942
H	-6.260937213841	-2.579155025951	2.587054814555
H	-7.013029097402	-4.856867841479	2.782670351072
C	-5.570749547558	-2.847506828430	1.792229605290
C	-3.886162979653	-3.552615388496	-0.349017009853
H	-3.899319835727	-6.263244350649	-0.816503317646
H	-5.278671392604	-7.922672175172	0.327241487696
H	-5.010476450411	-6.492351942927	-5.733871628187
H	-6.888848739938	-7.231674178960	2.097733014439

C	-1.321206294160	-2.903885063758	3.599550251512
C	-1.416375249052	0.019858801154	4.688303007083
C	-1.025440206015	1.229257607841	5.308804336200
C	-1.550321963021	-4.189600382918	3.040002680956
C	-5.292788415362	-3.224565953339	6.338177953717
C	-4.159657113106	-2.532469376209	5.984196170505
C	-3.244728316115	-3.072708887309	5.047601761511
C	-3.536498965192	-4.339614836508	4.459104335634
C	-4.694818789460	-5.041731103137	4.871754547409
C	-5.557122380436	-4.497471187204	5.790043061216
H	-5.988677624102	-2.789567051473	7.049522304446
H	-3.960439079089	-1.555611095210	6.413276475378
C	-2.061529849204	-2.382835534960	4.645510969801
C	-2.658406834302	-4.877550883656	3.497128172721
H	-4.895695422888	-6.013300555713	4.429039851541
H	-2.855075105171	-5.875431896291	3.112924966061
C	-0.734029232314	-1.182478346598	9.521286019429
C	-0.645744371025	-0.016919227766	8.803358378156
C	-0.917605828458	0.002884672057	7.414293241594
C	-1.323931441854	-1.203737461574	6.763476794543
C	-1.367503876394	-2.396073479778	7.526282567560
C	-1.079938589472	-2.383441033958	8.869935296050
H	-0.501249249878	2.109776483430	7.172248070706
H	-0.351488840127	0.910826368797	9.287512500969
H	-1.623737985495	-3.330396909465	7.039195747918
H	-1.114370617495	-3.310702463176	9.434301846712
C	-0.789027346621	1.192230911699	6.664511739414
C	-1.615578193155	-1.164531134667	5.362868424113
H	-6.446468034350	-5.041554693035	6.094825083548
H	-0.517556813143	-1.187215284866	10.585435351784
C	-4.872692234588	-0.475150667419	1.720541402464
C	-4.821107772257	0.628138178621	0.853371037742
C	-5.087992106112	-0.257694642071	3.093559538617
C	-5.291390703003	1.017404842093	3.600642584953
C	-5.255642258444	2.102514682732	2.726958921145
C	-4.996077948077	1.900536411906	1.363394028816
C	-5.411374824429	3.534139127952	2.958299356653
C	-5.216456479525	4.207967480064	1.735581947252
C	-4.853950278837	3.220131718385	0.641094359270
C	-5.690322490542	4.235982268462	4.127855277881
C	-5.789634435526	5.623172142243	4.069811137822
C	-5.628222309732	6.292439856084	2.856970604293
C	-5.344493489932	5.589264228024	1.683055299853
C	-5.640444017459	3.398025806741	-0.676748151470
C	-4.855573983672	4.447320295627	-1.504808172754
C	-3.458302909266	4.569152103414	-0.843866592086

C	-3.388486444043	3.414250433907	0.155642456904
H	-4.659092976029	0.487297874416	-0.209065511435
H	-5.075330378512	-1.104212093133	3.774179107369
H	-5.457460648778	1.157058212619	4.666048970119
H	-5.827556395849	3.714103541366	5.071396521238
H	-6.002979733523	6.188192746892	4.972320190528
H	-5.236322362642	6.129034558446	0.747544096297
H	-5.648643731818	2.438091869111	-1.204389739067
H	-6.680826611552	3.683065171184	-0.491135588459
H	-5.370872454009	5.413635878777	-1.517914658528
H	-4.767157041932	4.124993176952	-2.546711509249
H	-2.651775207398	4.519331366181	-1.578734299697
H	-3.351825173200	5.528632307006	-0.324243791318
H	-2.703836455936	3.574291686071	0.994129990412
H	-3.090037541290	2.491756099404	-0.355495936830
H	-5.726746911438	7.373758575407	2.823388551349
C	0.585663937320	-3.489888222506	-2.703396514156
C	1.065718412879	-3.223509437126	-4.000739978753
C	1.450738790157	-3.325785658110	-1.610485339612
C	2.763725672299	-2.907373933994	-1.799187425225
C	3.225272429028	-2.658177782531	-3.088408746881
C	2.373853503506	-2.830196753608	-4.195259785228
C	4.509845207861	-2.158347017864	-3.567120568321
C	4.440788500523	-2.024454012462	-4.964580272972
C	3.100391008483	-2.495435575152	-5.486476447687
C	5.666671703406	-1.814557639466	-2.873176124616
C	6.757594323687	-1.336546667229	-3.592941894186
C	6.690565699253	-1.200680975147	-4.980103416736
C	5.530810750205	-1.547896347985	-5.675161760919
C	2.335675772607	-1.497416912788	-6.387639144655
C	1.360273216839	-2.348036708631	-7.231639612088
C	1.891676938237	-3.805499330793	-7.168313391106
C	3.231980346526	-3.724341003206	-6.424871302657
H	0.376960736041	-3.289233314390	-4.835756443935
H	1.107564385889	-3.532417251900	-0.604130945454
H	3.406190343157	-2.767060474887	-0.934920184206
H	5.716719220081	-1.907895370841	-1.792306113291
H	7.667068464318	-1.057281092649	-3.069815805171
H	5.490980797088	-1.437508245957	-6.756735653221
H	1.832504759657	-0.725227033711	-5.798357685898
H	3.058996439751	-0.992967687207	-7.039364657160
H	1.313253153123	-1.976774513167	-8.260152814706
H	0.341645667369	-2.286532748422	-6.836405461517
H	1.193245196610	-4.448118159896	-6.620962636246
H	2.010321612836	-4.248517102098	-8.162103047252
H	4.046680938675	-3.522341000929	-7.131531066201

H	3.486942435076	-4.634807635731	-5.873048172626
H	7.549974429343	-0.819418276101	-5.523976139993
C	-0.654270039797	-4.825869620995	2.048321543184
C	0.746807091096	-4.825292332191	2.184166901741
C	-1.237067557393	-5.532133049540	0.988532460733
C	-0.466825316201	-6.183096892778	0.036195762131
C	0.915799446196	-6.166329874641	0.170848135783
C	1.519112118519	-5.520982264317	1.267787903573
C	1.961038394338	-6.701337230917	-0.691905086334
C	3.201286856997	-6.447721541501	-0.086474997964
C	3.022817244885	-5.762931028026	1.252581808520
C	1.887373900097	-7.309837230098	-1.942422463678
C	3.071975045906	-7.665088007269	-2.581110593061
C	4.306361067733	-7.419289643033	-1.976970792620
C	4.376995666183	-6.811982003483	-0.722341120512
C	3.434129806824	-6.703195829837	2.438176075227
C	3.971732163683	-5.761028967709	3.514816965397
C	4.783103636837	-4.755761126427	2.694801385600
C	3.904293805571	-4.489277352089	1.459202307186
H	1.206831674067	-4.294851684536	3.011539087329
H	-2.316013346093	-5.529650065536	0.893874363137
H	-0.942833834923	-6.677529808872	-0.807299257178
H	0.927013544549	-7.490040706744	-2.419319182578
H	3.038190530912	-8.132848651344	-3.560892713168
H	5.343926203740	-6.616031608255	-0.263546343527
H	2.600201440970	-7.333378204739	2.762002547588
H	4.241287596752	-7.364073673568	2.097856686607
H	4.565417525843	-6.283043685498	4.273098656652
H	3.146716937626	-5.254980741174	4.032602580110
H	5.024704687925	-3.839621651135	3.241813293577
H	5.731884548521	-5.219860356174	2.393948252674
H	4.503257936516	-4.281330158514	0.567577561520
H	3.260456497430	-3.622672854015	1.621450005751
H	5.221019018955	-7.697203239359	-2.492639986197
C	-0.929536924254	2.512186265852	4.572924129580
C	0.289963837515	3.199780765225	4.496400219870
C	-2.094020020700	3.088021343846	4.055065755463
C	-2.066250401182	4.351657082149	3.478198964929
C	-0.852687808884	5.025614374284	3.399553545647
C	0.327545255145	4.456100050573	3.910613266742
C	-0.533351835074	6.321141298986	2.811164289196
C	0.843435902288	6.548033439183	2.965631866035
C	1.501954589555	5.398260056173	3.697725203950
C	-1.341891547837	7.243305500656	2.149918321055
C	-0.757685770614	8.406574823022	1.654306182189
C	0.608452472644	8.643017065174	1.822514048789

C	1.416420061445	7.713786241004	2.480935038624
C	2.685267312111	4.741743896392	2.941521308670
C	3.596692334386	4.118402353847	4.025498794755
C	3.132864842154	4.703998248746	5.383338286156
C	2.168511096898	5.840876326788	5.025963348580
H	1.186449633225	2.720448649543	4.874196776438
H	-3.031641681323	2.546448964413	4.127585759713
H	-2.983186497963	4.804287575074	3.111540871291
H	-2.406970395037	7.059980961271	2.024699709388
H	-1.368810379839	9.138329455233	1.133643437232
H	2.481020966355	7.904493420706	2.596867522094
H	2.337614930648	4.009227215099	2.207013295828
H	3.222105913120	5.522591836523	2.390838234688
H	4.644428842146	4.364556244720	3.828417955513
H	3.528435860128	3.027601506178	4.013739304940
H	2.618561282251	3.943116296150	5.980401296095
H	3.970210472886	5.062215073668	5.991023022394
H	2.729642643184	6.764066727852	4.832097825329
H	1.429508484300	6.058281142504	5.804251861392
H	1.046834159993	9.557274473659	1.432490093692

TS0'

C	1.61058394138507	2.90733105709257	-2.20971242912577
H	2.16129989273655	0.97239563830365	-1.53549957054410
H	1.16891694752064	1.96478236575273	-0.35335742933001
C	1.45996589309976	1.79478034990480	-1.38913910988686
C	0.97951731720963	4.17835630666914	-1.88093595331324
C	-0.10249052606806	4.23411201024852	-0.98234541093282
C	1.45057619368329	5.38322874405187	-2.43329325206061
C	0.87686772996171	6.59658187801456	-2.08390950261129
C	-0.19511153096663	6.63156890648905	-1.19660978112426
C	-0.68396966995615	5.44516043575279	-0.65460821896972
H	-0.50239204027308	3.32058708667476	-0.55321326508528
H	2.29246911836869	5.37478107463275	-3.11753616258234
H	1.27012567833425	7.51883688890208	-2.50092501720746
H	-0.64171357345699	7.57821077807614	-0.90752238505803
H	-1.51218722577280	5.47603209924047	0.04306325084174
C	2.43511030193342	2.79141051816501	-3.45202564655908
H	3.27174257031264	3.50109986370084	-3.35068362409723
H	2.89195546720882	1.79729953729939	-3.49399813631925
C	1.69492597466591	3.07835390851209	-4.77166982833136
H	1.02772237656277	3.94062866643101	-4.66048524938348
H	1.04445152982458	2.22524619815151	-4.99944662721895
C	2.67462318388828	3.31744925764497	-5.91837462415992
H	3.27528050395515	4.21165880693490	-5.69692563191664
H	3.38582478472125	2.48184497339267	-5.97203000249859

C	1.96610933134178	3.49349476952896	-7.25848750699835
H	1.27236573725785	4.34125824627186	-7.22737948606692
H	1.38347404444412	2.60185032679438	-7.51660412190903
H	2.68057979798006	3.67303889387651	-8.06854533726254
O	-2.66543699119016	-0.88993754754186	-0.80651714944648
O	-0.44584853932824	-2.05909973696753	-1.04776327768739
O	0.57635483342781	-1.50750919181433	2.56477508269465
O	-1.09233520157309	0.41837370332010	2.91698727594162
P	0.13511458744272	-0.05331510183736	1.98752331549130
P	-1.08792611105976	-0.61123931679137	-0.76583041415313
S	2.84978750776411	0.78621661572059	1.75608510401824
S	-1.85586749382793	1.19019953784700	-3.01490303890365
O	3.01806144184037	-0.18771453540409	0.65736979825156
O	3.48884755841648	2.10189080100281	1.64238249468967
O	-3.25098604893919	1.18691552848148	-2.57543802749759
O	-1.20714070771054	2.43568138409995	-3.43761007440811
N	-0.81112103624585	0.44170900950147	-1.99971764685350
N	1.34972773020320	0.95048298356598	2.27107535098895
N	-0.46448093459679	-0.06066505323458	0.53581196425238
H	0.36549692338620	1.19907745905346	-1.81364291347791
C	3.79542687991437	-0.06219541540640	3.20284509819003
F	3.55975772222411	-1.39116794780873	3.14772550829002
F	5.11142046592085	0.15325727941301	2.97190936955633
C	3.43911103466816	0.43956277129985	4.63056438330617
F	2.26178702443610	-0.11322658518542	5.00104677691671
F	3.31178945051498	1.78590358529983	4.61780629864263
C	4.51210670636438	0.07336191095091	5.71290531387458
F	4.96203080206028	-1.18686630934212	5.50264346573942
F	5.54902671398461	0.93147612828558	5.61388413775890
C	3.98018239946263	0.13599026523710	7.18205436970693
F	3.15903370504850	-0.88460914767496	7.44071978353363
F	5.02321240641220	0.06546965306896	8.02398908183175
F	3.33387966117582	1.29130386470316	7.39903441024294
C	-1.77970145868469	0.05419714521292	-4.54781975716291
F	-0.48806271355915	-0.26495824353704	-4.75494743687370
F	-2.48295006539931	-1.06226879827961	-4.26923633881041
C	-2.34428419032998	0.71470285177724	-5.84020787245682
F	-1.42918644571922	1.59345914224483	-6.31223457959681
F	-3.48098211715196	1.37823971907756	-5.54569020183261
C	-2.66084906194473	-0.32945014848461	-6.96871828449653
F	-1.68720302213714	-1.27383094785120	-7.00040006167428
F	-3.83911945401425	-0.92489030829222	-6.70014653057306
C	-2.75124200958470	0.28914588492216	-8.40122573252078
F	-1.54084489976125	0.64506391151938	-8.84753338687399
F	-3.25919527187294	-0.62531604720993	-9.23922026921047
F	-3.55194613438203	1.36306960519498	-8.39666066985660

C	-0.90012935634976	-2.80856168517692	-2.13431768382109
C	-3.20780132955627	-2.03658856154735	-0.21906494217072
C	0.00328642600051	-3.01082130147822	-3.21438246867932
C	-4.00798711794448	-1.85865762158468	0.94140963405014
C	-4.47172053967785	-5.03260849208736	-4.50840957057144
C	-4.03556758333827	-4.38714691823270	-3.37646544567722
C	-2.67828740874247	-4.01294876993108	-3.23385756849396
C	-1.78188017764264	-4.27842944592088	-4.31231055048276
C	-2.25674134713393	-4.97261138494228	-5.45279391736642
C	-3.57322602508162	-5.34592236890818	-5.54937732218976
H	-5.52041125505776	-5.29730875657802	-4.60524393592951
H	-4.73791901463885	-4.14582228064093	-2.58586053056857
C	-2.17373888848038	-3.34624868417251	-2.07361988282625
C	-0.45641370456296	-3.80791065150866	-4.24756001397742
H	-1.56129890594164	-5.17943133555164	-6.26238391589645
H	0.21357778260500	-4.05569888118815	-5.06577607551749
C	-4.70492588983872	-6.67513901696964	1.02701206492144
C	-4.94804123947745	-5.42061916172749	1.52489465423151
C	-4.36444519076865	-4.27519132584895	0.92978489762612
C	-3.55057873425006	-4.42535730335201	-0.23422899253470
C	-3.29780266974128	-5.73845470971535	-0.70403832440745
C	-3.85589914596555	-6.83237161443014	-0.08732396859523
H	-5.22383686746377	-2.90521052291802	2.34638722459839
H	-5.57840220617598	-5.28177357859111	2.39903808194462
C	-4.57617633106272	-2.99606150860113	1.47860933345108
C	-2.98913580971442	-3.25145419985780	-0.83844298708742
H	-2.64583259521979	-5.88396232210967	-1.55764219126468
H	-3.63874542097606	-7.82748890982785	-0.46403710960738
H	-3.93113258608646	-5.86375157153695	-6.43421739207900
H	-5.15342691247790	-7.54724095642476	1.49388309345374
C	-0.34457738052510	-2.41677903462693	3.07796122378609
C	-0.83518167782275	0.40890201085134	4.29126732182105
C	-0.62117079888108	1.64978807393880	4.93194401352593
C	-0.41482023601320	-3.69829915983723	2.46577157730029
C	-4.02449194066783	-3.48093930671108	6.03302783432326
C	-3.03900159545587	-2.60389374995508	5.64787944485839
C	-2.11458927490440	-2.95129840536863	4.63210698144806
C	-2.24737757020034	-4.22223692696754	3.99623158677454
C	-3.25352644663348	-5.11522129521083	4.43890671124728
C	-4.12568413276412	-4.75485778052288	5.43534129677184
H	-4.73024585321958	-3.19139385585052	6.80610816889019
H	-2.96778509426062	-1.62698259668385	6.11514836546083
C	-1.08582449881718	-2.06312873697401	4.19299489985491
C	-1.36719014105078	-4.57216968390390	2.95324337320378
H	-3.32965821190037	-6.08660121757444	3.95792060869409
H	-1.43771105598714	-5.56889478193859	2.52474227709881

C	0.26716482823035	-0.77814615738130	9.04769202135108
C	0.11699311793682	0.40284338961848	8.36563282163206
C	-0.22332287895268	0.41052481642486	6.99179210373894
C	-0.44358855929968	-0.83193561094335	6.31840823627520
C	-0.24409630865979	-2.03222715261678	7.04306376354703
C	0.10109778400606	-2.00403143817796	8.37263081554302
H	-0.17120724947248	2.56420303311237	6.79871516600444
H	0.27188665951836	1.35509578865808	8.86644565731695
H	-0.35864575663338	-2.98370759150429	6.53560078621000
H	0.25538349908691	-2.93718057339460	8.90645657320602
C	-0.33146865996568	1.62441145444305	6.27591387597279
C	-0.80365366011085	-0.80982508025149	4.93342484971580
H	-4.89769352062322	-5.44526850855868	5.76278497908709
H	0.53495852097391	-0.77198899999893	10.10011350024480
C	-4.28001215653206	-0.53753808697095	1.54280004277487
C	-4.50549349402117	0.59815667713217	0.74918444842152
C	-4.38926908575443	-0.42919610562453	2.94012434865571
C	-4.74567439903539	0.76702526470974	3.54743469320671
C	-4.98231136911510	1.88369113740785	2.74778066169856
C	-4.83868970660600	1.79323065873478	1.35612802344040
C	-5.35978571840574	3.25345833302544	3.08059989944388
C	-5.41529805255747	4.00607783641271	1.88970478705962
C	-5.03072749814761	3.14095642922688	0.70214051349307
C	-5.63219987809302	3.84143351998240	4.31296737172846
C	-5.96722055382811	5.19202451895175	4.35358373749248
C	-6.03807435423660	5.93738043152021	3.17707670024492
C	-5.76498846124904	5.34837630351944	1.93935788457271
C	-6.02409443112837	3.18555987354615	-0.48001260509696
C	-5.61201848299784	4.40640453610752	-1.33288419504954
C	-4.14907077597749	4.74146621164404	-0.93436634928108
C	-3.71920626406210	3.62448145630108	0.02541851239185
H	-4.43270559441407	0.53693456434064	-0.33109209368636
H	-4.17179364573794	-1.29559308596337	3.55805907820951
H	-4.82455082436155	0.82162717131986	4.63056806870664
H	-5.58372439153843	3.26000771059624	5.23027649410889
H	-6.17962339886936	5.66797382404018	5.30643386520930
H	-5.83402038742732	5.94702887089868	1.03640445299087
H	-5.89688765423784	2.26665482076867	-1.06283347650490
H	-7.06366704914208	3.22385198029383	-0.13959732331126
H	-6.27289561460697	5.25948862388756	-1.14604518643380
H	-5.69422462159765	4.17551912720827	-2.39922290970885
H	-3.48450036870886	4.79195647036192	-1.80253814284379
H	-4.09563788277986	5.71810844811450	-0.43901529748924
H	-2.97495946130638	3.92286379002644	0.77124099999649
H	-3.31172007542596	2.78279425049071	-0.54572006094291
H	-6.31178448790742	6.98771465536376	3.22243226434628

C	1.33126276526774	-2.36608173785544	-3.31089297031518
C	1.80266010709218	-2.04060097623432	-4.59914251906179
C	2.12505198388760	-2.02708946132323	-2.20201916160135
C	3.31939756339770	-1.33290018176209	-2.35940266712933
C	3.74768863992043	-0.99029069029267	-3.63622124844549
C	3.00307945272959	-1.38198858169848	-4.76220396546811
C	4.86968796006691	-0.17281793783612	-4.08367462006478
C	4.82150523620754	-0.08382963318309	-5.48619377903196
C	3.67630887082740	-0.90777371529158	-6.03807945201152
C	5.84746078360330	0.50411844695734	-3.35799955218763
C	6.78194783052352	1.26862536610283	-4.05140925353244
C	6.74089693027846	1.35173965204411	-5.44422911659614
C	5.76112458656735	0.67114069280541	-6.16999942888276
C	2.71604986636311	-0.17694436639807	-7.00610565200560
C	2.07816163643236	-1.27438880506654	-7.89313009941593
C	2.92664185856640	-2.55490292271847	-7.67820614073138
C	4.16948598521095	-2.09360180478639	-6.90949963379074
H	1.17401347021037	-2.24221792866442	-5.45867729394855
H	1.81497666976187	-2.29402144412346	-1.20094534812700
H	3.88173875144139	-1.04293519539001	-1.47809860119158
H	5.87578560865857	0.44562842127663	-2.27282929603245
H	7.54991141193569	1.80741203251988	-3.50386576745001
H	5.73755061708619	0.74673608924660	-7.25502232339174
H	1.97339193242457	0.41290246590095	-6.45996014326161
H	3.29521387579145	0.51977656002588	-7.62341155216531
H	2.07965297955117	-0.96588505695756	-8.94332458474108
H	1.03047413749117	-1.44407439089986	-7.62376322174168
H	2.37255779791783	-3.28990549858295	-7.08377998218483
H	3.18956832688374	-3.04526783313366	-8.62082199376393
H	4.92939598796498	-1.71392430457539	-7.60476853305343
H	4.63019268391588	-2.88040243252090	-6.30330513408559
H	7.47745133013328	1.95401484913324	-5.96821366427423
C	0.48661612073923	-4.13425543524467	1.37507543426210
C	1.87695783451187	-3.91793273960918	1.40967235817848
C	-0.06562593197404	-4.86019664599396	0.31216625857903
C	0.71390040449455	-5.31350260594154	-0.74181006589695
C	2.08175268955878	-5.07361559528120	-0.71180608511043
C	2.66795910471592	-4.41555671351761	0.38664511033006
C	3.11920309511084	-5.36782331447505	-1.69111500086295
C	4.35179174835355	-4.95766424428102	-1.16038913485178
C	4.18361996697046	-4.40750015496367	0.24098885610790
C	3.03404675310111	-5.89234273473597	-2.97841655110988
C	4.20005307129198	-6.00548169544827	-3.73037738724290
C	5.42817389112136	-5.60420386630751	-3.20121995947797
C	5.51064347409823	-5.08129421749723	-1.90963014184708
C	4.83542888376460	-5.35118799029468	1.31064216974396

C	5.30619589536994	-4.41270186784506	2.42077511327388
C	5.88292452902745	-3.23636928698001	1.63101705701265
C	4.87106435265871	-3.02652811671036	0.48958945540851
H	2.31534203481205	-3.37582535366261	2.24097837367978
H	-1.13521322923142	-5.02924016218068	0.29591880630994
H	0.25342078339665	-5.82726130212361	-1.58238476001306
H	2.07596712603944	-6.19378816160559	-3.39504244663091
H	4.15561707426179	-6.40538016973262	-4.73952637535681
H	6.47078310732316	-4.76265955346435	-1.50908144604301
H	4.14126497038746	-6.13069575037302	1.63945376721095
H	5.70508681154593	-5.84486370485069	0.85894283183722
H	6.03177229364087	-4.88449771741455	3.09240696259244
H	4.45679522687916	-4.08043144157049	3.03163614240838
H	6.02543050609010	-2.33457326250031	2.23385610283457
H	6.86278986538783	-3.52073911205330	1.22432812351069
H	5.34853824387969	-2.66058419557439	-0.42424204472227
H	4.11753900336720	-2.28868375161849	0.77347803553238
H	6.32763036395785	-5.69500023029427	-3.80349143083358
C	-0.74649969285809	2.94331062307896	4.20881878233244
C	0.38942754876054	3.66315623799754	3.82063063861337
C	-2.02114055291007	3.47484856441486	3.98653304265780
C	-2.18607251834520	4.73015024356819	3.41127496562862
C	-1.05131110029196	5.44948150990086	3.04629759569425
C	0.23109685415339	4.90927922704133	3.23694456392762
C	-0.90713776600824	6.78524655187089	2.47325374396953
C	0.46385575593943	7.05442530694815	2.31815010885483
C	1.30322120425822	5.87373119593507	2.76531294066623
C	-1.86602214941448	7.72185451657853	2.09386921534084
C	-1.43991395101207	8.93965419863295	1.56689663482888
C	-0.07850619789475	9.21191216247160	1.41972460552884
C	0.88036254426429	8.26720889681265	1.79258349101004
C	2.19099174758378	5.27306407497556	1.61388369007014
C	3.64719478470182	5.52253471048837	2.02345914800816
C	3.59844123675199	5.44264200770656	3.54945922295983
C	2.33184984896800	6.23507808342074	3.88669662356548
H	1.37465496902101	3.23254139029493	3.96727300839707
H	-2.89154038524562	2.90101762626251	4.28639034557126
H	-3.18499914056877	5.13580739509913	3.27225901437726
H	-2.92641104297032	7.51125744152651	2.21545010542555
H	-2.17212865153686	9.68682004735606	1.27345546900761
H	1.93868537698668	8.48454179342392	1.66509662105291
H	2.01458193057332	4.19467293376608	1.55177879519287
H	1.93552200006545	5.70907203746203	0.64406293742120
H	3.96940700779256	6.52749918557247	1.71649038313157
H	4.32735210240261	4.79260115748884	1.57400406343273
H	3.49646862252798	4.39421931787814	3.85577820236003

H	4.49039048888859	5.84748550229074	4.04001626067415
H	2.55062055378849	7.30869817484700	3.83488018143164
H	1.92832806880016	6.02905769462314	4.88295855430349
H	0.23707602961628	10.16816054540030	1.01163358134296

II

H	4.23515135805451	1.12179670168609	0.67133616363309
O	4.79818757266709	1.29794818790896	-0.12704584265004
C	6.04459319714928	1.78053742923220	0.23224279468940
C	5.93459089011179	2.85277153648099	1.37301082999691
F	5.78188881649429	2.25603123661031	2.56693826491695
F	7.02318205567321	3.63971107016454	1.43703526058966
F	4.86529229271702	3.63456817617597	1.16320902338767
C	6.63708040440518	2.44197232792185	-1.05211746069445
F	7.95568003156307	2.66435256310930	-0.93589142044817
F	6.43840062952256	1.66480514631654	-2.12504667591801
F	6.03185459431417	3.62354528218869	-1.29360061047579
C	6.96565446015052	0.60035756922177	0.70151553513475
F	6.28279039300543	-0.20950666023687	1.52112516557914
F	7.36679559384988	-0.13725653368291	-0.35246552484247
F	8.06281358704112	1.03305014786141	1.34570346546381
H	-1.48086312218223	3.17717131633754	-3.78207375400665
O	-2.10365259863054	3.91991054005628	-3.95388089915058
C	-1.63446311057163	4.84149687601703	-4.86514091178040
C	-0.51602192044012	5.73468090445289	-4.21878391545867
F	-0.28840535456239	6.86897839091678	-4.89958751317045
F	-0.83879086184164	6.06000604358760	-2.96149355535458
F	0.64923022590639	5.04414328791984	-4.16571046280055
C	-2.86696969329083	5.73255943234419	-5.23430953719052
F	-2.61139538535444	6.48626668135274	-6.32030397778000
F	-3.94063958310695	4.98008753868301	-5.48679382323925
F	-3.17229346552689	6.55702155375232	-4.21638404793152
C	-1.07587340879729	4.14426799359105	-6.15514957218387
F	-0.34352028722738	3.06926798690949	-5.80384462659097
F	-2.06842548889952	3.71728575775255	-6.94752799398270
F	-0.28877805156174	4.96250406886254	-6.87767526239102
C	2.45157990371300	1.54914577896969	-2.06152763227429
H	3.03934256792992	-0.29667363677714	-1.33623916681784
H	1.27058430310392	-0.16839221065247	-1.43278720694326
C	2.19816730280168	0.11461908025808	-1.92008890097934
C	1.79530840155727	2.52598139802961	-1.27366225217971
C	0.85875634927575	2.14085713888642	-0.27849286092372
C	2.02404468300740	3.91152866791285	-1.48740373283359
C	1.30220551558320	4.85196479824967	-0.78794199282147
C	0.36489467718324	4.44405426109282	0.16715118943275
C	0.17466757799361	3.09123199726138	0.44906942635724

H	0.66102674015191	1.09434044662585	-0.08774061578881
H	2.75080740799109	4.23757084008688	-2.22210053101190
H	1.45282141838411	5.91056318878370	-0.97324109615287
H	-0.21086767793187	5.19402322569396	0.70062117477922
H	-0.51787278984535	2.78649528485647	1.22783056113701
C	3.40436409014542	1.89422825370395	-3.13736403446726
H	3.99365567181942	2.78788841741534	-2.92207107165035
H	4.09125492803084	1.05094219806729	-3.27878666786106
C	2.60933565786292	2.10261567422937	-4.45872949559330
H	2.04355015128938	3.03726411174413	-4.39033119033409
H	1.86499116392357	1.30475335325234	-4.57476688530658
C	3.54865163132002	2.12381402767073	-5.66210114866398
H	4.31746403543829	2.89412195863110	-5.51179812199445
H	4.07874480633395	1.16358370840797	-5.71638739692723
C	2.79620899283602	2.38685997982962	-6.96381801395244
H	2.31336728621977	3.36980017625510	-6.95071268967452
H	2.01392094713505	1.63664932691787	-7.12572966605700
H	3.47559271221667	2.35707888224140	-7.82185738440433
O	-3.25581840122405	-1.27735463682265	-0.53134211265052
O	-1.23295748538767	-2.80232747739887	-0.52677221909932
O	-0.27823882019892	-2.12784811219026	3.04515167658724
O	-1.64674265661617	0.04986177116316	3.26900561783287
P	-0.52258356868177	-0.67136298391197	2.36350983444598
P	-1.64593797388426	-1.24311256255688	-0.43421698535233
S	2.27763298298711	-0.22252892264210	2.12888289876901
S	-1.80046008765323	0.57807385469048	-2.68438021313323
O	2.37071730113480	-1.23541570206051	1.05814175123000
O	3.09417864095587	1.00888009570841	1.95704759197599
O	-3.12951742885951	1.07051404089275	-2.29270774618947
O	-0.79957992721987	1.58018752950578	-3.13827686841901
N	-1.08911203351529	-0.44826458940525	-1.71271436797736
N	0.83007948433330	0.17541738922493	2.61359967054087
N	-1.09875965143810	-0.68391248622733	0.91572401337382
H	2.26876716678726	-0.37938530007093	-2.89458566233421
C	3.10244448200569	-1.09963705292077	3.62887890837032
F	2.68271299750328	-2.38068153779209	3.62970765675666
F	4.43540414663470	-1.07521419676739	3.40973310961543
C	2.80106099368213	-0.48354733697231	5.02432025835973
F	1.55828229866522	-0.85628517934077	5.39968092501974
F	2.85631982787508	0.86521181213659	4.94303407556461
C	3.80680430665535	-0.94019182037237	6.13783419800663
F	4.09848533100161	-2.25324633508000	5.97732356049332
F	4.94291359590731	-0.22115636211617	6.02877458653565
C	3.26082702439196	-0.76018889389417	7.59200333887727
F	2.31602388893897	-1.66329185113346	7.86285186641126
F	4.26947083744781	-0.92540377631987	8.46029922640338

F	2.75601984199322	0.47326687565338	7.75209723979619
C	-2.09601202448120	-0.50240723556192	-4.23970001584650
F	-0.89676226582961	-0.69151160280393	-4.84132973865911
F	-2.57656844989264	-1.69337980827451	-3.84095626278433
C	-3.09803724728516	0.12392449689634	-5.25596597317699
F	-2.88284256992483	1.45478183008172	-5.33363179761290
F	-4.35593949257755	-0.09321296116163	-4.81539249480944
C	-2.96333489093733	-0.46973883161075	-6.69898139487442
F	-1.86756343005180	0.05649859709490	-7.28980557843153
F	-2.81875078515052	-1.81524405350707	-6.62171049067586
C	-4.18380173715985	-0.18270380959920	-7.63160790988635
F	-4.49222003413092	1.12023538856798	-7.61211092105580
F	-3.85896589420990	-0.53020624980688	-8.88652238306330
F	-5.25259142183842	-0.89422837768530	-7.26002581805993
C	-1.77801560742194	-3.55481278466049	-1.57300606062340
C	-3.99681610469735	-2.24754136920854	0.13943077684452
C	-0.91043577620705	-3.96342716251004	-2.62011398638266
C	-4.82145505644968	-1.83204008940604	1.22064067880157
C	-5.61500676157912	-5.35620434998514	-3.90387430019220
C	-5.10206722271827	-4.70560961796105	-2.80782030979584
C	-3.70497328294345	-4.53582002797796	-2.65343305674885
C	-2.84600114017551	-5.00836307352437	-3.69024425812663
C	-3.40397541428231	-5.70085670783418	-4.79317784220142
C	-4.76031442729506	-5.87711017439129	-4.89732851125529
H	-6.69047726413824	-5.46298936942570	-4.00968265083299
H	-5.77231883472692	-4.30299827025040	-2.05641036500228
C	-3.12220719385935	-3.87729255071701	-1.52455914908104
C	-1.46643175346770	-4.73679215777782	-3.62139953632584
H	-2.73647070295879	-6.06774614885161	-5.56882279316679
H	-0.82413894303193	-5.12244697049363	-4.40864770364157
C	-6.33783589257052	-6.44214374754747	1.65004532042670
C	-6.40449901875502	-5.12137540883712	2.01438823415007
C	-5.58775424231570	-4.15041230386670	1.38442182873923
C	-4.72290269388538	-4.54692780755945	0.32054401488356
C	-4.65682705791572	-5.92345660139338	-0.01037421062810
C	-5.43870745800434	-6.84644805846297	0.64186417845227
H	-6.30409795251314	-2.52625153194511	2.59031153666164
H	-7.07263030531029	-4.79816995027401	2.80818638648088
C	-5.61641400120639	-2.80510701023568	1.79699105327735
C	-3.94992139830911	-3.54128334488396	-0.34360682009769
H	-3.97777548135267	-6.25245234124473	-0.78933686532416
H	-5.36580723608097	-7.89616293654163	0.37320596703546
H	-5.18031856022950	-6.39694698305861	-5.75322451668483
H	-6.96580851484645	-7.17899394227590	2.14258499401143
C	-1.34594929625665	-2.87369534611515	3.54845027777897
C	-1.42078574851364	0.04042405143704	4.65035528033030

C	-1.01108350487227	1.24118921110360	5.27645338559208
C	-1.58465612949060	-4.16036206618742	2.99540252465074
C	-5.27719780664430	-3.18942995437183	6.34740274723793
C	-4.14536924350296	-2.50205438579079	5.98004211642771
C	-3.24971929999368	-3.04222360059908	5.02518129129275
C	-3.55797882977776	-4.30352721764926	4.43371294781274
C	-4.71414269806002	-5.00090122360599	4.85962314631858
C	-5.55843022748635	-4.45677191738140	5.79480888318513
H	-5.95926130386847	-2.75485097253622	7.07236410188310
H	-3.93340376729302	-1.52861815784329	6.41094368031552
C	-2.07018119112642	-2.35732132928679	4.60693413654556
C	-2.69261188579858	-4.84280480987884	3.46078244025686
H	-4.92894193627110	-5.96768236662785	4.41326519090953
H	-2.89585626437759	-5.83997883251550	3.07819566015529
C	-0.58000773454995	-1.22065481774880	9.44737029727941
C	-0.51068476862453	-0.04663122224052	8.74109968982615
C	-0.83320688291645	-0.00839980373391	7.36338351766051
C	-1.26853531140985	-1.20428499302283	6.71248158811193
C	-1.29587462877312	-2.40521922684501	7.46262812537678
C	-0.95986645687733	-2.41087293132373	8.79505553087764
H	-0.42499871578545	2.10066840065841	7.13152529869915
H	-0.19382073378939	0.87331580675190	9.22592003599445
H	-1.57862625122091	-3.33114654617982	6.97408011493598
H	-0.98262735300035	-3.34428369169893	9.34982037174012
C	-0.72859841955936	1.18865285243207	6.62289287082793
C	-1.60327490637177	-1.14760430301009	5.32253538964162
H	-6.44684108327466	-4.99690910440763	6.10939597608441
H	-0.32457138269690	-1.24007307583983	10.50269487687679
C	-4.89702805001003	-0.43924961781546	1.70788589683472
C	-4.81213320085153	0.66534916197846	0.84343190260839
C	-5.13278903270481	-0.21773683180601	3.07771380464772
C	-5.33664472248408	1.05834086748968	3.58147451912144
C	-5.27837369180516	2.14294804627615	2.70850087748337
C	-4.98679230578699	1.93930060393528	1.35154739705797
C	-5.44315340938511	3.57420167571315	2.93459404533349
C	-5.22366289327863	4.24701736298264	1.71506306007235
C	-4.82246065495220	3.26014670518178	0.63397580320637
C	-5.75171601453909	4.27774224028035	4.09580320288475
C	-5.85515539672439	5.66474965767267	4.03245916129929
C	-5.66898335224163	6.33251093039904	2.82220889503757
C	-5.35545452250038	5.62754198653456	1.65659678842368
C	-5.55379573256024	3.43213451886218	-0.71562559880814
C	-4.73302490946523	4.47068927442886	-1.52253651729460
C	-3.36819630296321	4.60694489339362	-0.79966166261437
C	-3.33855889662819	3.46133904361717	0.21164147291610
H	-4.62329917211748	0.52581745243332	-0.21484247885462

H	-5.13883991873179	-1.06318281448476	3.75972903221296
H	-5.52418093412231	1.19857627565044	4.64340176427382
H	-5.90953210054422	3.75740535380220	5.03702172719328
H	-6.09248698899255	6.23051375462771	4.92863397798487
H	-5.22855017042294	6.16440291527757	0.72142219020438
H	-5.54241462360544	2.46872687579373	-1.23587761216285
H	-6.59983077363514	3.72229388353214	-0.57444199116034
H	-5.24883490271677	5.43548532374649	-1.57687880448768
H	-4.59363505594612	4.12700551292719	-2.55161237081874
H	-2.53006548398603	4.54832888906719	-1.49897521195440
H	-3.29006132318262	5.57222073598821	-0.28526106197121
H	-2.69685572689999	3.64235791488203	1.07817994580443
H	-3.00727899225856	2.53899880497177	-0.27769226735421
H	-5.77336424015728	7.41324535041013	2.78406591002000
C	0.50481017879333	-3.55568503788433	-2.72376844965033
C	0.99842642494612	-3.25592675559098	-4.00792256564742
C	1.36955473172004	-3.46839782737060	-1.62220851259249
C	2.69557453258509	-3.08884438666750	-1.78868622719173
C	3.16751061482586	-2.78934942298768	-3.06249230805301
C	2.31987734239704	-2.89195239043254	-4.17996422596381
C	4.43840607894428	-2.21763032818268	-3.49653512712994
C	4.37296325594392	-1.99508803443647	-4.88384176049952
C	3.05278981193226	-2.47819095051821	-5.44517008060820
C	5.56878945128743	-1.85014059120151	-2.76929276679988
C	6.63905993738232	-1.27101019778383	-3.44661790252799
C	6.57963519374582	-1.06003061573356	-4.82457878636720
C	5.44481138115878	-1.42492389818438	-5.55207048662545
C	2.27841970757666	-1.45771928031011	-6.31189152152855
C	1.34470810788710	-2.28913697636189	-7.22248442911392
C	1.88809479335314	-3.74239860369459	-7.18235292253375
C	3.22675973676435	-3.65764149030908	-6.43924406647763
H	0.30827817196078	-3.25817531287197	-4.84475116130161
H	1.00652923698530	-3.69386927789760	-0.62697144877337
H	3.33446802440503	-2.98836050366267	-0.91683145047526
H	5.61843916020502	-2.00717803512075	-1.69472107585062
H	7.52751601986065	-0.97598356518105	-2.89734185049526
H	5.41010979822613	-1.25575227149636	-6.62636067553262
H	1.73667146422032	-0.73146078458587	-5.69835665826872
H	3.00135911829220	-0.90195972040013	-6.92068817295804
H	1.34172124962996	-1.88690869806724	-8.24033692912638
H	0.30901919622646	-2.24613797237021	-6.87139867148080
H	1.19836514860704	-4.39712659364273	-6.63844066817662
H	2.00680535104845	-4.17151065800633	-8.18216942978276
H	4.03315025505970	-3.39492790181866	-7.13574920578212
H	3.51069864400896	-4.58463653073977	-5.93109437897329
H	7.42365402381949	-0.60503665053781	-5.33477959476394

C	-0.68195898033842	-4.81886484150574	2.02573293681822
C	0.71351854102387	-4.83255359561027	2.20044811762310
C	-1.24667532983553	-5.54851216162473	0.97197120488102
C	-0.46050821433307	-6.25949558257524	0.07716467556845
C	0.91748187989683	-6.27242224287206	0.26134364251279
C	1.50060241028005	-5.57712857389175	1.33729983767767
C	1.97582190570848	-6.90294982587069	-0.51790337808619
C	3.20147323936014	-6.64084260774498	0.11331312806930
C	3.00078037751907	-5.83030163501426	1.37775700951365
C	1.92605679268257	-7.62697455052561	-1.70656564499568
C	3.11825716973899	-8.08837874408096	-2.25705054291031
C	4.33781914694465	-7.83115005229353	-1.62847574064095
C	4.38486868043517	-7.10712341405625	-0.43614118463704
C	3.40061446799870	-6.64049063270724	2.65845242039610
C	3.96441410171831	-5.59961967175311	3.62628262491530
C	4.77926144910432	-4.69738194340604	2.69789987322795
C	3.88311311611946	-4.53873751882618	1.45720505322566
H	1.15547257881720	-4.28185235970132	3.02446597858930
H	-2.32227106191388	-5.52681598865254	0.84260098402704
H	-0.92135824516469	-6.78466349471727	-0.75592149949923
H	0.97839552121374	-7.82161664364157	-2.20220300750113
H	3.10180747685602	-8.65074195891877	-3.18625069079124
H	5.34081257606542	-6.90669305922277	0.04345385554404
H	2.55627429879171	-7.21376820470942	3.05284459662590
H	4.19193212466867	-7.35072768033781	2.38892913531156
H	4.56002407916748	-6.04902307886480	4.42833878888622
H	3.15452317237703	-5.02815971146253	4.09709137220714
H	5.04981995100185	-3.73671796746563	3.14629444335720
H	5.71272572138036	-5.20906151610234	2.42748842358092
H	4.46811606577215	-4.41349650195924	0.54125854330020
H	3.23851638438698	-3.66075479124718	1.55419306076549
H	5.25904678232716	-8.19502225309560	-2.07429531387845
C	-0.94581760504326	2.53794835297063	4.56397237956913
C	0.26417992874047	3.24194187047574	4.47959157941685
C	-2.12819629360832	3.11178698171410	4.08450328400060
C	-2.12747698030344	4.38982938211560	3.53940402947643
C	-0.92361815039002	5.07967093688641	3.45374285352645
C	0.27440963473758	4.51216688135801	3.92390874901809
C	-0.62599332567866	6.37374905299506	2.85123464172900
C	0.75926207887215	6.59233312635748	2.93823557448315
C	1.43480645374095	5.46830292111102	3.69698219746157
C	-1.45647575350725	7.27978860051361	2.19509575813378
C	-0.88329896602098	8.41318692670603	1.62328175121993
C	0.49244908542753	8.63488597528923	1.71013623531291
C	1.32121099193291	7.72644952572752	2.37261736513332
C	2.64898641268066	4.81660944121686	2.99068283529508

C	3.56169330733982	4.26293161461379	4.11253021031282
C	3.02887735595092	4.85103911389306	5.44260864792351
C	2.06123282118122	5.96363903898126	5.02928967011560
H	1.17459368830928	2.76244539152598	4.82330447295588
H	-3.05750506155700	2.55574191639367	4.15794490741656
H	-3.05450682297851	4.83750198042596	3.19338502478647
H	-2.52686673953877	7.09971492446672	2.12237576960287
H	-1.51039789006454	9.13058941483988	1.10176590766683
H	2.39173406021763	7.91035062132291	2.43186274163275
H	2.33598599579286	4.04033258119746	2.28675184263658
H	3.17446352631270	5.58652899578242	2.41436105918933
H	4.60022731784621	4.56212304732777	3.93914618646554
H	3.55156526405965	3.17009820011890	4.12347237695985
H	2.50018748499364	4.08602506220124	6.02163416238516
H	3.83178004374384	5.22944031812455	6.08349586266441
H	2.61464882178526	6.88824141190219	4.81948392436629
H	1.29895603149080	6.19354534811517	5.78091889262641
H	0.92228091585967	9.52331784336199	1.25643748133037

II’

C	2.06060581780022	2.94806836734194	-2.77750372137215
H	2.90503431932999	1.07975701086532	-3.36391734868945
H	2.86566347230337	1.37048066899847	-1.60853649153623
C	2.32195356160007	1.51953831776617	-2.55507173677387
C	1.25498581607124	3.69960927849558	-1.89127156680845
C	0.69771742953776	3.08013918447072	-0.74200428704882
C	0.90960901666058	5.04920909987457	-2.16938380308275
C	0.03241342953607	5.72406609492954	-1.35227015207691
C	-0.50712689084743	5.08544473300175	-0.23017501957760
C	-0.15913938017558	3.77248679197569	0.08534340772572
H	0.93197768798253	2.04882930171831	-0.51813288108575
H	1.31303896796598	5.54987497608550	-3.04159274712661
H	-0.24576340258787	6.75063463607304	-1.56858403472272
H	-1.19885945995928	5.62883009036686	0.40413074798434
H	-0.56326972555018	3.29291199022815	0.97200969907951
C	2.61532124721901	3.51861959346456	-4.02418396128200
H	2.78822752107005	4.59606363392133	-3.97392509941160
H	3.56859987074175	3.02271548710079	-4.24411910089947
C	1.62866080392496	3.20933758639510	-5.18991558962746
H	0.70159150827357	3.77007609327958	-5.02738304985691
H	1.34522551326091	2.15068061729531	-5.15770589044339
C	2.24886600282345	3.54935132548479	-6.54319612297438
H	2.51738051096899	4.61443014230676	-6.56614688494181
H	3.18521787500072	2.98784089230480	-6.66321106978145
C	1.29918623668340	3.22513284050712	-7.69430148257056
H	0.36132435744118	3.78344904787168	-7.60384987592494

H	1.04818327693929	2.15877843912616	-7.70746557305209
H	1.75088503668223	3.47719579078319	-8.65899450671520
O	-2.81151777404864	-0.84219033878617	-0.92114719801672
O	-0.56529619257095	-1.99002358058590	-1.09702121605793
O	0.50988661087350	-1.39994407603587	2.52159163306133
O	-1.17576340107305	0.49781749468538	2.97467135061272
P	-0.03271143209715	0.02053531305696	1.93566989832530
P	-1.22752446742246	-0.53461526444474	-0.84047646658021
S	2.62147675959737	0.89348231368071	1.45613841078569
S	-1.78451355172507	1.34106597906969	-2.99312825634434
O	2.71222690341254	-0.14839790741975	0.40792646244241
O	3.22724071708128	2.20361845869570	1.17399573704363
O	-3.18393394641135	1.49433707506343	-2.57199834036721
O	-1.04155359386509	2.53414776843850	-3.43550896634234
N	-0.85353854795302	0.47110401598100	-2.03264339698730
N	1.17803766059168	1.07164276563687	2.10190718030786
N	-0.77948968188893	-0.06343145050056	0.57210520237879
H	1.38401263490845	0.95745328024483	-2.42657591220908
C	3.70508733829224	0.16795100927601	2.86817984981422
F	3.43925881168834	-1.15115617796349	2.97300010188803
F	4.99548035203997	0.32323303435422	2.48818032331273
C	3.50264672634359	0.81929137068269	4.26441043663907
F	2.34408389257856	0.36332896409229	4.79036210789199
F	3.43013461862746	2.16419625070452	4.12481885108478
C	4.65322676605186	0.51000212623911	5.28290737179825
F	5.04346745495275	-0.78070250727486	5.15780626554345
F	5.70552473103158	1.31246345304229	5.01741870178990
C	4.24746408661371	0.72913541167441	6.77769007708043
F	3.43218172060531	-0.23853859833983	7.20244796704144
F	5.35451410807284	0.71606623914276	7.53578057381992
F	3.63993924982041	1.91720935852880	6.93006170842558
C	-1.81960545144590	0.24540419415069	-4.55818123797148
F	-0.54648270817472	-0.13462355377144	-4.81563535789678
F	-2.56427818518827	-0.84994152131224	-4.29127661889488
C	-2.38357888723455	0.94485564798193	-5.82771926505256
F	-1.45464367179297	1.81654789362353	-6.29492776218889
F	-3.50241246938014	1.62795276944223	-5.51160160781462
C	-2.73592138684263	-0.05816579627796	-6.98257327336280
F	-1.78321906639314	-1.02109499772339	-7.06368962088107
F	-3.92243900723099	-0.63945349551019	-6.71715965807927
C	-2.83625326132826	0.60312007539764	-8.39536611602822
F	-1.62667241792734	0.95760503100703	-8.84952889782458
F	-3.36483284990550	-0.28083203849511	-9.25406052309313
F	-3.62282568683432	1.68681613041816	-8.35442759264490
C	-1.01162487674686	-2.72500055921362	-2.19567640838927
C	-3.32452630621292	-1.97465131849322	-0.29719523898436

C	-0.12376015485543	-2.90474695335423	-3.29300549488601
C	-4.11040419611501	-1.80435362081724	0.87546343135277
C	-4.61235322762384	-4.90809115938250	-4.57731288333841
C	-4.15710108454607	-4.30316156178264	-3.43068895171398
C	-2.80292849854196	-3.90933076057469	-3.30840574483227
C	-1.93124398091517	-4.11722694366520	-4.41861023257044
C	-2.42535041742851	-4.76724280526922	-5.57655836017024
C	-3.73792562471418	-5.15807370449563	-5.65539766035262
H	-5.65829269075952	-5.18918814524859	-4.65721606009809
H	-4.84169112886045	-4.10810569271480	-2.61232701796499
C	-2.28194214583302	-3.27289442879272	-2.14014220595149
C	-0.60576725080031	-3.64874194347685	-4.35530662555743
H	-1.74805843820587	-4.92894072670692	-6.41150566762692
H	0.05324441672851	-3.87060583785392	-5.18966651995114
C	-4.73965945848810	-6.63305246388177	0.98163636979191
C	-5.00295011935430	-5.38021498353135	1.47496738269996
C	-4.43578677316067	-4.22820193125315	0.87705738282661
C	-3.61904585712165	-4.37334601118225	-0.28542899021219
C	-3.34475466238824	-5.68299675016838	-0.75182240349296
C	-3.88691646141335	-6.78301226768279	-0.13133029886341
H	-5.30701703344691	-2.86474941561218	2.28953958540698
H	-5.63754391202498	-5.24871980973107	2.34736313914071
C	-4.66190948084246	-2.94838347850828	1.41926260744203
C	-3.08777796951677	-3.19452946531112	-0.89885115531822
H	-2.69344813237830	-5.81735437322982	-1.60863704326517
H	-3.65685532111541	-7.77666024744703	-0.50439670548162
H	-4.11144020012747	-5.64268947302970	-6.55257560344112
H	-5.17645125841152	-7.50993782589013	1.45083951859475
C	-0.38294377185991	-2.34226923634028	3.02636184357525
C	-0.85173270035738	0.44288728292302	4.33006539462589
C	-0.61848267279337	1.65905488309467	5.01259954320586
C	-0.45918188315138	-3.60861893146586	2.38604527039791
C	-3.99998057180631	-3.48090017708294	6.02760379203624
C	-3.01482059054676	-2.59933527552743	5.65171196671727
C	-2.12145424038955	-2.91776315350144	4.59992407651293
C	-2.27949011538432	-4.16359438330944	3.92082021001219
C	-3.28396490649939	-5.06226278986429	4.35476221545758
C	-4.12905085872742	-4.72971497777421	5.38407542144342
H	-4.68338215994272	-3.21522713610279	6.82892196832068
H	-2.91966783270802	-1.64165043312207	6.15377944144720
C	-1.09856345819860	-2.02351575591594	4.16544681631938
C	-1.41493642014874	-4.48860967927218	2.85554608900413
H	-3.38083995860963	-6.01523577373205	3.84204942578453
H	-1.49235594122049	-5.47434538888978	2.40314930134578
C	0.54428567744130	-0.91485731476523	8.96664151987795
C	0.34549172148576	0.29107663846544	8.34318362367106

C	-0.08499127807812	0.34982606591203	6.99622799288404
C	-0.34580766885824	-0.86673096434141	6.29108637549710
C	-0.10537578013743	-2.09379588733020	6.95610860279888
C	0.32936346437433	-2.11511949118212	8.25944975387868
H	-0.06636511443328	2.50870429733790	6.88330097578387
H	0.53214622155440	1.22374985203360	8.86962372503545
H	-0.25855060622201	-3.02519221659444	6.42196209021110
H	0.51494773251456	-3.06735002315360	8.74758630743218
C	-0.24755636186046	1.58721731437469	6.33503465156376
C	-0.78420015631277	-0.79491839636577	4.93145670331760
H	-4.90047454909180	-5.42440960227467	5.70378376292997
H	0.88309195085218	-0.94804970001666	9.99787779085554
C	-4.37993064853423	-0.48707576646893	1.48681557070431
C	-4.57088960214621	0.66576193233877	0.70776022611103
C	-4.51182498776680	-0.39625372807348	2.88451391266660
C	-4.86630935553764	0.79425283241670	3.50344932810681
C	-5.07170343721291	1.92651503902513	2.71700741687591
C	-4.89605023512100	1.85662988535833	1.32789149451572
C	-5.43683800762916	3.29594386307742	3.06437770982026
C	-5.45295258118565	4.06892299177072	1.88483914721740
C	-5.03768530983349	3.22185730917443	0.69558210847037
C	-5.72759631664992	3.86923141534261	4.29951003135966
C	-6.04233713944101	5.22456311904338	4.35407866733077
C	-6.07494100908894	5.98970141472290	3.18868457534165
C	-5.78207325443833	5.41583626300286	1.94795350151506
C	-5.96695401039870	3.31538514342497	-0.53423479838305
C	-5.48234984586819	4.53979337238048	-1.34311704972170
C	-4.03706375099595	4.83681355908134	-0.85938012705882
C	-3.68255783304793	3.69583592249619	0.10214101394080
H	-4.47055645647987	0.62374771916077	-0.37090514223714
H	-4.31396292739153	-1.27253657708228	3.49537997168741
H	-4.96611757855774	0.83298218602929	4.58559568448182
H	-5.70837094958948	3.27297504310118	5.20829406604355
H	-6.26896280896981	5.68877708402653	5.30949754201296
H	-5.82038728283731	6.02823141062069	1.05209040407206
H	-5.83276372320315	2.40523967543343	-1.12852954942580
H	-7.02121681662851	3.37518699012788	-0.24595979175498
H	-6.13426759606068	5.40479549855019	-1.18023255651905
H	-5.50806769799590	4.32444837144656	-2.41538532083329
H	-3.32695729036117	4.88161070250742	-1.69193450872245
H	-3.99050530235425	5.80447053468628	-0.34493807113383
H	-2.98141621405568	3.97798954288037	0.89303669404996
H	-3.24980533180907	2.86027491822117	-0.45788647773009
H	-6.33442419001124	7.04316022474544	3.24507206702204
C	1.24362175577789	-2.34667100517476	-3.35568813771228
C	1.77075641217042	-2.02047417822115	-4.62260012907020

C	2.04984177431967	-2.15889785073957	-2.22232071475855
C	3.34254246880621	-1.66474178961205	-2.33725626253061
C	3.84843249729588	-1.35816074029874	-3.59330576108354
C	3.06300687542795	-1.55129567544409	-4.74386309785850
C	5.09910549047491	-0.71914401344344	-3.98504132177323
C	5.08617121996010	-0.53517585248618	-5.37893721700599
C	3.82182270234069	-1.10967762300859	-5.98477065512194
C	6.17274735979517	-0.27310242683060	-3.21721143591572
C	7.23832350209087	0.35244410789778	-3.85908958141850
C	7.23043604536226	0.52925807879214	-5.24357961247966
C	6.15266526478368	0.08342674356115	-6.01195072685726
C	3.01913498669698	-0.14592545586195	-6.88921097347254
C	2.17960867934658	-1.03962643458361	-7.83209516318383
C	2.80699720903457	-2.45728076226593	-7.75291067747341
C	4.10602275313774	-2.28540979219185	-6.95564714441665
H	1.12765164110030	-2.09219987691442	-5.49234843916282
H	1.67216763834707	-2.39402579797566	-1.23722685848217
H	3.92421569498895	-1.48874456476992	-1.43948319807367
H	6.17719938157992	-0.40480426549917	-2.13818296712001
H	8.08390024016143	0.70829465866693	-3.27743790159780
H	6.15839552530902	0.22637341559845	-7.09053572388044
H	2.40440360596487	0.53906216133195	-6.29762595667747
H	3.72582662286063	0.46010259500618	-7.46836042887407
H	2.19870291619213	-0.64730179673859	-8.85374919159104
H	1.12787514684576	-1.05686795209949	-7.52751026259721
H	2.13552853565012	-3.14692686512833	-7.23004109738379
H	2.99224305398432	-2.88723391888988	-8.74220112643924
H	4.92470617424256	-1.98515737488049	-7.62225715796084
H	4.42004845359217	-3.18868056835660	-6.42273230319168
H	8.07025051309493	1.01944688908257	-5.72755260021519
C	0.47253791121821	-4.03934113947597	1.32019978524929
C	1.86131878855652	-3.84291782342021	1.43246233226273
C	-0.03146413435972	-4.75829595268417	0.22918029906827
C	0.80378358891471	-5.24362860951366	-0.76718979199845
C	2.17417769609137	-5.04193343038831	-0.64981267457713
C	2.70416930254653	-4.36943281160947	0.46780517684238
C	3.26686554527490	-5.38227905006413	-1.55231713991814
C	4.47146527982874	-4.97840782397589	-0.95592037030063
C	4.22587407627656	-4.38486386612542	0.41600504503354
C	3.25601965502199	-5.94546612383075	-2.82586572415496
C	4.46561607029720	-6.10070858720476	-3.49733583846304
C	5.66465636779466	-5.70291962359629	-2.90360152307007
C	5.67337099260424	-5.14285996952974	-1.62501384228280
C	4.79942730571801	-5.30018923213727	1.55170500932797
C	5.19685574498357	-4.33246394700802	2.66562100446168
C	5.83972310672202	-3.18480857735673	1.88446291635679

C	4.91761336359122	-3.00413929842494	0.66427687313594
H	2.25697820966121	-3.29738653667497	2.28328678402593
H	-1.10271290742691	-4.90283313317960	0.14724593876664
H	0.38357748126552	-5.75488044138771	-1.62996682366938
H	2.32284199195790	-6.24650058673237	-3.29511126371534
H	4.47768346882441	-6.53086624898622	-4.49481740297772
H	6.61189857525412	-4.82869969840960	-1.17263442111142
H	4.08099695781246	-6.06893079497009	1.85184856534426
H	5.69658941200397	-5.80598768089881	1.17317888593438
H	5.86751400845816	-4.78753716226418	3.40257128517129
H	4.30860138839868	-3.97401295496217	3.20168765167300
H	5.94807478512293	-2.26622941792711	2.46891918326460
H	6.84335198829367	-3.48914775342489	1.55836079854552
H	5.47122710722013	-2.68406182975906	-0.22360253228685
H	4.15489348450606	-2.24635754526149	0.86070494228847
H	6.59874347483993	-5.82544179781027	-3.44426152821982
C	-0.78738443096458	2.97408387733248	4.34928706684494
C	0.32767257392346	3.77886666120999	4.08769831327793
C	-2.07232664248300	3.43204949887199	4.03842999970575
C	-2.26742569513093	4.69532475163030	3.49189380825107
C	-1.15341716369448	5.48781064435857	3.23116254349059
C	0.14206687715808	5.02643386819505	3.51708525001317
C	-1.04697374098960	6.79892241769808	2.59830397332015
C	0.31613689480326	7.12194388299276	2.47911214966465
C	1.18553186277789	6.04785339781836	3.10255330904670
C	-2.02975781419202	7.64033861687655	2.08126975877820
C	-1.63384163166482	8.81028915907326	1.43617561563082
C	-0.28043269859508	9.13138625177320	1.31320650216578
C	0.70196128721951	8.28915273444132	1.83925290768286
C	2.27904355670627	5.44645674384262	2.15097045999870
C	3.63858799557761	5.74443634977640	2.80306617814918
C	3.31849134114917	5.81039962654100	4.29859692482937
C	2.00171244163080	6.58860763912124	4.32356149655115
H	1.32025959936451	3.39439815638995	4.29896356980419
H	-2.92564329272343	2.79111157777413	4.23526771482175
H	-3.27282969333746	5.04509595349973	3.27424707515202
H	-3.08342836404203	7.38442841026331	2.17207464889077
H	-2.38390236574785	9.47998786437472	1.02493151915299
H	1.75445998630241	8.54528516499019	1.73587867613530
H	2.13767587692962	4.36497087646889	2.07067945988321
H	2.19524666425072	5.85479704211939	1.13873960522646
H	4.02162193031910	6.71901188824502	2.47085525622007
H	4.38673785170155	4.98643884116379	2.54997185382735
H	3.17689877828574	4.79939316357964	4.70024155166978
H	4.10332555801415	6.29312826235928	4.89100282304465
H	2.20705036570587	7.65471491528553	4.16525597542754

H	1.43922536132982	6.49286515936416	5.25744656200115
H	0.01007099687319	10.04711426092617	0.80603501385675

III

H	-0.20939871817609	2.65805345932996	3.92005294817754
O	-0.70289102570180	3.51043055785750	3.82081545268771
C	-0.25579383271836	4.48181178729488	4.69867390185174
C	-0.85511487850772	4.22779250304439	6.12463894711969
F	-0.24037583002065	4.95320741266478	7.07474740337382
F	-2.16483239298088	4.53308278505025	6.14142675947707
F	-0.73814223407313	2.92997381611330	6.44095107505301
C	-0.77917761536970	5.83962707776267	4.12662893728250
F	-0.74981424335467	6.81087663749998	5.05357986643295
F	-0.03369772448402	6.23389912816618	3.08192495965734
F	-2.04534290650828	5.70953284361830	3.69565759500064
C	1.30829780883069	4.50114522836055	4.76560725853549
F	1.75910821771842	3.51932752581631	5.56769094998378
F	1.82086490619373	4.29086296730652	3.54367600043988
F	1.78438849155739	5.66932251332163	5.22925457301723
H	-1.69492912487341	-0.59075822378016	-5.33592229839517
O	-2.09290396432318	-0.92092505013607	-6.17391554316170
C	-1.12912389142978	-1.13149321292695	-7.14362568215358
C	-0.27246865617267	-2.39736627789455	-6.79134005850598
F	-0.99564933923447	-3.51955575300561	-6.95196921461992
F	0.83070507448376	-2.50216058454237	-7.55255308755418
F	0.11105657579972	-2.34731549357818	-5.50336856615092
C	-0.19301674953539	0.11916242388242	-7.30263385733089
F	0.69345183863443	0.17141883915719	-6.29355202079899
F	0.49155783684715	0.09812474698734	-8.46062441917660
F	-0.91573955338005	1.24775690216343	-7.26467998113287
C	-1.91601127925299	-1.37706218986218	-8.47239350810363
F	-1.12868644177816	-1.92633436318888	-9.41518629733962
F	-2.95268111861646	-2.19940652121710	-8.26534141199651
F	-2.40230378386919	-0.22193582916460	-8.95652272134978
O	-3.43659525720273	2.39384280793589	3.31561626459624
H	-2.85506258646318	1.72663720652469	3.72636676968837
H	-2.89046063354466	3.19169598431529	3.34690648426768
C	-3.37389177837506	1.78917582030692	0.96437007244764
H	-3.33992941766268	-0.05689410207511	2.02238848182980
H	-1.84428534936456	0.25117657898491	1.08610803534873
C	-2.91906152614672	0.39779827065413	1.11903260696838
C	-2.46994342610442	2.85798312484569	0.64229917025947
C	-2.95323882050835	4.12929636710130	0.24875817819894
C	-1.07615539814510	2.69171471575471	0.79501459084215
C	-0.21146728587040	3.75273000314515	0.60268600307242
C	-0.70872743790424	4.99880650306138	0.23811403964127

C	-2.08142682800101	5.17908615243795	0.04194658685172
H	-4.01533727222688	4.28622527363603	0.09778079609816
H	-0.66146923460526	1.73862866379963	1.09791536195112
H	0.85474223166425	3.60929541318721	0.74006375811759
H	-0.02463009719486	5.83391998005827	0.11023502089724
H	-2.46351308095215	6.14870092642287	-0.26232275800572
C	-4.84658862604925	1.96437875270410	0.89104915807748
H	-5.32659320817439	1.25769830608593	1.57680792050286
H	-5.14683596885109	2.97050363947628	1.19167856088260
C	-5.35604226059402	1.66880668342298	-0.54420992922420
H	-5.21672620549642	0.60244591609149	-0.76085331268407
H	-4.75765697748366	2.21085134096543	-1.28452235052694
C	-6.83170860765412	2.03514994229188	-0.68265287602176
H	-7.40099965477083	1.57015604919867	0.13249070703014
H	-6.95000018184670	3.12206228903504	-0.57304408831369
C	-7.39345729031310	1.58216543787644	-2.02748662187836
H	-7.32319519484629	0.49287728945987	-2.12694958916725
H	-6.83808074384257	2.02734587592867	-2.86092875500584
H	-8.44632065188347	1.86296771112514	-2.13353538296795
O	0.53509356496447	-1.63581417712583	-2.00593036375754
O	2.37132084933937	-0.06595702147653	-1.13320455019865
O	1.94549235082802	-2.16959763863758	2.16009222868329
O	-0.23711969332314	-3.02796731240140	1.16706790451476
P	0.48416004570759	-1.66838133550582	1.66206748602446
P	0.79307548618199	-0.37311113713119	-1.03155008784349
S	-0.38479693078106	-0.04853290082313	3.97384625329660
S	-1.24581414629004	0.82534340852411	-2.64312944273145
O	0.55019241726428	1.08510594198817	3.72540821292231
O	-1.78329269442246	0.32833935371507	4.28116388765377
O	-1.06025445523238	-0.09198871670819	-3.79421277486107
O	-2.56835730184707	0.81987125525494	-1.99334741242248
N	-0.02965561963980	0.86178744172681	-1.63248580235830
N	-0.32359340627947	-1.24087087288106	2.97654029932915
N	0.49865636846834	-0.62072429142647	0.49903890380449
H	-3.35275793195261	-0.16750005128009	0.28086858710989
C	0.12755623127503	-0.82051741969857	5.65592713157640
F	0.31267381739832	0.20458777531636	6.51499752025231
F	-0.90462301472635	-1.57438560775320	6.07750746463713
C	1.41278331474746	-1.69286122368924	5.61748748246912
F	2.33470651825538	-1.06149799971659	4.85265559274047
F	1.11668279599806	-2.88823170047954	5.06356726272887
C	2.04901923457042	-1.95012325259652	7.02675542270387
F	2.75417857699598	-0.86061837119440	7.39819196770342
F	1.07035701668168	-2.17138514973457	7.93404470968622
C	3.01418946478348	-3.18052016876452	7.06473412682165
F	3.86092517208440	-3.14197247600592	6.02323340031627

F	3.72799482837496	-3.14106153534622	8.19866258616839
F	2.33008975815237	-4.32622858761897	7.03272984773416
C	-1.07994544178062	2.58224692172338	-3.38392766417934
F	-0.82068314130157	3.45496899329026	-2.39480561555350
F	-0.04928490539793	2.58501414122712	-4.25546192217619
C	-2.38860650519047	3.01860983289135	-4.11865865450804
F	-3.33185981139538	3.31122082114501	-3.19014382973138
F	-2.82379933007161	1.99677693752890	-4.88732457747738
C	-2.22253023677482	4.27751048423215	-5.03280180021420
F	-1.54098089022110	5.23467331788301	-4.35561662562846
F	-1.52793222160912	3.94161117261213	-6.13723261743754
C	-3.57294255734347	4.91867825694704	-5.49612938051051
F	-4.18680870824302	5.53495904388130	-4.47776870134459
F	-3.31373918937895	5.82943511731943	-6.44492056012377
F	-4.39270528688816	3.98951198724057	-6.00195778158212
C	2.85319119449604	0.19380722116138	-2.42318434913670
C	1.46399136651841	-2.61549040465033	-2.32949691955791
C	3.17060314199550	1.53425996219921	-2.76529909644217
C	1.02579208897571	-3.96405058949522	-2.20972840168172
C	3.75057453802685	-1.30348707968273	-6.93651698140767
C	3.37578168667314	-1.60793456526132	-5.65043718684120
C	3.36780870534940	-0.61522917472672	-4.64181553211399
C	3.71177752702482	0.72109500985348	-5.00860672052190
C	4.11147274848282	0.99825017376679	-6.33873513368071
C	4.13700465116403	0.00586268502286	-7.28491318330578
H	3.73184310093086	-2.07861193620685	-7.69654247782156
H	3.05932150655190	-2.61606588803721	-5.41034781229283
C	2.98616535379051	-0.87499168378185	-3.28449755610338
C	3.62816905297708	1.74941704911120	-4.05060294964163
H	4.37818634942210	2.01948748338320	-6.59882024466411
H	3.93474024289161	2.75065896513534	-4.34089113834918
C	5.50157411296326	-5.39374978330224	-3.47477514693016
C	4.21447218680796	-5.68746305852180	-3.10030784549994
C	3.27495488663235	-4.65731342496001	-2.85560465340060
C	3.66241053852922	-3.29825019889447	-3.05246547200563
C	5.00260445065434	-3.02821437094212	-3.42342959618425
C	5.90151437491831	-4.04946773052132	-3.61813526976939
H	1.64274385958553	-5.99354214814414	-2.41952491516152
H	3.89706373002452	-6.71789439990854	-2.96764602214770
C	1.95126566879770	-4.95247436535834	-2.47167184793905
C	2.69536035089280	-2.26224092966637	-2.85103347303793
H	5.31954389228302	-1.99835834623972	-3.55233428198896
H	6.92608557877880	-3.81772971455394	-3.89391979951481
H	4.43206683469034	0.22935195429720	-8.30574145818068
H	6.21498376338151	-6.19280794464528	-3.65495305753400
C	2.59298512128143	-3.28277230768912	1.62286711857488

C	-0.35995345474505	-3.97909705473755	2.19564728714559
C	-1.59696930661984	-4.07071408321224	2.88266345021660
C	3.86696814180250	-3.07680862243190	1.02729608489156
C	2.72178621040425	-7.99798220001221	0.52670146328426
C	2.06096054537096	-6.93903958628228	1.10183326387202
C	2.65252127951080	-5.65275949040616	1.14596144179052
C	3.93067516853161	-5.47008333885679	0.53797688705065
C	4.59910178408626	-6.58875846332422	-0.01529027477604
C	4.01152113741102	-7.82883893193612	-0.01836584755279
H	2.24888528233465	-8.97533004655518	0.49840217980676
H	1.07428026520544	-7.08387582551192	1.52987138855759
C	2.01125084541503	-4.53108937547688	1.75393353325891
C	4.50610303760623	-4.18458745443350	0.50666007809777
H	5.57974254671814	-6.43822125818577	-0.45789732718094
H	5.49428727381533	-4.06520548271383	0.06936096482013
C	0.52643806484319	-7.16098780103245	5.98545483315999
C	-0.58336115923944	-6.48641610188548	5.54177961054352
C	-0.51998053518118	-5.65923783628690	4.39449316226860
C	0.71145877575969	-5.55429201764082	3.67730688479807
C	1.84433051578738	-6.23794190990028	4.17951622440973
C	1.75161848550420	-7.02361054783642	5.30315770675419
H	-2.58447768576990	-5.04375267023034	4.49882807494345
H	-1.52817142715648	-6.56671865994701	6.07333652873010
H	2.79780243155210	-6.12950613100042	3.67483865380102
H	2.63521691333778	-7.53327266182229	5.67605869385599
C	-1.64601687150988	-4.92608312174636	3.96265247384401
C	0.76156396213517	-4.70417916956817	2.52698988565763
H	4.53137408509284	-8.67938806401696	-0.44990497830125
H	0.46757657208869	-7.78741832034374	6.87057456481692
C	-0.38872587861961	-4.32172738747088	-1.97144836306990
C	-1.41263773251377	-3.64332662840655	-2.65295670341143
C	-0.71309261589072	-5.42906186505274	-1.17143729539135
C	-2.02737948376632	-5.86178725348717	-1.03780169001739
C	-3.03614196604123	-5.17618278446431	-1.71336525868939
C	-2.71929419962200	-4.07469647382108	-2.52433587237684
C	-4.48759869594866	-5.32936462460583	-1.69761775000796
C	-5.05171612719856	-4.33553052150908	-2.52373614061852
C	-3.95955023077291	-3.51625082479370	-3.18860453151077
C	-5.29546788193557	-6.19590099575078	-0.96727552059315
C	-6.67808912735712	-6.06050328061373	-1.05707715860408
C	-7.24085982174838	-5.06978878707313	-1.86046434262229
C	-6.43108360082434	-4.20285133901898	-2.59866416757820
C	-3.93082568150768	-3.71223031810740	-4.73042505964248
C	-4.99877035785255	-2.75559258115586	-5.27174424211832
C	-5.02599473902257	-1.56563702705941	-4.27844684767536
C	-4.14367877420098	-1.98501987250687	-3.08133085198324

H	-1.17379643052801	-2.79900998702907	-3.29178634182332
H	0.08106865884387	-5.93982827835298	-0.63293569330249
H	-2.25868996904484	-6.70361079707051	-0.39021706941522
H	-4.85780104027715	-6.95118990497357	-0.31984727070763
H	-7.32390784262109	-6.72121808933062	-0.48615408484417
H	-6.88825774022329	-3.42942803206310	-3.20878794606740
H	-2.94117601131641	-3.40758198392081	-5.08923004069582
H	-4.08728546864924	-4.75781699264728	-5.01513346696875
H	-5.97305104325250	-3.25567809927908	-5.30569385724034
H	-4.76513668985814	-2.43592363496666	-6.29080967145929
H	-4.63362445351657	-0.65221912480917	-4.73639146153886
H	-6.05066635710430	-1.34165359993301	-3.96122427876496
H	-4.56096183036519	-1.68058834000038	-2.11613793686431
H	-3.16111660008996	-1.51353904051490	-3.15952754886670
H	-8.32112825768835	-4.96586846008701	-1.90783402625358
C	3.05327708081082	2.67869131108234	-1.83889425113048
C	2.65545290859934	3.92324017258355	-2.36293472634356
C	3.42192588062189	2.59295088679222	-0.48869435500575
C	3.42508984792425	3.72038840895261	0.32413004299914
C	3.04587647421460	4.94723683688360	-0.21068004614148
C	2.66769697363936	5.04948086530596	-1.56236774452034
C	2.87916481985471	6.25264764072273	0.41902293522565
C	2.38532800776822	7.15000700517488	-0.54487216855014
C	2.31848218375207	6.48790437522959	-1.90605813396768
C	3.10476552822089	6.66790236713554	1.72900867494617
C	2.80406123438436	7.98215224528764	2.07459724055980
C	2.29636741778818	8.86884302602450	1.12422011231495
C	2.09513256724296	8.45964784475854	-0.19585868768551
C	1.00735876468334	6.65205193816365	-2.70601285711181
C	1.39035829652376	6.46271173011404	-4.19373452190227
C	2.93001734517284	6.63430191758416	-4.27166095015651
C	3.35737675885231	7.11256238152048	-2.88011899603015
H	2.31205646835999	3.97159964337723	-3.39091862485582
H	3.73595177199577	1.64189330492938	-0.07706932702588
H	3.73088118799042	3.63578639211004	1.36340120053797
H	3.50493381635940	5.97991323180643	2.46799889346872
H	2.96196261374991	8.32054489495408	3.09468608878245
H	1.71608813724413	9.16524019159695	-0.93194813181635
H	0.23770038000127	5.94874254878089	-2.37589898224849
H	0.62466333632423	7.66630570411138	-2.54039593414416
H	0.86834574744841	7.19022290110548	-4.82310796929485
H	1.09115630843987	5.47407562021508	-4.55523718578475
H	3.41140389043299	5.67835402410643	-4.50568272133239
H	3.23346753754688	7.33919556267104	-5.05199772183833
H	3.27010204912705	8.20418973541642	-2.80874459165827
H	4.38350600056174	6.84091457607832	-2.61251734512794

H	2.06121242927249	9.88909661018695	1.41303873715668
C	4.53409728618221	-1.75886600486430	0.98690660919990
C	4.59110166411292	-0.93138347480013	2.12003647146060
C	5.18333504445194	-1.35893362158504	-0.18973797669089
C	5.86237359697564	-0.14998266830581	-0.26490423250645
C	5.90669049319227	0.66667167734619	0.86147970259591
C	5.28267360796902	0.26482314812464	2.05627723703159
C	6.45195909014663	2.00712254094277	1.05217513136002
C	6.18319064371648	2.40706050319102	2.37124155358170
C	5.48673886426272	1.30566621490477	3.14358842084186
C	7.06733967707831	2.87774242739735	0.15622456035345
C	7.40534673395803	4.15580374028639	0.59143129548789
C	7.13622093814116	4.55541805307521	1.90152996419062
C	6.52793655291966	3.67856404677118	2.80136502485610
C	6.37731023344086	0.76118263399531	4.31036017887292
C	5.38990572775280	0.35359563559156	5.40383719908402
C	4.35536570021087	1.48046317359240	5.35475718211072
C	4.16112949384109	1.74556420799287	3.85145104750293
H	4.09961539706551	-1.24179907533173	3.03594323732961
H	5.11669254497864	-1.99466104553990	-1.06678697873284
H	6.32284685591671	0.15819437773987	-1.20041138806440
H	7.26145685674957	2.57707755600339	-0.87000534701625
H	7.87364473708962	4.85366114277740	-0.09649271539378
H	6.31757865835021	3.99979192919781	3.81984241862855
H	7.03627048260520	-0.04503316738365	3.97352298268034
H	7.00994083934852	1.57911490466977	4.67702701255636
H	5.86654568254323	0.24556948856191	6.38430865999746
H	4.92194451741841	-0.60765954309228	5.15872850321754
H	3.41578765683429	1.23449175082149	5.85675295688085
H	4.76763588441239	2.37126960202360	5.84731789198800
H	3.94149850966081	2.79648747567155	3.64679246069807
H	3.31946753112572	1.16412265423812	3.46175050129694
H	7.39831543793111	5.55990987571395	2.22103596074984
C	-2.80323705222528	-3.32937917592341	2.46595851029464
C	-3.62678344549759	-2.72795821242166	3.43342165153126
C	-3.19460336169581	-3.32843039728217	1.12190576771255
C	-4.42249735352583	-2.80710011406771	0.74068116254441
C	-5.24735185823310	-2.24559042884310	1.70787018099111
C	-4.84489286864867	-2.18262241286924	3.05556261033680
C	-6.56452676317393	-1.63757420887631	1.57826545628409
C	-6.96918257325194	-1.18491666127375	2.84510446098982
C	-5.92271434801240	-1.50676503453269	3.89286414309828
C	-7.38700399233116	-1.48446624922140	0.46390474366483
C	-8.62271501676632	-0.86521545770478	0.62962379977074
C	-9.02222444369630	-0.40085102904186	1.88431977869807
C	-8.19715860355274	-0.55983982653116	2.99986545573960

C	-5.42054200444242	-0.27973754632725	4.68850630542539
C	-4.78492707905439	-0.85011434068570	5.96938513034370
C	-5.44648547430313	-2.23864772860392	6.18866883405467
C	-6.44990712301561	-2.41429317777955	5.03372984551322
H	-3.27927144838193	-2.68816199110268	4.46076526445998
H	-2.55119686757060	-3.78188223853747	0.37825363554068
H	-4.74106402038303	-2.87382879531291	-0.29473383346421
H	-7.07991285297316	-1.85876455743620	-0.51022696086250
H	-9.28141069809862	-0.73766501020080	-0.22512713013823
H	-8.52540126979098	-0.20115899361797	3.97281270107895
H	-4.73501198984011	0.34179144713505	4.10806976376442
H	-6.28892682742689	0.34271377896240	4.93741659376636
H	-4.94575865106993	-0.17919195975477	6.81881307978971
H	-3.70150971003571	-0.94308845787029	5.84823148717155
H	-4.69384806415241	-3.03383511883824	6.16910234010647
H	-5.94758702493031	-2.30974436078550	7.15916074637621
H	-7.43830278227494	-2.04357457919038	5.33201161237951
H	-6.57008865742813	-3.45293276143509	4.70955678930495
H	-9.98773199048859	0.08440007095368	1.99465542212323

III'

H	-0.79573052614585	-0.05287901894070	4.92258382940466
O	-1.59307467787915	-0.12056264893489	5.50108418400916
C	-1.25147603161789	-0.29715124541864	6.82793675530083
C	-2.59854102213326	-0.25297425551289	7.61942876635472
F	-2.46731789070665	-0.76584704821187	8.85300851397858
F	-3.03579258326999	1.01555756744506	7.73770440185427
F	-3.55233670071295	-0.94485328245560	6.97864652888461
C	-0.31132192942669	0.85409055048943	7.33614931329486
F	-0.31849614280956	0.95894171318778	8.67670625571773
F	0.95519457490324	0.63145848573302	6.94402510981390
F	-0.69576439914166	2.02867693876901	6.81845775554462
C	-0.55697459796396	-1.68792013365615	7.02597621597448
F	-1.45915653198405	-2.68245059823811	6.89850451893399
F	0.37701689448764	-1.86984076203047	6.08049114934677
F	0.02910932618032	-1.80741852276341	8.22871658194700
H	0.52169547646841	-2.89426203249608	-3.70324719612937
O	1.17823422635384	-3.62825113998941	-3.56697017306092
C	1.48990821530465	-4.29894781577924	-4.74011684619410
C	0.20787445859974	-4.92156051151816	-5.40214416020015
F	0.51181574664326	-5.92567673524504	-6.24179894123551
F	-0.61414895305514	-5.39220688767712	-4.45760015795455
F	-0.45617918680391	-3.98262664973709	-6.09927782867933
C	2.47166367029427	-5.43832112337854	-4.31958180768516
F	3.10531698419983	-5.96649959756001	-5.37754356189714
F	3.39307265540035	-4.97513196525362	-3.46667529314475

F	1.79785018816474	-6.42352902087355	-3.69391060973649
C	2.18916648644843	-3.32850546178330	-5.74967138331306
F	1.50425542003833	-2.17460534894002	-5.81678135036490
F	3.43800062787728	-3.02933800371205	-5.34129519841667
F	2.26851537485031	-3.84732975559237	-6.98365930130276
O	0.09856346760272	-4.83369929512502	-0.99356582891803
H	0.46157983902567	-4.63051601792500	-1.87208226578893
H	-0.75299020214832	-4.36529548537428	-1.00939968600219
C	1.38541237700720	-3.78131053779171	0.63310993019569
H	2.46864588426805	-3.19515931579600	-1.12805825100758
H	1.22983800609129	-2.01338734045523	-0.61156004276652
C	1.94684844999050	-2.76129617443239	-0.27469111124913
C	0.31238599229540	-3.45412002953261	1.53213062329036
C	0.10386334239264	-4.18875768415083	2.72313624746342
C	-0.51169576898705	-2.33807291989964	1.28366297924600
C	-1.47080928248522	-1.94374265567227	2.19536365513156
C	-1.62609208539052	-2.65280036196917	3.38393874107604
C	-0.84555594586245	-3.78130014082504	3.63837730283413
H	0.71939649975855	-5.04849743453109	2.95822735143832
H	-0.41345147901350	-1.77932294435004	0.36488935132167
H	-2.09676742420490	-1.08041932525777	1.98862167794082
H	-2.36055555290708	-2.33210705856753	4.11360948291612
H	-0.98242979443347	-4.33189272970107	4.56358171955235
C	2.20785913252068	-4.99696579231577	0.89651744155685
H	2.83723085778545	-4.71977711388768	1.76277004915102
H	1.55481436477197	-5.80359286101867	1.24178080704157
C	3.09875255863789	-5.52448288340797	-0.22490502714430
H	3.85390106044506	-4.78494136470688	-0.51892388204989
H	2.47785332836564	-5.72124782803626	-1.10551250291038
C	3.80044448483678	-6.81914732583068	0.18840609895926
H	4.52945202621255	-6.60032087769623	0.98183270107037
H	3.06426353376386	-7.50896417501026	0.62433615836302
C	4.49747998017044	-7.49407952635034	-0.98881623188794
H	5.21841586635165	-6.81604042141439	-1.45607618584882
H	3.76839081827753	-7.78809123255128	-1.75242425531865
H	5.03408417070907	-8.39417285008805	-0.67184854748720
O	0.50137482538233	2.55089012059157	1.51145205794512
O	1.90662992281594	1.88376037161088	-0.51777284804581
O	-1.37555160025225	1.34187953939142	-2.52141852485254
O	-2.72980501871991	1.31278497314958	-0.34632695703893
P	-1.52204792080408	0.56614854154610	-1.11425919623918
P	0.90430652184017	1.27448577504819	0.59441497698875
S	-1.81816590824357	-1.87162071360860	-2.67890494589163
S	1.61942756576790	-0.11178826494909	3.02747612137992
O	-0.60692565635164	-1.60308526066392	-3.50886619263976
O	-2.05094322368540	-3.27467422109384	-2.28626981061805

O	0.34539660277476	0.31786721699371	3.65893226912920
O	2.09564082971609	-1.48285901236449	3.25187170611948
N	1.74729276180930	0.25275766505729	1.49434247609621
N	-2.02387256711658	-0.93025868793913	-1.44051707689717
N	-0.27570835099542	0.60084581889467	-0.18132111044385
H	2.69684463041657	-2.21022062767835	0.32246774039013
C	-3.29374938861491	-1.43453796766250	-3.83041048474692
F	-4.42738588107218	-1.77165724675375	-3.17647377886519
F	-3.27582910609778	-0.10409669842400	-4.02667891039812
C	-3.28226493664659	-2.15447112670371	-5.20802138815200
F	-3.04135998176356	-3.46823110375994	-5.01471539025577
F	-2.29523899763735	-1.62329012325785	-5.96593950782031
C	-4.63704624481521	-2.02517104071138	-5.99109213994114
F	-5.52702258317375	-2.89657893946339	-5.46921051395639
F	-5.12695737189367	-0.77072911199657	-5.86568672737521
C	-4.51561505519062	-2.33107633813034	-7.51971413096701
F	-3.86183351367398	-3.48434270522302	-7.71671911156044
F	-5.74713562736459	-2.44060363156263	-8.03880586716033
F	-3.87344946926753	-1.34735981423580	-8.15781397203606
C	2.93752762772667	1.03681685635774	3.88797931747328
F	3.68494652184118	1.61623516021740	2.93002880059470
F	2.28561522247944	1.99877521154497	4.57031358978850
C	3.87933944649274	0.26632186449649	4.85738670069902
F	4.70530012770164	-0.51723949201685	4.11555798892838
F	3.14778712323284	-0.50982299327768	5.68009805002565
C	4.76734223873616	1.19968493923561	5.75279704276434
F	5.24752494852917	2.22943180833721	5.01717381268314
F	4.00857236125721	1.69275992245542	6.75365796526903
C	5.99703031198642	0.48154453104776	6.39963948321294
F	6.93653031567650	0.21646150485956	5.48191194541301
F	6.53069941990200	1.28724637931731	7.32829188511535
F	5.62392449734762	-0.66393456144047	6.98548510981423
C	2.97983276891761	2.61379444666364	0.00796838785619
C	0.29166640668471	3.81636241982598	0.96146353247235
C	4.25112610181301	1.99719464016032	0.05409235585470
C	-0.99450510586701	4.40980482138811	1.10812043788376
C	4.65052163799319	6.48291943404104	2.38467945368913
C	3.59969328173403	5.83902987004905	1.77752879991348
C	3.78924412023965	4.59668500800187	1.12430024455440
C	5.08919359403631	4.00457956393696	1.15954651379906
C	6.15440931361330	4.70392028838742	1.77742030951702
C	5.94389087028083	5.92197600745596	2.37195799090548
H	4.48041303565406	7.43042930890335	2.88727350466389
H	2.60933018252498	6.27906586294486	1.80729528720608
C	2.72793138948784	3.88463993087856	0.48003039874625
C	5.28375095035829	2.72240212175355	0.60862303568689

H	7.14022098643502	4.24597094493785	1.78618175101779
H	6.27907266177808	2.28523483182786	0.63167933975380
C	0.66078775126941	8.24937787844480	-1.39819525350347
C	-0.34301681152786	7.61120349958787	-0.71409190995952
C	-0.13433969604990	6.33274345824357	-0.14189731808554
C	1.15477387917994	5.73043230033998	-0.23904469670753
C	2.16427595956085	6.40618435389561	-0.96603019782995
C	1.92051302148093	7.63080583030804	-1.54008680909334
H	-2.11430595911310	6.17390943174310	0.67047001573627
H	-1.32342318304144	8.06883301088589	-0.61587099799372
C	-1.16299312160874	5.66328002854077	0.54798665204229
C	1.36987777978745	4.46701240230575	0.38936901631559
H	3.14235018494962	5.94786215996821	-1.06137166858620
H	2.70798085084674	8.13090152584550	-2.09684994310515
H	6.76542438693313	6.44630207789512	2.85084624440028
H	0.48704163876036	9.22813254119446	-1.83630283022716
C	-1.61961027217756	2.70211688488723	-2.67720636070740
C	-3.92220453576898	1.35899052216861	-1.08331937566429
C	-4.94570658589187	0.44485504594316	-0.74141053080044
C	-0.55841321294546	3.49426139619911	-3.19602897648170
C	-4.50923553356074	6.56253023615470	-2.30537213730577
C	-4.32614966368755	5.20810854592386	-2.15831351761708
C	-3.10370041771909	4.59646653342734	-2.52445690147898
C	-2.04543433987019	5.42404169998678	-3.00866537979725
C	-2.27671230187560	6.81084063083742	-3.17534744321719
C	-3.48299885626236	7.37080105729660	-2.83587746060678
H	-5.45598800571256	7.01145409849091	-2.01860756344642
H	-5.12750094393314	4.59152380751953	-1.76512520885559
C	-2.88836193350701	3.18819382732299	-2.42225637085645
C	-0.80637853420589	4.84405804099261	-3.34815267114966
H	-1.47085044791669	7.42631303282297	-3.56526307263402
H	-0.03472464290792	5.47883129185567	-3.77640842440375
C	-7.44437103750277	1.98503880582698	-4.61323661757528
C	-7.34976483789840	1.21568445886650	-3.48143901131391
C	-6.20852155770567	1.28889602894952	-2.64658351315622
C	-5.16098824367512	2.20652553351341	-2.96987472303959
C	-5.27748445929139	2.96251810022199	-4.16198422851520
C	-6.38962141603625	2.85265444336193	-4.96127952049539
H	-6.88296404348163	-0.24466977113244	-1.28121419824856
H	-8.14230478711163	0.52079183402759	-3.21605427944058
H	-4.46996601779869	3.62413880599801	-4.45417878593849
H	-6.45141983899078	3.43420319325191	-5.87640189029829
C	-6.07640604162386	0.44310672120221	-1.52423005189376
C	-4.00653236590740	2.26091554097400	-2.12414021250615
H	-3.64773536822618	8.43628413253018	-2.96788216736050
H	-8.31925336513416	1.91272099658724	-5.25238586169940

C	-2.08908573921179	3.81381411316757	1.90273959164052
C	-3.42604881595676	4.05614260396089	1.53000740627215
C	-1.82821414312295	3.11849891668130	3.09524361094551
C	-2.86272455004774	2.68020754330538	3.91190457690389
C	-4.17587835574879	2.94618497870341	3.54270262088512
C	-4.45615932583159	3.63732206239393	2.35047995786789
C	-5.43760308095845	2.57097844703473	4.17053004986722
C	-6.48879250047696	3.03757090944546	3.36461364431208
C	-5.95689657182603	3.81296823786290	2.17460215645586
C	-5.69734792466652	1.84622763891930	5.33161623474276
C	-7.02126095342429	1.58449455271431	5.67283252849992
C	-8.06662783740416	2.04156853368347	4.86855620516495
C	-7.80472358714143	2.77732385842283	3.71142634718971
C	-6.49172754115982	3.32815362329815	0.78537388950627
C	-7.34131315402027	4.47800921983749	0.22456656313499
C	-6.66393970449747	5.72632710338367	0.79499718275952
C	-6.38409936662581	5.32091851286933	2.24251538034476
H	-3.63620872923538	4.55701322116603	0.58686704079838
H	-0.80661899519628	2.92975810917725	3.40185811776753
H	-2.63546806641180	2.13454307762605	4.82263828512756
H	-4.88480419333468	1.48641687075559	5.95672598933477
H	-7.24358255941566	1.01717217867578	6.57208638755027
H	-8.62605150238914	3.13121097284575	3.09165712807326
H	-5.64048943330212	3.13979196737016	0.12250089619825
H	-7.04133653070283	2.38635121388221	0.87217984152642
H	-8.37043870886441	4.41590092163620	0.60263148264849
H	-7.39529930598717	4.46290574691735	-0.87039498502181
H	-5.72135350013742	5.92002945410842	0.26665159392146
H	-7.27855351696673	6.63040867662284	0.72081109303526
H	-7.30790356900592	5.39809664042300	2.82797337956859
H	-5.62395983537047	5.93141541616905	2.73872417055573
H	-9.09343332803615	1.82282342173422	5.14692080167472
C	4.51286399970447	0.63605671467357	-0.44956139196267
C	5.21122163791636	-0.25994902830610	0.37379718063389
C	4.18549402585983	0.26555976352135	-1.76032241840370
C	4.61694897676063	-0.94822927615391	-2.28509113659782
C	5.36476570295139	-1.80538938979352	-1.47986091382643
C	5.62544490177908	-1.47379297014958	-0.13951277147922
C	6.01644168997040	-3.08125261157538	-1.76818816798325
C	6.63169454573361	-3.54220419825792	-0.58995335847647
C	6.41856150702435	-2.56630272235974	0.55105886415311
C	6.14405620057836	-3.79124875172954	-2.95928952205905
C	6.87831274179384	-4.97386975144281	-2.95935412400407
C	7.47409789669313	-5.44093369486797	-1.78719404826718
C	7.35230960312744	-4.72586283407816	-0.59393861999041
C	5.65244847218437	-3.16911417416700	1.78441817415688

C	6.63852504433857	-3.13617051950851	2.96188343206686
C	7.51621173787611	-1.91975590096897	2.65985464966288
C	7.76496927061138	-2.04921833181158	1.15545567822008
H	5.41498086957406	0.01687906766795	1.40554180055745
H	3.62957687057555	0.95609632900357	-2.38676515253002
H	4.39493489990875	-1.19682966035079	-3.31954141442470
H	5.68883538829070	-3.42655329782101	-3.87536965474115
H	6.99025606528582	-5.53858488417881	-3.88057738576820
H	7.83056811567948	-5.09262570642449	0.31195907543978
H	4.78216989985916	-2.54188141349304	2.01267397257119
H	5.28790104706693	-4.17814550032344	1.56538304495894
H	7.25901923787382	-4.04237740411264	2.96628552930572
H	6.12948578367395	-3.07612989558745	3.92934587358056
H	6.96936845740158	-0.99645346684519	2.88574714739407
H	8.44486254697335	-1.90072501178922	3.24036739585568
H	8.54556654794895	-2.79880228995311	0.97893543183770
H	8.08675309618725	-1.12060206550843	0.67438829738680
H	8.04054617655725	-6.36777065381680	-1.80454198608184
C	0.71534273347696	2.90990063089915	-3.66722309163192
C	1.91910214172607	3.61785086635347	-3.50215693655741
C	0.72434310931469	1.70322167882783	-4.38750302799266
C	1.89723919003444	1.20088595832089	-4.93213233129024
C	3.08189085538719	1.90875398638529	-4.76002518523176
C	3.08887726885212	3.11884783625486	-4.04706468735185
C	4.44660908376706	1.60056340068440	-5.16928663403564
C	5.29020970311229	2.63092571564393	-4.71671311221329
C	4.48025141393972	3.72458111003891	-4.03612486631495
C	4.96191496757248	0.48969748508611	-5.83180478351174
C	6.33724619076687	0.40717515000178	-6.02971579497756
C	7.17999604093427	1.41454538190421	-5.56103543284792
C	6.66113019634761	2.53012908049022	-4.89961548672497
C	5.04818125680675	4.12813247078420	-2.64168698185053
C	5.70682357958040	5.52062498756564	-2.80129801376184
C	5.74935355854992	5.79254562929325	-4.31400706622424
C	4.51827267405259	5.05829115970004	-4.84026270362665
H	1.92114887109505	4.54576840915261	-2.93274574202745
H	-0.19874173244062	1.15791166506131	-4.54279021271642
H	1.87630847678943	0.27031564748447	-5.49012285320058
H	4.30653788008421	-0.30615296032380	-6.17775512593238
H	6.75949141265294	-0.45397089368472	-6.53962263085338
H	7.33725934357055	3.28931960180808	-4.51662936084436
H	4.24418465567774	4.15093817153130	-1.90202502857444
H	5.76057419257611	3.37282333576676	-2.29911201132070
H	6.69536370035688	5.56918842611308	-2.33380878116279
H	5.09042601466652	6.28525905363394	-2.31235403954099
H	5.73327339600143	6.86153536269113	-4.55239535641830

H	6.65918156767215	5.37803806056105	-4.76290555450977
H	4.52507845025604	4.88274323509980	-5.92077956436077
H	3.61367467241004	5.63239437134724	-4.59784541350555
H	8.25306530625362	1.32668762421878	-5.70410692478461
C	-4.84050562817250	-0.47849921520311	0.41565797008311
C	-4.75971573176810	-1.86078503306008	0.20542207629376
C	-4.89720257424730	0.02638590837239	1.71914123569305
C	-4.92271539425132	-0.82722843400816	2.81747305146012
C	-4.85930327239554	-2.20086846464164	2.59896675005923
C	-4.74469644943945	-2.71152077396881	1.29607673554281
C	-4.81063605745759	-3.32049482806824	3.53586830750615
C	-4.63139560472300	-4.50483866743073	2.80131846225114
C	-4.58346279240307	-4.21976678124164	1.31346535356085
C	-4.86557713845059	-3.34558950995007	4.92729759546866
C	-4.72091237834057	-4.56679372927260	5.58027795018963
C	-4.53280549783123	-5.74313785701770	4.85227711973533
C	-4.49354445518295	-5.71803644321950	3.45670455407498
C	-3.24476709086753	-4.67935892900591	0.62997509548966
C	-3.62526809568905	-5.79383740609025	-0.35238929339577
C	-5.03005176535483	-5.39318238624627	-0.80566811283113
C	-5.69559681307289	-4.96649344131910	0.50615197423009
H	-4.67075937439348	-2.24428193258116	-0.80598770222878
H	-4.93806764756113	1.09931923393841	1.86883281480638
H	-4.99329736553789	-0.41459523516096	3.82055023802475
H	-5.01354326736919	-2.43091494803085	5.49393311938509
H	-4.75611909848888	-4.60500339589700	6.66534534142843
H	-4.34719280642575	-6.64007774430555	2.89794425584680
H	-2.83047350557177	-3.83065824095960	0.07687675504070
H	-2.50289278400553	-4.98550596411074	1.37392072526606
H	-3.66525078955562	-6.76421789493857	0.16102202324116
H	-2.91318999519843	-5.88291121847818	-1.17961770238287
H	-4.96570534900423	-4.54837397392943	-1.50403642249101
H	-5.57324700940281	-6.19946080321829	-1.31021696434912
H	-6.00034527116101	-5.85909309082202	1.06582705837620
H	-6.58346530907673	-4.34063836267426	0.37401527487348
H	-4.42119459555498	-6.68745987433697	5.37755682503536

TS1

H	-0.19167692634891	2.65679205763607	3.93648265676525
O	-0.68783309451639	3.50726911246445	3.83465197001094
C	-0.23748001856740	4.48690085003210	4.70177666879119
C	-0.82333570298196	4.23944926273678	6.13445815350384
F	-0.20554865550726	4.97547521205809	7.07421314008368
F	-2.13488116290040	4.53682688426577	6.15933784332391
F	-0.69592352061207	2.94462900692028	6.45905636859969
C	-0.77160363630244	5.83873191677998	4.12514093426599

F	-0.74303769876263	6.81503065586206	5.04673904165765
F	-0.03396411018398	6.23204944787616	3.07462936100491
F	-2.03949970773599	5.69876458475480	3.70177697626945
C	1.32702598181656	4.51408848254503	4.75460142969522
F	1.78955481996441	3.54077007839211	5.56035939679830
F	1.82964740627172	4.29679346713183	3.52978419935106
F	1.80124976208929	5.68804979749481	5.20533695842108
H	-1.67885747762906	-0.56021786352163	-5.34337948601780
O	-2.07586897789691	-0.88276434643127	-6.18503912654429
C	-1.10935132835402	-1.08560408349927	-7.15390424334644
C	-0.25967742933002	-2.35899991163208	-6.81288643079723
F	-0.98663946779743	-3.47617388002833	-6.99055256322281
F	0.84612449808286	-2.45910440669546	-7.57063987844968
F	0.11843760869128	-2.32581856482767	-5.52290705853264
C	-0.16678428290295	0.16263809135855	-7.29557370647346
F	0.72135631101017	0.19698905704086	-6.28683213128638
F	0.51679573601580	0.15425014700999	-8.45437587253884
F	-0.88377707536121	1.29417497511390	-7.24159864181233
C	-1.89204255452296	-1.31118157043845	-8.48894672802715
F	-1.10300892944467	-1.84947330323551	-9.43725545500478
F	-2.93170919573167	-2.13335550902142	-8.29701570897616
F	-2.37368947631080	-0.14812075835087	-8.95868896779936
O	-3.42000822801196	2.39717468005664	3.33365168518491
H	-2.84596861347336	1.71905096356212	3.73913656401978
H	-2.86537630528337	3.18920370999519	3.37419860101596
C	-3.36701737033885	1.82587967893937	1.01800046708640
H	-3.45765051788248	-0.08734409052149	1.95270370318998
H	-1.83047825951118	0.31777600649651	1.32718293800804
C	-2.89849573321623	0.43937234596802	1.17379080800524
C	-2.46966182713645	2.89716864276566	0.67268122622664
C	-2.96342756923632	4.16234953562238	0.27422991205296
C	-1.07345771625800	2.73761117188071	0.80216141985326
C	-0.21637965911415	3.80076158587396	0.59017739449091
C	-0.72419959503273	5.04416927145324	0.23064254866833
C	-2.09961460563334	5.21590052941320	0.05023356444091
H	-4.02784751125398	4.31407194956150	0.13587287122985
H	-0.64759450444851	1.78704691187615	1.09660728620875
H	0.85206513486502	3.66119661412600	0.71096082230671
H	-0.04604051307098	5.88288650916322	0.09457293660393
H	-2.49127120179329	6.18180118305290	-0.25360248985214
C	-4.84176053760959	1.98418879313639	0.92367735374331
H	-5.32364513687995	1.30511389639495	1.63470599577202
H	-5.15617937758086	2.99946149291271	1.17445327204966
C	-5.32860028808153	1.62027199125102	-0.50372921851166
H	-5.19657412657179	0.54230149766542	-0.65946219339259
H	-4.71121528928381	2.11916412720017	-1.25835134726280

C	-6.79701633887520	1.99288003525426	-0.69027082871227
H	-7.38931423408740	1.56232286226053	0.12722214720413
H	-6.90825721480002	3.08402854751016	-0.62335118638014
C	-7.33036707120792	1.49630352223521	-2.03123692230163
H	-7.26133303839196	0.40414890897822	-2.09337587451420
H	-6.75518600002986	1.91237039722055	-2.86633451820858
H	-8.37975865173174	1.77591461589968	-2.16988826648032
O	0.54233504437391	-1.63190548006101	-1.98888544209360
O	2.38201457777010	-0.06378832762734	-1.11876307113533
O	1.95009779408252	-2.16257288284945	2.15940747359619
O	-0.23516323234519	-3.01387464334957	1.16687334702766
P	0.48793733626050	-1.65766866413872	1.66752269098772
P	0.80214919572809	-0.36414955555322	-1.02053694763142
S	-0.37672015663767	-0.04928985268617	3.98957412376227
S	-1.22698196539384	0.82449338161419	-2.64364442612875
O	0.56120983606837	1.08311092316174	3.74487113985412
O	-1.77491122219473	0.32941465878551	4.29522008179299
O	-1.02935061954491	-0.08040338355961	-3.80284648380607
O	-2.54950954458296	0.79302023981634	-1.99357859366355
N	-0.00983587805169	0.87146334210710	-1.63511688009116
N	-0.31483042703406	-1.23811749136910	2.98801215875233
N	0.49733212473926	-0.60251918199775	0.51054452983679
H	-3.13749574781775	-0.06438234219501	0.22283876773096
C	0.13318231533199	-0.82873179910627	5.66849163311240
F	0.31826229644673	0.19251201227763	6.53210886800496
F	-0.89972807227874	-1.58356808067899	6.08601015132388
C	1.41844393296120	-1.70123581329005	5.62600918737935
F	2.33956817827227	-1.06783667820539	4.86183282075652
F	1.12169368220554	-2.89500545413307	5.06915605362033
C	2.05619526738015	-1.96198736632992	7.03384826492186
F	2.76086916100337	-0.87297009577423	7.40760366909323
F	1.07829249403720	-2.18652481681830	7.94116658995508
C	3.02249527266580	-3.19162958124478	7.06753819992540
F	3.86780948356252	-3.14980334040656	6.02499526020525
F	3.73773043413061	-3.15427784727704	8.20063369012035
F	2.33935948517417	-4.33783710365380	7.03366618956297
C	-1.08982873977460	2.58992691735643	-3.37027166748419
F	-0.83544866308215	3.45745741850127	-2.37580995451195
F	-0.06589936389784	2.61424862429301	-4.24937482136860
C	-2.40939715929951	3.01550761733861	-4.09184791104491
F	-3.35002031573039	3.28667687248283	-3.15437793540676
F	-2.83619380924920	1.99543515016200	-4.86746187429727
C	-2.26609388852637	4.28536300197467	-4.99508003174610
F	-1.58340795824958	5.24170292391138	-4.31795220528834
F	-1.58367728665575	3.96648067401212	-6.11192057379125
C	-3.62864869450585	4.91938019269750	-5.43205408707231

F	-4.23042714323471	5.52341327517658	-4.39926669737446
F	-3.39244401637829	5.83908311247072	-6.37815753876708
F	-4.44968118631300	3.98753793152760	-5.93117247949196
C	2.86658340376016	0.19116054686723	-2.40931617039005
C	1.46856818544619	-2.61397805109081	-2.31293693135456
C	3.18553073042282	1.53001329660184	-2.75574942547850
C	1.02478083843384	-3.96095621401457	-2.19599808658063
C	3.77200384711561	-1.31933936177448	-6.91738820054516
C	3.39083605658823	-1.61948372806700	-5.63203439806378
C	3.38404063024392	-0.62471804957549	-4.62526234553661
C	3.73395061210944	0.70942367048142	-4.99426747210615
C	4.13992943786308	0.98204658507610	-6.32338224252142
C	4.16561399356473	-0.01254281663574	-7.26707024922910
H	3.75314114095903	-2.09584116554420	-7.67595010654626
H	3.06866723294485	-2.62562832395487	-5.39119204427989
C	2.99861623535954	-0.88011665139032	-3.26781033107957
C	3.64822720208492	1.74061329346383	-4.03981355415696
H	4.41140518251150	2.00165821356939	-6.58487099366527
H	3.95636745303448	2.74086348420506	-4.33192697984790
C	5.49504640351574	-5.40879651152597	-3.45947342376708
C	4.20646434827306	-5.69735231162163	-3.08612320565095
C	3.27152811174646	-4.66340716599933	-2.83994068414943
C	3.66504397575241	-3.30572833532089	-3.03423824456562
C	5.00667489146839	-3.04116025194901	-3.40410837390041
C	5.90107871651810	-4.06608032634428	-3.60018639847619
H	1.63222746758538	-5.99278845631715	-2.40829598153799
H	3.88443580371066	-6.72658894728727	-2.95541234960960
C	1.94602369754028	-4.95317483180920	-2.45803915675219
C	2.70229127233209	-2.26561224814078	-2.83279370650616
H	5.32824098852767	-2.01259134111681	-3.53178751361761
H	6.92679772106991	-3.83840772769388	-3.87509563718929
H	4.46567554064973	0.20745745147897	-8.28719919592585
H	6.20488947899303	-6.21075759437191	-3.64083338782997
C	2.59525849249977	-3.27801728279495	1.62418905578481
C	-0.36097030399963	-3.96818964673904	2.19180996660426
C	-1.59956986732797	-4.05981320990457	2.87518794337165
C	3.87207773261792	-3.07539518041708	1.03353801806757
C	2.71903140589128	-7.99439450706644	0.53156783003811
C	2.05759940699408	-6.93354275616613	1.10247329128612
C	2.65151883555846	-5.64839996239331	1.14842191624803
C	3.93294030475858	-5.46906639720675	0.54645738454484
C	4.60178374448692	-6.58959493619796	-0.00247457987238
C	4.01184184054696	-7.82854474405621	-0.00708840329487
H	2.24426432145694	-8.97080178743126	0.50204209131843
H	1.06873567970216	-7.07631003668856	1.52610040452921
C	2.01023782960322	-4.52498505812736	1.75354980455505

C	4.51119670203328	-4.18478706309098	0.51658037940385
H	5.58490590764932	-6.44146758001548	-0.44036532223511
H	5.50164432534779	-4.06770527528664	0.08380487066299
C	0.51034720197722	-7.16472717677142	5.97287562948228
C	-0.59751459157014	-6.48801800598285	5.52773875336178
C	-0.52951211451604	-5.65634372409365	4.38387099516982
C	0.70432444884461	-5.54972827488172	3.67118655449806
C	1.83518394693183	-6.23539236574784	4.17524388019765
C	1.73813299511475	-7.02492077079679	5.29577918156216
H	-2.59341393463230	-5.03894553546508	4.48393845224809
H	-1.54432001966959	-6.56998945157013	6.05542623623954
H	2.79064942488651	-6.12532344465363	3.67474951561061
H	2.62028649714304	-7.53601660880871	5.67013822305452
C	-1.65313628307464	-4.92009439947627	3.95125464331320
C	0.75853450371925	-4.69621509073355	2.52348970767628
H	4.53202637310658	-8.68051700134393	-0.43540879085202
H	0.44803583919513	-7.79454323225662	6.85534800113698
C	-0.39200030512708	-4.31276229663369	-1.96178574731122
C	-1.41018265376025	-3.63288022707298	-2.65035952187177
C	-0.72384228157528	-5.41553860461054	-1.15881716223104
C	-2.04066135550727	-5.84176178053720	-1.02796453615312
C	-3.04380779006260	-5.15448305461834	-1.71010729745815
C	-2.71917979430566	-4.05837667138124	-2.52529852825774
C	-4.49615316673856	-5.30018156131947	-1.69781380762966
C	-5.05303500262021	-4.30803270587373	-2.53090310973878
C	-3.95454311177626	-3.49790679664549	-3.19693918945169
C	-5.31022305096239	-6.15863305747579	-0.96480597800299
C	-6.69194520429903	-6.01695159975621	-1.05886583915591
C	-7.24781770484821	-5.02827442480373	-1.86952241656470
C	-6.43176132820156	-4.16954851310559	-2.61065469424576
C	-3.92122560129177	-3.70203717378168	-4.73761234959086
C	-4.98361197532066	-2.74391363711360	-5.28786304410522
C	-5.01444586386639	-1.55127239228114	-4.29759742392503
C	-4.13071760398662	-1.96534812669030	-3.10002194072442
H	-1.16470437131672	-2.79207302070909	-3.29148753425077
H	0.06641845354283	-5.92706694866119	-0.61537545691383
H	-2.27824229444643	-6.67946140009789	-0.37733142159012
H	-4.87791146986232	-6.91240354255703	-0.31203940801977
H	-7.34246153643873	-6.67113738374567	-0.48576430770797
H	-6.88378015005128	-3.39788489414299	-3.22674913613735
H	-2.92879256753158	-3.40339647648686	-5.09432682558588
H	-4.08076393521395	-4.74841532016097	-5.01764472075507
H	-5.95883480385459	-3.24179066829382	-5.32664822589025
H	-4.74267214310212	-2.42737861151119	-6.30627575289377
H	-4.62569330497669	-0.63748694875845	-4.75782089086870
H	-6.03969136594306	-1.33013395985331	-3.98037367452723

H	-4.54126807534498	-1.64983097172134	-2.13542647949203
H	-3.14580817892910	-1.50047457782269	-3.18687765188099
H	-8.32766010160432	-4.91955952511368	-1.92034035559806
C	3.06319941496918	2.67913839858214	-1.83577231392686
C	2.65771386410027	3.91779855463333	-2.36754376827491
C	3.43400218422460	2.60410958936676	-0.48557056166002
C	3.43224073762529	3.73703444786041	0.31986484285640
C	3.04550302155343	4.95836031337458	-0.22279395832364
C	2.66490120478640	5.04943314431886	-1.57455142008118
C	2.87044947157393	6.26676243214129	0.39854479910507
C	2.37161057545015	7.15499883408232	-0.57124764042082
C	2.30648452825329	6.48325183596899	-1.92772400592869
C	3.09046332753120	6.69109040761644	1.70653875950183
C	2.78004363708320	8.00514530158300	2.04412884280161
C	2.26872275950318	8.88307086333977	1.08760453191207
C	2.07250569433187	8.46477709482570	-0.23041815734641
C	0.99148668249882	6.63384968760683	-2.72422005104526
C	1.36987217208873	6.43690118080992	-4.21209691163147
C	2.90870333894042	6.61165203366177	-4.29585031966410
C	3.33815818910741	7.10589610570488	-2.91053175707368
H	2.31236079942402	3.95706056158699	-3.39523618427674
H	3.75358258127779	1.65717225325955	-0.06853069750527
H	3.73993513559666	3.66082744344056	1.35938768862201
H	3.49303096754221	6.00999537726642	2.45049956259152
H	2.93345372594577	8.35030270704974	3.06263546361031
H	1.68959579825130	9.16339213810457	-0.97114627542967
H	0.22761283692784	5.92813104803689	-2.38665535706334
H	0.60290087940943	7.64673526354183	-2.56395606498870
H	0.84465197163486	7.16035786810934	-4.84346860628858
H	1.07107854750922	5.44588446607338	-4.56758355129515
H	3.39221494802962	5.65452700288458	-4.52032715990679
H	3.20776814770849	7.30804200645936	-5.08518761616031
H	3.24412387255858	8.19758923068720	-2.84892086924915
H	4.36695289797722	6.84310657480928	-2.64415294562223
H	2.02640903994418	9.90344523037602	1.36998973314815
C	4.54184038753136	-1.75880929458174	0.99485919616507
C	4.59793221529645	-0.93138851293754	2.12801192138323
C	5.19321403404548	-1.35970333201633	-0.18074502176430
C	5.87289757974540	-0.15109590971413	-0.25541019399846
C	5.91623268722427	0.66562511614023	0.87088142105291
C	5.29045371908854	0.26423581870212	2.06494460192105
C	6.46242842625100	2.00560420036093	1.06234905403238
C	6.19186212938108	2.40586266416672	2.38094438182053
C	5.49333728910875	1.30512111059458	3.15224508004920
C	7.08179859429206	2.87495876969063	0.16789450890897
C	7.42244398809706	4.15191856908123	0.60428652380329

C	7.15144588314924	4.55187583222293	1.91390891928135
C	6.53877290109562	3.67639864446827	2.81213624375106
C	6.38173979352683	0.76062706947250	4.32075940024266
C	5.39229668171740	0.35448031020019	5.41294317236713
C	4.35845313087908	1.48188936051301	5.36123096835807
C	4.16652762532330	1.74529461939561	3.85730410413771
H	4.10472878145605	-1.24118404818902	3.04326383325732
H	5.12711478134359	-1.99562478067744	-1.05759435035299
H	6.33471220361673	0.15665631438934	-1.19038783176661
H	7.27766810859792	2.57392694375183	-0.85789497597964
H	7.89461801310738	4.84855095304574	-0.08224327695179
H	6.32720757522404	3.99775708016860	3.83032022588886
H	7.04064983208197	-0.04622687570363	3.98537884028046
H	7.01432963596392	1.57832863753577	4.68797046254664
H	5.86726455612005	0.24703251863775	6.39429593639772
H	4.92422312655554	-0.60674518985862	5.16791026366831
H	3.41801480315214	1.23688194896979	5.86218620770365
H	4.77059140612888	2.37303335456908	5.85328971633744
H	3.94663762428510	2.79573047523611	3.65049329394589
H	3.32597836105398	1.16250473989298	3.46730423394013
H	7.41601667045335	5.55538625833965	2.23446587876691
C	-2.80328989312585	-3.31340399782059	2.46057489942921
C	-3.62864387799063	-2.72149582635177	3.43178581344083
C	-3.19176026800551	-3.29847413579737	1.11552674055240
C	-4.42037113606929	-2.77651933990310	0.73735331342119
C	-5.24832369595831	-2.22731809644178	1.70896840460369
C	-4.84626876889821	-2.17402960659606	3.05726052465834
C	-6.56960711249617	-1.62654072668872	1.58527046948903
C	-6.97495191055514	-1.18436042783306	2.85559043736186
C	-5.92334817307411	-1.50320119900413	3.89893712115286
C	-7.39630861997287	-1.47444067561265	0.47401020588547
C	-8.63579680581700	-0.86449889845309	0.64579009972224
C	-9.03540252280174	-0.40917510710899	1.90372913262087
C	-8.20677954895054	-0.56854470427773	3.01655583138340
C	-5.42069110821029	-0.27180309851766	4.68794068525162
C	-4.77132430582174	-0.83676855546022	5.96363247589615
C	-5.43388981573637	-2.22259160764701	6.19686414532087
C	-6.44029114203560	-2.40885712662501	5.04541212973271
H	-3.28315390235729	-2.69166761712636	4.46013736067816
H	-2.54628677905434	-3.74300359263162	0.36850952396720
H	-4.73676242002329	-2.83280505684179	-0.29937715976782
H	-7.08878742024335	-1.84175800167443	-0.50264423219677
H	-9.29735784490622	-0.73720442363624	-0.20676176965906
H	-8.53534907579185	-0.21750892224671	3.99218750477250
H	-4.74411978463378	0.35271156097168	4.09961569448131
H	-6.29074613239798	0.34578849287598	4.94343646169833

H	-4.91978958219329	-0.16139212974191	6.81179173717683
H	-3.68984020725307	-0.93343802083332	5.82924095249912
H	-4.68190698975985	-3.01844468755876	6.18433617325833
H	-5.93357054726427	-2.28358037178713	7.16876906169139
H	-7.43009102923119	-2.04371977047899	5.34573372094748
H	-6.55480237470389	-3.44957300817670	4.72594556260478
H	-10.00406692369147	0.06858790094079	2.01891875283810

TS1'

H	-0.80062373707089	-0.06147563978272	4.90414932039144
O	-1.59606285934776	-0.11127555080945	5.48638064389271
C	-1.25150272972993	-0.29414864548680	6.81141895121567
C	-2.59449526247946	-0.23229283246073	7.60857923411313
F	-2.46545251632906	-0.74742998212968	8.84142308448073
F	-3.01381458030106	1.04233858471666	7.72910457849181
F	-3.56064967162096	-0.91033461205058	6.97176980864547
C	-0.29472364111058	0.84430011594668	7.31809657392422
F	-0.29897655369628	0.95006516078766	8.65881763358156
F	0.96832914906470	0.60443945182930	6.92551619498537
F	-0.66367180785880	2.02387343971319	6.80030300825726
C	-0.57472313098010	-1.69423478565019	7.00470329917121
F	-1.48972849150664	-2.67648760412038	6.87818645225076
F	0.35434097337086	-1.88600506271926	6.05643655013104
F	0.01335412806299	-1.82284847713514	8.20589550484400
H	0.54495737544590	-2.87544693177903	-3.69131739602988
O	1.20228248962624	-3.61080168821668	-3.55510256758023
C	1.51132283629526	-4.28421357475649	-4.72944345379006
C	0.22855804224064	-4.90973366732865	-5.38703577833882
F	0.53291695830382	-5.91862223048652	-6.22031891807485
F	-0.59239644445608	-5.37466001436962	-4.43841362246352
F	-0.43556846531698	-3.97574733342248	-6.08978576640360
C	2.49647583391160	-5.42254394487097	-4.31453811043650
F	3.13079800768460	-5.94353440608897	-5.37485315844371
F	3.41570379499128	-4.96078425405613	-3.45933716184821
F	1.82445958961953	-6.41279793583994	-3.69380408687059
C	2.20427086815331	-3.31076261928307	-5.74025735659135
F	1.51520708673304	-2.15926481419060	-5.80336716531976
F	3.45312242999973	-3.00906064075080	-5.33584276327686
F	2.27966402231432	-3.82911545010812	-6.97439800208772
O	0.21174649835167	-4.71652521066806	-0.98048405317032
H	0.59957463240491	-4.50475763767319	-1.85143677923099
H	-0.64271499298371	-4.24371885656849	-1.00795287742011
C	1.35118978764819	-3.84506532897996	0.52552296762017
H	2.57795694725868	-3.17563966524965	-1.11433187750974
H	1.31178515248169	-2.01030571667024	-0.63729309352052
C	1.99790035962898	-2.77859778608166	-0.28042192430339

C	0.28887957029841	-3.48962617260722	1.45420770602119
C	0.08032443868488	-4.21520944923904	2.64455375384673
C	-0.51005685916999	-2.35974365722525	1.21153516684897
C	-1.45783795417583	-1.94588225731800	2.12878513954989
C	-1.61756838239410	-2.64753843086581	3.32038162037296
C	-0.85448121336377	-3.78630141561541	3.56981405192980
H	0.67941218481786	-5.08726296340176	2.87714268580777
H	-0.40127299621172	-1.79890729647042	0.29463343960283
H	-2.06813081373573	-1.07105403276997	1.92272955134219
H	-2.34051302701121	-2.31227986840001	4.05513827496181
H	-0.99097834614935	-4.33339085669549	4.49739278064300
C	2.17315251849010	-5.05978499124992	0.82404914496115
H	2.77944763088766	-4.76776896332793	1.70003567096389
H	1.51333444849923	-5.86461421557476	1.16097549943364
C	3.09200935486048	-5.59558429104005	-0.27024693664373
H	3.84248254446413	-4.85157381195592	-0.56490002680483
H	2.49439693554003	-5.82244760937868	-1.15992201813525
C	3.80833243658541	-6.86982862026120	0.17989671123429
H	4.51672314278346	-6.62239485121652	0.98336633509609
H	3.07739252274569	-7.56618392433148	0.61413344404346
C	4.54267993580007	-7.55036663768264	-0.97121039187834
H	5.25672492002108	-6.86443869068294	-1.43796393907393
H	3.83598022666055	-7.87567215107846	-1.74337221588624
H	5.09400409325197	-8.43174878800076	-0.62788154897758
O	0.51002464872340	2.56600541692446	1.51204132927992
O	1.91549670279934	1.90687221977925	-0.52040926425871
O	-1.38363365105723	1.35586274864581	-2.52671120759357
O	-2.73207312286110	1.33280214944963	-0.34703172929365
P	-1.52144226247678	0.58959694345180	-1.11289121632096
P	0.91329875668033	1.29303518506678	0.58974630159567
S	-1.78912942979519	-1.87002956039205	-2.64471511854696
S	1.62413954501510	-0.12556516135523	3.00534231957784
O	-0.58375954454506	-1.59477561443786	-3.48220412931385
O	-1.97986076323092	-3.27328758108206	-2.22104088123705
O	0.35225299053000	0.30109699097280	3.64203351651029
O	2.09554272626063	-1.50150522059978	3.20718766795150
N	1.75729110513269	0.26632964394821	1.47939708987880
N	-2.02415718281106	-0.90898061442228	-1.43155448933695
N	-0.27106665190714	0.63095917048781	-0.18943634191469
H	2.69532068081968	-2.26689502427395	0.40405521236929
C	-3.27892481845730	-1.48717632378500	-3.79893719080207
F	-4.40302535951269	-1.83602648161392	-3.13586359764142
F	-3.28470114741978	-0.15970862533143	-4.01237010036248
C	-3.25950172262030	-2.22719190411443	-5.16464512151346
F	-2.99859029119527	-3.53394162350249	-4.94810670268890
F	-2.28177274962032	-1.69354396180011	-5.93233533113121

C	-4.61742215432444	-2.13149731035209	-5.94728591609023
F	-5.49315308627129	-3.00795907194902	-5.41019955600437
F	-5.12541401851342	-0.88263154776979	-5.83974001325675
C	-4.49387478565150	-2.45940370104883	-7.47116874623352
F	-3.82575702937275	-3.60759049415926	-7.65026069888666
F	-5.72430650378398	-2.59224650038441	-7.98714284023311
F	-3.86436775918553	-1.47804797003939	-8.12517357283674
C	2.94718443047811	1.00486063363683	3.88309614869917
F	3.70726543322129	1.58551458998221	2.93539793936680
F	2.29919808671015	1.96755076417045	4.56822332966898
C	3.87483621709347	0.21768899986508	4.85276968922056
F	4.70197941176682	-0.56356568225374	4.10979189664688
F	3.13072057234046	-0.56251259460964	5.66007000872093
C	4.76218131992598	1.13393049861767	5.76597803397558
F	5.25977163768763	2.16563451620032	5.04458412723863
F	3.99835530032354	1.62506817405337	6.76387662989398
C	5.97804547503485	0.39740750430312	6.41845724476394
F	6.92433422931213	0.13146815583940	5.50786466423641
F	6.51066120769348	1.18844480762115	7.36037090082785
F	5.58703675985955	-0.74971923585490	6.98906282861188
C	2.98766728640020	2.63480993689480	0.01017895780185
C	0.29774436894263	3.83158917804993	0.96358456395482
C	4.25925378274654	2.01884312997672	0.05794129739197
C	-0.98977114505679	4.42268511794459	1.10906175379422
C	4.65094236251644	6.50193829329325	2.39559570999460
C	3.60183024896870	5.85820121716795	1.78533627459515
C	3.79328207931054	4.61605326130696	1.13228248914095
C	5.09315324267943	4.02397602522428	1.17067488680984
C	6.15642872792638	4.72283398048342	1.79237311254747
C	5.94422195853620	5.94065290695386	2.38681669949350
H	4.47944901476010	7.44929010066520	2.89802116905469
H	2.61143171011510	6.29840036150638	1.81201102286552
C	2.73392089668324	3.90437256555936	0.48460328057271
C	5.28974819778078	2.74285309365260	0.61779983440478
H	7.14220859289717	4.26486871850965	1.80382329286954
H	6.28509149300747	2.30582118684787	0.64294418758965
C	0.65948441538138	8.26418305526490	-1.39852399346937
C	-0.34367435069145	7.62411233864755	-0.71521623653435
C	-0.13260249636816	6.34669337082678	-0.14155721211249
C	1.15818839947189	5.74761262416973	-0.23620344075710
C	2.16714948912726	6.42538784399148	-0.96201749463184
C	1.92112121792692	7.64881290599512	-1.53769735121117
H	-2.11279946149286	6.18419042808280	0.66920310042142
H	-1.32533437415787	8.07940631267236	-0.61855222120322
C	-1.16038877573480	5.67535612494003	0.54774781405744
C	1.37510920335215	4.48486952737668	0.39272866046511

H	3.14658692011175	5.96951234536053	-1.05514840954508
H	2.70823661077389	8.15055183725475	-2.09348277366992
H	6.76437249544735	6.46473376017369	2.86834137588812
H	0.48390223154772	9.24207581647070	-1.83783364883723
C	-1.62614311198441	2.71692043046966	-2.68159266512295
C	-3.92415526582534	1.37126897754475	-1.08565792929240
C	-4.94320683119451	0.45155390605640	-0.74449379295740
C	-0.56499936677056	3.51114525641469	-3.19690267321781
C	-4.52237439444096	6.57184690623278	-2.30753670929862
C	-4.33748795338027	5.21745978875857	-2.16246881510595
C	-3.11312861770734	4.60874358076183	-2.52693864105853
C	-2.05486525799722	5.43871683599388	-3.00724115734336
C	-2.28784960076189	6.82556267104182	-3.17128054958075
C	-3.49586832126506	7.38282278919539	-2.83346089419513
H	-5.47030370119176	7.01892464359721	-2.02181840608740
H	-5.13869444707970	4.59851921931315	-1.77253631003015
C	-2.89535227882863	3.20101620708847	-2.42657597772405
C	-0.81436060152510	4.86113319061675	-3.34571956379057
H	-1.48207589494879	7.44316519982564	-3.55798811127754
H	-0.04258455094231	5.49810713533967	-3.77050723772827
C	-7.43662963448317	1.96729735267531	-4.63039772691036
C	-7.34044803256942	1.19913857638971	-3.49788086793789
C	-6.20321245044538	1.28172253746189	-2.65842647821660
C	-5.16146871891947	2.20710172209923	-2.97856960807353
C	-5.27958816463296	2.96253820559773	-4.17085566527060
C	-6.38781445878998	2.84376451332267	-4.97434890719762
H	-6.87305248235495	-0.25263964697068	-1.29179703105368
H	-8.12881952810784	0.49853986339369	-3.23507082758247
H	-4.47662597120724	3.63135808848468	-4.45931310082147
H	-6.45121974066768	3.42532473513763	-5.88934934045842
C	-6.06991125873680	0.43961566980421	-1.53346125548393
C	-4.01050483012039	2.26988097239596	-2.12904738625509
H	-3.66196414295596	8.44832592169182	-2.96356181345924
H	-8.30855573865188	1.88799802629088	-5.27275543866406
C	-2.08332031278592	3.82616426468959	1.90456620588220
C	-3.42080120255520	4.06754863237969	1.53303165553029
C	-1.82096359536380	3.13216815321029	3.09753545578651
C	-2.85436238258440	2.69515645663202	3.91642043953657
C	-4.16808288840034	2.96044305341496	3.54844807003555
C	-4.44973197982194	3.64944158191003	2.35521774327325
C	-5.42931266391304	2.58499125386197	4.17744004367010
C	-6.48123295597568	3.04902679005938	3.37105011928585
C	-5.95067389717787	3.82244200601796	2.17926368668613
C	-5.68795819531891	1.86174709492680	5.33970674222887
C	-7.01148983578378	1.59895926378178	5.68155525706943
C	-8.05759503890742	2.05382110042258	4.87694910809411

C	-7.79675430874941	2.78801966842593	3.71869035786868
C	-6.48435805340075	3.33187390114457	0.79179259776407
C	-7.34777696850911	4.47256617894088	0.23440301431221
C	-6.67425266987498	5.72729360735379	0.79506475762150
C	-6.38150285375022	5.32981388983604	2.24236928105436
H	-3.63257084212018	4.56702513238189	0.58941856427576
H	-0.79888007301578	2.94382348288440	3.40292822974594
H	-2.62548800431160	2.15029224589276	4.82725152229875
H	-4.87493770906903	1.50349402035793	5.96502483227248
H	-7.23288302440057	1.03272625198489	6.58170161468736
H	-8.61864351647866	3.14018516797929	3.09868504652142
H	-5.63259103862250	3.15518127409912	0.12631427880288
H	-7.02211196022362	2.38330587694223	0.87914482660108
H	-8.37314960853032	4.40396805506407	0.62148793082322
H	-7.41075391451233	4.45353904003288	-0.86002692572982
H	-5.73647247313172	5.92360990983001	0.25921609764303
H	-7.29444745322729	6.62757799777454	0.72130701916547
H	-7.30039134326591	5.40795340388040	2.83533251233871
H	-5.61883415469097	5.94500793552126	2.72877256290811
H	-9.08411217940353	1.83427892982244	5.15576021234554
C	4.52337268564673	0.66016114755721	-0.45133402320663
C	5.21555436204485	-0.24172907748535	0.37061847212105
C	4.20233566576588	0.29783140026904	-1.76590498143755
C	4.63382635125496	-0.91391187917150	-2.29530376215112
C	5.37334853674650	-1.77827033832100	-1.49010704551414
C	5.62764941078351	-1.45439172110771	-0.14680596795299
C	6.01869711293416	-3.05720211806314	-1.78004145871814
C	6.62220592713622	-3.52786934993358	-0.59958493377325
C	6.40842941706066	-2.55534674068269	0.54404470841605
C	6.14992213195189	-3.76314488955562	-2.97314090457019
C	6.87511770957465	-4.95148427142176	-2.97312900848636
C	7.45917291735688	-5.42779559945276	-1.79888518268801
C	7.33404371951658	-4.71676047356467	-0.60348752824031
C	5.62918702717600	-3.15925153098288	1.76854484385618
C	6.61267108387205	-3.15282589896581	2.94833069013997
C	7.50166278433224	-1.94017572115446	2.66510033435626
C	7.75500865839609	-2.05199815334748	1.16004490793481
H	5.41465448548129	0.02844505076704	1.40503936593535
H	3.65077338206283	0.99301866608179	-2.39113362646583
H	4.41751467675882	-1.15561846637458	-3.33261665724310
H	5.70513891794615	-3.39064804170309	-3.89118347624424
H	6.99010779146553	-5.51270067753420	-3.89613354065465
H	7.80339786816541	-5.09049456744077	0.30421042039637
H	4.76758957588363	-2.52191697704046	2.00113056191481
H	5.24911065055342	-4.16006048743485	1.53752631640315
H	7.22503324740939	-4.06458636076397	2.94081325321152

H	6.10216397350998	-3.10213866026647	3.91553540328831
H	6.96203753233782	-1.01520066772692	2.90101908544016
H	8.42843509328293	-1.93663994717416	3.24895703762276
H	8.53305476499484	-2.80249217295069	0.97654289615098
H	8.08256544232796	-1.11858316268090	0.69234181335125
H	8.01975020505219	-6.35822479825926	-1.81631042889138
C	0.70919450503929	2.92884828113720	-3.66918387731110
C	1.91282955293928	3.63654807167773	-3.50232581817004
C	0.71832732619684	1.72423276578857	-4.39289620243061
C	1.89114717817292	1.22391170606097	-4.93952277186622
C	3.07568807719657	1.93148832688161	-4.76575777150624
C	3.08252094678031	3.13935068314312	-4.04894792662124
C	4.44037039792160	1.62488913872387	-5.17628064550618
C	5.28387062616028	2.65371888663438	-4.71999474886478
C	4.47368303178319	3.74549291186813	-4.03684435421257
C	4.95610450812335	0.51625923918915	-5.84220654749200
C	6.33164576223848	0.43387273455311	-6.03882253964588
C	7.17424019153058	1.43922925521167	-5.56556191844314
C	6.65491932427852	2.55296148233417	-4.90148431891890
C	5.04255194145091	4.14708636967418	-2.64241437978134
C	5.70523767278927	5.53777678874878	-2.80175557219276
C	5.74349425800745	5.81289607670511	-4.31413170917183
C	4.51055925372051	5.08060397029653	-4.83872482074001
H	1.91519823312279	4.56266623157377	-2.92999176733042
H	-0.20486527313218	1.17930716298059	-4.54933037790287
H	1.87032565714232	0.29538549661992	-5.50092780749593
H	4.30086056563558	-0.27822578107682	-6.19159691389541
H	6.75418779878204	-0.42573867241939	-6.55108523313634
H	7.33081644085607	3.31058204520009	-4.51499392680974
H	4.23841336688864	4.17217526903349	-1.90296584913557
H	5.75244931310515	3.38955719595522	-2.29961282666860
H	6.69577887925800	5.58202337325567	-2.33809935459357
H	5.09335621547788	6.30338917207977	-2.30879112196796
H	5.72735235399858	6.88242208219202	-4.55008651371236
H	6.65176935311965	5.39892869350706	-4.76664759994049
H	4.51457540992402	4.90707248202374	-5.91959435005605
H	3.60694583870706	5.65483033369673	-4.59297185175720
H	8.24748928810671	1.35113020321316	-5.70682721181952
C	-4.83790122460026	-0.46648712867867	0.41609356057188
C	-4.78446851536912	-1.85110090542197	0.21198350751170
C	-4.86721599192862	0.04499819506890	1.71799226046910
C	-4.89569881924880	-0.80338642454683	2.82027010585541
C	-4.86028335936865	-2.17900247805593	2.60761416641130
C	-4.76974494521592	-2.69706054766654	1.30596509103133
C	-4.82243330995608	-3.29560543729619	3.54862690807925
C	-4.67143463821128	-4.48579864818060	2.81754701692350

C	-4.62905133619269	-4.20723399850189	1.32828840520035
C	-4.86553667190294	-3.31420258345616	4.94030863196571
C	-4.73814653591864	-4.53528299240838	5.59709663675571
C	-4.57832890417273	-5.71774538637582	4.87248416607382
C	-4.55023183606515	-5.69872440990437	3.47651610303143
C	-3.29819037780167	-4.68369747680250	0.64072681339941
C	-3.69432258550995	-5.80099088114629	-0.33187339873541
C	-5.09718072253653	-5.38945900836030	-0.78224818212403
C	-5.75252008137347	-4.94485159036077	0.52901522798401
H	-4.71427878996800	-2.24070012166009	-0.79829862242122
H	-4.88560596707952	1.11912328999323	1.86362794006675
H	-4.94629344196789	-0.38540171037807	3.82228159043066
H	-4.99146950588403	-2.39477225428354	5.50408694217534
H	-4.76442907211709	-4.56835564476311	6.68258640536746
H	-4.42543957190292	-6.62546540357180	2.92024684313323
H	-2.87899089508822	-3.84314891094989	0.07955816836874
H	-2.55474151867151	-4.99042359687521	1.38262453304678
H	-3.74239895014191	-6.76787749134102	0.18749667612278
H	-2.98657716788391	-5.90176689020948	-1.16187819642364
H	-5.02779810821892	-4.55084565349398	-1.48768863259425
H	-5.65116545731975	-6.19388439136873	-1.27801247220823
H	-6.06727235250824	-5.82951387844249	1.09567403997361
H	-6.63289738221113	-4.30890082757465	0.39483879112140
H	-4.47940035494926	-6.66186955959129	5.40066396386935

IV

H	-0.09789717870410	2.59535106647114	3.96457261273612
O	-0.63471116414527	3.41656134120082	3.80926925718282
C	-0.45929215063505	4.39262080101145	4.78129243481822
C	-1.31330066589133	4.03255591239874	6.04538025989965
F	-0.98483897012453	4.77150288300472	7.11483299679514
F	-2.62172773371297	4.21484561562065	5.78637946793395
F	-1.14220269620727	2.73775985619412	6.35376488411191
C	-0.97153009570462	5.72356133416248	4.13582870702547
F	-1.22429018347145	6.65195455453565	5.07286173061056
F	-0.06658655260648	6.21944083039162	3.28303486988568
F	-2.10331807086329	5.49456115468495	3.44987778427555
C	1.05078756330006	4.52410782014647	5.16543808406146
F	1.41742041955068	3.50422425559797	5.96510637247768
F	1.81539085845791	4.46691682287086	4.06569979543972
F	1.30725147493945	5.67479013461656	5.80382830841857
H	-1.58338860603849	-0.63866627732171	-5.32351892733694
O	-2.02926396198566	-1.02199780619184	-6.11680494594666
C	-1.12137119999150	-1.27593576420857	-7.12813067144721
C	-0.22944603748902	-2.51087706345328	-6.75504671043278
F	-0.94809128974867	-3.64658635718994	-6.80732275594339

F	0.82933194958146	-2.65154536054518	-7.57261011849710
F	0.22734869417552	-2.38436725944758	-5.49672247829244
C	-0.21368217186066	-0.02635008074752	-7.41775896737094
F	0.74377350627995	0.09030611217553	-6.48304887619596
F	0.38624698627570	-0.10787168889234	-8.62072255245274
F	-0.94564195548933	1.09590052195984	-7.38972750277656
C	-1.98039122023586	-1.60671693244310	-8.39279098677152
F	-1.24612033335369	-2.20861272747297	-9.34644109666531
F	-2.99596176622455	-2.42097744690528	-8.07653115960732
F	-2.50506566195279	-0.48589973254716	-8.91595003713263
O	-2.97684294645994	2.18238452780229	2.94435030018357
H	-2.54466948246188	1.35777942035022	3.37387344467593
H	-2.30731364947211	2.89369070144517	3.08792148117702
C	-3.20418555743868	1.98618435623234	1.35832733962632
H	-3.04347083290675	-0.15449038026760	1.59685381064783
H	-1.50111693782046	0.64219761308391	1.18759330703989
C	-2.57813549699220	0.65591402892321	1.02453318735334
C	-2.50882964537445	3.19924008876794	0.78032069628179
C	-3.22454708807803	4.31913506875523	0.34291286976708
C	-1.11042198986220	3.26361762550667	0.79012208563557
C	-0.44986166592985	4.42900041569548	0.42891431155936
C	-1.17199527314301	5.54295995966328	0.01726876321454
C	-2.55976030503333	5.47637836887926	-0.04729480841717
H	-4.30779773623038	4.29918881559024	0.30045740167333
H	-0.51718792298598	2.40999680878157	1.10252888127271
H	0.63375592012555	4.45862099140997	0.46272676604917
H	-0.64966925440109	6.45764063695652	-0.25056622698412
H	-3.13338520711290	6.33421050860566	-0.38559122920788
C	-4.71555417627452	1.95477388882352	1.23604796762868
H	-5.10269519742693	1.18384090027658	1.91389287771949
H	-5.13694556553746	2.90669473524894	1.57843575987309
C	-5.17515144213592	1.63696440290062	-0.19388072665563
H	-5.05346030648413	0.56182212540298	-0.37893038064385
H	-4.53588229975307	2.14151324898452	-0.92997449407316
C	-6.63370063877946	2.02586331543706	-0.42265237223954
H	-7.25633540032782	1.58228359554930	0.36585424404808
H	-6.73787173842527	3.11654130564866	-0.32829278692334
C	-7.13680354260283	1.57555755911372	-1.79096982348508
H	-7.07690514380712	0.48502401304644	-1.88482321899239
H	-6.53243554888543	2.00800873596979	-2.59668603243250
H	-8.17874912268137	1.87039944091889	-1.95264187450130
O	0.50787433377801	-1.65669946577074	-2.04253796436509
O	2.35054539727154	-0.09244855859699	-1.18998485405860
O	1.94750671776943	-2.24533387221760	2.16824239539493
O	-0.22259166907362	-3.08758824833383	1.14346773456960
P	0.50747650485533	-1.73264518159247	1.62815993381909

P	0.77557774503424	-0.40493930905892	-1.05852396147848
S	-0.37741715179808	-0.11180482768279	3.93050281100509
S	-1.15771054853475	0.83448761337407	-2.77370947024977
O	0.56000660413421	1.02759614244118	3.71993003049492
O	-1.79305996975699	0.29682707366048	4.20572964746637
O	-0.78699874334485	-0.01679360526565	-3.93259863594126
O	-2.55788241568189	0.78256073376643	-2.32136769681684
N	-0.11214217552451	0.81649535440336	-1.58140969885516
N	-0.30778636788635	-1.29652089217099	2.94740578262638
N	0.55711846752116	-0.68434313466560	0.48092145769349
H	-2.73571142139729	0.46798443596547	-0.04103155946467
C	0.06101700485576	-0.86916985956650	5.63893972784913
F	0.23892174799113	0.16107231125140	6.49078730485111
F	-0.98953974556788	-1.60483911022971	6.03942946703800
C	1.33791961256151	-1.75879942159904	5.62449334439632
F	2.27594918418725	-1.14426373475600	4.86628686323916
F	1.03138324508284	-2.95338049162376	5.07751822073613
C	1.95094553269601	-2.00851336601470	7.04450836064780
F	2.65373170403264	-0.91824093747854	7.41763773229349
F	0.95549645107641	-2.21843824121806	7.93631393830577
C	2.91067717834065	-3.24247509060655	7.10498474881674
F	3.77892908533910	-3.20591604974645	6.08194365974397
F	3.59876700428469	-3.20250027160054	8.25417796506598
F	2.22342088404813	-4.38560365963884	7.05909149937795
C	-0.93341261963037	2.62570661729625	-3.41283319604061
F	-0.73337425696968	3.45334832717590	-2.37635461911628
F	0.14994747281434	2.65708985358011	-4.21956858031675
C	-2.19473968285660	3.09309232457328	-4.21098003172119
F	-3.18195243236178	3.38768755525663	-3.33360293664291
F	-2.60087656614739	2.09150421684834	-5.02229332590548
C	-1.95093467470459	4.35839952300095	-5.09816389656301
F	-1.26394326997524	5.28282413608022	-4.38252347752626
F	-1.22471240969605	4.01277450556234	-6.18047743166432
C	-3.25948326409176	5.05048105276015	-5.60443262917066
F	-3.88764693500569	5.68080733012753	-4.60446185823180
F	-2.93394396217369	5.95856105424414	-6.53647073240252
F	-4.09168587341256	4.15421535741841	-6.14811875007640
C	2.82486479026325	0.17774115921338	-2.47838413840335
C	1.43910506811680	-2.63226183119512	-2.37538884822276
C	3.15781363973221	1.51835360122400	-2.81086289526153
C	1.00438076428916	-3.98165159584855	-2.25343155624016
C	3.76366677879619	-1.32108952047874	-6.98369109049752
C	3.37175822693448	-1.62520375866688	-5.70274106555817
C	3.35423870028588	-0.63335269808249	-4.69354363040604
C	3.71312204768144	0.70095556616465	-5.05238662832898
C	4.13069719635557	0.97774562065323	-6.37745990492185

C	4.16075552574506	-0.01290791192456	-7.32517063559858
H	3.75043585324671	-2.09521229032559	-7.74488463461441
H	3.04897434261945	-2.63234377853946	-5.46783656569637
C	2.95884116928854	-0.89060438297044	-3.34038381189038
C	3.63283412668793	1.72697722050298	-4.09224965130063
H	4.40734725041278	1.99761559260619	-6.63255135036291
H	3.96029230078571	2.72188854211538	-4.38008084588428
C	5.47932575143942	-5.40452942600105	-3.52519226440000
C	4.19220983414440	-5.70053760731236	-3.15255147589192
C	3.25120244870549	-4.67216168602621	-2.90620865814094
C	3.63602735854623	-3.31224087361580	-3.10283041184387
C	4.97718959328342	-3.03987937876187	-3.46887118533314
C	5.87807626157896	-4.05940604535755	-3.66348647652757
H	1.62076145817674	-6.01079498162317	-2.46559400526606
H	3.87614213902037	-6.73165334505730	-3.02209285768535
C	1.92887089441263	-4.96961256487899	-2.51900259076886
C	2.66704514139355	-2.27678235882298	-2.90399366256808
H	5.29319363666988	-2.00941319352096	-3.59449905761527
H	6.90310067088079	-3.82550624002138	-3.93577496931813
H	4.46874794852693	0.21089826065047	-8.34211378565563
H	6.19374053794888	-6.20237120734328	-3.70693169038598
C	2.60465656244020	-3.34229016045706	1.60562443778796
C	-0.34230050065994	-4.04161745080191	2.17111410740827
C	-1.57442343496257	-4.12605904356425	2.86845383872745
C	3.87121962407845	-3.11663029032401	1.00267318378972
C	2.76024151666100	-8.04237645154113	0.45587978871512
C	2.09239371414000	-6.99433934613853	1.04259422270642
C	2.67427994321728	-5.70425139213380	1.09763336473354
C	3.94941065716153	-5.50450072534972	0.48767904109248
C	4.62452701463808	-6.61263268265676	-0.07857400601085
C	4.04695755727576	-7.85733819764271	-0.09107168603819
H	2.29538574242915	-9.02328296142104	0.41945367659399
H	1.10796324046568	-7.15108205253745	1.47179052293944
C	2.02792186157145	-4.59391405447110	1.71951936043421
C	4.51486364240098	-4.21401998250990	0.46532195739975
H	5.60235728970205	-6.45039555556099	-0.52318186975055
H	5.49793421254647	-4.08224503689216	0.02011875555301
C	0.54930865291705	-7.25457104852399	5.93331197058383
C	-0.56089235863229	-6.57574471706253	5.49754565208747
C	-0.49937060500697	-5.73902957829061	4.35682758067135
C	0.72996186161023	-5.63168342425192	3.63619187970546
C	1.86364427838506	-6.32006230588173	4.13055806040223
C	1.77332861553637	-7.11267396381132	5.24950342290887
H	-2.55806492808753	-5.10967153873379	4.48085383239688
H	-1.50461791637469	-6.65947487422233	6.03050702974319
H	2.81546808172773	-6.21015031134519	3.62310573519011

H	2.65717733290542	-7.62646600403533	5.61605157721420
C	-1.62263268315145	-4.99406314835592	3.93903276067102
C	0.77824711501246	-4.77421877612284	2.49148261937746
H	4.57221118919345	-8.69933231436992	-0.53269315984659
H	0.49199834753572	-7.88792737492575	6.81359671850506
C	-0.40824483523320	-4.33746641494265	-2.00204835851629
C	-1.43629180247307	-3.66722787954700	-2.68535478858891
C	-0.72776322973361	-5.42762182024016	-1.17748663042324
C	-2.04329897278379	-5.84661120249348	-1.01508685201322
C	-3.05697693267165	-5.16369163691933	-1.68587945432295
C	-2.74459743388626	-4.08346928522110	-2.52707202493205
C	-4.50959028087806	-5.29539493138655	-1.63243915834768
C	-5.07920201216632	-4.31019860348317	-2.46478461433021
C	-3.99241099356492	-3.52180911595142	-3.17402331902329
C	-5.31100399981797	-6.13288186090808	-0.86222282055505
C	-6.69313253127400	-5.97608750174163	-0.91758757666947
C	-7.26100973349911	-4.99261628271627	-1.72635738670425
C	-6.45782072166873	-4.15532985409529	-2.50473282868845
C	-4.00580976772393	-3.74624860058538	-4.71229615858507
C	-5.07993890591323	-2.78898893424435	-5.24004471241625
C	-5.05999983779064	-1.57747769147809	-4.27328574897639
C	-4.15209746365271	-1.98663103186417	-3.09201893569419
H	-1.19800254564328	-2.83886151676307	-3.34569573388217
H	0.07077230110176	-5.93234448375879	-0.63955714317203
H	-2.27188629759924	-6.67320331361278	-0.34716042852142
H	-4.86781015819147	-6.88098748435645	-0.21018651633123
H	-7.33401428772814	-6.61337024395401	-0.31532040963814
H	-6.91838239245989	-3.38598617570801	-3.11733148269739
H	-3.02420894105031	-3.45382147430728	-5.10204325834034
H	-4.17806476507181	-4.79515794912179	-4.97501521572282
H	-6.06048769214038	-3.27809831248741	-5.23118570957766
H	-4.87742856499687	-2.49492506143363	-6.27313392652516
H	-4.66387810912615	-0.68111462103641	-4.76021793029846
H	-6.07123912762541	-1.32865318943343	-3.93267954866810
H	-4.53814719162045	-1.65487562372872	-2.12321162491539
H	-3.16624879500874	-1.52959796084132	-3.20482169669132
H	-8.34023351840597	-4.87037634825536	-1.74604351779798
C	3.04531873093255	2.66229025095730	-1.88207012212382
C	2.76026717130801	3.93324083061735	-2.42002896667346
C	3.29222441347181	2.55038331467462	-0.50519682387741
C	3.28472546909584	3.67036180048094	0.31669545619874
C	3.03288133663263	4.92070013323348	-0.23420961428517
C	2.77342510734061	5.05220476447518	-1.61070155435095
C	2.87777033136685	6.22604722990504	0.39645413952140
C	2.50508564891051	7.15387982430791	-0.59159449801361
C	2.49917499921628	6.50534309161433	-1.96051249508335

C	3.01224850277804	6.61581593718782	1.72658485867335
C	2.74988297879238	7.94033712570227	2.06424497969757
C	2.36712910998226	8.86035858240291	1.08699775631817
C	2.25209537724592	8.47315665962669	-0.24981381258823
C	1.22808523220661	6.70856530371164	-2.81549380024472
C	1.67192763965939	6.52691530936170	-4.28707580892221
C	3.21822423395411	6.65093809322867	-4.29408958381158
C	3.59748289787761	7.10519505217607	-2.88028798768052
H	2.49513855580842	4.01154969473168	-3.46855025915647
H	3.51597979240139	1.58417423558835	-0.07298351679328
H	3.48686027591237	3.56086139145690	1.37828135505863
H	3.31645679353644	5.90179249433951	2.48647102376916
H	2.84126730350531	8.26188491799704	3.09793424250408
H	1.96521676780305	9.20276841577351	-1.00402651643121
H	0.43296507655206	6.01817579048875	-2.52149363535113
H	0.85963663125152	7.72885118045615	-2.65463481539725
H	1.20254246825035	7.28021862360460	-4.92750595860922
H	1.35793524624125	5.55411448930205	-4.67752964469532
H	3.68058243660460	5.68232507984291	-4.51443900620486
H	3.57819531624311	7.35118093670324	-5.05434684901664
H	3.53996362361934	8.19850788846583	-2.80385287547773
H	4.60246762069597	6.80067060100778	-2.57071899870387
H	2.16099833970844	9.88886773810905	1.36883416926007
C	4.51512460003429	-1.78747716886138	0.96404940907848
C	4.55257672582160	-0.95795541955619	2.09659258555589
C	5.14543175635120	-1.36724176381450	-0.21598045770871
C	5.78234027640237	-0.13561410102216	-0.29552108261262
C	5.80056030090590	0.68567905389089	0.82834719445332
C	5.19772128841309	0.26294966847309	2.02636141628736
C	6.28495426287381	2.05044832485741	1.01163182211065
C	5.99708070209811	2.44641216521675	2.32794840167941
C	5.34943717967004	1.31958070542230	3.10676197094390
C	6.85190891977937	2.94494443947569	0.10757306682415
C	7.11830458155207	4.24368831293712	0.53036841047743
C	6.82912361982671	4.63975422017200	1.83721252210590
C	6.27293620008324	3.73845831810848	2.74639475674673
C	6.25038977609680	0.82836739727704	4.28935478901534
C	5.27243326952991	0.41521486723913	5.39038775423978
C	4.19876499150938	1.50254617647323	5.30511826056748
C	3.99404686597100	1.69861129629604	3.79421479899270
H	4.07763758403054	-1.28315743117688	3.01634552144024
H	5.09176507497174	-2.00310807817220	-1.09407473951600
H	6.22597309086896	0.18735654834994	-1.23409852304581
H	7.05740793384527	2.64725947163572	-0.91729501437797
H	7.54422868525749	4.96086128179005	-0.16514435328102
H	6.04524853874274	4.05798760192674	3.76173946500506

H	6.93398901184768	0.03465567047395	3.97301950997096
H	6.85728407361283	1.67234252446428	4.63976416498376
H	5.74777876134820	0.35004605723676	6.37519029965301
H	4.83765871524858	-0.56740669062559	5.16868375193853
H	3.26901714181459	1.24307998081996	5.82000277049097
H	4.58102307449486	2.42560681298875	5.76167219569140
H	3.69689417383747	2.72115174533416	3.54703274456102
H	3.20089737043310	1.04077826723508	3.42672095131685
H	7.03238846253768	5.66103692492515	2.14613468506824
C	-2.77146713912663	-3.35482050313255	2.48034870987084
C	-3.57697488170969	-2.76733126587868	3.47187494152907
C	-3.16899676497410	-3.29899863719414	1.13930007477905
C	-4.38808418327686	-2.74172074906204	0.78325445879570
C	-5.19703748510102	-2.19821940814964	1.77391129419855
C	-4.78341742323803	-2.18216865502248	3.11983361790385
C	-6.51627604270613	-1.58862786620233	1.67697677719024
C	-6.90466186305683	-1.16653337523546	2.95883167067331
C	-5.84390104197959	-1.51280352246191	3.98483620013268
C	-7.35832614771300	-1.42189664557290	0.58021913681928
C	-8.59352256285815	-0.81129512938802	0.77671771418447
C	-8.97477295060254	-0.37172249475171	2.04579643633996
C	-8.13218538344791	-0.54960549367697	3.14538442668680
C	-5.31701410561426	-0.29917556179762	4.78672670798020
C	-4.66429702683296	-0.89039875654989	6.04814076911200
C	-5.34650479419894	-2.26792930780371	6.27195442607711
C	-6.36023947994279	-2.42869057739690	5.12313954540787
H	-3.22669101641397	-2.77527353404632	4.49918889708604
H	-2.54030535136225	-3.74044845442825	0.37651411491948
H	-4.71211353278188	-2.76847832630159	-0.25203294908346
H	-7.06360591696193	-1.77625023382382	-0.40479695779715
H	-9.26522873325271	-0.66999099593251	-0.06549870831700
H	-8.44826153998419	-0.21364474830122	4.13051513833170
H	-4.63002161025152	0.31659423189785	4.19960466712179
H	-6.17437621699761	0.33018293993010	5.05607557734949
H	-4.78924605629121	-0.22380159701075	6.90710306667515
H	-3.58765008267539	-1.00568807193526	5.89612420450165
H	-4.60591074084135	-3.07421154639223	6.25060186957022
H	-5.84324674551168	-2.32896114393166	7.24530127311743
H	-7.34521987531706	-2.06066040165585	5.43520321218538
H	-6.48529207108039	-3.46359613052653	4.78951124159741
H	-9.94007935515284	0.10771970703737	2.18028229780664

IV'

H	-0.83324326533029	-0.03309752187361	4.91106810735198
O	-1.62771070726084	-0.04751459359726	5.49563002937839
C	-1.27868130362086	-0.18529663893157	6.82564977848549

C	-2.60415231199879	-0.00022231746109	7.63175343479114
F	-2.49355020932384	-0.45995319712837	8.88758449489138
F	-2.94174118828704	1.30325763215414	7.68924891203842
F	-3.61962795808878	-0.64659573730827	7.04019313899681
C	-0.24959670547677	0.91518403857554	7.27247160671063
F	-0.25550059741695	1.10200851953521	8.60462899650232
F	0.99712286201346	0.56890049288844	6.91102634591786
F	-0.53488147425421	2.08353242905227	6.68092095664849
C	-0.68491970931413	-1.61316494849043	7.07770867233002
F	-1.65656146827781	-2.54318202863138	6.99985464517004
F	0.22388084719258	-1.89928646215733	6.13389534916750
F	-0.09602423934547	-1.72257198927930	8.28126847866952
H	0.53900359780889	-2.92892785519456	-3.73308509682985
O	1.16605141642550	-3.69627990483214	-3.62656163971419
C	1.55803989147171	-4.26112260580863	-4.83663264313436
C	0.35208979758791	-4.97239990280374	-5.55066415498375
F	0.76962687160599	-5.88883491174114	-6.43904106412066
F	-0.42203507030396	-5.58074155362043	-4.64411460254496
F	-0.40015901984982	-4.07201908975120	-6.20519605837596
C	2.65156021083765	-5.31981119719889	-4.48530945555665
F	3.35065969355125	-5.68574399445836	-5.56892146843807
F	3.50290846785080	-4.83087463113722	-3.57919721803802
F	2.07917489970969	-6.42055799201348	-3.95811466038817
C	2.15716146165603	-3.15825702517614	-5.77108194130867
F	1.35526616940946	-2.08007245545813	-5.76767090060233
F	3.36561155128762	-2.76220422573238	-5.33083831871821
F	2.28897001918780	-3.58634687821211	-7.03358250552892
O	0.36218760615750	-4.50349996106251	-1.03814158599309
H	0.83769237081092	-4.39657512593977	-1.89687961812445
H	-0.48360074117958	-3.97088069366929	-1.16120043709634
C	1.27999600502019	-3.98172088888041	0.20990612948285
H	2.76472433823368	-3.12114796308837	-1.12739534158915
H	1.43970331498724	-2.01188258314367	-0.71464220363369
C	2.07519757709481	-2.81588090761938	-0.33600458291944
C	0.27949554250122	-3.60322751254507	1.27015400966027
C	0.13102000204030	-4.33388635040884	2.45398544247589
C	-0.49083867710597	-2.45156341595926	1.09551421731898
C	-1.38887918487658	-2.03456323603361	2.06499618672960
C	-1.50915489962959	-2.75299793829111	3.24935244005635
C	-0.75013250862891	-3.90178715631253	3.43829585075296
H	0.71880726290883	-5.22427317538970	2.64221142221106
H	-0.38648546321890	-1.85333569348529	0.20095558411187
H	-1.98156589981203	-1.13851788593995	1.90360126377319
H	-2.19203580911387	-2.42009676910119	4.02347246291668
H	-0.84208840123553	-4.46371764218682	4.36293916152867
C	2.12000670640662	-5.20074450891982	0.54543080961564

H	2.71473627142075	-4.91156306962199	1.42348176770205
H	1.45128228274675	-6.00763650385124	0.86437531086831
C	3.04416012962575	-5.73964970860001	-0.54353650604754
H	3.77782785140774	-4.98658164455238	-0.85633260361051
H	2.45787030911785	-6.00820177767692	-1.43172970934391
C	3.79540211205669	-6.98700486855078	-0.07336620825573
H	4.49583467605310	-6.70363157305931	0.72476769786119
H	3.08475521427784	-7.69569888321699	0.37419363863359
C	4.55048813328021	-7.66666991064781	-1.21181268766514
H	5.24728427578484	-6.96910606585430	-1.68808143448516
H	3.85597081204057	-8.02543412838373	-1.98022017404987
H	5.12513674950787	-8.52629584615634	-0.85221652707396
O	0.49825182121426	2.58226945048217	1.52434336100152
O	1.93625787267679	1.94051972168779	-0.49274039349468
O	-1.37716184230643	1.34524631138160	-2.54125821950289
O	-2.71286887518527	1.36972935396571	-0.35602924963053
P	-1.50234171310213	0.61476263616975	-1.10510808575977
P	0.92770383976181	1.31442595597747	0.60513079884956
S	-1.71695866998804	-1.90326052955110	-2.53272980000835
S	1.61124865800472	-0.14397022624701	3.00727039065676
O	-0.53664984448353	-1.62547729974078	-3.40446996733173
O	-1.79699788363007	-3.29954444365703	-2.01653223402030
O	0.34140800367290	0.29123809238060	3.64125810291240
O	2.06322342828024	-1.52843824934288	3.18163855989520
N	1.76601731758515	0.28717737464642	1.49205490513695
N	-2.02126041041809	-0.88847670210966	-1.39618545622658
N	-0.24432801531973	0.65745359833268	-0.20089651207580
H	2.65714039839244	-2.39012868681279	0.48908429901852
C	-3.24949547401399	-1.70474221059189	-3.67676916201348
F	-4.33152010909565	-2.11753256530068	-2.98572995468061
F	-3.35860632911062	-0.39069961248343	-3.93267462861817
C	-3.17566151243733	-2.49318823174665	-5.01183788152345
F	-2.79135637431037	-3.75960858010259	-4.73944433449402
F	-2.25774502862740	-1.90477084723940	-5.81133952412796
C	-4.54216691223399	-2.55601595055801	-5.78022037810014
F	-5.33447010789776	-3.47423963175252	-5.18692374186397
F	-5.15068551364556	-1.34952271705720	-5.72692783228838
C	-4.40493468090070	-2.95091112914304	-7.28676759494514
F	-3.65235604645645	-4.05340836911388	-7.41209531466453
F	-5.62484847418412	-3.20164135710293	-7.78127851675378
F	-3.85477234126786	-1.96089585369790	-7.99626936044129
C	2.95052077850245	0.94680847384708	3.91119861329131
F	3.72919568621426	1.52904787819904	2.97857797790201
F	2.31881082237559	1.91200386043369	4.60812973240398
C	3.85929398541546	0.12521871012291	4.87102047104139
F	4.67998488347043	-0.65257078485346	4.11708965954057

F	3.09972579049788	-0.66079458460006	5.65751377738960
C	4.75529013318051	1.00800899245892	5.80833820031494
F	5.27629128300627	2.04527419617469	5.11149515090021
F	3.99352838595943	1.49256693107244	6.81088230913706
C	5.95422885101356	0.23790576514576	6.45371470156942
F	6.90339358340795	-0.02293233938404	5.54442382657052
F	6.49248155333000	0.99935357353764	7.41678460963938
F	5.54131475244985	-0.91508968416140	6.99597301532433
C	3.00043876720569	2.66568127010826	0.05605814148304
C	0.29373058201021	3.84980023708485	0.97802953786975
C	4.27233647378458	2.05177612342854	0.11420219719610
C	-0.99648406558678	4.43971125512988	1.10724513319580
C	4.62511199495038	6.52172887673488	2.48401542900642
C	3.58546572463435	5.88033085288085	1.85537281259171
C	3.78670352304759	4.64007209109438	1.20181814713688
C	5.08592175042883	4.04712899603422	1.25782851139454
C	6.13904591783701	4.74337352972570	1.89931073099256
C	5.91787686793382	5.95939987772064	2.49436333832901
H	4.44630113137748	7.46781124447851	2.98630240406105
H	2.59477336903332	6.32069341563485	1.86753995141675
C	2.73741877913101	3.93090188271008	0.53576869166767
C	5.29293756275818	2.77040946474108	0.69768884068443
H	7.12440486532262	4.28511359161955	1.92497440581148
H	6.28846573817604	2.33441036806514	0.73365928315560
C	0.67539263053480	8.28005231981311	-1.38765862930197
C	-0.33586738271309	7.63708902353314	-0.71908447725234
C	-0.12793238173387	6.36299619508279	-0.13687534847524
C	1.16713683884078	5.76988100991172	-0.20715399465657
C	2.18540445992838	6.45167597044352	-0.91653304554629
C	1.94305959571271	7.67179477254813	-1.50082798410432
H	-2.11809433045267	6.19702756151466	0.64837231387835
H	-1.32140883588070	8.08750667398865	-0.64048542255791
C	-1.16307528215497	5.69021186672224	0.53981897744991
C	1.37820286659298	4.50785387332899	0.42537422720157
H	3.16932889264683	6.00199495246019	-0.99002651522996
H	2.73792311831106	8.17604448254754	-2.04317886252525
H	6.73053684092532	6.48135066943620	2.99068530875433
H	0.50230460098582	9.25544200193001	-1.83347923761513
C	-1.61146370846398	2.71038656091878	-2.69873992677205
C	-3.90581041766157	1.38270536786620	-1.09654828307140
C	-4.91356213957761	0.45349636384204	-0.74420590280358
C	-0.54202345793038	3.50871361536475	-3.18815268060830
C	-4.52376213335571	6.55756004066076	-2.36176069044066
C	-4.33842811105471	5.20331550242569	-2.21624233098149
C	-3.10584482117949	4.59920273771916	-2.56060543921512
C	-2.04116489798453	5.43275739290832	-3.01966819713355

C	-2.27411159710970	6.81967632750529	-3.18352403517052
C	-3.48957682273469	7.37258332194946	-2.86630187817559
H	-5.47781977338096	7.00189399489603	-2.09284589099484
H	-5.14436553035012	4.58142879958923	-1.84054413769578
C	-2.88398915808256	3.19298799120556	-2.45886096701798
C	-0.79317177399847	4.85920580923363	-3.33538096704246
H	-1.46281989569551	7.44043502835593	-3.55321058972193
H	-0.01520404297406	5.50064423634420	-3.74159062030489
C	-7.40018948197884	1.87615993088544	-4.67316756639220
C	-7.29717729707422	1.11925514974010	-3.53349395349546
C	-6.16658218792335	1.22872803909458	-2.68793069188091
C	-5.13856149983716	2.16824204987731	-3.01319118866789
C	-5.26318745988071	2.91237204971631	-4.21162523739051
C	-6.36562993285386	2.76873051204509	-5.01923507843646
H	-6.82764167685961	-0.28904280058575	-1.29955643940382
H	-8.07624254421924	0.40932014775788	-3.26797348845498
H	-4.47072594084370	3.59408303283486	-4.49945813508725
H	-6.43605488833937	3.34354784027266	-5.93795474609132
C	-6.03015842485369	0.40724064104291	-1.54839670444754
C	-3.99467117082425	2.25799747843423	-2.15793982893804
H	-3.65607349000192	8.43806881083608	-2.99598017533994
H	-8.26797561164071	1.77728890484570	-5.31844707932780
C	-2.09675167401060	3.85047489653269	1.89942422174790
C	-3.43176885955089	4.09133143091769	1.51796345442894
C	-1.84327978320230	3.17076591249492	3.10240495413835
C	-2.88255696703063	2.75318724172013	3.92454317456856
C	-4.19385367429823	3.01881834187271	3.54736845198948
C	-4.46637766597611	3.68831388238645	2.34064547322673
C	-5.46019391338508	2.65104867629319	4.17095871060509
C	-6.50522814754610	3.09941043041642	3.34749985950288
C	-5.96570065977485	3.84920715928109	2.14485851302694
C	-5.72838975833262	1.94686926372673	5.34250283639674
C	-7.05415236383051	1.68708375105291	5.67672907121608
C	-8.09366192549320	2.12726491994334	4.85577993655260
C	-7.82351318308886	2.84266568365734	3.68799664415567
C	-6.47767357739375	3.31442142025962	0.76604381206612
C	-7.38082682824358	4.41245438833121	0.18963012581408
C	-6.73190390748538	5.69897181165515	0.70529222811196
C	-6.41212265109621	5.35383302656906	2.16082832997105
H	-3.63788756282430	4.57830589351575	0.56639629793210
H	-0.82327184632742	2.97975052662035	3.41376222134863
H	-2.65927868264911	2.22059634648660	4.84399134827320
H	-4.92125523336644	1.60120485672298	5.98204587739543
H	-7.28224941904240	1.13403705803007	6.58331108628230
H	-8.64043637454964	3.18336875343181	3.05519760619323
H	-5.61834038405850	3.16446709377698	0.10359857202506

H	-6.97541344981053	2.34573143255147	0.86783825870115
H	-8.39860081912124	4.32715649071331	0.59322179281127
H	-7.45725367203926	4.36091230562803	-0.90294548533267
H	-5.80462728547573	5.89892564191375	0.15294996209788
H	-7.37308035327253	6.58249522751493	0.61135456333852
H	-7.32089184352355	5.44444026012371	2.76718231923522
H	-5.64820525301208	5.99414271477899	2.61154664991668
H	-9.12233362291759	1.91053264798891	5.12893337317443
C	4.54490923134354	0.70568663011086	-0.42381890106324
C	5.21617763345465	-0.22544996839138	0.38188506752743
C	4.24982169302406	0.38905652823935	-1.75604010960525
C	4.68805732063584	-0.80478989917568	-2.31776761340439
C	5.40256756762003	-1.70085939066955	-1.52534605492895
C	5.62849567869906	-1.42471391949407	-0.16645255194723
C	6.03287489009265	-2.98031906264020	-1.84193222055343
C	6.59461639920908	-3.50214828609189	-0.66255535438157
C	6.38054830252686	-2.55807870699188	0.50423680051030
C	6.18027424914293	-3.65016058216094	-3.05371620471220
C	6.87397265536619	-4.85703544772208	-3.07302973657737
C	7.41162887278358	-5.38660406536317	-1.89937322674101
C	7.27455786583983	-4.70930904253907	-0.68576366204773
C	5.58040383348445	-3.18250300364908	1.70384519939994
C	6.55371516207719	-3.22721324000390	2.89130894235103
C	7.46365625097375	-2.02091206353539	2.65140674582071
C	7.73008807609060	-2.09442541755386	1.14646551122621
H	5.39446013940119	0.00784420180594	1.42887804815353
H	3.71019576573259	1.10568720645615	-2.36688226844748
H	4.49431307219388	-1.00889900767271	-3.36756284047364
H	5.77019828442888	-3.23657916060914	-3.97086874789132
H	7.00124515153064	-5.39098144505182	-4.01059071193948
H	7.71045457201167	-5.12278210307043	0.22139234176076
H	4.72825039790948	-2.53764449796273	1.94833853190450
H	5.18298344227234	-4.16887249249837	1.44167130719910
H	7.15247876150032	-4.14758493685721	2.86109181495014
H	6.03536465524663	-3.19779840327242	3.85514499723321
H	6.93608955569090	-1.09455916820585	2.90806050541413
H	8.38462182465193	-2.04818997814075	3.24381843345477
H	8.49709801706714	-2.85285827929098	0.94939539778081
H	8.07832517524219	-1.15385677611328	0.70882598406809
H	7.94856652547178	-6.33048126572115	-1.93205047809277
C	0.74549509690855	2.94016772758426	-3.64092548127445
C	1.93612917522239	3.66444165348379	-3.45352343895607
C	0.78153937938019	1.74341148032553	-4.37665770247556
C	1.96571572114792	1.27709099006168	-4.93027806575372
C	3.13432544199116	2.00899504786585	-4.74859194098221
C	3.11678689775774	3.19974224741708	-4.00426588525740

C	4.50227919275039	1.74971918973873	-5.18051251896762
C	5.32351966342020	2.79005974477314	-4.70869620261613
C	4.49449258975949	3.83411615461487	-3.97530561561517
C	5.03907249486972	0.67386432061979	-5.88274351424735
C	6.41317959640977	0.63482834190351	-6.10040677066228
C	7.23459778769441	1.64968296406133	-5.61061985693827
C	6.69473730494557	2.73025726142949	-4.90950683454788
C	5.06535309052819	4.17664433883989	-2.56824488190080
C	5.80029927103429	5.53633877423737	-2.69117041744071
C	5.74760501266152	5.91838192094801	-4.18310483528763
C	4.49656810186406	5.21079144206393	-4.69931034849747
H	1.91858926622827	4.58078188352821	-2.86634307313012
H	-0.13087251837928	1.18290302115990	-4.54207539460728
H	1.96582180911620	0.35801933244627	-5.50791648050302
H	4.40117252725563	-0.12820297954390	-6.24655415569004
H	6.85175602896822	-0.19818074447285	-6.64224849395807
H	7.35619190320450	3.49409661574382	-4.51082806747301
H	4.24801018680691	4.23518055539543	-1.84516551408131
H	5.72521607878261	3.37570348353641	-2.22467517567604
H	6.82601132823778	5.48704606425326	-2.31224126739898
H	5.28429854364656	6.29678484710712	-2.09288004707166
H	5.70911077402766	7.00166256750334	-4.33970834267423
H	6.63336239915818	5.55085098501761	-4.71259876212531
H	4.45851733568939	5.10104004256146	-5.78790320649339
H	3.60096556534697	5.76297733975533	-4.38307703758639
H	8.30778949621274	1.59495536710890	-5.76822672010807
C	-4.80123282311177	-0.43126196471082	0.43605519603440
C	-4.90347207561046	-1.81960729535697	0.28046500834520
C	-4.65247727554544	0.11345874075991	1.71708824630779
C	-4.67180668008833	-0.69660056239336	2.84740310800105
C	-4.79614502105416	-2.07412687822206	2.68386353004721
C	-4.86328009834168	-2.63185264138307	1.39737351904460
C	-4.80513781627086	-3.16556130815133	3.65454795152432
C	-4.82369251039736	-4.38273356472731	2.95313184967908
C	-4.83036135516823	-4.14539708280143	1.45641321853650
C	-4.77127145928905	-3.14614535545505	5.04591377387460
C	-4.73849600915850	-4.35738213255580	5.73178161931888
C	-4.74506285291442	-5.56737645852912	5.03611741381324
C	-4.79197021913888	-5.58514198334529	3.64052191038493
C	-3.54528063232506	-4.71380043891732	0.74731658915158
C	-4.02983793617593	-5.86611992888834	-0.13869222981859
C	-5.41840300273885	-5.40205031711412	-0.58325418656504
C	-6.01553520715596	-4.83814990007949	0.71125050581392
H	-4.96927989266800	-2.24534863967970	-0.71537583317415
H	-4.53938355637943	1.18621387772841	1.82676228992218
H	-4.58110348055886	-0.24901470767941	3.83372010816831

H	-4.76825114658993	-2.20541601820093	5.58666871621636
H	-4.70797945531924	-4.36037072706393	6.81766533949641
H	-4.79668771572345	-6.53304485184857	3.10640384248690
H	-3.11012438897912	-3.92718628085533	0.12295370266988
H	-2.78599448402741	-5.00672116814846	1.47846520429316
H	-4.12103482796868	-6.79263882686880	0.44472735202076
H	-3.34916907200494	-6.06177749147572	-0.97433068970449
H	-5.31958230791769	-4.61338217939252	-1.34153443922177
H	-6.03026869841026	-6.20098671251003	-1.01609348246674
H	-6.38877104168431	-5.66395102484061	1.32865136708292
H	-6.84871384745251	-4.14691651615846	0.55117476930152
H	-4.71811472738923	-6.50363967300175	5.58643437967993

2a

C	-1.02195104411970	1.82103669213149	0.25534432500717
C	0.21871391979571	1.81898280584442	-0.37127084989667
C	1.10302530127133	2.89242226635681	-0.21934082006285
C	0.71444729592101	3.96088454618388	0.58915837452414
C	-0.53006392379207	3.96602189419125	1.21519361356939
C	-1.40479404656638	2.89893396717602	1.04915598899399
H	-1.69291570958523	0.97686484872766	0.12256371956734
H	0.49915040328504	0.96405519152690	-0.98043145536479
H	1.40333593261209	4.78424704476465	0.74722216606798
H	-0.81186867324562	4.80809054895427	1.84138668698841
H	-2.37507267051230	2.90238744402455	1.53725280414321
C	2.47480953050135	2.86813143713185	-0.89376050988454
C	2.34602640307574	2.56095077404670	-2.39017475781137
H	3.33499567117234	2.61339909854165	-2.85616133488494
H	1.93174332403081	1.56500110534794	-2.57012682786424
H	1.68499882067341	3.28845477081909	-2.87615898496516
C	3.38808835249494	1.83467406221599	-0.21491820734651
H	2.95465210708071	0.83845639329889	-0.37539865997751
H	4.35277186370202	1.85474652221387	-0.74055772378950
C	3.60741452531012	2.06500619382607	1.27796667558417
H	3.99899410514373	3.07779055292536	1.43472683874648
H	2.64294330158578	2.01851196191428	1.80186601000398
C	4.56525975061348	1.04268916589782	1.88878725054077
H	5.53206968371418	1.09303042264519	1.36875919901896
H	4.17595678221908	0.02956200692199	1.71457509385179
C	4.77609309467621	1.26020620098862	3.38563998177769
H	5.46376520963707	0.52016503419194	3.80774956702416
H	5.19198566574315	2.25494353947321	3.58161338093715
H	3.82751421409550	1.18588905326332	3.92929134620285
O	3.14672534294995	4.12847467511245	-0.74027564575065
H	2.66072968654049	4.78453148931581	-1.25470525496093

References

(1) Akiyama, T.; Itoh, J.; Yokota, K.; Fuchibe, K. Enantioselective Mannich-type reaction catalyzed by a chiral Bronsted acid. *Angew. Chem., Int. Ed. Engl.* **2004**, *43*, 1566-1568.
(2) Uraguchi, D.; Terada, M. Chiral Bronsted acid-catalyzed direct Mannich reactions via electrophilic activation. *J. Am. Chem. Soc.* **2004**, *126*, 5356-5357.
(3) Coric, I.; List, B. Asymmetric spiroacetalization catalysed by confined Bronsted acids. *Nature* **2012**, *483*, 315-319.
(4) Garcia-Garcia, P.; Lay, F.; Garcia-Garcia, P.; Rabalakos, C.; List, B. A powerful chiral counteranion motif for asymmetric catalysis. *Angew. Chem., Int. Ed. Engl.* **2009**, *48*, 4363-4366.
(5) Liu, L.; Kaib, P. S.; Tap, A.; List, B. A General Catalytic Asymmetric Prins Cyclization. *J. Am. Chem. Soc.* **2016**, *138*, 10822-10825.
(6) Wakchaure, V. N.; DeSnoo, W.; Laconsay, C. J.; Leutzsch, M.; Tsuji, N.; Tantillo, D. J.; List, B. Catalytic asymmetric cationic shifts of aliphatic hydrocarbons. *Nature* **2024**, *625*, 287-292.
(7) Leinung, W.; Tsuji, N.; Merher, M.; Leutzsch, M.; Raut, R. K.; List, B. Catalytic Asymmetric Ionic Hydrogenation of alpha-Alkyl Styrenes. *J. Am. Chem. Soc.* **2025**, *147*, 31463-31469.
(8) Kolb, S.; Werz, D. B. Site-selective Hydrogenation/Deuteration of Benzylic Olefins Enabled by Electroreduction Using Water. *Chem. Eur. J.* **2023**, *29*, e202300849.
(9) Zhang, S.; Bedi, D.; Cheng, L.; Unruh, D. K.; Li, G.; Findlater, M. Cobalt(II)-Catalyzed Stereoselective Olefin Isomerization: Facile Access to Acyclic Trisubstituted Alkenes. *J. Am. Chem. Soc.* **2020**, *142*, 8910-8917.
(10) Leitch, D. C.; Labinger, J. A.; Bercaw, J. E. Scope and Mechanism of Homogeneous Tantalum/Iridium Tandem Catalytic Alkane/Alkene Upgrading using Sacrificial Hydrogen Acceptors. *Organometallics* **2014**, *33*, 3353-3365.
(11) Gesmundo, N. J.; Nicewicz, D. A. Cyclization-endoperoxidation cascade reactions of dienes mediated by a pyrylium photoredox catalyst. *Beilstein J. Org. Chem.* **2014**, *10*, 1272-1281.
(12) He, R. D.; Bai, Y.; Han, G. Y.; Zhao, Z. Z.; Pang, X.; Pan, X.; Liu, X. Y.; Shu, X. Z. Reductive Alkylation of Alkenyl Acetates with Alkyl Bromides by Nickel Catalysis. *Angew. Chem., Int. Ed. Engl.* **2022**, *61*, e202114556.
(13) Baldwin, S. W.; Wilson, J. D.; Aube, J. Regiochemistry in the intramolecular cycloadditions of substituted 5-alkenyl and 6-alkenyl nitrones. *J. Org. Chem.* **2002**, *50*, 4432-4439.
(14) Yus, M.; Torregrosa, R.; Pastor, I. M. Masked omega-lithio ester enolates: synthetic applications. *Molecules* **2004**, *9*, 330-348.
(15) Monasterolo, C.; Muller-Bunz, H.; Gilheany, D. G. Very short highly enantioselective Grignard synthesis of 2,2-disubstituted tetrahydrofurans and tetrahydropyrans. *Chem. Sci.* **2019**, *10*, 6531-6538.
(16) Ghosh, S.; Das, S.; De, C. K.; Yepes, D.; Neese, F.; Bistoni, G.; Leutzsch, M.; List, B. Strong and Confined Acids Control Five Stereogenic Centers in Catalytic Asymmetric Diels-Alder Reactions of Cyclohexadienones with Cyclopentadiene. *Angew. Chem., Int. Ed. Engl.* **2020**, *59*, 12347-12351.
(17) Xie, Y.; Cheng, G. J.; Lee, S.; Kaib, P. S.; Thiel, W.; List, B. Catalytic Asymmetric Vinylogous Prins Cyclization: A Highly Diastereo- and Enantioselective Entry to Tetrahydrofurans. *J. Am. Chem. Soc.* **2016**, *138*, 14538-14541.
(18) Dolomanov, O. V.; Bourhis, L. J.; Gildea, R. J.; Howard, J. A. K.; Puschmann, H. OLEX2: a complete structure solution, refinement and analysis program. *J. Appl. Crystallogr.* **2009**, *42*, 339-341.
(19) Sheldrick, G. M. SHELXT - integrated space-group and crystal-structure determination. *Acta Crystallogr. A Found Adv.* **2015**, *71*, 3-8.
(20) Bourhis, L. J.; Dolomanov, O. V.; Gildea, R. J.; Howard, J. A.; Puschmann, H. The anatomy of a comprehensive constrained, restrained refinement program for the modern computing environment - Olex2 dissected. *Acta Crystallogr. A Found Adv.* **2015**, *71*, 59-75.
(21) Staub, R.; Gantzer, P.; Harabuchi, Y.; Maeda, S.; Varnek, A. Challenges for Kinetics Predictions via Neural Network Potentials: A Wilkinson's Catalyst Case. *Molecules* **2023**, *28*.
(22) Staub, R.; Harabuchi, Y.; Seraphim, C.; Varnek, A.; Maeda, S. An Accurate and Efficient Reaction Path Search with Iteratively Trained Neural Network Potential: Answering the Passerini Mechanism Controversy. *J. Chem. Theory Comput.* **2026**, *22*, 422-440.
(23) Harabuchi, Y.; Staub, R.; Gao, M.; Tsuji, N.; List, B.; Varnek, A.; Maeda, S. Predicting Enantioselectivity via Kinetic Simulations on Gigantic Reaction Path Networks. *ACS Cent. Sci.* **2026**, *12*, 524-531.
(24) Maeda, S.; Harabuchi, Y.; Takagi, M.; Taketsugu, T.; Morokuma, K. Artificial Force Induced Reaction (AFIR) Method for Exploring Quantum Chemical Potential Energy Surfaces. *Chem. Rec.* **2016**, *16*, 2232-2248.
(25) Maeda, S.; Ohno, K.; Morokuma, K. Systematic exploration of the mechanism of chemical reactions: the global reaction route mapping (GRRM) strategy using the ADDF and AFIR methods. *Phys. Chem. Chem. Phys.* **2013**, *15*, 3683-3701.
(26) Anstine, D. M.; Zubatyuk, R.; Isayev, O. AIMNet2: a neural network potential to meet your neutral, charged,

organic, and elemental-organic needs. *Chem. Sci.* **2025**, *16*, 10228-10244.
(27) Neese, F. The ORCA program system. *WIREs Comput. Mol. Sci.* **2011**, *2*, 73-78.
(28) Neese, F. Software Update: The ORCA Program System—Version 6.0. *WIREs Comput. Mol. Sci.* **2025**, *15*.
(29) Froitzheim, T.; Müller, M.; Hansen, A.; Grimme, S. g-xTB: A General-Purpose Extended Tight-Binding Electronic Structure Method For the Elements H to Lr (Z=1–103). *ChemRxiv.* **2025**, https://doi.org/10.26434/chemrxiv-2025-bjxvt.
(30) Grimme, S.; Hansen, A.; Ehlert, S.; Mewes, J. M. r(2)SCAN-3c: A "Swiss army knife" composite electronic-structure method. *J. Chem. Phys.* **2021**, *154*, 064103.
(31) Marenich, A. V.; Cramer, C. J.; Truhlar, D. G. Universal solvation model based on solute electron density and on a continuum model of the solvent defined by the bulk dielectric constant and atomic surface tensions. *J. Phys. Chem. B* **2009**, *113*, 6378-6396.
(32) Weigend, F.; Ahlrichs, R. Balanced basis sets of split valence, triple zeta valence and quadruple zeta valence quality for H to Rn: Design and assessment of accuracy. *Phys. Chem. Chem. Phys.* **2005**, *7*, 3297-3305.
(33) Mardirossian, N.; Head-Gordon, M. omegaB97M-V: A combinatorially optimized, range-separated hybrid, meta-GGA density functional with VV10 nonlocal correlation. *J. Chem. Phys.* **2016**, *144*, 214110.
(34) Goddard, T. D.; Huang, C. C.; Meng, E. C.; Pettersen, E. F.; Couch, G. S.; Morris, J. H.; Ferrin, T. E. UCSF ChimeraX: Meeting modern challenges in visualization and analysis. *Protein Sci.* **2018**, *27*, 14-25.
(35) *Blender - a 3D modelling and rendering package.*; Stichting Blender Foundation, Amsterdam. Available at: http://www.blender.org.
(36) Peng, Q.; Duarte, F.; Paton, R. S. Computing organic stereoselectivity - from concepts to quantitative calculations and predictions. *Chem. Soc. Rev.* **2016**, *45*, 6093-6107.
(37) Bickelhaupt, F. M.; Houk, K. N. Analyzing Reaction Rates with the Distortion/Interaction-Activation Strain Model. *Angew. Chem., Int. Ed. Engl.* **2017**, *56*, 10070-10086.
